# Palladium-Catalyzed Tsuji–Trost-Type Reaction of 3-Indolylmethylacetates with O, and S Soft Nucleophiles

**DOI:** 10.3390/molecules29143434

**Published:** 2024-07-22

**Authors:** Antonia Iazzetti, Antonio Arcadi, Marco Chiarini, Giancarlo Fabrizi, Antonella Goggiamani, Federico Marrone, Andrea Serraiocco, Roberta Zoppoli

**Affiliations:** 1 Dipartimento di Scienze Biotecnologiche di Base, Cliniche Intensivologiche e Perioperatorie, Università Cattolica del Sacro Cuore, L. go Francesco Vito 1, 00168 Rome, RM, Italy; 2 Policlinico Universitario ‘A. Gemelli’ Foundation-IRCCS, 00168 Rome, RM, Italy; 3 Dipartimento di Scienze Fisiche e Chimiche, Università degli Studi di L’Aquila, Via Vetoio, 67100 Coppito, AQ, Italy; antonio.arcadi@univaq.it; 4 Dipartimento di Bioscienze e Tecnologie Agro-Alimentari e Ambientali, Università di Teramo, Via R. Balzarini, 64100 Teramo, TE, Italy; mchiarini@unite.it; 5 Dipartimento di Chimica e Tecnologie del Farmaco, Sapienza, Università di Roma, P. le A. Moro 5, 00185 Rome, RM, Italy; antonella.goggiamani@uniroma1.it (A.G.); federico.marrone@uniroma1.it (F.M.); andrea.serraiocco@uniroma1.it (A.S.); roberta.zoppoli@uniroma1.it (R.Z.)

**Keywords:** 3-carbinol, Tsuji–Trost-type reaction, O and S soft nucleophiles, renewable sources

## Abstract

The chemical valorization of widespread molecules in renewable sources is a field of research widely investigated in the last decades. In this context, we envisaged that indole-3-carbinol, present in different *Cruciferae* plants, could be a readily available building block for the synthesis of various classes of indoles through a palladium-catalyzed Tsuji–Trost-type reaction with O and S soft nucleophiles. The regiochemical outcome of this high-yielding functionalization shows that the nucleophilic substitution occurs only at the benzylic position. Interestingly, with this protocol, the sulfonyl unit could be appended to the indole nucleus, providing convenient access to new classes of molecules with potential bioactivity.

## 1. Introduction

Nowadays, the valorization of bioactive compounds found in natural sources is gaining high interest due to the growing trend of promoting the use of renewable resources following a circular biobased approach towards natural product-based drug discovery [1]. Significant efforts have been made to establish sustainable processes in which natural scaffolds are extracted and chemically modified using efficient catalytic methods, aiming to create molecules with improved biological properties [2].

One of the most common kinds of natural products of biological relevance is represented by indole-containing alkaloids, with more than 4100 examples. A large number of them have been deeply examined for their remarkable anticancer, antibacterial, antiviral, antifungal, and antiplasmodial activities, attracting significant attention as possible leads for novel therapies [3].

**Figure 1:**
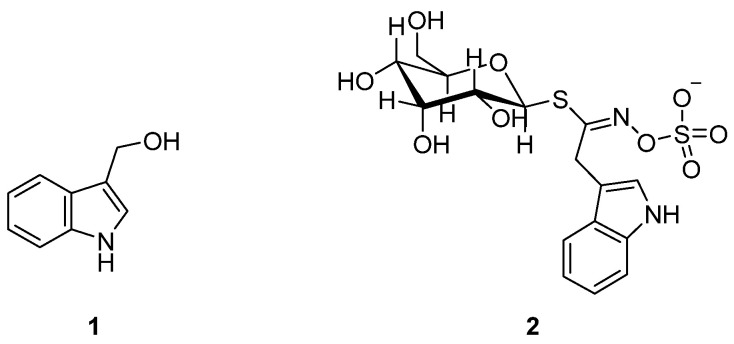
Indole-3-carbinol (I3C) **1** and glycobrassicin **2** structures.

In this regard, an excellent instance is represented by the indole-3-carbinol (I3C) **1** (Figure 1).

Particularly, it is a naturally occurring phytochemical found in the *Brassicaceae* species of cruciferous vegetables derived from the myrosinase-catalyzed hydrolysis of glycobrassicin **2** (Figure 1) [4]. I3C has been found to exhibit antioxidative, anti-inflammatory, antiatherogenic, antiviral, antithrombotic, and, most notably, anticarcinogenic activity [5,6,7,8,9,10,11,12,13,14,15,16].

Despite this broad spectrum of activities and its high therapeutic potential, many drawbacks, notably its metabolic instability, limit the I3C development in drug discovery; therefore, structural modifications of this nucleus remain a challenging and demanding research subject [17].

Synthetic transformations for the functionalization of the indole nucleus based on indole carbinole and its derivatives are described and widely employed. Among them, two main approaches are explored:

1. in situ generation under acidic or basic reaction conditions of highly unstable and reactive transient indole methides followed by the regioselective conjugated addition of nucleophiles [18,19]. This approach has been strictly correlated to in situ aza-o-QMs generation/nucleophile Michael-type [20,21];
2. Tsuji–Trost-type reaction. Indeed, analogous to electrophilic systems of heteroaromatics with extended π conjugation featuring carboxylate and carbonate leaving groups, activated carbinols can generate π-(η3 indolyl)–palladium electrophilic intermediates in the presence of Pd(0) in equilibrium with cationic π-(η1-indolyl)-palladium complexes [22].

**Scheme 1:**
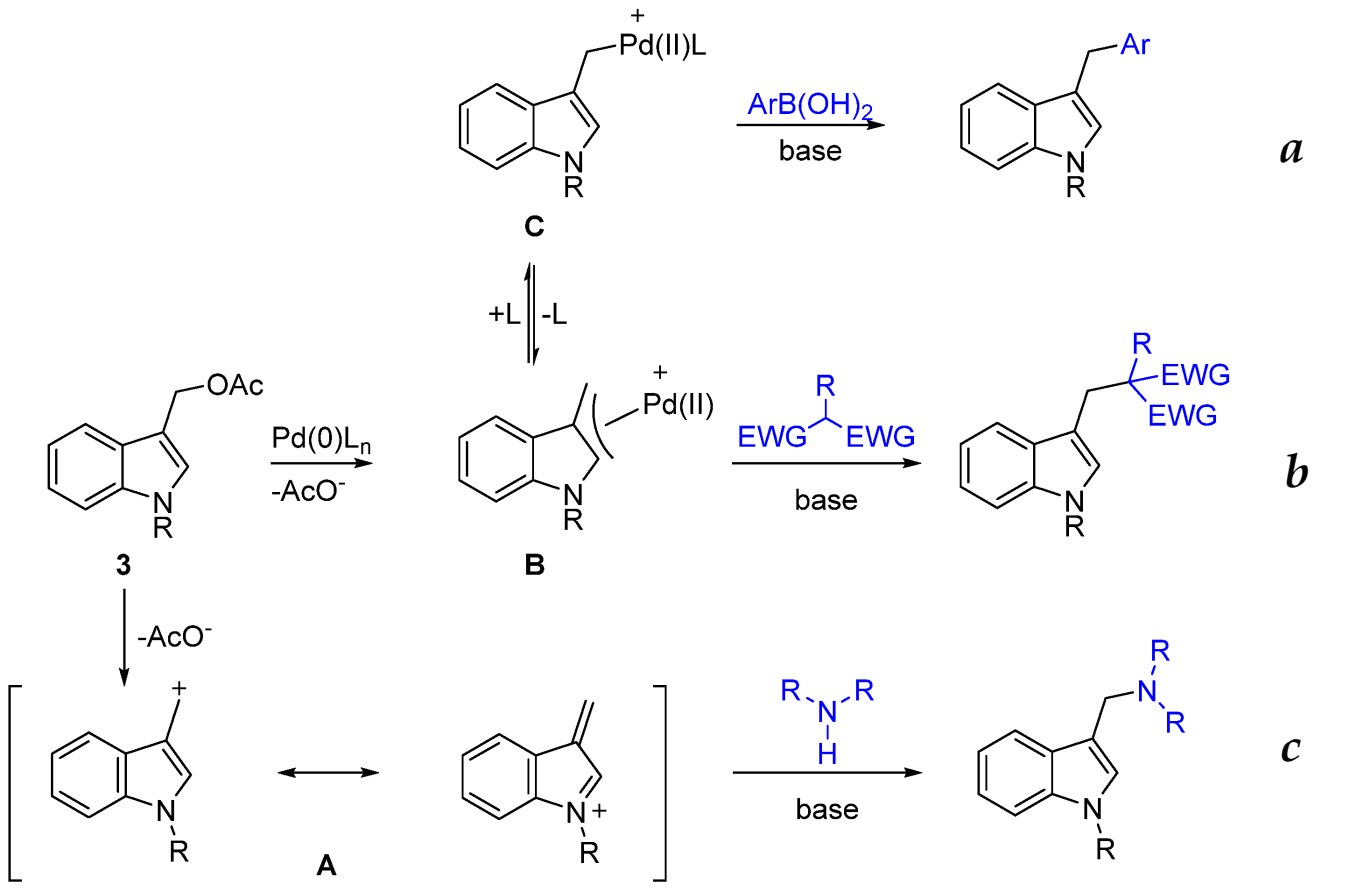
Our previous works on the reactivity of activated indole3carbinol (I3C). (**a**) [24] (**b**) [23] (**c**) [18].

Recently, part of our studies focused on the functionalization of indoles using activated carbinols as precursors of transient indole methides [18] or as substrates for palladium-catalyzed Tsuji–Trost-type reactions with different classes of carbon soft nucleophiles [23] and Suzuki–Miyaura cross-coupling with aryl boronic acids [24]. These synthetic approaches are summarized in Scheme 1.

**Figure 2:**
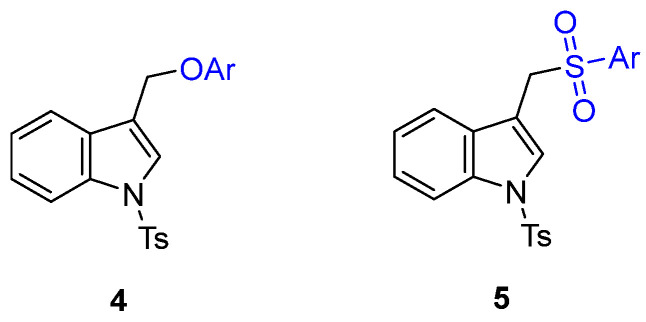
Structures of 1-substituted 3-(aryloxymethyl)-1*H*-indole **4** and 3-((arylsulfonyl)methyl)-1*H*-indole **5**.

**Scheme 2:**
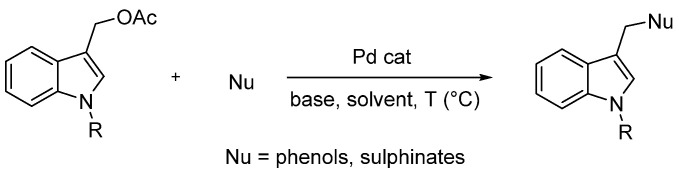
Working hypothesis.

However, since the functionalization of indole-3-carbinol with O and S soft nucleophiles was not explored, based on our background, we hypothesized that (1-substituted-indol-3-yl)methyl acetates **3** could be readily available precursors of two classes of indole-containing derivatives: 1-substituted 3-(aryloxymethyl)-1*H*-indole **4** and 3-((arylsulfonyl)methyl)-1*H*-indole **5** (Figure 2) through palladium-catalyzed Tsuji–Trost-type reactions with phenols or aryl sulfinates as soft nucleophiles, respectively (Scheme 2).

The aryloxy and arylsulfonyl groups attached to the indole scaffold appeared interesting to us allowing for structural modifications that can fine-tune pharmacological properties. This versatility is crucial for optimizing drug potency, selectivity, and pharmacokinetic properties.

To the best of our knowledge, the compounds **4** were synthesized for the first time by Yongxiang Liu from (1-tosyl-1*H*-indol-3-yl)methanol and phenols via Mitsunobu reaction with an approximate yield of 50% [25].

Hereafter we report the results of our investigation.

## 2. Results

The choice of (1-substituted-1*H*-indol-3-yl)methyl acetates **3** instead of (1*H*-indol-3-yl)methyl acetate as precursors for the functionalization of activated I3C with O and S soft nucleophiles is due to the low selectivity in N/O acetylation of the N-free I3C under different reaction conditions [18,23].

**Scheme 3:**
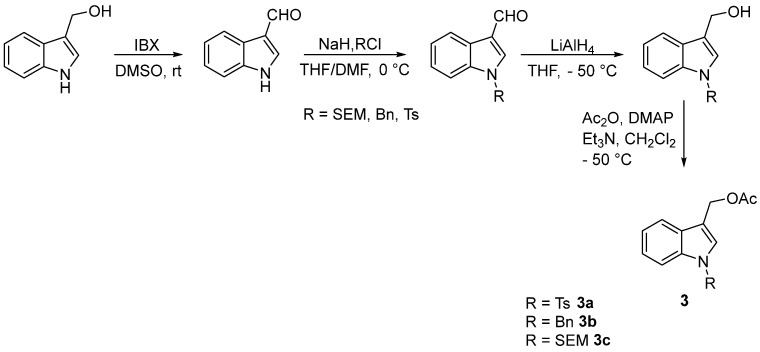
Synthesis of (1–substituted–indol–3−yl)methyl acetates **3**.

The (1-substituted-indol-3-yl)methyl acetates **3** were obtained with excellent overall yield from renewable sources I3C **1** according to the four-step sequence outlined in Scheme 3. Interestingly, the oxidation **1** to the corresponding 3-formylindole derivative was performed using IBX [26], a nonmetallic green oxidant with excellent recyclability [27]. Moreover, the reduction and acetylation reactions did not require any purification. 

The reaction of **3a**–**c** with 4-methoxyphenol **6a** was initially explored. Part of our optimization study using different ligands, solvents, and bases is summarized in Table 1.

Based on our previous results in analogs of gramine synthesis [18], we started our investigation by reacting the acetates **3a**–**b** with **6a** in the absence of palladium catalyst. No evidence of the substitution products **4aa** was observed (Table 1, entries 1–3). Slightly better results, but still unsatisfactory from a synthetic point of view, were obtained by switching to (1-SEM-1*H*-indol-3-yl)methyl acetate **3c** (Table 1, entries 4–5). These results suggested that the approach based on the sequential in situ generation under basic condition of indole-based iminium methide (A)/aza-Michael-type addition (Scheme 1) could not be a good strategy for synthesizing the target compounds. We then continued our screening in the presence of palladium catalysis, assuming that the Tsuji–Trost-type reaction could be a suitable protocol for converting **3c** into the corresponding **4ca**.

Taking advantage of our results in palladium-catalyzed benzylic-like nucleophilic substitution of benzofuran-2-ylmethyl acetates with S, O, and C soft nucleophiles [28], we thought that the neutral Pd(ally)LCl complexes [29,30] containing Buchwald dialkylmonophosphine ligands [31] could be highly effective precatalysts also in the conversion of **3c** to **4ca**. As reported by us, the active palladium Pd(ally)LCl complex could be generated in situ by dissolving [Pd(*η*^3^-C_3_H_5_)Cl]_2_ and Buchwald-type ligand in THF at room temperature. Initial attempts were based on the use of [Pd(*η*^3^-C_3_H_5_)Cl]_2_ as a source of palladium, XPhos as the ligand, MeCN as a solvent in the presence of different bases (Table 1, entries 6, 9–12) at 120 °C. The best results were obtained with K_2_CO_3_, having isolated **4ca** in 75% yield (Table 1, entry 6). Substitution of [Pd(*η*^3^-C_3_H_5_)Cl]_2_ with other sources of palladium [Pd_2_dba_3_ and Pd(PPh_3_)_4_] and XPhos with other ligands, keeping all other parameters the same, led to worse outcomes (Table 1, entries 7–8, 14–18). A poor yield of **4ca** was obtained by carrying out the reaction in DMF or DMSO instead of MeCN (Table 1, entries 19–20). In approximately all tests starting from **3c**, the *C*-alkylated compound **7ca** was isolated together with the expected *O*-alkylated main product **4ca**. Experimental results suggested that the O/C-alkylation ratio, usually affected by the degree of aggregation with bidentate anions, was influenced by several parameters such as the M^+^ size, solvation, and nature of the ligand. Eventually, with the optimized reaction conditions in hand ([Pd(C_3_H_5_)Cl]_2_, XPhos, MeCN/THF, K_2_CO_3_, 120 °C), we compared the reactivity of **3c** with **3b** and **3a**, and we were pleased to find that the desired final product was isolated in 97% yield starting from the *N*-Ts substrate (Table 1, entry 19).

We next explored the scope and generality of the reaction (Table 2).

Good to excellent results were usually obtained with various indoles and phenols. Phenols bearing neutral, electron-releasing, and electron-withdrawing substituents except for the nitro group (Table 2, entries 7 and 8) can be used. Furthermore, the presence of groups close to the C-OH bond does not hamper the reaction (Table 2, entries 3 and 13). Among tested indoles, (6-chloro-1-tosyl-1*H*-indol-3-yl)methyl acetate **3f** leads to a complex reaction mixture, probably for competitive cross-coupling reactions.

The potential of the developed strategy is further highlighted by the investigation of the palladium-catalyzed regioselective sulfonylation of (1-tosyl-1*H*-indol-3-yl)methyl acetates **3** with sulfinic acid salt **8**.

Even if the aryl sulfone fragment is present in several compounds exhibiting important biological activities [32,33,34], and many protocols have been developed for their synthesis [35,36,37,38,39,40,41,42,43], less attention has been devoted to the formation of 3-((arylsulfonyl)methyl)-1*H*-indole [44,45]. The sulfonylation of **3a** with commercially available sodium 4-methylbenzenesulfinate **8a** was attempted under the reaction conditions successfully employed with phenols. Lowering the reaction temperature to 100 °C, the indole **9aa** was isolated in 98% yield (Table 3, entry 2). Also with this class of soft nucleophiles, the formation of product **9** by a base-promoted reaction can be ruled out by recovering almost quantitatively the starting indole acetate **3a** under metal-free conditions (Table 3, entry 1). Subsequently, the protocol was extended to include functionalized indoles and arylsulfinates **8** (Table 3).

**Figure 3:**
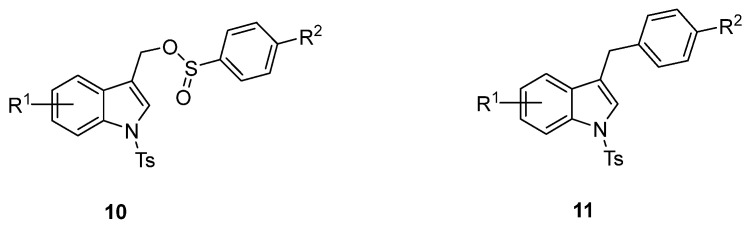
Structures of expected byproducts **10** and **11**.

**Figure 4:**
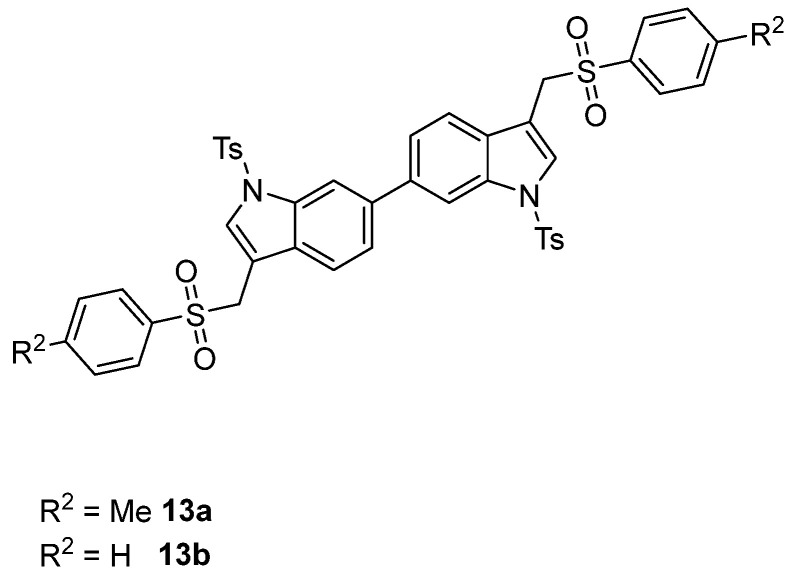
Structures of homocoupling byproducts **13a** and **13b**.

The formation of the (1-tosyl-1*H*-indol-3-yl)methyl arylsulfinate **10** resulting from the competitive O-attack of the ambident sulfinate anion as well as the desulfination product **11** (Figure 3) was never observed [46,47,48,49,50]. Interestingly, good yields were also obtained by using the (6-chloro-1-tosyl-1*H*-indol-3-yl)methyl acetate **3f** (Table 3, entries 8 and 9) along with a small amount of homocoupling byproduct **13** (Figure 4). Furthermore, the presence of nitro and methoxy on the indole nucleus as substituents was well tolerated (Table 3, entries 4, 5, 10, 11).

## 3. Discussion

**Scheme 4:**
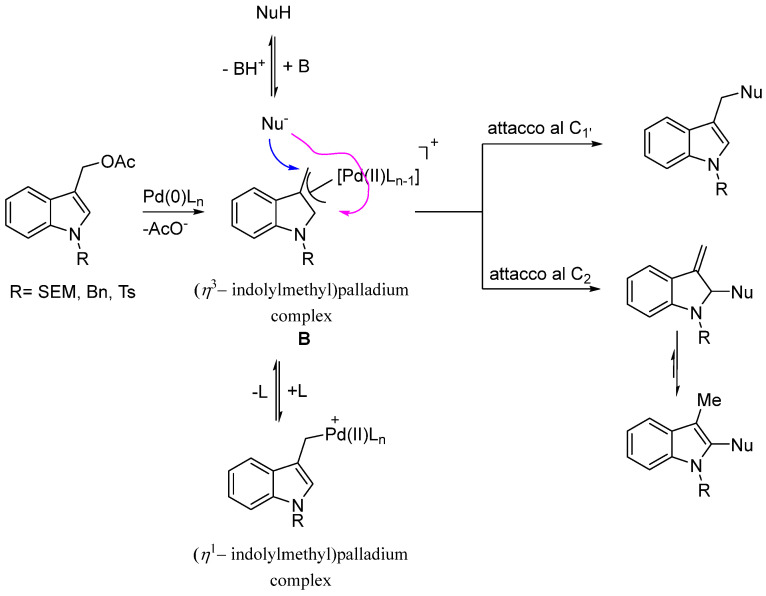
Formation of the (*η*^3^–indolylmethyl)palladium complex **B** and its reaction with nucleophiles.

The regioselective outcome of the functionalization of the 1-substituted-indol-3-yl)methyl acetates **3** with S and O soft nucleophiles represents the principal goal of our investigation. It is known that this type of substrate could generate the (*η*^3^-indolylmethyl)palladium complex **B** which undergoes the nucleophilic attack of the added nucleophile at the benzylic carbon C_1′_ or C_2_ position of the indole ring (Scheme 4).

In all the tested cases, under our reaction conditions, regardless of the nature of the nucleophiles, the reaction led only to the formation of the C_1′_ substituted products in high overall yield.

## 4. Conclusions

In conclusion, I3C represents a readily available building block for synthesizing highly desirable biologically active indole-containing sulfone and aryloxy fragments. The procedure is of wide scope, tolerates a variety of functional groups, and proceeds in yields ranging from good to excellent in a regioselective manner.

In addition, given the highlighted efficiency and atom economy, the proposed method may represent a reasonable valorization route for the exploitation of I3C and/or its derivative obtained from renewable sources.

## 5. Materials and Methods

### 5.1. General Information

All of the commercially available reagents, catalysts, bases, and solvents were used as purchased, without further purification. Starting materials and reaction products were purified by flash chromatography using SiO_2_ as stationary phase, eluting with *n*-hexane/ethyl acetate (EtOAc) mixtures. ^1^H NMR (400.13 MHz), ^13^C NMR (100.6 MHz), and ^19^F spectra (376.5 MHz) were recorded with a Bruker Avance 400 spectrometer. Splitting patterns were designed as s (singlet), d (doublet), t (triplet), q (quartet), m (multiplet), or bs (broad singlet). HRMS of samples were recorded using a MALDI-TOF spectrometer AB SCIEX TOF/TOF 5800 using matrix in combination with KI for the ionization, with an Orbitrap Exactive (Thermo Fisher, Norristown, PA, USA) mass spectrometer with ESI source positive as well negative. Melting points were determined with a Büchi B-545 apparatus and are uncorrected.

### 5.2. General Experimental Procedures

Starting materials **3a**–**c** were prepared according to the literature procedures [18,19,20,22], through the four-step sequence of reactions depicted in Scheme 2. Starting materials **3d**, **f**, **g** were obtained from the corresponding commercially available 3-formylindole. Experimental procedures for **3e** are detailed in Supplementary Materials.

#### 5.2.1. Typical Procedure for the Preparation of 1-Substituted 3-(aryloxymethyl)-1*H*-indole 4: 3-((4-methoxyphenoxy)methyl)-1-tosyl-1*H*-indole **4aa**

In a 50 mL Carousel Tube Reactor (Radely Discovery Technology) containing a magnetic stirring bar, [Pd(η^3^-C_3_H_5_)Cl]_2_ (2.6 mg, 0.007 mmol, 0.025 equiv.) and XPhos (6.7 mg, 0.014 mmol, 0.05 equiv.) were dissolved at room temperature with 0.5 mL of anhydrous THF under argon. Then, (1-tosyl-1*H*-indol-3-yl)methyl acetate **3a** (100.0 mg, 0.290 mmol, 1.00 equiv.), 4-methoxyphenol 6a (71.9 mg, 0.580 mmol, 2.00 equiv.), K_2_CO_3_ (80.3 mg, 0.580 mmol, 2.00 equiv.), and 2.0 mL of anhydrous MeCN were added, and the resulting mixture was stirred for 1.5 h at 120 °C under argon. After this time, the reaction mixture was cooled to room temperature, diluted with Et_2_O, extracted twice with NaOH 2.0 N, and then washed with brine. The organic layer was dried over Na_2_SO_4,_ iltered, and concentrated under reduced pressure. The residue was purified by chromatography on SiO_2_ (25–40 μm), eluting with 80/20 (*v*/*v*) *n*-hexane/AcOEt mixture (R*_f_* = 0.24) to obtain 3-((4-methoxyphenoxy)methyl)-1-tosyl-1*H*-indole **4aa** (97% yield, 114.5 mg).

3-((4-methoxyphenoxy)methyl)-1-tosyl-1*H*-indole **4aa**: 97% yield; yellow solid; mp: 132–133 °C; R*_f_* = 0.19 (*n*-hexane/AcOEt, 90:10); IR (neat): 2919, 1694, 1447, 1357, 1176 cm^−1^; ^1^H NMR (400.13 MHz) (CDCl_3_): δ 7.95 (d, *J* = 8.3 Hz, 1H), 7.70 (d, *J* = 8.3 Hz, 2H), 7.58 (s, 1H), 7.57 (d, *J* = 7.6 Hz, 1H), 7.31 (td, *J*_1_ = 7.8 Hz, *J*_2_ = 0.8 Hz, 1H), 7.23 (td, *J*_1_ = 7.8 Hz, *J*_2_ = 0.6 Hz, 1H), 7.17 (d, *J* = 8.2 Hz, 2H), 6.88 (d, *J* = 9.1 Hz, 2H), 6.80 (d, *J* = 9.1 Hz, 2H), 5.10 (s, 2H), 3.75 (s, 3H), 2.31 (s, 3H); ^13^C NMR (100.6 MHz) (CDCl_3_): δ 154.3 (C), 152.7 (C), 145.1 (C), 135.4 (C), 135.2 (C), 130.0 (CH), 129.7 (C), 127.0 (CH), 125.1 (CH), 125.0 (CH), 123.5 (CH), 120.1 (CH), 118.7 (C), 116.2 (CH), 114.8 (CH), 113.8 (CH), 63.3 (CH_2_), 55.8 (CH_3_), 27.7 (CH_3_); HRMS: *m/z* (MALDI-TOF) positive ion, calculated for C_23_H_21_KNO_4_S: [M+K]^+^ 446.0828, found: 446.0833.

#### 5.2.2. Typical Procedure for the Preparation of 1-tosyl 3-((arylsulfonyl)methyl)-1-tosyl-1*H*-indole 9: Synthesis of 1-tosyl-3-(tosylmethyl)-1*H*-indole **9aa**

In a 50 mL Carousel Tube Reactor (Radely Discovery Technology) containing a magnetic stirring bar, [Pd(η^3^-C_3_H_5_)Cl]_2_ (2.6 mg, 0.007 mmol, 0.025 equiv.) and XPhos (6.7 mg, 0.014 mmol, 0.05 equiv.) were dissolved at room temperature with 0.5 mL of anhydrous THF under argon. Then, (1-tosyl-1*H*-indol-3-yl)methyl acetate **3a** (100.0 mg, 0.290 mmol, 1.00 equiv.), sodium 4-tolylsulfinate **8a** (103.3 mg, 0.580 mmol, 2.00 equiv.), K_2_CO_3_ (80.3 mg, 0.580 mmol, 2.00 equiv.), and 2.0 mL of anhydrous MeCN were added, and the mixture was stirred for 1.5 h at 100 °C. After this time, the reaction mixture was cooled to room temperature, diluted with Et_2_O, and washed with brine. The organic layer was dried over Na_2_SO_4_, filtered, and concentrated under reduced pressure. The residue was purified by chromatography on SiO_2_ (25–40 μm), eluting with 80/20 (*v*/*v*) *n*-hexane/AcOEt mixture (R*_f_* = 0.24) to obtain 1-tosyl-3-(tosylmethyl)-1*H*-indole **9aa**.

1-tosyl-3-(tosylmethyl)-1*H*-indole **9aa**: 98% yield; red solid; mp: 207–208 °C; R*_f_* = 0.24 (*n*-hexane/AcOEt, 80:20); IR (neat): 2927, 1598, 1309, 1163, 659 cm^−1^; ^1^H NMR (400.13 MHz) (CDCl_3_): δ 7.95 (d, *J* = 8.2 Hz, 1H), 7.75 (d, *J* = 8.4 Hz, 2H), 7.46 (d, *J* = 8.2 Hz, 2H), 7.39 (s, 1H), 7.32 (d, *J* = 7.7 Hz, 2H), 7.29–7.26 (m, 2H), 7.19–7.15 (m, 1H), 7.13 (d, *J* = 8.2 Hz, 2H), 4.43 (s, 2H), 2.40 (s, 3H), 2.39 (s, 3H); ^13^C NMR (100.6 MHz) (CDCl_3_): δ 145.3 (C), 144.9 (C), 135.0 (C), 134.7 (C), 130.0 (overlapping) (CH), 129.7 (C), 129.6 (CH), 128.5 (CH), 127.3 (CH), 126.9 (CH), 125.1 (CH), 123.5 (CH), 119.6 (CH), 113.5 (CH), 110.1 (C), 53.6 (CH_2_), 21.6 (overlapping) (CH_3_); HRMS: *m/z* (MALDI-TOF) positive ion, calculated for C_23_H_21_KNO_4_S_2_: [M+K]^+^ 478.0549, found: 478.0554.

### 5.3. Characterization Data of Synthesized Compounds

Characterization data of starting materials **3a**–**g** are reported in Supplementary Materials.

#### Characterization Data of Final Compounds **4aa**–**ed** and **9aa**–**gb**

*1-benzyl-3-((4-methoxyphenoxy)methyl)-1H-indole* **4ba**: 57% yield; yellow oil; R*_f_* = 0.19 (*n*-hexane/AcOEt, 90:10); IR (neat): 2879, 1678, 1435, 1267, 1098, 895 cm^−1^; ^1^H NMR (400.13 MHz) (CDCl_3_): δ 7.62 (d, *J* = 7.7 Hz, 1H), 7.20–6.98 (m, 10H), 6.86 (d, *J* = 9.2 Hz, 2H), 6.75 (d, *J* = 9.2 Hz, 2H), 6.80 (d, *J* = 9.1 Hz, 2H), 5.16 (s, 2H), 5.09 (s, 2H), 3.66 (s, 3H); ^13^C NMR (100.6 MHz) (CDCl_3_): δ 154.0 (C), 153.3 (C), 137.3 (C), 137.0 (C), 128.9 (CH), 128.0 (CH), 127.7 (CH), 127.0 (CH), 122.3 (CH), 119.9 (CH), 119.5 (CH), 116.2 (CH), 114.7 (CH), 114.7 (CH), 110. 0 (CH), 63.3 (CH_2_), 55.8 (CH_3_), 50.1 (CH_3_); HRMS: *m/z* (MALDI-TOF) positive ion, calculated for C_23_H_21_NNaO_2_: [M+Na]^+^ 366.1470, found: 366.1472.

*2-((1-benzyl-1H-indol-3-yl)methyl)-4-methoxyphenol* **7ba**: 13% yield; brown oil; R*_f_* = 0.19 (*n*-hexane/AcOEt, 90:10); IR (neat): 2919, 1596, 1347, 1256, 1177, 989 cm^−1^; ^1^H NMR (400.13 MHz) (CDCl_3_): δ 7.49 (d, *J* = 8.2 Hz, 1H), 7.23–7.17 (m, 4H), 7.10 (td, *J_1_* = 7.5 Hz, *J_2_* = 0.8 Hz, 1H), 7.03–7.00 (m, 3H), 6.84 (s, 2H), 6.75 (d, *J* = 2.9 Hz, 1H), 6.80 (d, *J* = 8.7 Hz, 1H), 6.62 (dd, *J_1_* = 8.7 Hz, *J_2_* = 2.9 Hz, 1H), 5.16 (s, 2H), 5.18 (s, 2H), 4.64 (bs, 1H), 4.02 (s, 2H), 3.67 (s, 3H); ^13^C NMR (100.6 MHz) (CDCl_3_): δ 153.8 (C), 148.4 (C), 137.6 (C), 137.2 (C), 128.9 (CH), 128.0 (CH), 127.7 (CH), 127.6 (C), 126.8 (C), 126.6 (CH), 122.4 (CH), 119.54 (CH), 119.49 (CH), 116.8 (CH), 116.3 (CH), 112.6 (CH), 112.5 (C), 110. 0 (CH), 55.8 (CH_3_), 50.1 (CH_2_), 27.4 (CH_2_); HRMS: *m/z* (MALDI-TOF) positive ion, calculated for C_23_H_21_NNaO_2_: [M+Na]^+^ 366.1470, found: 366.1473.

*3-((4-methoxyphenoxy)methyl)-1-((2-(trimethylsilyl)ethoxy)methyl)-1-H-indole* **4ca:** 97% yield; yellow solid; mp: 111–113 °C; R*_f_* = 0.23 (*n*-hexane/AcOEt, 80:20); IR (neat): 3030, 2789, 1447, 1327, 1145 cm^−1^; ^1^H NMR (400.13 MHz) (CDCl_3_): δ 7.75 (d, *J* = 7.9 Hz, 1H), 7.70 (d, *J* = 8.0 Hz, 1H), 7.31 (td, *J_1_* = 7.0 Hz, *J_2_* = 0.9 Hz, 1H), 7.28–7.21 (m, 2H), 7.03–6.99 (m, 2H), 6.91–6.87 (m, 2H), 5.49 (s, 2H), 5.24 (s, 2H), 3.81 (m, 3H), 3.52 (t, *J* = 8.1 Hz, 2H), 0.93 (t, *J* = 8.1 Hz, 1H), 0.00 (s, 9H); ^13^C NMR (100.6 MHz) (CDCl_3_): δ 154.0 (C), 153.3 (C), 137.0 (C), 128.0 (C), 127.5 (CH), 122.7 (CH), 120.5 (CH), 119.5 (CH), 116.1 (CH), 114.7 (CH), 112.2 (C), 110.2 (CH), 75.7 (CH_2_), 66.0 (CH_2_), 63.3 (CH_2_), 55.8 (CH_3_), 17.8 (CH_2_), −1.33 (CH_3_); HRMS: *m/z* (MALDI-TOF) positive ion, calculated for C_22_H_29_KNO_3_Si: [M+K]^+^ 422.1554, found: 422.1554.

*4-methoxy-2-((1-((2-(trimethylsilyl)ethoxy)methyl)-1H-indol-3-yl)methyl)phenol* **7ca**: 97% yield; yellow oil; R*_f_* = 0.21 (*n*-hexane/AcOEt, 80:20); IR (neat): 2988, 1567, 1347, 1278, 1165, 982 cm^−1^; ^1^H NMR (400.13 MHz) (CDCl_3_): δ 7.57 (d, *J* = 8.0 Hz, 1H), 7.48 (d, *J* = 8.0 Hz, 1H), 7.25 (dd, *J_1_* = 8.1 Hz, *J_2_* = 0.9 Hz, 1H), 7.15 (dd, *J_1_* = 7.5 Hz, *J_2_* = 0.7 Hz, 1H), 6.99 (s, 1H), 6.82 (d, *J* = 2.9 Hz, 1H), 6.78 (d, *J* = 8.7 Hz, 1H), 6.72 (dd, *J_1_* = 8.7 Hz, *J_2_* = 2.9 Hz, 1H), 5.43 (s, 2H), 4.65 (bs, 1H), 4.09 (s, 2H), 3.76 (m, 3H), 3.45 (t, *J* = 8.1 Hz, 2H), 0.88 (t, *J* = 8.1 Hz, 1H), 0.00 (s, 9H); ^13^C NMR (100.6 MHz) (CDCl_3_): δ 148.0 (C), 137.2 (C), 128.4 (C), 127.8 (CH), 126. 3 (CH), 122.7 (CH), 120.5 (C), 120.1 (CH), 119.4 (CH), 116.7 (CH), 116.4 (CH), 113.6 (C), 112.6 (CH), 110 (CH), 75.4 (CH_2_), 66.0 (CH_2_), 63.3 (CH_2_), 55.8 (CH_3_), 26.9 (CH) 17.8 (CH_2_), −1.39 (CH_3_); HRMS: *m/z* (MALDI-TOF) positive ion, calculated for C_22_H_29_KNO_3_Si: [M+K]^+^ 422.1554, found: 422.1554.

*3-((4-(tert-butyl)phenoxy)methyl)-1-tosyl-1H-indole* **4ab:** 83% yield; yellow solid; mp: 126–128 °C; R*_f_* = 0.19 (*n*-hexane/AcOEt, 90:10); IR (neat): 2957, 1596, 1361, 1215, 1100 cm^−1^; ^1^H NMR (400.13 MHz) (CDCl_3_): δ 7.98 (d, *J* = 8.2 Hz, 1H), 7.75 (d, *J* = 8.3 Hz, 2H), 7.62 (s, 1H), 7.59 (d, *J* = 7.8 Hz, 1H), 7.36–7.28 (m, 3H), 7.27–7.24 (m, 1H), 7.23–7.18 (m, 2H), 6.92 (d, *J* = 8.8 Hz, 2H), 5.15 (s, 2H), 2.33 (s, 3H), 1.31 (s, 9H); ^13^C NMR (100.6 MHz) (CDCl_3_): δ 154.3 (C), 152.7 (C), 145.1 (C), 135.4 (C), 135.2 (C), 130.0 (CH), 129.7 (C), 127.0 (CH), 125.1 (CH), 125.0 (CH), 123.5 (CH), 120.1 (CH), 118.7 (C), 116.2 (CH), 114.8 (CH), 113.8 (CH), 63.3 (CH_2_), 55.8 (CH_3_), 27.7 (CH_3_); HRMS: *m/z* (MALDI-TOF) positive ion, calculated for C_26_H_27_KNO_3_S: [M+K]^+^ 472.1349, found: 472.1349.

*3-((4-fluorophenoxy)methyl)-1-tosyl-1H-indole*  **4ac:** 81% yield; white solid; mp: 126–127 °C; R*_f_* = 0.19 (*n*-hexane/AcOEt, 90:10); IR (neat): 2957, 1596, 1474, 1366, 1172 cm^−1^; ^1^H NMR (400.13 MHz) (CDCl_3_): δ 7.98 (d, *J* = 8.3 Hz, 1H), 7.73 (d, *J* = 8.3 Hz, 2H), 7.60 (s, 1H), 7.59 (d, *J* = 7.4 Hz, 1H), 7.34 (td, *J_1_* = 7.7 Hz, *J_2_* = 0.9 Hz, 1H), 7.25 (td, *J_1_* = 8.4 Hz, *J_2_* = 0.8 Hz, 1H), 7.20 (d, *J* = 8.1 Hz, 2H), 6.98–6.87 (m, 4H), 5.14 (s, 2H), 2.34 (s, 3H); ^13^C NMR (100.6 MHz) (CDCl_3_): δ 157.6 (d, *J_CF_* = 240.0 Hz, C), 154.60 (d, *J_CF_* = 2.2 Hz, C), 145.2 (C), 135.4 (C), 135.2 (C), 130.0 (CH), 129.6 (C), 126.9 (CH), 125.1 (d, *J_CF_* = 20.4 Hz, CH), 123.6 (CH), 120.0 (CH), 118.3 (C), 116.3 (d, *J_CF_* = 8.0 Hz, CH), 116.1 (CH), 115.9 (CH), 113.9 (CH), 63.2 (CH_2_), 21.7 (CH_3_); HRMS: *m/z* (MALDI-TOF) positive ion, calculated for C_22_H_19_FNO_3_S: [M+H]^+^ 396.1070, found: 396.1071.

*3-(([1,1′-biphenyl]-2-yloxy)methyl)-1-tosyl-1H-indole* **4ad**: 80% yield; white solid; mp: 125–127 °C; R*_f_* = 0.22 (*n*-hexane/AcOEt, 90:10); IR (neat): 2957, 1596, 1443, 1281, 1118, 1053, cm^−1^; ^1^H NMR (400.13 MHz) (CDCl_3_): δ 7.96 (d, *J* = 8.3 Hz, 1H), 7.67 (d, *J* = 8.2 Hz, 2H), 7.51 (d, *J* = 7.1 Hz, 2H), 7.47 (s, 1H), 7.43–7.27 (m, 7H), 7.20–7.15 (m, 3H), 7.12–7.06 (m, 2H), 5.15 (s, 2H), 2.32 (s, 3H); ^13^C NMR (100.6 MHz) (CDCl_3_): δ 155.4 (C), 145.0 (C), 138.5 (C), 135.4 (C), 135.3 (C), 132.0 (C), 131.1 (CH), 130.0 (CH), 129.7 (CH), 129.4 (C), 128.7 (CH), 128.1 (CH), 127.1 (CH), 126.9 (CH), 125.0 (CH), 124.8 (CH), 123.4 (CH), 121.9 (CH), 119.9 (CH), 118.9 (C), 113.9 (CH), 113.8 (CH), 63.5 (CH_2_), 21.7 (CH_3_); HRMS: *m/z* (MALDI-TOF) positive ion, calculated for C_28_H_23_KNO_3_S: [M+K]^+^ 492.1036, found: 492.1038.

*methyl 3-((1-tosyl-1H-indol-3-yl)methoxy)benzoate* **4ae**: 83% yield; pale-yellow solid; mp: 128–130 °C; R*_f_* = 0.19 (*n*-hexane/AcOEt, 90:10); IR (neat): 2957, 1714, 1509, 1367, 1099, 998 cm^−1^; ^1^H NMR (400.13 MHz) (CDCl_3_): δ 7.95 (d, *J* = 8.3 Hz, 1H), 7.70 (d, *J* = 8.3 Hz, 2H), 7.58 (s, 1H), 7.57 (d, *J* = 7.6 Hz, 1H), 7.31 (td, *J_1_* = 7.8 Hz, *J_2_* = 0.9 Hz, 1H), 7.23 (td, *J_1_* = 7.8 Hz, *J_2_* = 0.6 Hz, 1H), 7.17 (d, *J* = 8.3 Hz, 2H), 6.88 (d, *J* = 9.1 Hz, 2H), 6.80 (d, *J* = 9.1 Hz, 2H), 5.10 (s, 2H), 3.75 (s, 3H), 2.31 (s, 3H); ^13^C NMR (100.6 MHz) (CDCl_3_): δ 154.3 (C), 152.7 (C), 145.1 (C), 135.4 (C), 135.2 (C), 130.0 (CH), 129.7 (C), 127.0 (CH), 125.1 (CH), 125.0 (CH), 123.5 (CH), 120.1 (CH), 118.7 (C), 116.2 (CH), 114.8 (CH), 113.8 (CH), 63.3 (CH_2_), 55.8 (CH_3_), 27.7 (CH_3_); HRMS: *m*/*z* (MALDI-TOF) positive ion, calculated for C_24_H_21_KNO_5_S: [M+K]^+^ 474.0778, found: 474.0779.

*4-((1-tosyl-1H-indol-3-yl)methoxy)benzonitrile* **4af**: 75% yield; yellow solid; mp: 142–144 °C; R*_f_* = 0.25 (*n*-hexane/AcOEt, 90:10); IR (neat): 2957, 1509, 1216, 1083, 1032, 971 cm^−1^; ^1^H NMR (400.13 MHz) (CDCl_3_): δ 8.01 -7.99 (m, 2H), 7.75 (d, J = 8.2 Hz, 2H), 7.64 (s, 1H), 7.60–7.56 (m, 3H), 7.30–7.22 (m, 3H), 7.38–7.34 (m, 1H), 7.29–7.25 (m, 1H), 7.22 (d, *J* = 8.2 Hz, 2H), 7.02 (d, *J* = 8.8 Hz, 2H) 5.23 (s, 2H), 2.35 (s, 3H); ^13^C NMR (100.6 MHz) (CDCl_3_): δ 161.8 (C), 145.4 (C), 135.4 (C), 135.2 (C), 134.2 (CH), 130.1 (CH), 129.3 (C), 127.0 (CH), 125.4 (CH), 125.2 (CH), 123.7 (CH), 119.9 (CH), 119.2 (C), 117.2 (C), 115.7 (CH), 113.9 (CH), 104.6 (C), 62.8 (CH_2_), 21.7 (CH_3_); HRMS: *m/z* (MALDI-TOF) positive ion, calculated for C_23_H_19_N_2_O_3_S: [M+H]^+^ 403.1116, found: 403.1119.

*5-methoxy-3-((4-methoxyphenoxy)methyl)-1-tosyl-1H-indole* **4da:** 83% yield; yellow solid; mp: 108–110 °C; R*_f_* = 0.19 (*n*-hexane/AcOEt, 90:10); IR (neat): 2955, 1596, 1366, 1082, 972 cm^−1^; ^1^H NMR (400.13 MHz) (CDCl_3_): δ 7.88 (d, *J* = 9.0 Hz, 1H), 7.70 (d, *J* = 8.3 Hz, 2H), 7.56 (s, 1H), 7.18 (d, *J* = 8.2 Hz, 2H), 7.03 (d, *J* = 2.3 Hz, 1H), 6.95 (dd, *J*_1_ = 9.0 Hz, *J*_2_ = 2.4 Hz, 1H), 6.91 (d, *J* = 9.0 Hz, 2H), 6.83 (d, *J* = 9.1 Hz, 2H), 5.09 (s, 2H), 3.80 (s, 3H), 3.78 (s, 3H); ^13^C NMR (100.6 MHz) (CDCl_3_): δ 154.3 (C), 152.7 (C), 145.1 (C), 135.4 (C), 135.2 (C), 130.0 (CH), 129.7 (C), 127.0 (CH), 125.1 (CH), 125.0 (CH), 123.5 (CH), 120.1 (CH), 118.7 (C), 116.2 (CH), 114.8 (CH), 113.8 (CH), 63.3 (CH_2_), 55.8 (CH_3_), 27.7 (CH_3_); HRMS: *m*/*z* (MALDI-TOF) positive ion, calculated for C_24_H_23_KNO_5_S: [M+K]^+^ 476.0934, found: 476.0931.

*3-((4-(tert-butyl)phenoxy)methyl)-5-methoxy-1-tosyl-1H-indole* **4db**: 97% yield; orange solid; mp: 122–124 °C; R*_f_* = 0.22 (*n*-hexane/AcOEt, 90:10); IR (neat): 2965, 2865, 1509, 1215, 1032, 973 cm^−1^; ^1^H NMR (400.13 MHz) (CDCl_3_): δ 7.87 (d, *J* = 9.0 Hz, 1H), 7.73 (d, *J* = 8.3 Hz, 2H), 7.59 (s, 1H), 7.33 (d, *J* = 8.8 Hz, 2H), 7.20 (d, *J* = 8.2 Hz, 2H), 7.02 (d, *J* = 2.3 Hz, 1H), 7.73 (d, *J* = 8.3 Hz, 2H), 7.98–6.91 (m, 3H), 5.12 (s, 2H), 3.80 (s, 3H), 2.34 (s, 3H), 1.32 (s, 9H); ^13^C NMR (100.6 MHz) (CDCl_3_): 156.6 (C), 156.4 (C), 145.0 (C), 144.1 (C), 135.3 (C), 130.8 (C), 130.1 (C), 130.0 (CH), 126.9 (CH), 126.4 (CH), 125.7 (CH), 118.7 (C), 114.7 (CH), 114.4 (CH), 114.4 (CH), 102.4 (CH), 62.5 (CH_2_), 55.8 (CH_3_), 34.2 (C), 31.6 (CH_3_), 21.7 (CH_3_). HRMS: *m/z* (MALDI-TOF) positive ion, calculated for C_27_H_29_KNO_4_S: [M+K]^+^ 502.1454, found: 502.1452.

*3-(([1,1′-biphenyl]-2-yloxy)methyl)-5-methoxy-1-tosyl-1H-indole*  **4dc**: 83% yield; yellow solid; mp: 132–135 °C; R*_f_* = 0.19 (*n*-hexane/AcOEt, 90:10); IR (neat): 2975, 1596, 1475, 1294, 1055, 895 cm^−1^; ^1^H NMR (400.13 MHz) (CDCl_3_): δ 7.83 (d, *J* = 9.1 Hz, 1H), 7.64 (d, *J* = 8.3 Hz, 2H), 7.49 (dd, *J_1_* = 8.3 Hz, *J_2_* = 1.4 Hz, 2H), 7.43 (s, 1H), 7.38–7.27 (m, 5H), 7.16 (d, *J* = 8.1 Hz, 2H), 7.08 (dd, *J_1_* = 7.8 Hz, *J_2_* = 5.4 Hz, 2H), 6.89 (dd, *J_1_* = 9.0 Hz, *J_2_* = 2.4 Hz, 1H), 6.82 (d, *J* = 2.4 Hz, 1H), 5.12 (s, 2H), 3.68 (s, 3H), 2.32 (s, 3H); ^13^C NMR (100.6 MHz) (CDCl_3_): δ 154.3 (C), 152.7 (C), 145.1 (C), 135.4 (C), 135.2 (C), 130.0 (CH), 129.7 (C), 127.0 (CH), 125.1 (CH), 125.0 (CH), 123.5 (CH), 120.1 (CH), 118.7 (C), 116.2 (CH), 114.8 (CH), 113.8 (CH), 63.3 (CH_2_), 55.8 (CH_3_), 27.7 (CH_3_); HRMS: *m/z* (MALDI-TOF) positive ion, calculated for C_29_H_25_KNO_4_S: [M+K]^+^ 522.1141, found: 522.1138.

*Methyl 3-((5-methoxy-1-tosyl-1H-indol-3-yl)methoxy)benzoate*  **4de:** 83% yield; yellow solid; mp: 136–138 °C; R*_f_* = 0.19 (*n*-hexane/AcOEt, 90:10); IR (neat): 2956, 1716, 1595, 1475, 1032, 754 cm^−1^; ^1^H NMR (400.13 MHz) (CDCl_3_): δ 7.80 (d, *J* = 9.0 Hz, 1H), 7.63 (d, *J* = 8.3 Hz, 2H), 7.61–7.57 (m, 2H), 7.53 (s, 1H), 7.27 (t, *J* = 8.1 Hz, 1H), 7.13–7.06 (m, 3H), 6.93 (d, *J* = 2.4 Hz, 1H), 6.87 (d, *J*_1_ = 9.1 Hz, *J*_2_ = 2.4 Hz, 2H), 5.11 (s, 2H), 3.84 (s, 3H), 3.73 (s, 3H), 2.25 (s, 3H); ^13^C NMR (100.6 MHz) (CDCl_3_): δ 154.3 (C), 152.7 (C), 145.1 (C), 135.4 (C), 135.2 (C), 130.0 (CH), 129.7 (C), 127.0 (CH), 125.1 (CH), 125.0 (CH), 123.5 (CH), 120.1 (CH), 118.7 (C), 116.2 (CH), 114.8 (CH), 113.8 (CH), 63.3 (CH_2_), 55.8 (CH_3_), 27.7 (CH_3_); HRMS: *m/z* (MALDI-TOF) positive ion, calculated for C_25_H_23_KNO_6_S: [M+K]^+^ 504.0883, found: 504.0880.

*3-(([1,1′-biphenyl]-2-yloxy)methyl)-5-phenyl-1-tosyl-1H-indole*  **4ed**: 89 yield%; gray solid; mp: 130–132 °C d; yellow solid; mp: 110–112 °C; R*_f_* = 0.22 *n*-hexane/AcOEt 90:10); IR (neat): 2965, 2372, 1594, 1374, 1054, 891 cm^−1^; ^1^H NMR (400.13 MHz) (CDCl_3_): δ 7.99 (d, *J* = 8.7 Hz, 1H), 7.70 (d, *J* = 8.1 Hz, 2H), 7.59 (d, *J* = 0.9 Hz, 1H), 7.53–7.46 (m, 6H), 7.42–7.38 (m, 2H), 7.34–7.20 (m, 6H), 7.17 (d, *J* = 8.1 Hz, 2H), 7.09–7.04 (m, 2H), 5.18 (s, 2H), 2.31 (s, 3H); ^13^C NMR (100.6 MHz) (CDCl_3_): δ 155.3 (C), 145.1 (C), 141.2 (C), 138.4 (C), 136.9 (C), 135.3 (C), 134.8 (C), 131.9 (C), 131.2 (CH), 130.0 (CH), 129.6 (CH), 128.8 (CH), 128.7 (CH), 128.0 (CH), 127.5 (CH), 127.1 (CH), 127.0 (CH), 126.9 (CH), 125.3 (CH), 124.6 (CH), 121.8 (CH), 119.0 (C), 118.6 (CH), 113.9 (CH), 113.7 (CH), 63.3 (CH_2_), 21.7 (CH_3_); HRMS: *m/z* (MALDI-TOF) positive ion, calculated for C_34_H_27_KNO_3_S: [M+K]^+^ 568.1349, found: 568.1347.

*3-((phenylsulfonyl)methyl)-1-tosyl-1H-indole* **9ab**: 98% yield; yellow solid; mp: 185–187 °C; R*_f_* = 0.20 (*n*-hexane/AcOEt, 87:13); IR (neat): 3000, 2919, 1653, 1454, 1337, 1008 cm^−1^; ^1^H NMR (400.13 MHz) (DMSO-*d*_6_): δ 7.85 (d, *J* = 8.3 Hz, 1H), 7.72–7.66 (m, 5H), 7.55–7.49 (m, 4H), 7.41 (d, *J* = 8.3 Hz, 2H), 7.31 (t, *J* = 7.5 Hz, 1H), 7.19 (t, *J* = 7.5 Hz, 1H), 4.92 (s, 2H), 2.34 (s, 3H); ^13^C NMR (100.6 MHz) (DMSO-*d_6_*): δ 146.3 (C), 138.3 (C), 134.34 (CH), 134.26 (C), 134.2 (C), 130.8 (CH), 130.1 (C), 129.6 (CH), 128.4 (CH), 128.0 (CH), 127.1 (CH), 125.5 (CH), 123.7 (CH), 121.1 (CH), 113.4 (CH), 111.1 (C), 52.0 (CH_2_), 21.5 (CH_3_); HRMS: *m/z* (MALDI-TOF) positive ion, calculated for C_22_H_19_KNO_4_S_2_: [M+K]^+^ 464.0393, found: 464.0389.

*5-methoxy-1-tosyl-3-(tosylmethyl)-1H-indole* **9da**: 84% yield; brown solid; mp: 159–160 °C; R*_f_* = 0.21 (*n*-hexane/AcOEt, 80:20); IR (neat): 2925, 1597, 1167, 802, 535 cm^−1^; ^1^H NMR (400.13 MHz) (CDCl_3_): δ 7.73 (d, *J* = 9.0 Hz, 1H), 7.61 (d, *J* = 8.2 Hz, 2H), 7.32 (d, *J* = 8.1 Hz, 2H), 7.25 (s, 1H), 7.16 (d, *J* = 8.1 Hz, 2H), 7.02 (d, *J* = 8.1 Hz, 2H), 6.80 (dd, *J_1_* = 9.0 Hz, *J_2_* = 2.1 Hz, 1H), 6.56 (d, *J* = 2.1 Hz, 1H), 4.29 (s, 2H), 3.64 (s, 3H), 2.29 (s, 3H), 2.28 (s, 3H); ^13^C NMR (100.6 MHz) (CDCl_3_): δ 156.7 (C), 145.3 (C), 145.0 (C), 134.9.7 (C), 134.7 (C), 130.8 (C), 130.0 (CH), 129.6 (CH), 129.4 (C), 128.7 (CH), 128.0 (CH), 126.9 (CH), 114.7 (CH), 114.6 (CH), 110.3 (C), 101.5 (CH), 55.6 (CH_3_), 53.8 (CH_2_), 21.70 (CH_3_), 21.69 (CH_3_); HRMS: *m/z* (MALDI-TOF) positive ion, calculated for C_24_H_23_KNO_5_S_2_: [M+K]^+^ 508.0655, found: 508.0652.

*5-methoxy-3-((phenylsulfonyl)methyl)-1-tosyl-1H-indole* **9db**: 76% yield; brown solid; mp: 173–174 °C; R*_f_* = 0.23 (*n*-hexane/AcOEt, 78:22); IR (neat): 2925, 1594, 1167, 812, 593 cm^−1^; ^1^H NMR (400.13 MHz) (DMSO-*d*_6_): δ 7.72 (d, *J* = 9.0 Hz, 1H), 7.69–7.65 (m, 3H), 7.63 (d, *J* = 7.3 Hz, 2H), 7.49–7.45 (m, 3H), 7.40 (d, *J* = 8.2 Hz, 2H), 6.97 (d, *J* = 2.4 Hz, 1H), 6.89 (dd, *J_1_* = 9.0 Hz, *J_2_* = 2.4 Hz, 1H), 4.89 (s, 2H), 3.66 (s, 3H), 2.34 (s, 3H); ^13^C NMR (100.6 MHz) (DMSO-*d*_6_): δ 156.4 (C), 146.1 (C), 138.4 (C), 134.30 (CH), 134.28 (C), 131.3 (C), 130.7 (CH), 129.5 (CH), 128.8 (C), 128.7 (CH), 128.5 (CH), 127.0 (CH), 114.5 (CH), 114.3 (CH), 111.4 (C), 103.3 (CH), 55.8 (CH_3_), 52.0 (CH_2_), 21.5 (CH_3_); HRMS: *m/z* (MALDI-TOF) positive ion, calculated for C_23_H_21_KNO_5_S_2_: [M+K]^+^ 494.0498, found: 494.0498.

*5-phenyl-1-tosyl-3-(tosylmethyl)-1H-indole*  **9ea**: 80% yield; white solid; mp: 162–164 °C; R*_f_* = 0.22 (*n*-hexane/AcOEt 90:10); IR (neat): 2966, 2371, 2260, 1595, 1372, 1014 cm^−1^; ^1^H NMR (400.13 MHz) (CDCl_3_): δ 7.96 (d, *J* = 8.3 Hz, 1H), 7.67 (d, *J* = 8.2 Hz, 2H), 7.51 (d, *J* = 7.5 Hz, 2H), 7.47 (s, 1H), 7.42–7.28 (m, 7H), 7.20–7.15 (m, 3H), 7.12–7.06 (m, 2H), 5.15 (s, 2H), 2.32 (s, 3H); ^13^C NMR (100.6 MHz) (CDCl_3_): 155.4 (C), 145.0 (C), 138.5 (C), 135.4 (C), 135.3 (C), 132.0 (C), 131.1 (CH), 130.0 (CH), 129.7 (CH), 129.4 (C), 128.7 (CH), 128.1 (CH), 127.1 (CH), 126.9 (CH), 125.0 (CH), 124.8 (CH), 123.4 (CH), 121.9 (CH), 119.9 (CH), 118.9 (C), 113.9 (CH), 113.8 (CH), 63.5 (CH_2_), 21.7 (CH_3_); HRMS: *m/z* (MALDI-TOF) positive ion, calculated for C_29_H_25_KNO_4_S_2_: [M+K]^+^ 554.0862, found: 554.0859.

*5-phenyl-3-((phenylsulfonyl)methyl)-1-tosyl-1H-indole* **9eb**: 97% yield; yellow solid; mp: 158–160 °C; R*_f_* = 0.19 *n*-hexane/AcOEt 90:10); IR (neat): 2967, 1450, 1301, 1171, 1032, 887 cm^−1^; ^1^H NMR (400.13 MHz) (CDCl_3_): δ 7.98 (d, *J* = 8.7 Hz, 1H), 7.76 (d, *J* = 8.1 Hz, 2H), 7.56 (d, *J* = 7.7 Hz, 2H), 7.53–7.36 (m, 9H), 7.33 (d, *J* = 7.1 Hz, 1H), 7.31–7.23 (m, 3H), 4.46 (s, 2H), 2.37 (s, 3H); ^13^C NMR (100.6 MHz) (CDCl_3_): δ 145.5 (C), 140.9 (C), 137.7 (C), 137.2 (C), 135.0 (C), 134.1 (C), 134.0 (CH), 130.2 (C), 130.1 (CH), 129.0 (CH), 128.8 (CH), 128.7 (CH), 128.0 (CH), 127.4 (CH), 127.3 (CH), 127.1 (CH), 124.8 (CH), 117.9 (CH), 113.9 (CH), 110.3 (C), 53.6 (CH_2_), 21.7 (CH_3_); HRMS: *m/z* (MALDI-TOF) positive ion, calculated for C_28_H_23_KNO_4_S_2_: [M+K]^+^ 540.0706, found: 540.0710

*6-chloro-1-tosyl-3-(tosylmethyl)-1H-indole* **9fa**: 75% yield; white solid; mp: 177–178 °C; R*_f_* = 0.23 (*n*-hexane/AcOEt, 85:15); IR (neat): 2922, 2860, 1591, 1137, 673 cm^−1^; ^1^H NMR (400.13 MHz) (CDCl_3_): δ 7.88 (d, *J* = 1.6 Hz, 1H), 7.65 (d, *J* = 8.4 Hz, 2H), 7.36 (d, *J* = 8.2 Hz, 2H), 7.28 (s, 1H), 7.23–7.17 (m, 3H), 7.06 (d, *J* = 8.4 Hz, 3H), 4.30 (s, 2H), 2.32 (s, 6H); ^13^C NMR (100.6 MHz) (CDCl_3_): δ 145.8 (C), 145.2 (C), 135.1 (C), 134.8 (C), 134.7 (C), 131.3 (C), 130.3 (CH), 129.8 (CH), 128.6 (CH), 128.3 (C), 127.9 (CH), 127.0 (CH), 124.4 (CH), 120.7 (CH), 113.8 (CH), 110.0 (C), 53.6 (CH_2_), 21.79 (CH_3_), 21.77 (CH_3_); HRMS: *m*/z (MALDI-TOF) positive ion, calculated for C_23_H_20_ClKNO_4_S_2_: [M+K]^+^ 512.0159, found: 512.0157.

*6-chloro-3-((phenylsulfonyl)methyl)-1-tosyl-1H-indole* **9fb**: 62% yield; white solid; mp: 210–211 °C; R*_f_* = 0.21 (*n*-hexane/AcOEt, 85:15); IR (neat): 2928, 2794, 1597, 1302, 1173, 668 cm^−1^; ^1^H NMR (400.13 MHz) (DMSO-*d*_6_): δ 7.84 (s, 1H), 7.75 (d, *J* = 8.3 Hz, 2H), 7.69 (t, *J* = 7.1 Hz, 1H), 7.64 (d, *J* = 8.0 Hz, 2H), 7.60–7.54 (m, 2H), 7.50 (t, *J* = 7.5 Hz, 2H), 7.45 (d, *J* = 8.3 Hz, 2H), 7.27 (d, *J* = 8.6 Hz, 1H), 4.36 (s, 2H), 2.30 (s, 3H); ^13^C NMR (100.6 MHz) (DMSO-*d*_6_): δ 145.8 (C), 137.6 (C), 135.1 (C), 134.9 (C), 134.1 (CH), 131.5 (C), 130.3 (CH), 129.1 (CH), 128.6 (CH), 128.2 (C), 127.9 (CH), 127.1 (CH), 124.5 (CH), 120.6 (CH), 113.9 (CH), 109.9 (C), 53.6 (CH_2_), 21.8 (CH_3_); HRMS: *m/z* (MALDI-TOF) positive ion, calculated for C_22_H_18_ClKNO_4_S_2_: [M+K]^+^ 498.0003, found: 497.9998.

*5-nitro-1-tosyl-3-(tosylmethyl)-1H-indole* **9ga**: 92% yield; white solid; mp: 230–231 °C; R*_f_* = 0.23 (*n*-hexane/AcOEt, 70:30); IR (neat): 2912, 1508,1336, 1122, 532 cm^−1^; ^1^H NMR (400.13 MHz) (DMSO- *d_6_*): δ 8.36 (d, *J* = 1.8 Hz, 1H), 8.18 (dd, *J*_1_ = 9.2 Hz, *J*_2_ = 1.8 Hz, 1H), 8.11 (d, *J* = 9.2 Hz, 1H), 7.84–7.82 (m, 3H), 7.50 (d, *J* = 8.2 Hz, 2H), 7.46 (d, *J* = 8.2 Hz, 2H), 7.27 (d, *J* = 8.0 Hz, 2H), 5.00 (s, 2H), 2.37 (s, 3H), 2.33 (s, 3H); 13C NMR (100.6 MHz) (DMSO-*d_6_*): δ 147.0 (C), 145.1 (C), 144.1 (C), 137.2 (C), 135.4 (C), 133.8 (C), 131.2 (CH), 131.1 (CH), 130.1 (C), 130.0 (CH), 128.6 (CH), 127.3 (CH), 120.5 (CH), 117.6 (CH), 114.3 (CH), 112.3 (C), 51.9 (CH_2_), 21.6 (CH_3_), 21.4 (CH_3_). HRMS: *m/z* (MALDI-TOF) positive ion, calculated for C_23_H_20_KN_2_O_6_S_2_: [M+K]^+^ 523.0400, found: 523.0400.

*5-nitro-3-((phenylsulfonyl)methyl)-1-tosyl-1H-indole* **9gb**: 83% yield; white solid; mp: 229–230 °C; R*_f_* = 0.20 (*n*-hexane/AcOEt, 70:30); IR (neat): 3100, 1522, 1345, 1125, 535 cm^−1^; ^1^H NMR (400.13 MHz) (CDCl_3_): δ 8.16 (dd, *J_1_* = 8.3 Hz, *J_2_* = 2.2 Hz, 1H), 8.07–8.00 (m, 2H), 7.77 (d, *J* = 8.3 Hz, 2H), 7.68 (s, 1H), 7.63–7.58 (m, 2H), 7.57–7.50 (m, 1H), 7.39–7.28 (m, 4H), 4.45 (s, 2H), 2.40 (s, 3H); ^13^C NMR (100.6 MHz) (CDCl_3_): δ 146.4 (C), 144.4 (C), 137.7 (C), 137.5 (C), 134.4 (CH), 134.3 (CH), 130.5 (CH), 130.4 (CH), 129.7 (C), 129.3 (CH), 128.6 (CH), 127.2 (CH), 120.4 (CH), 116.0 (CH), 114.0 (CH), 110.7 (C), 53.3 (CH_2_), 21.8 (CH_3_); HRMS: *m/z* (MALDI-TOF) positive ion, calculated for C_22_H_18_KN_2_O_6_S_2_: [M+K]^+^ 509.0243, found: 509.0241.

*1,1′-ditosyl-3,3′-bis(tosylmethyl)-1H,1′H-6,6′-biindole* **13a**: 12% yield; white solid; mp: 228–230 °C; R*_f_* = 0.23 (*n*-hexane/AcOEt, 70:30); IR (neat): 2943, 1600, 1283, 1159, 535 cm^−1^; ^1^H NMR (400.13 MHz) (CDCl_3_): δ 8.04 (s, 1H), 7.72 (d, 2H, *J* = 8.3 Hz), 7.45 (d, 2H, *J* = 8.2 Hz), 7.42–7.33 (m, 2H), 7.32 (s, 1H), 7.25 (d, *J* = 8.2 Hz, 2H), 7.18 (s, 1H), 7.12 (d, *J* = 8.2 Hz, 2H), 4.38 (s, 2H), 3.64 (s, 3H), 2.35 (s, 3H), 2.31 (s, 3H); ^13^C NMR (100.6 MHz) (CDCl_3_): δ 145.7 (C), 145.1 (C), 138.7 (C), 135.4 (C), 134.9 (C), 134.8 (C), 130.3 (CH), 129.8 (CH), 129.1 (C), 128.7 (CH), 127.8 (CH), 127.2 (CH), 123.5 (CH), 120.2 (CH), 112.6 (CH), 110.0 (C), 53.8 (CH_2_), 21.82 (CH_3_), 21.79 (CH_3_); HRMS: *m/z* (MALDI-TOF) positive ion, calculated for C_46_H_40_KN_2_O_8_S_4_: [M+K]^+^ 915.1305, found: 915.1306.

*3,3′-bis((phenylsulfonyl)methyl)-1,1′-ditosyl-1H,1′H-6,6′-biindole* **13b**: 8% yield; white solid; mp: 230–231 °C; R*_f_* = 0.23 (*n*-hexane/AcOEt, 70:30); IR (neat): 2922, 1597, 1294, 1170, 583 cm^−1^; ^1^H NMR (400.13 MHz) (CDCl_3_): δ 8.04 (s, 1H), 7.65–7.49 (m, 3H), 7.41–7.28 (m, 5H), 7.26 (d, 2H, *J* = 8.2 Hz), 4.41 (s, 2H), 2.31 (s, 3H); ^13^C NMR (100.6 MHz) (CDCl3): δ 145.6 (C), 138.6 (C), 137.6 (C), 135.2 (C), 134.8 (C), 134.0 (CH), 130.2 (CH), 129.1 (CH), 128.9 (C), 128.5 (CH), 127.7 (CH), 127.0 (CH), 123.4 (CH), 119.9 (CH), 112.5 (CH), 109.7 (C), 53.7 (CH_2_), 21.7 (CH_3_); HRMS: *m/z* (MALDI-TOF) positive ion, calculated for C_44_H_36_KN_2_O_8_S_4_: [M+K]^+^ 887.0992, found: 887.0991.

## Figures and Tables

**Table 1:** Optimization studies for the reaction of **3** with 4-methoxyphenol **6a** ^a^.

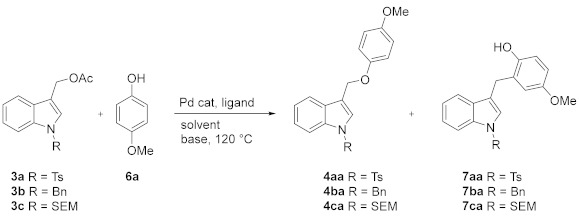								
Entry ^b^	R	Pd Cat	Ligand	Solvent	Base	t (h)	4 (%)	7 (%)
**1**	Ts	/	/	DMSO	K_2_CO_3_	0.5	/ (**4aa**)	/
**2**	Ts	/	/	MeCN	K_2_CO_3_	8	/ (**4aa**)	/
**3** ^c^	Bz	/	/	DMSO	K_2_CO_3_	5	/ (**4ba**)	/
**4** ^d^	SEM	/	/	DMSO	K_2_CO_3_	24	23 (**4ca**)	/
**5**	SEM	/	/	MeCN	K_2_CO_3_	24	32 (**4ca**)	/
**6**	SEM	[Pd(C_3_H_5_)Cl]_2_	XPhos	MeCN/THF 4:1	K_2_CO_3_	2	75 (**4ca**)	/
**7**	SEM	Pd_2_dba_3_	XPhos	MeCN	K_2_CO_3_	24	37 (**4ca**)	/
**8**	SEM	Pd(PPh_3_)_4_	/	MeCN	K_2_CO_3_	24	38 (**4ca**)	/
**9**	SEM	[Pd(C_3_H_5_)Cl]_2_	XPhos	MeCN/THF 4:1	Cs_2_CO_3_	17	52 (**4ca**)	18 (**7ca**)
**10**	SEM	[Pd(C_3_H_5_)Cl]_2_	XPhos	MeCN/THF 4:1	Na_2_CO_3_	23	62 (**4ca**)	6 (**7ca**)
**11**	SEM	[Pd(C_3_H_5_)Cl]_2_	XPhos	MeCN/THF 4:1	K_3_PO_4_	23	49 (**4ca**)	13 (**7ca**)
**12** ^e^	SEM	[Pd(C_3_H_5_)Cl]_2_	XPhos	MeCN/THF 4:1	*^t^*BuONa	0.5	9 (**4ca**)	/
**13** ^f^	SEM	[Pd(C_3_H_5_)Cl]_2_	XPhos	MeCN/THF 4:1	K_2_CO_3_	2	65 (**4ca**)	/
**14**	SEM	[Pd(C_3_H_5_)Cl]_2_	RuPhos	MeCN/THF 4:1	K_2_CO_3_	25	45 (**4ca**)	21 (**7ca**)
**15**	SEM	[Pd(C_3_H_5_)Cl]_2_	SPhos	MeCN/THF 4:1	K_2_CO_3_	24	31 (**4ca**)	20 (**7ca**)
**16**	SEM	[Pd(C_3_H_5_)Cl]_2_	*^t^*BuXPhos	MeCN/THF 4:1	K_2_CO_3_	4.5	64 (**4ca**)	2 (**7ca**)
**17**	SEM	[Pd(C_3_H_5_)Cl]_2_	JohnPhos	MeCN/THF 4:1	K_2_CO_3_	7.5	39 (**4ca**)	24 (**7ca**)
**18**	SEM	[Pd(C_3_H_5_)Cl]_2_	DavePhos	MeCN/THF 4:1	K_2_CO_3_	23	47 (**4ca**)	13 (**7ca**)
**19**	SEM	[Pd(C_3_H_5_)Cl]_2_	XPhos	DMF/THF 4:1	K_2_CO_3_	24	30 (**4ca**)	18 (**7ca**)
**20** ^g^	SEM	[Pd(C_3_H_5_)Cl]_2_	XPhos	DMSO/THF 4:1	K_2_CO_3_	24	20 (**4ca**)	3 (**7ca**)
**21**	Ts	[Pd(C_3_H_5_)Cl]_2_	XPhos	MeCN/THF 4:1	K_2_CO_3_	2	97 (**4aa**)	/
**22**	Bz	[Pd(C_3_H_5_)Cl]_2_	XPhos	MeCN/THF 4:1	K_2_CO_3_	3	57 (**4ba**)	13 (**7ba**)

^a^ Unless otherwise stated, reactions were carried out on a 0.291 mmol scale under an argon atmosphere using 0.05 equiv. of Pd, 0.05 equiv. of ligand, 2 equiv. of **6a**, and 2 equiv. of base in 2.5 mL of anhydrous solvent. ^b^ Yields are given for isolated products. ^c^ 3-formyl-*N*-benzylindole was isolated in 35% yield. ^d^ 3-formyl-*N*-SEMindole was isolated in 53% yield. ^e^ *N*-SEM-3-carbinol was isolated in 55%. ^f^ Reaction was carried out with potassium salt of **6a**. ^g^ 3-formyl-*N*-SEMindole was isolated in 55% yield.

**Table 2:** *N*-Ts-1-methyl-3-(aryloxymethyl)-1*H*-indole **4** from *N*-substituted-indol-3-ylmethyl acetates **3** and phenols **6 ^a^**.

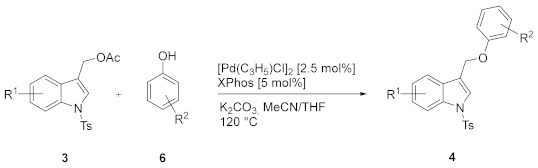						
Entry ^b^	R^1^	3	R^2^	6	t (h)	4 (%)
**1**	H	**3a**	4-*t*-Bu	**6b**	3	83 (**4ab**)
**2**	H	**3a**	4-F	**6c**	3	81 (**4ac**)
**3**	H	**3a**	2-Ph	**6d**	1.5	80 (**4ad**)
**4**	H	**3a**	3-CO_2_Me	**6e**	3	76 (**4ae**)
**5**	H	**3a**	4-CN	**6f**	3	46 (**4af**)
**6** ^c^	H	**3a**	4-CN	**6f**	1	75 (**4af**)
**7**	H	**3a**	4-NO_2_	**6g**	24	/
**8** ^c^	H	**3a**	4-NO_2_	**6g**	24	/
**9**	5-OMe	**3d**	4-OMe	**6a**	1	90 (**4da**)
**10**	5-OMe	**3d**	4-*t*-Bu	**6b**	1.5	94 (**4db**)
**11**	5-OMe	**3d**	2-Ph	**6c**	1.5	92 (**4dd**)
**12**	5-OMe	**3d**	3-CO_2_Me	**6e**	1.5	90 (**4de**)
**13**	5-Ph	**3e**	2-Ph	**6d**	1	89 (**4ed**)
**14**	6-Cl	**3f**	4-*t*-Bu	**6b**	24	/

^a^ Unless otherwise stated, reactions were carried out on a 0.291 mmol scale under an argon atmosphere at 120 °C using 2.0 equiv. of **6**, 0.025 equiv. of [Pd(C_3_H_5_Cl)]_2_ and 0.05 equiv. of XPhos, and 2 equiv. of K_2_CO_3_ in 2.5 mL of MeCN/THF mixture (4:1). ^b^ Yields are given for isolated products. ^c^ Reaction was carried out with potassium salt of **6f** and **6g**.

**Table 3:** *N*-Ts-1-methyl-3-((arylsulfonyl)methyl)-1*H*-indole **9** from *N*-substituted-indol-3-ylmethyl acetates **3** and sodium arylsulfinates **8 ^a^**.

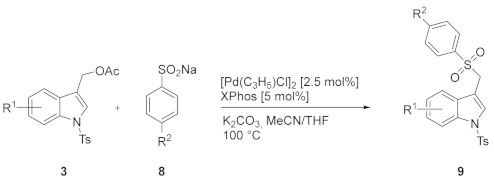						
Entry ^b^	R^1^	3	R^2^	8	t (h)	9 (%)
**1** ^c,d^	H	**3a**	Me	**8a**	16	/
**2**	H	**3a**	Me	**8a**	1.5	98 (**9aa**)
**3**	H	**3a**	H	**8b**	1	98 (**9ab**)
**4**	5-OMe	**3d**	Me	**8a**	1	84 (**9da**)
**5**	5-OMe	**3d**	H	**8b**	1	76 (**9db**)
**6**	5-Ph	**3e**	Me	**8a**	1	87 (**9ea**)
**7**	5-Ph	**3e**	H	**8b**	30	75 (**9eb**)
**8** ^e,f^	6-Cl	**3f**	Me	**8a**	26	75 (**9fa**)
**9** ^g^	6-Cl	**3f**	H	**8b**	7	62 (**9fb**)
**10**	5-NO_2_	**3g**	H	**8a**	1	92 (**9ga**)
**11**	5-NO_2_	**3g**	H	**8b**	6	83 (**9gb**)

^a^ Unless otherwise stated, reactions were carried out on a 0.291 mmol scale under an argon atmosphere at 100 °C using 2.0 equiv. of **8**, 0.025 equiv. of [Pd(C_3_H_5_Cl)]_2_ and 0.05 equiv. of XPhos, and 2 equiv. of K_2_CO_3_ in 2.5 mL of MeCN/THF mixture (4:1). ^b^ Yields are given for isolated products. ^c^ The reaction was carried out under metal-free conditions. ^d^ **3a** was isolated in 95% yield. ^e^ **3f** was isolated in 10% yield. ^f^ **13a** was isolated in 4% yield. ^g^ **13b** was isolated in 6% yield.

## Data Availability

Data contained within the article or Supplementary Materials.

## Supplementary Materials

The following supporting information can be downloaded at: https://www.mdpi.com/article/10.3390/molecules29143434/s1. Reference [51] is cited in Supplementary Materials.
