# Supplementary material for: Palladium-Catalyzed Tsuji–Trost-Type Reaction of 3-Indolylmethylacetates with O, and S Soft Nucleophiles
Source: Molecules. 2024 Jul 22;29(14):3434. doi: 10.3390/molecules29143434 (PMC11280231; doi:10.3390/molecules29143434)

"Palladium-Catalyzed Tsuji–Trost-Type Reaction of 3-Indolylmethylacetates with O, and S Soft Nucleophiles"

Antonia Iazzetti,* Antonio Arcadi,[3] Marco Chiarini[4], Giancarlo Fabrizi,* Antonella Goggiamani, Federico Marrone, Andrea Serraiocco, Roberta Zoppoli

Contents

1. GENERAL INFORMATION S1

1.1. Reagents and methods S1

2. SYNTHETIC PROCEDURES FOR STARTING MATERIALS S2

2.1 Typical procedure for the preparation of the 5-phenyl(1-tosyl-1*H*-indol-3-yl)methyl acetate **3e**: S2

2.2.1 Typical procedure for the preparation of the 5-phenyl-1H-indole-3-carbaldehyde: S2

2.2.2 Typical procedure for the preparation of ethyl 5-phenyl-1-tosyl-1H-indole-3-carbaldehyde: S3

4. CHARACTERIZATION DATA OF STARTING MATERIAL S4

4.1. Characterization data of substituted 1-tosyl-1*H*-indole-3-carbaldehydes: S4

4.2. Characterization data of substituted (1-tosyl-1*H*-indol-3-yl)methanols: S6

4.3. Characterization data of (1-tosyl-1*H*-indol-3-yl)methyl acetates **3a**-**3d** and **3f**-**3g**: S8

5. REFERENCES S10

6. COPIES OF NMR SPECTRA S11

1. GENERAL INFORMATION

1.1. Reagents and methods

All of the commercially available reagents, catalysts, bases and solvents were used as purchased, without further purification. Starting materials and reaction products were purified by flash chromatography using SiO_2 as stationary phase, eluting with *n*-hexane/ethyl acetate. ^{1}H NMR (400.13 MHz), ^{13}C NMR (100.6 MHz), and ^{19}F spectra (376.5 MHz) were recorded with a Bruker Avance 400 spectrometer. Splitting patterns are designed as s (singlet), d (doublet), t (triplet), q (quartet), m (multiplet), or bs (broad singlet). IR spectra were recorded with a Jasco FT/IR-430 spectrometer. Mass spectra were determined with a Shimadzu QP2010 Gas Chromatograph Mass spectrometer (EI ion source) or with a Shimadzu QP2010-Plus Gas Chromatograph Mass spectrometer (CI ion source/ NCI ionsource, DI). HRMS were recorded with an Orbitrap Exactive Mass spectrometer with ESI source. Melting points were determined with a Büchi B-545 apparatus and are uncorrected.

2. SYNTHETIC PROCEDURES FOR STARTING MATERIALS

Starting materials **3a-3d** and **3f-3g** were prepared according to literature procedures[1,2,3] through the four-step sequence of reactions depicted in the following scheme.

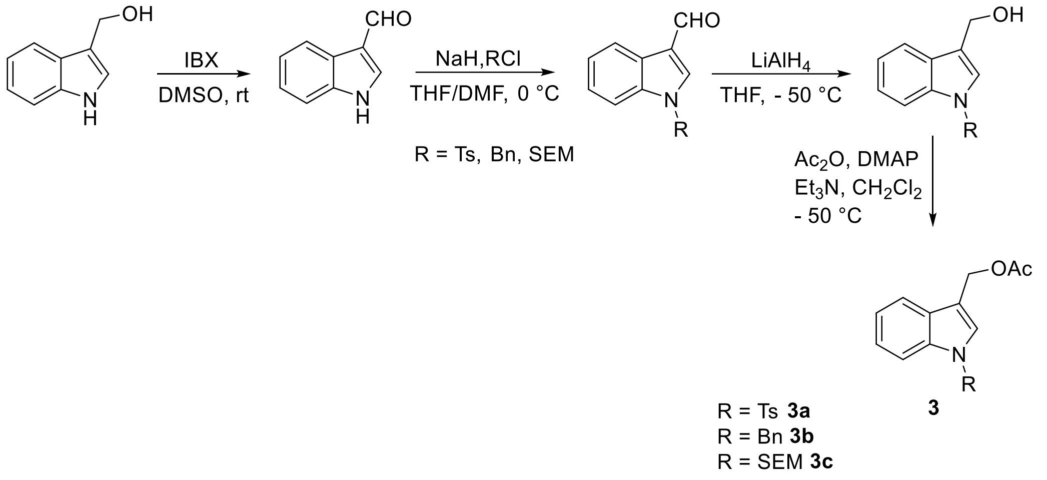

2.1 Typical procedure for the preparation of the 5-phenyl(1-tosyl-1*H*-indol-3-yl)methyl acetate 3e:

Starting materials **3e** was prepared through the four-step sequence of reactions depicted in the following scheme.

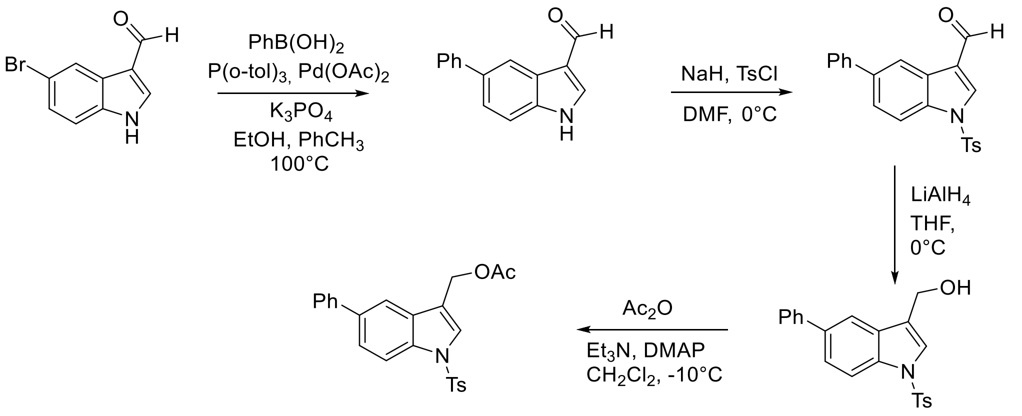

2.2.1 Typical procedure for the preparation of the 5-phenyl-1H-indole-3-carbaldehyde:

In a 100 ml two-necked round bottom flask equipped with a stirring bar P(o-tol)$_3$ (0.220 g, 0.725 mmol, 0.10 equiv.) and Pd(OAc)$_2$ (0.081 g, 0.363 mmol, 0.05 equiv.) were dissolved in ethanol (10 ml) and toluene (5 ml) under inert atmosphere. Then, 5-bromo-1*H*-indole-3-carbaldehyde (1.62 g, 7.250 mmol, 1.00 equiv.), phenylboronic acid (1.75 g, 14.50 mmol, 2.00 equiv.) and K_3PO_4 (3.07 g, 14.50 mmol, 2.00 equiv) were added to the mixture. The reaction was allowed to stir until disappearance of the starting material (monitored by HPLC), then toluene and ethanol were removed under reduced pressure. The residue was diluted with Et_2O, washed with a $KHSO_4$ solution (10% w/w) and brine (2x). The organic layer was dried over Na_2SO_4, filtered and concentrated under reduced pressure, then the crude was purified by flash chromatography (silica gel, *n*-hexane/AcOEt 85/15 v/v, R_f = 0.22) to afford 1.55 g of 5-phenyl-1*H*-indole-3-carbaldehyde in 95% yield.

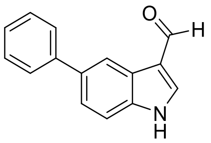

5-phenyl-1H-indole-3-carbaldehyde: known compound; 95% yield; orange solid; mp: 255-257 °C; lit.[4] mp: 257-258 °C; R_f = 0.21 (*n*-hexane/AcOEt, 75:25); IR (neat): 3330, 2360, 1732, 1582, 1082, 812 cm^{-1}; ^1H NMR (400.13 MHz) (DMSO-d_6): δ 11.7 (s, 1H), 9.43 (s, 1H), 7.81 - 7.76 (m, 2H), 7.13 - 7.11 (m, 2H), 7.08 - 7.00 (m, 2H), 6.95 - 6.91 (m, 2H) 6.82 - 6.78 (m, 1H); ^{13}C NMR (100.6 MHz) (DMSO-d_6): δ 185.1 (CH), 141.2 (C), 139.1 (CH), 136.6 (C), 134.8 (C), 128.9 (CH), 126.9 (CH), 126.8 (CH), 124.8 (C), 122.8 (CH), 118.8 (CH), 118.4 (C), 112.9 (CH); HRMS: *m/z* $[M + H]^+$ calcd for $C_{15}H_{11}NO$: 221.0800; found: 221.0801.

2.2.2 Typical procedure for the preparation of ethyl 5-phenyl-1-tosyl-1H-indole-3-carbaldehyde:

To a stirred solution of NaH (60% dispersion in mineral oil, 0.33 g, 8.27 mmol, 1.2 equiv.) in dry THF (5 mL) *5-phenyl-1H-indole-3-carbaldehyde* (1.52 g, 6.89 mmol, 1.0 equiv.), dissolved in anhydrous DMSO (7.5 mL), was added dropwise at 0°C under argon. The reaction mixture was warmed to room temperature, stirred for 5 minutes and, after cooling to 0°C, a solution of TsCl (1.45 g, 7.58 mmol, 1.1 equiv.) in anhydrous THF (25 mL) was added dropwise. The reaction mixture was allowed to warm to room temperature again and stirred for 5h. After the consumption of the substrate (TLC, *n*-hexane/AcOEt, 80:20), the reaction was quenched with the addition of H_2O, diluted with Et_2O, washed with a solution of $KHSO_4$ (10% w/w), a saturated solution of $NaHCO_3$, and brine. The organic layer was dried over Na_2SO_4, filtered, and concentrated under vacuum. The residue was purified by chromatography on SiO_2 (25-40 µm), eluting with 85/15 (v/v) *n*-hexane/AcOEt mixture (R_f = 0.23) to obtain 1.87 g (73% yield) of 1-tosyl-1*H*-indole-3-carbaldehyde.

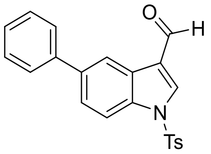

5-phenyl-1-tosyl-1H-indole-3-carbaldehyde: 73% yield; white solid; mp: 204-206 °C; R_f = 0.23 (*n*-hexane/AcOEt 85:15); IR (neat): 3123, 2830, 1676, 1495, 1230, 972 cm^{-1}; ^1H NMR (400 MHz) ($CDCl_3$): δ 10.12 (s, 1H), 8.48 (d, *J* = 1.4 Hz, 1H), 8.25 (s, 1H), 8.01 (d, *J* = 8.7 Hz, 1H), 7.88 (d, *J* = 8.3 Hz, 2H), 7.67 - 7.60 (m, 3H), 7.46 - 7.42 (m, 2H), 7.35 (tt, J_1 = 7.3 Hz, J_2 = 1.6 Hz, 1H), 7.31 (d, *J* = 8.3 Hz, 2H), 2.38 (s, 3H); ^{13}C NMR (100.6 MHz) ($CDCl_3$): δ 185.6 (CH), 146.4 (C),140.8 (C), 138.8 (C), 136.8 (CH), 134.7 (C), 134.5 (C), 130.5 (CH), 128.9 (CH), 127.6 (CH), 127.5 (CH), 127.4 (CH), 126.0 (CH), 122.6 (C), 121.1 (CH), 113.6 (CH_3); HRMS: *m/z* (MALDI-TOF) positive ion, calculated for $C_{22}H_{17}NNaO_3S$: $[M+Na]^+$ 398.0827, Found: 398.0829.

2.2.3 Typical procedure for the preparation of the 5-phenyl(1-tosyl-1H-indol-3-yl)methanol:

A flame-dried three-necked round bottom flask, equipped with a magnetic stirring bar, was charged with 1-tosyl-1*H*-indole-3-carbaldehyde (1.87 g, 4.98 mmol, 1.0 equiv.) dissolved in anhydrous THF (20 mL) under argon. The resulting solution was cooled to -50 °C before adding dropwise a solution of $LiAlH_4$ in THF (2 M, 1.50 mL, 2.99 mmol, 0.6 equiv.) and was stirred for 15 minutes. After consuming the starting material (TLC, *n*-hexane/AcOEt, 70/30), the reaction was quenched by slow addition of an 80% aqueous MeOH solution at 0 °C. The resulting mixture was extracted with Et_2O, washed with a solution of $NaHSO_4$ (10% w/w), brine, and the organic layer was dried over Na_2SO_4, filtered, and concentrated under reduced pressure at 30°C. The resulting crude product was used in the next step without further purification (1.63 g, 87% yield)

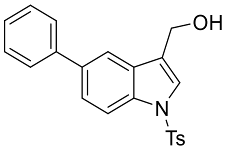

5-phenyl(1-tosyl-1H-indol-3-yl)methanol: 87% yield; yellow solid; mp: 93-95 °C; R_f = 0.20 (*n*-hexane/AcOEt 70:30); IR (neat): 2981, 1676, 1453, 1230, 1032, 816 cm^{-1}; ^1H NMR (400.13 MHz) ($CDCl_3$): δ 8.04 (d, *J* = 8.6 Hz ,1H), 7.83 - 7.77 (m, 3H), 8.62 - 7.54 (m, 4H), 7.45 - 7.41 (m, 2H), 7.35 - 7.32 (m, 1H), 7.23 (d, *J* = 8.1 Hz, 2H), 4.85 (s, 3H), 2.34 (s, 3H), 1.68 (s, 1H); ^{13}C NMR (100.6 MHz) ($CDCl_3$): δ 145.2 (C), 141.3 (C), 137.0 (C), 135.3 (C), 134.9 (C), 130.1 (CH), 128.9 (CH) overlapping, 127.5 (CH), 127.2 (CH), 127.0 (CH), 124.7 (CH), 124.5 (CH), 122.6 (C), 118.5 (CH), 114.5 (CH), 57.3 (CH_2), 21.7 (CH_3); HRMS: *m/z* (MALDI-TOF) positive ion, calculated for $C_{22}H_{19}KNO_3S$: $[M+K]^+$ 416.0723, Found: 416.0718.

2.2.4 Typical procedure for the preparation of the 5 phenyl(1-tosyl-1H-indol-3-yl)methyl acetate 3e:

To a stirred solution of 5-phenyl(1-tosyl-1*H*-indol-3-yl)methanol (1.63 g, 4.33 mmol, 1.0 equiv.) in CH_2Cl_2 (10 mL) DMAP (0.052 g, 0.43 mmol, 0.1 equiv.), triethylamine (0.933 mL, 6.50 mmol, 1.5 equiv.) and acetic anhydride (0.61 mL, 6.50 mmol, 1.5 equiv.) were added at -50 °C and the resulting reaction mixture was stirred for 15 minutes. After the consumption of substrate (TLC, *n*-hexane/AcOEt, 85:15), the reaction was diluted with Et_2O, washed with a saturated $NaHCO_3$ solution, and with brine. The combined organic layer was dried over Na_2SO_4, filtered, and concentrated under reduced pressure. The resulting compound **3e** was used in the next step without further purification (1.63 g, 90% yield).

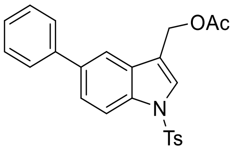

5 phenyl(1-tosyl-1H-indol-3-yl)methyl acetate ***3e***: 90% yield; pale pink solid; mp: 112-114 °C; R_f = 0.22 (*n*-hexane/AcOEt 85:15); IR (neat): 3679, 2980, 1732, 1455, 1214, 1003, 885 cm^{-1}; ^1H NMR (400 MHz) ($CDCl_3$): δ 8.03 (d, *J* = 8.6 Hz, 1H), 7.82 (d, *J* = 8.3 Hz, 2H), 7.75 (d, *J* = 1.2 Hz, 1H), 7.65 (s, 1H), 7.61 - 7.56 (m, 3H), 7.46 - 7.42 (m, 2H), 7.36 - 7.33 (m, 1H), 7.25 (d, *J* = 8.3 Hz, 2H), 5.27 (s, 2H), 2.35 (s, 3H), 2.08 (s, 3H); ^{13}C NMR (100.6 MHz) ($CDCl_3$): δ 145.3 (C), 141.2 (C), 137.1 (C), 135.3 (C), 134.6 (C), 130.1 (CH), 128.9 (CH), 127.5 (CH), 127.3 (CH), 127.1 (CH), 126.4 (CH), 124.8 (CH), 118.2 (CH), 117.6 (C), 114.0 (CH), 57.9 (CH_2), 21.7 (CH_3), 21.1 (CH_3); HRMS: *m/z* (MALDI-TOF) positive ion, calculated for $C_{24}H_{21}KNO_4S$: $[M+K]^+$ 458.0828, Found: 458.0830.

4. CHARACTERIZATION DATA OF STARTING MATERIAL

4.1. Characterization data of substituted 1-tosyl-1*H*-indole-3-carbaldehydes:

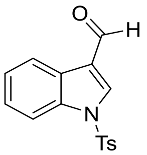

*1-tosyl-1H-indole-3-carbaldehyde***:** known compound; 95% yield; pink solid; mp: 138-140 °C; lit.[1] mp: 138-140 °C; R_f = 0.21 (*n*-hexane/AcOEt, 75:25); IR (neat): 2981, 1663, 1396, 1161, 780 cm^{-1}; ^1H NMR (400.13 MHz)

($CDCl_3$): δ 10.09 (s, 1H), 8.26 - 8.24 (m, 1H), 8.23 (s, 1H), 7.96 - 7.94 (m, 1H), 7.85 (d, *J* = 8.4 Hz, 2H), 7.41 (td, J_1 = 7.3 Hz, J_2 = 1.36 Hz, 1H), 7.36 (td, J_1 = 7.3 Hz, J_2 = 1.4 Hz, 1H), 7.29 (d, *J* = 8.4 Hz, 2H), 2.37 (s, 3H); ^{13}C NMR (100.6 MHz) ($CDCl_3$): δ 185.3 (CH), 146.2 (C), 136.24 (C), 136.21 (CH), 135.2 (C), 134.3 (C), 130.3 (CH), 127.2 (CH), 126.3 (CH), 125.1 (CH), 122.6 (CH), 122.3 (C), 113.3 (CH), 21.7 (CH_3); HRMS: *m/z* $[M + Na]^+$ calcd for $C_{16}H_{13}NO_3SNa$: 322.0508; found: 322.0509.

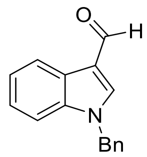

1-benzyl-1H-indole-3-carbaldehyde: known compound; 95% yield; yellow solid; mp: 101-103 °C lit.[1], mp: 101-103 °C; R_f = 0.27 (*n*-hexane/AcOEt ,75:25); IR (neat): 3460, 3278, 2933, 1649, 1353, 1018 cm^{-1}; ^1H NMR (400.13 MHz) ($CDCl_3$): δ 9.94 (s, 1H), 8.27 - 8.25 (m, 1H), 7.64 (s, 1H), 7.30 - 7.22 (m, 6H), 7.11 (d, *J* = 7.3 Hz, 2H), 5.29 (s, 2H); ^{13}C NMR (100.6 MHz) ($CDCl_3$): δ 184.7 (CH), 138.6 (C), 137.5 (C), 135.4 (C), 129.1 (CH), 128.4 (CH), 127.3 (CH), 125.5 (C), 124.2 (CH), 123.1 (CH), 122.2 (CH), 118.5 (C), 110.4 (CH), 50.9 (CH_2).

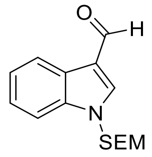

1-((2-(trimethylsilyl)ethoxy)methyl)-1H-indole-3-carbaldehyde: known compound;[1] 97% yield; yellow oil; R_f = 0.22 (*n*-hexane/AcOEt, 85:15); IR (neat): 2953, 2892, 1660, 1081, 744 cm^{-1}; ^1H NMR (400.13 MHz) ($CDCl_3$): δ 10.04 (s, 1H), 8.32 - 8.30 (m, 1H), 7.79 (s, 1H) , 7.54 - 7.52 (m, 1H), 7.39 - 7.32 (m, 2H), 5.53 (s, 3H), 3.51 (t, J = 8.0 Hz, 2H), 0.91 (t, J = 8.0 Hz, 2H), -0.05 (s, 9H); ^{13}C NMR (100.6 MHz) ($CDCl_3$): δ 185.1 (CH), 138.3 (CH), 137.3 (C), 125.7 (C), 124.5 (CH), 123.5 (CH), 122.2 (CH), 119.0 (C), 110.8 (CH), 76.6 (CH_2), 66.7 (CH_2), 17.8 (CH_2), -1.33 (CH_3).

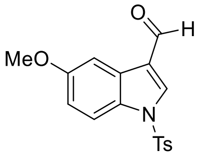

5-methoxy-1-tosyl-1H-indole-3-carbaldehyde: known compond; quantitative yield; white solid; mp: 103-105 °C lit.[3] mp: 128-130 °C; IR (neat): 3662, 2830, 1678, 1251, 1094, 848 cm^{-1}; ^1H NMR (400.13 MHz) (DMSO-d_6): δ 10.1 (s, 1H), 8.82 (s, 1H), 7.98 (d, *J* = 8.4 Hz, 2H), 7.85 (d, *J* = 9.1 Hz, 1H), 7.58 (d, *J* = 2.5 Hz, 1H), 7.44 (d, *J* = 8.2 Hz, 2H), 7.05 (dd, J_1 = 9.1 Hz, J_2 = 2.6 Hz, 1H), 3.78 (s, 3H), 2.34 (s, 3H); ^{13}C NMR (100.6 MHz) (DMSO-d_6): δ 187.2 (CH), 157.7 (C), 146.9 (C), 139.4 (CH), 133.8 (C), 131.0 (CH), 129.3 (C), 127.7 (CH), 127.4 (C), 121.9 (C), 115.6 (CH), 114.6 (CH), 104.3 (CH), 21.5 (CH_3); HRMS: *m/z* $[M + Na]^+$ calcd for $C_{17}H_{15}NO_4SNa$: 352.0614; found: 352.0614.

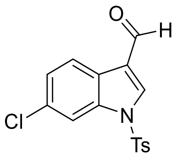

6-chloro-1-tosyl-1H-indole-3-carbaldehyde: known compound; 96% yield; orange solid; mp: 149-151 °C lit.[3]; mp: 149-151 °C; IR (neat): 2914, 1698, 1420, 1223, 1045 cm-1; 1H NMR (400 MHz) ($CDCl_3$): δ 10.0 (s, 1H), 8.20 (s, 1H), 8.16 (d, J = 8.5 Hz, 1H), 7.96 (d, J = 1.7 Hz, 1H), 7.84 (d, J = 8.5 Hz, 2H), 7.34 - 7.32 (m, 3H), 2.40 (s, 3H); 13C NMR (100.6 MHz) ($CDCl_3$): δ 185.1 (CH), 146.6 (C),136.5 (CH), 135.7 (C), 134.2 (C), 132.5 (C), 130.6 (CH), 127.4 (CH), 125.9 (CH), 124.9 (C), 123.6 (CH), 122.1 (C), 113.6 (CH), 21.8 (CH3); HRMS: m/z [M + H]+ calcd for $C_{16}H_{13}ClNO_3S$: 334.0299; found: 334.0295.

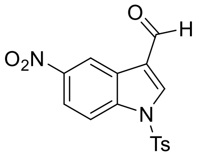

5-nitro-1-tosyl-1H-indole-3-carbaldehyde: known compound; 90% yield; orange solid; mp: 165-167 °C; lit.[2] mp: 165-167 °C; R_f = 0.17 (*n*-hexane/AcOEt, 75:25); IR (neat): 3052, 2830, 1736, 1255, 984 cm^{-1}; ^1H NMR (400.13 MHz) (DMSO-d_6): δ 10.11 (s, 1H), 9.13 (s, 1H), 8.87 (s, 1H), 8.30 (dd, J_1 = 9.3 Hz, J_2 = 2.2 Hz, 1H), 8.21 (d, *J* = 9.3 Hz, 1H), 8.08 (d, *J* = 8.5 Hz, 2H), 7.50 (d, *J* = 8.5 Hz, 2H), 2.36 (s, 3H); ^{13}C NMR (100.6 MHz) (DMSO-d_6): δ 187.2 (CH), 147.7 (C), 145.3 (C), 141.3 (CH), 137.6 (C), 133.3 (C), 131.3 (CH), 128.0 (CH), 121.83 (CH), 121.79 (C), 118.0 (CH), 114.7 (CH), 21.6 (CH_3); HRMS: *m/z* (MALDI-TOF) positive ion, calculated for $C_{16}H_{12}N_2NaO_5S$: $[M+Na]^+$ 367.0365, Found: 367.0367.

4.2. Characterization data of substituted (1-tosyl-1*H*-indol-3-yl)methanols:

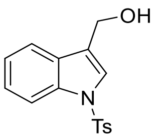

(*1-tosyl-1H-indol-3-yl)methanol***:** known compound; 98% yield; pink solid; mp: 104-105 °C; lit.[1] mp: 104-105 °C; R_f = 0.24 (*n*-hexane/AcOEt, 70:30); ^1H NMR (400.13 MHz) ($CDCl_3$): δ 7.92 (d, *J* = 8.3 Hz, 1H), 7.70 (d, *J* = 8.3 Hz, 2H), 7.53 (d, *J* = 7.8 Hz, 1H), 7.47 (s, 1H), 7.26 (dt, J_1 = 8.2 Hz, J_2 = 1.0 Hz, 1H), 7.20 - 7.13 (m, 3H), 4.75 (s, 2H), 2.26 (s, 3H); ^{13}C NMR (100.6 MHz) ($CDCl_3$): δ 145.1 (C), 135.6 (C), 135.4 (C), 130.0 (CH), 129.6 (C), 127.0 (CH), 125.1 (CH), 123.9 (CH), 123.4 (CH), 122.4 (C), 120.0 (CH), 113.9 (CH), 57.3 (CH_2), 21.7 (CH_3); HRMS: *m/z* $[M + Na]^+$ calcd for $C_{16}H_{15}NO_3SNa$: 324.0665; found: 324.0663

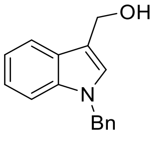

(*1-benzyl-1H-indol-3-yl)methanol* **:** known compound; 97% yield; yellow solid; mp: 87-89 °C; lit.[1] mp: 87-89 °C; R_f = 0.28 (*n*-hexane/AcOEt, 70:30); ^1H NMR (400.13 MHz) ($CDCl_3$): δ 77.78 (d, *J* = 7.6 Hz, 1H), 7.35 - 7.29 (m, 4H), 7.24 (td, J_1 = 7.6 Hz, J_2 = 1.1 Hz, 1H), 7.20 - 7.15 (m, 4H), 5.32 (s, 2H), 4.91 (d, *J* = 4.1 Hz, 2H), 1.55 (bs, 1H); ^{13}C NMR (100.6 MHz) ($CDCl_3$): δ 137.2 (C), 136.9 (C), 128.8 (CH), 127.7 (CH), 127.2 (C), 127.0 (CH),

126.9 (CH), 122.3 (CH), 119.7 (CH), 119.2 (CH), 115.4 (C), 109.9 (CH), 57.3 (CH_2), 50.0 (CH_2); HRMS: *m/z* (MALDI-TOF) positive ion, calculated for $C_{16}H_{15}KNO$: $[M+K]^+$ 276.0791, Found: 276.0790.

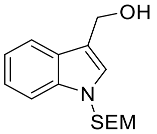

*(1-((2-(trimethylsilyl)ethoxy)methyl)-1H-indol-3-yl)methanol***:** known compound[1]; 98% yield; yellow oil; R_f = 0.23 (*n*-hexane/AcOEt, 85:15); ^{1}H NMR (400.13 MHz) (DMSO-*d$_6$*): δ 7.69 (d, *J* = 8.0 Hz, 1H), 7.58 (d, *J* = 8.0 Hz, 1H), 7.42 (s, 1H), 7.24 (t, *J* = 7.4 Hz, 1H), 7.14 (t, *J* = 7.4 Hz, 1H), 5.57 (s, 2H), 4.92 (t, *J* = 5.1 Hz, 1H), 4.71 (d, *J* = 5.1 Hz, 2H), 3.53 (t, *J* = 8.0 Hz, 2H), 0.89 (t, *J* = 8.0 Hz, 2H), 0.00 (s, 9H); ^{13}C NMR (100.6 MHz) (DMSO-*d$_6$*): δ 137.4 (C), 128.4 (C), 127.7 (CH), 122.4 (CH), 120.14 (CH), 120.11 (CH), 117.3 (C), 111.0 (CH), 75.5 (CH_2), 65.7 (CH_2), 56.1 (CH_2), 18.1 (CH_2), -0.50 (CH_3); HRMS: *m/z* (MALDI-TOF) positive ion, calculated for $C_{15}H_{23}KNO_2Si$: $[M+K]^+$ 316.1135, Found: 316.1140.

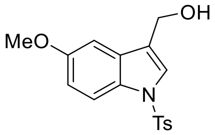

(5-methoxy-1-tosyl-1H-indol-3-yl)methanol: known compound; quantitative yield; brown solid; mp: 122-124 °C; lit.[3] mp: 112-124; IR (neat): 3636, 2364, 1710, 1434, 1078 cm^{-1}; ^{1}H NMR (400 MHz) ($CDCl_3$): δ 7.86 (d, *J* = 9.0 Hz, 1H), 7.73 (d, *J* = 8.3 Hz, 2H), 7.48 (s, 1H), 7.20 (d, *J* = 8.3 Hz, 2H), 7.03 (d, *J* = 2.4 Hz, 1H), 6.94 (dd, J_1 = 9.0 Hz, J_2 = 2.5 Hz, 1H), 4.77 (s, 2H), 3.81 (s, 3H), 2.33 (s, 3H); ^{1}H NMR (100.6 MHz) ($CDCl_3$): δ 156.6 (C), 145.0 (C), 135.3 (C), 130.6 (C), 130.2 (C), 130.0 (CH), 126.9 (CH), 124.7 (CH), 122.4 (C), 114.8 (CH), 114.3 (CH), 102.3 (CH), 57.3 (CH_2), 55.8 (CH_3), 21.7 (CH_3); HRMS: *m/z* $[M + Na]^+$ calcd for $C_{17}H_{17}NO_4SNa$: 354.0770; found: 354.0771.

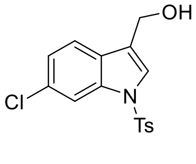

(6-chloro-1-tosyl-1H-indol-3-yl)methanol: known compound; quantitative yield; pink solid; mp: 123-125 °C; lit.[3] mp: 123-125 °C; IR (neat): 3357, 2871, 1725, 1169, 1084, 908 cm^{-1}; ^{1}H NMR (400 MHz) ($CDCl_3$): δ 7.93 (d, *J* = 1.7 Hz, 1H), 7.69 (d, *J* = 8.4 Hz, 2H), 7.45 - 7.43 (m, 2H), 7.19 - 7.13 (m, 3H), 4.71 (s, 2H), 2.28 (s, 3H), 1.65 (s, 1H); ^{13}C NMR (100.6 MHz) ($CDCl_3$): δ 145.5 (C), 135.9 (C), 135.1 (C), 131.2 (C), 130.2 (CH), 128.0 (C), 127.0 (CH), 124.3 (CH), 124.1 (CH), 122.2 (q), 120.9 (CH), 114.0 (CH), 57.1 (CH_2), 21.7 (CH_3); HRMS: *m/z* $[M - H]^-$ calcd for $C_{16}H_{13}Cl\,NO_3S$: 334.0310; found: 334.0304.

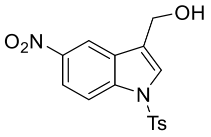

(5-nitro-1-tosyl-1H-indol-3-yl)methanol: known compound; 98% yield; yellow solid; mp: 120-122°C; lit[2] mp: 120-122°C; R_f = 0.17 (*n*-hexane/AcOEt, 65:35); IR (neat): 3014, 1731, 1143, 1064 cm^{-1}; ^1H NMR (400.13 MHz) (DMSO-d_6): δ 8.60 (d, *J* = 2.2 Hz, 1H), 8.23 (dd, J_1 = 9.2 Hz, J_2 = 2.2 Hz, 1H), 8.16 (d, *J* = 9.2 Hz, 1H), 7.95 - 7.92 (m, 3H), 7.42 (d, *J* = 8.1 Hz, 2H), 4.68 (d, *J* = 0.7 Hz, 2H), 2.33 (s, 3H); ^{13}C NMR (100.6 MHz) (DMSO-d_6): δ 146.7 (C), 143.9 (C), 138.0 (C), 134.2 (C), 131.0 (CH), 130.0 (C), 127.3 (CH), 126.9 (CH), 125.0 (C), 120.4 (CH), 117.4 (CH), 114.3 (CH), 55.1 (CH_2), 21.5 (CH_3); HRMS: *m/z* (MALDI-TOF) positive ion, calculated for $C_{16}H_{12}N_2NaO_5S$: $[M+Na]^+$ 367.0365, Found: 367.0367.

4.3. Characterization data of (1-tosyl-1*H*-indol-3-yl)methyl acetates 3a-3d and 3f-3g:

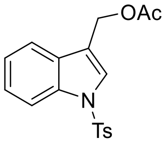

(1-tosyl-1H-indol-3-yl)methyl acetate **3a:** known compound; 98% yield; pale pink solid; mp: 138-140°C; lit.[1] mp: 138-140 °C; R_f = 0.20 (*n*-hexane/AcOEt, 85:15); IR (neat): 2982, 1662, 1538, 1368, 1099, 968 cm^{-1}; ^1H NMR (400.13 MHz) ($CDCl_3$): δ 7.90 (d, *J* = 8.3 Hz, 1H), 7.71 (d, *J* = 8.3 Hz, 2H), 7.55 (s, 1H), 7.49 (d, *J* = 7.7 Hz, 1H), 7.27 (td, J_1 = 8.3 Hz, J_2 = 1 Hz, 1H), 7.21-7.15 (m, 3H); 5.16 (s, 2H), 2.27 (s, 3H), 2.00 (s, 3H); ^{13}C NMR (100.6 MHz) ($CDCl_3$): δ 171.0 (C), 145.2 (C), 135.34 (C), 135.28 (C), 130.1 (CH), 129.6 (C), 127.1 (CH), 125.8 (CH), 125.2 (CH), 123.6 (CH), 119.8 (CH), 117.4 (C), 113.8 (CH), 57.9 (CH_2), 21.7 (CH_3), 21.1(CH_3); HRMS: *m/z* $[M + Na]^+$ calcd for $C_{18}H_{17}NO_4SNa$: 366.0770; found: 366.0768.

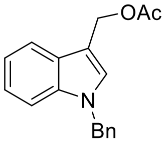

(1-benzyl-1H-indol-3-yl)methyl acetate **3b:** known compound;[1] 98% yield; orange oil; R_f = 0.24 (*n*-hexane/AcOEt, 75:25); IR (neat): 3032, 2933, 1730, 1468, 1226, 943cm^{-1}; ^1H NMR (400.13 MHz) ($CDCl_3$): δ 7.61 (d, *J* = 7.6 Hz, 1H), 7.19 - 7.15 (m, 4H), 7.12 - 7.09 (m, 3H), 7.07 - 7.01 (m, 2H), 5.23 (s, 2H), 5.16 (s, 2H), 1.95 (s, 3H); ^{13}C NMR (100.6 MHz) ($CDCl_3$): δ 171.4 (C), 137.1 (q), 136.8 (C), 129.0 (CH), 128.9 (CH), 127.8 (CH), 127.7 (C), 127.0 (CH), 122.4 (CH), 120.1 (CH), 119.3 (CH), 110.3 (C), 110.0 (CH), 58.6 (CH_2), 50.2 (CH_2), 21.3 (CH_3); HRMS: *m/z* (MALDI-TOF) positive ion, calculated for $C_{18}H_{17}KNO_2$: $[M+K]^+$ 318.0896, Found: 318.0898.

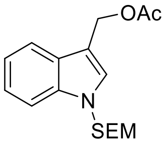

(1-((2-(trimethylsilyl)ethoxy)methyl)-1H-indol-3-yl)methyl acetate **3c:** known compound;[1] yellow oil; 98% yield ; R$_f$ = 0.18 (*n*-hexane/ACOEt, 85:15); IR (neat): 2954, 1735, 1245, 1077, 833 cm^{-1}; ^{1}H NMR (400.13 MHz) (DMSO-*d$_6$)*: δ 7.70 (d, *J* = 8.0 Hz, 1H), 7.65 - 7.63 (m, 2H), 7.29 (t, *J* = 7.4 Hz, 1H), 7.20 (t, *J* = 7.4 Hz, 1H), 5.62 (s, 2H), 5.33 (s, 2H), 3.53 (t, *J* = 8.0 Hz, 2H), 2.09 (s, 3H), 0.90 (t, *J* = 8.0 Hz, 2H), 0.00 (s, 9H); ^{13}C NMR (100.6 MHz) (DMSO-*d$_6$*): δ 171.3 (C), 137.2 (C), 130.6 (CH), 128.4 (C), 122.9 (CH), 120.9 (CH), 119.8 (CH), 111.4 (CH), 111.0 (C), 75.6 (CH_2), 65.8 (CH_2), 58.6 (CH_2), 21.7 (CH_3), 18.0 (CH_2), -0.50 (CH_3); HRMS: *m/z* (MALDI-TOF) positive ion, calculated for $C_{17}H_{25}KNO_3Si$: $[M+K]^+$ 358.1241, Found: 358.1244.

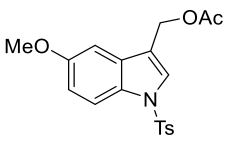

(5-methoxy-1-tosyl-1H-indol-3-yl)methyl acetate **3d**: known compound; quantitative yield; brown solid; mp: 163-165 °C; lit.[3] mp: 163-165 °C; IR (neat): 3553, 2365, 1785, 1529, 1168, 1014 cm^{-1}; ^{1}H NMR (400.16 MHz) (DMSO-*d$_6$*): δ 7.84 - 7.81 (m, 4H), 7.38 (d, *J* = 8.2 Hz, 2H), 7.13 (d, *J* = 2.4 Hz, 1H), 6.98 (dd, J_1 = 9.0 Hz, J_2 = 2.4 Hz, 1H), 5.18 (s, 2H), 3.77 (s, 3H), 2.31 (s, 3H), 2.04 (s, 3H); ^{13}C NMR (100.6 MHz) (DMSO-*d$_6$*): δ 170.9 (C), 156.6 (C), 146.0 (C), 134.5 (C), 130.9 (C), 130.7 (CH), 129.4 (C), 127.4 (CH), 127.1 (CH), 118.2 (C), 114.6 (CH), 114.4 (CH), 103.2 (CH), 57.7 (CH_2), 55.9 (CH_3), 21.5 (CH_3), 21.1 (CH_3); HRMS: *m/z* $[M + Na]^+$ calcd for $C_{19}H_{19}NO_4SNa$: 396.0876; found: 396.0871.

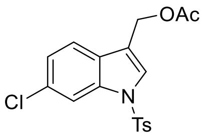

(6-chloro-1-tosyl-1H-indol-3-yl)methyl acetate **3f**: known compound; 95% yield (1.36 mmol scale, 0.51 g); yellow solid; mp: 104-106 °C; lit.[3] mp: 104-106 °C; IR (neat): 2965, 2364, 1877, 1593, 1104, 950 cm^{-1}; ^{1}H NMR (400 MHz) ($CDCl_3$): δ 7.92 (d, *J* = 1.5 Hz, 1H), 7.71 (d, *J* = 8.4 Hz, 2H), 7.52 (s, 1H), 7.41 (d, *J* = 8.4 Hz, 1H), 7.20 (d, *J* = 7.5 Hz, 2H), 7.16 (dd, J_1 = 8.4 Hz, J_2 = 1.8 Hz, 1H) 5.12 (s, 2H), 2.29 (s, 3H), 2.00 (s, 3H); ^{13}C NMR (100.6 MHz) ($CDCl_3$): δ 170.9 (C), 145.6 (C), 135.6 (C), 135.0 (C), 131.3 (C), 130.3 (CH), 128.1 (C), 127.0 (CH), 126.3 (CH), 124.3 (CH), 120.7 (CH), 117.3 (C), 114.0 (CH), 57.6 (CH_2), 21.7 (CH_3), 21.0 (CH_3); HRMS: *m/z* $[M + Na]^+$ calcd for $C_{18}H_{16}ClNO_4SNa$: 400.0381; found: 400.0382.

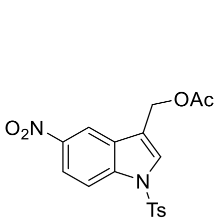

(5-nitro-1-tosyl-1H-indol-3-yl)methyl acetate **3g:** known compound; 73% yield (6.22 mmol scale, 1.76 g); yellow solid; mp: 163-165°C; lit.[2] mp: 163-165°C; R$_f$ = 0.25 (*n*-hexane/AcOEt, 65:35); IR (neat): 2952, 2790, 1710, 1247, 1078 cm^{-1}; ^{1}H NMR (400.13 MHz) (DMSO-*d$_6$*): δ 8.76 (d, *J* = 2.1 Hz, 1H), 8.40 (dd, J_1 = 9.2 Hz, J_2 = 2.1 Hz, 1H), 8.35 (s, 1H), 8.33 (d, *J* = 9.2 Hz, 1H), 8.12 (d, *J* = 8.4 Hz, 2H), 7.60 (d, *J* = 8.4 Hz, 2H), 5.45 (s, 2H), 2.49 (s, 3H), 2.20 (s, 3H); ^{13}C NMR (100.6 MHz) (DMSO-*d$_6$*): δ 170.8 (C), 146.9 (C), 144.2 (C), 137.6 (C), 134.0 (C), 131.0 (CH), 129.8 (CH), 129.6 (C), 127.5 (CH), 120.6 (CH), 118.8 (C), 117.1 (CH), 114.4 (CH), 57.2 (CH_2),

21.5 (CH_3), 21.1 (CH_3). HRMS: *m/z* (MALDI-TOF) positive ion, calculated for $C_{18}H_{17}N_2O_6S$: $[M+H]^+$ 389.0807, Found: 389.0810.

5. REFERENCES

[1] Arcadi, A.; Calcaterra, A.; Chiarini, M.; Fabrizi, G.; Fochetti, A.; Goggiamani, A.; Iazzetti, A.; Marrone, F.; Marsicano, V.; Serraiocco, A., Synthesis of Indole/Benzofuran-Containing Diarylmethanes through Palladium-Catalyzed Reaction of Indolylmethyl or Benzofuranylmethyl Acetates with Boronic Acids. *Synthesis* **2021,** *54* (03), 741-753. DOI: 10.1055/s-0041-1737275.

[2] Arcadi, A.; Aschi, M.; Chiarini, M.; Fabrizi, G.; Fochetti, A.; Goggiamani, A.; Iavarone, F.; Iazzetti, A.; Serraiocco, A.; Zoppoli, R. *ACS Omega* **2024** 9 (26), 28450-28462. DOI: 10.1021/acsomega.4c02409.

[3] Arcadi, A.; Berden, G.; Ciogli, A.; Corinti, D.; Crestoni, M. E.; De Angelis, M.; Fabrizi, G.; Goggiamani, A.; Iazzetti, A.; Marrone, F.; Marsicano, V.; Oomens, J.; Serraiocco, A. Reactivity of Indolylmethylacetates with N, O, and S Soft Nucleophiles: Evidence of 2-Alkylideneindolenines and 3-Alkylideneindoleninium Generation by ESI-MS and IRMPD Spectroscopy. *European Journal of Organic Chemistry* **2022,** *2022* (43), e202201166. https://doi.org/10.1002/ejoc.202201166

[4] Moseley, J. D.; Murray, P. M.; Turp, E. R.; Tyler, S. N. G.; Burn, R. T., A mild robust generic protocol for the Suzuki reaction using an air stable catalyst. *Tetrahedron* **2012,** *68* (30), 6010-6017. https://doi.org/10.1016/j.tet.2012.05.030

6. COPIES OF NMR SPECTRA

1-tosyl-1H-indole-3-carbaldehyde

^{1}H NMR-spectrum (400.13 MHz) ($CDCl_3$)

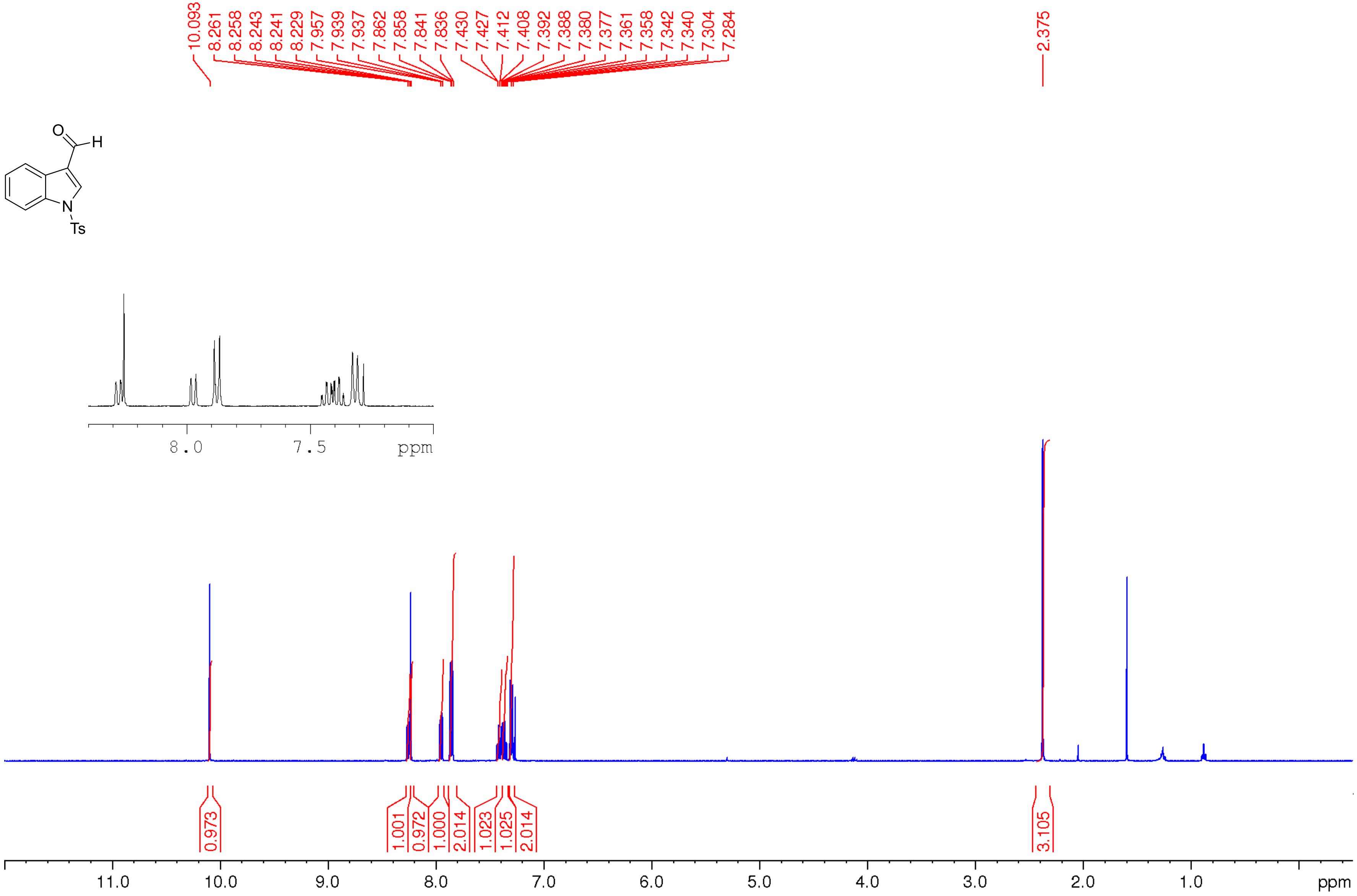

1-tosyl-1H-indole-3-carbaldehyde

^{13}C NMR-spectrum (100.6 MHz) ($CDCl_3$)

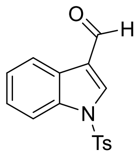

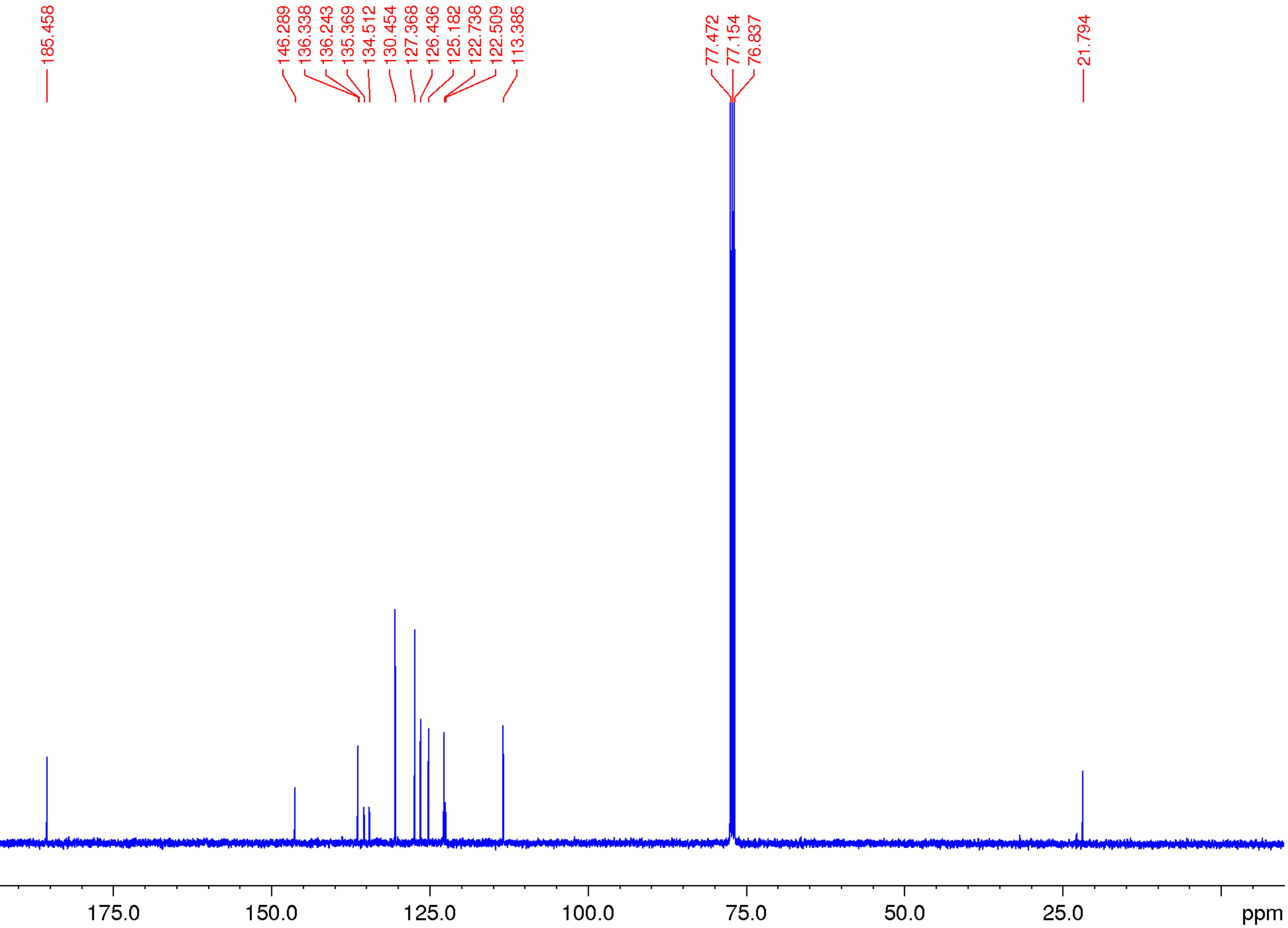

1-tosyl-1H-indole-3-carbaldehyde

DEPT 135 NMR-spectrum ($CDCl_3$)

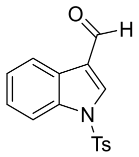

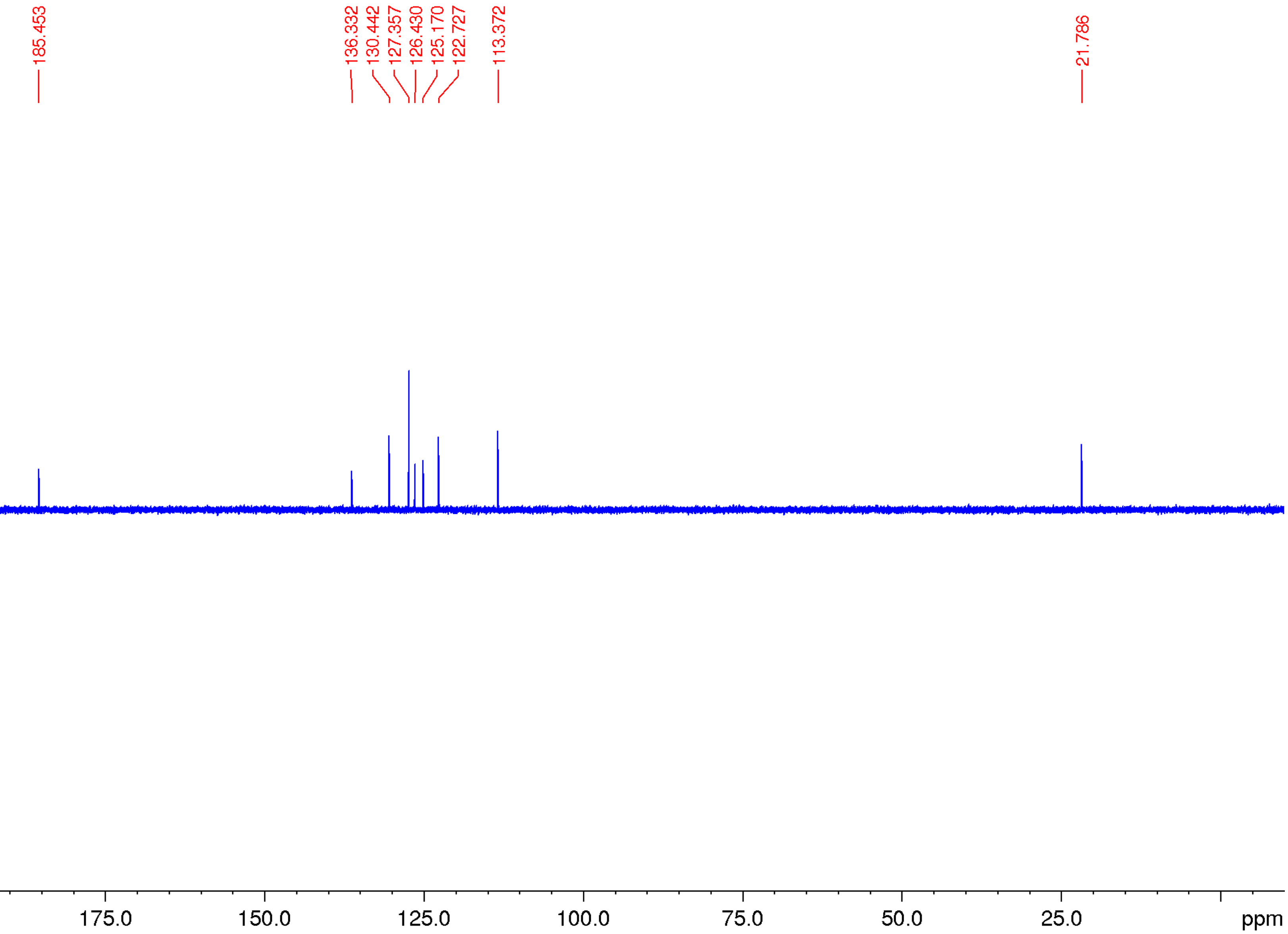

1-tosyl-1H-indole-3-carbaldehyde

^{1}H NMR-spectrum (400.13 MHz) ($CDCl_3$)

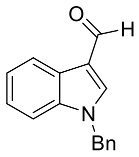

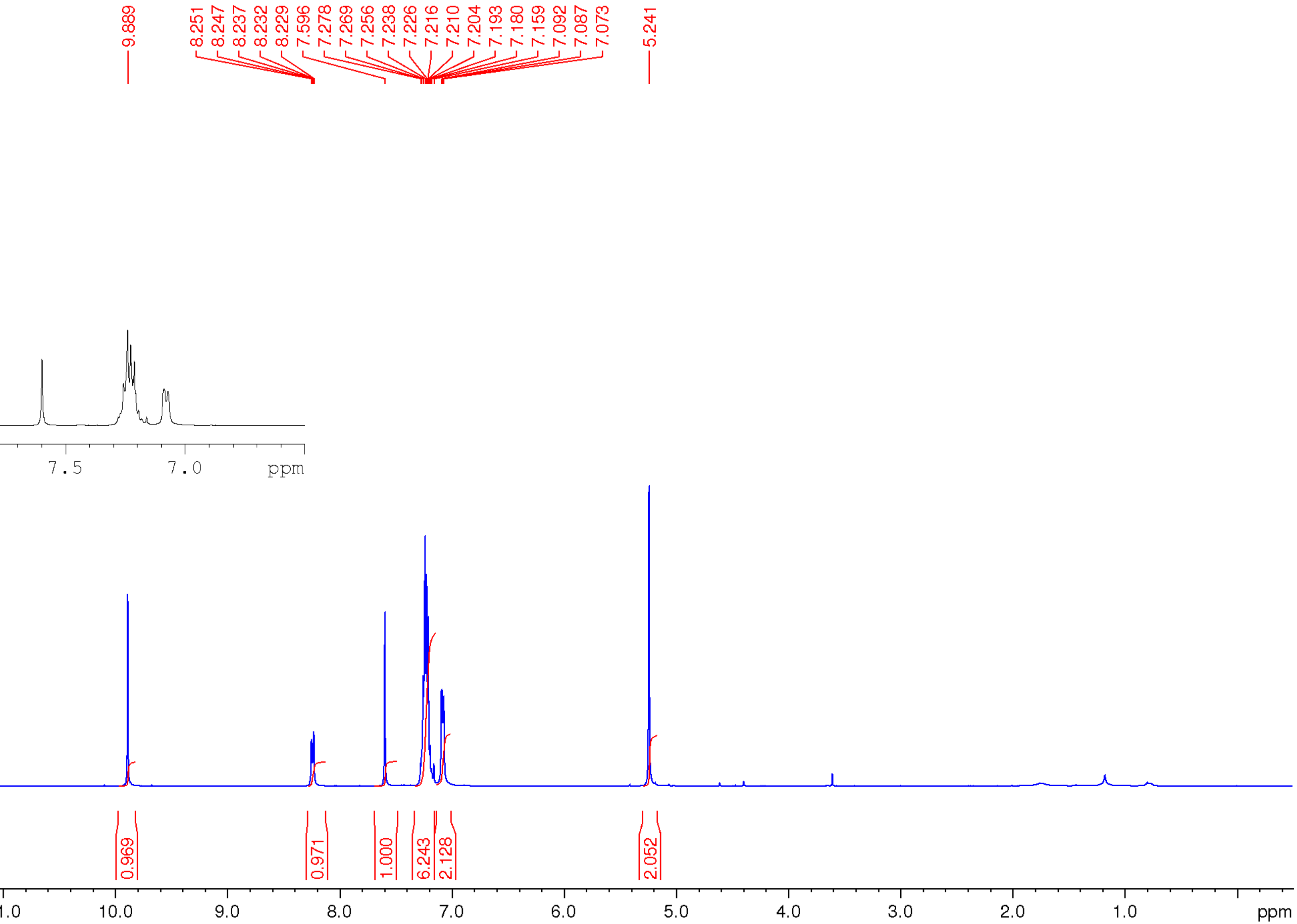

1-tosyl-1H-indole-3-carbaldehyde

^{13}C NMR-spectrum (100.6 MHz) ($CDCl_3$)

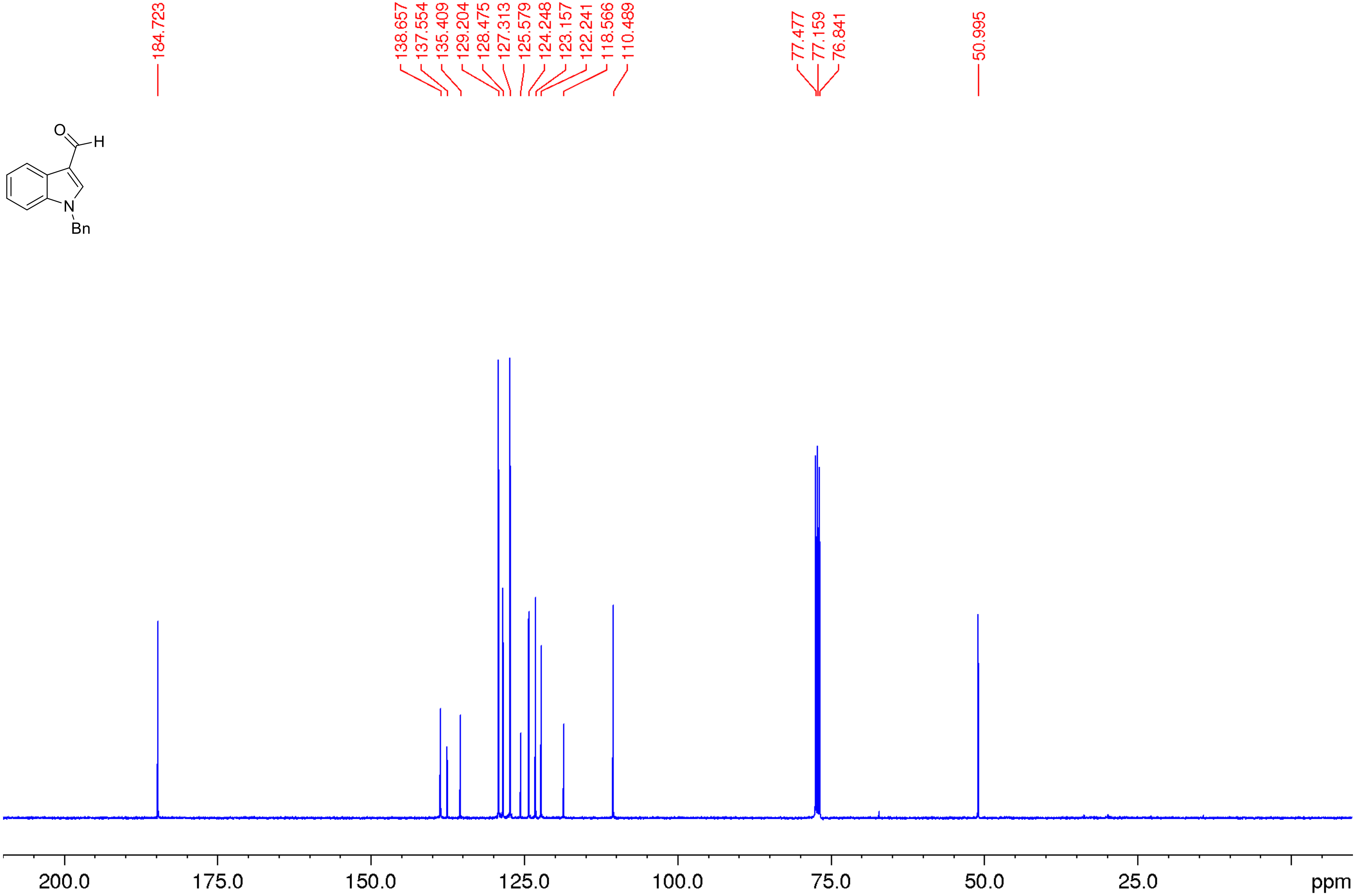

1-tosyl-1H-indole-3-carbaldehyde

DEPT 135 NMR-spectrum ($CDCl_3$)

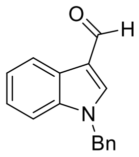

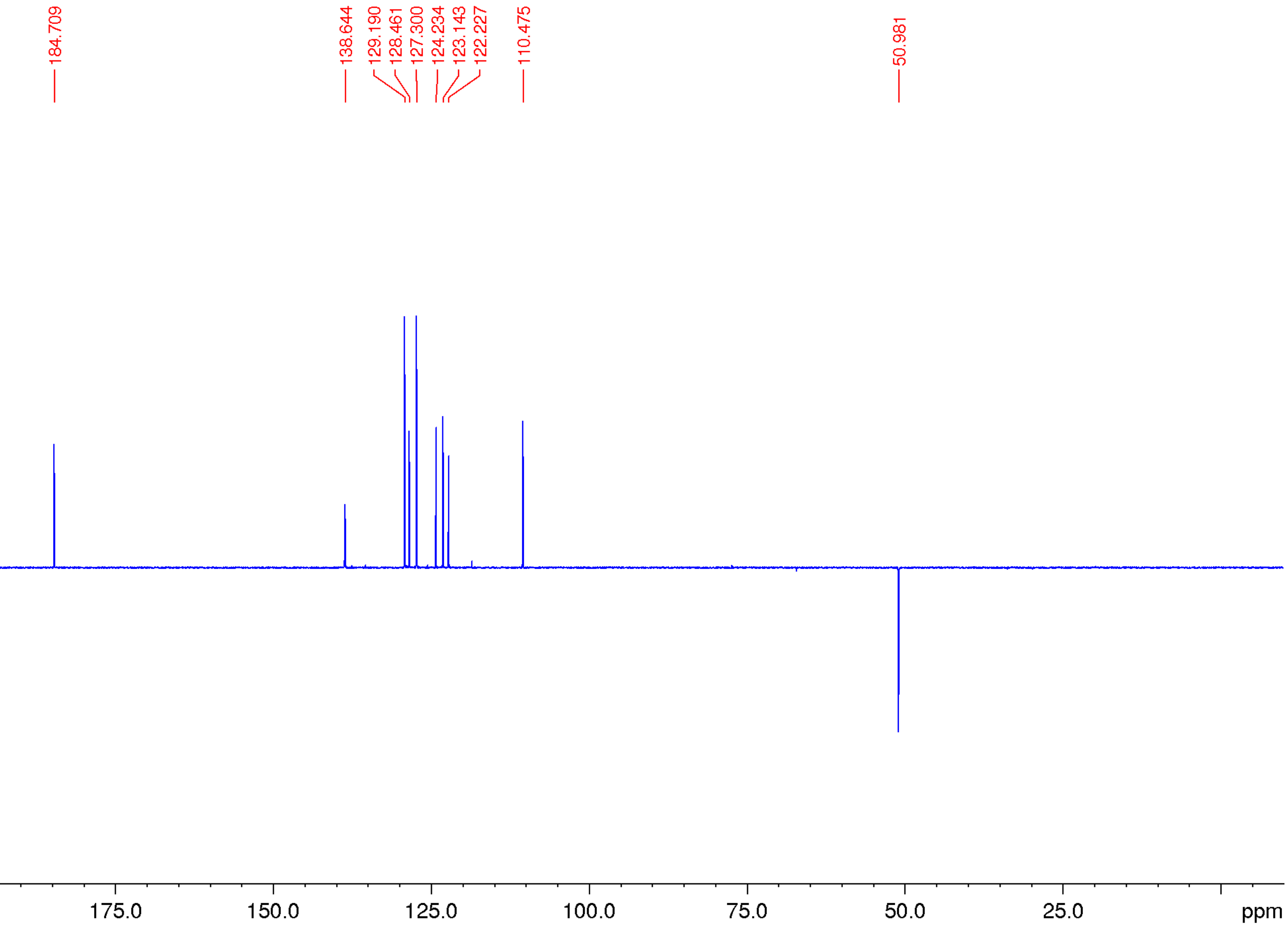

1-tosyl-1H-indole-3-carbaldehyde

^{1}H NMR-spectrum (400.13 MHz) ($CDCl_3$)

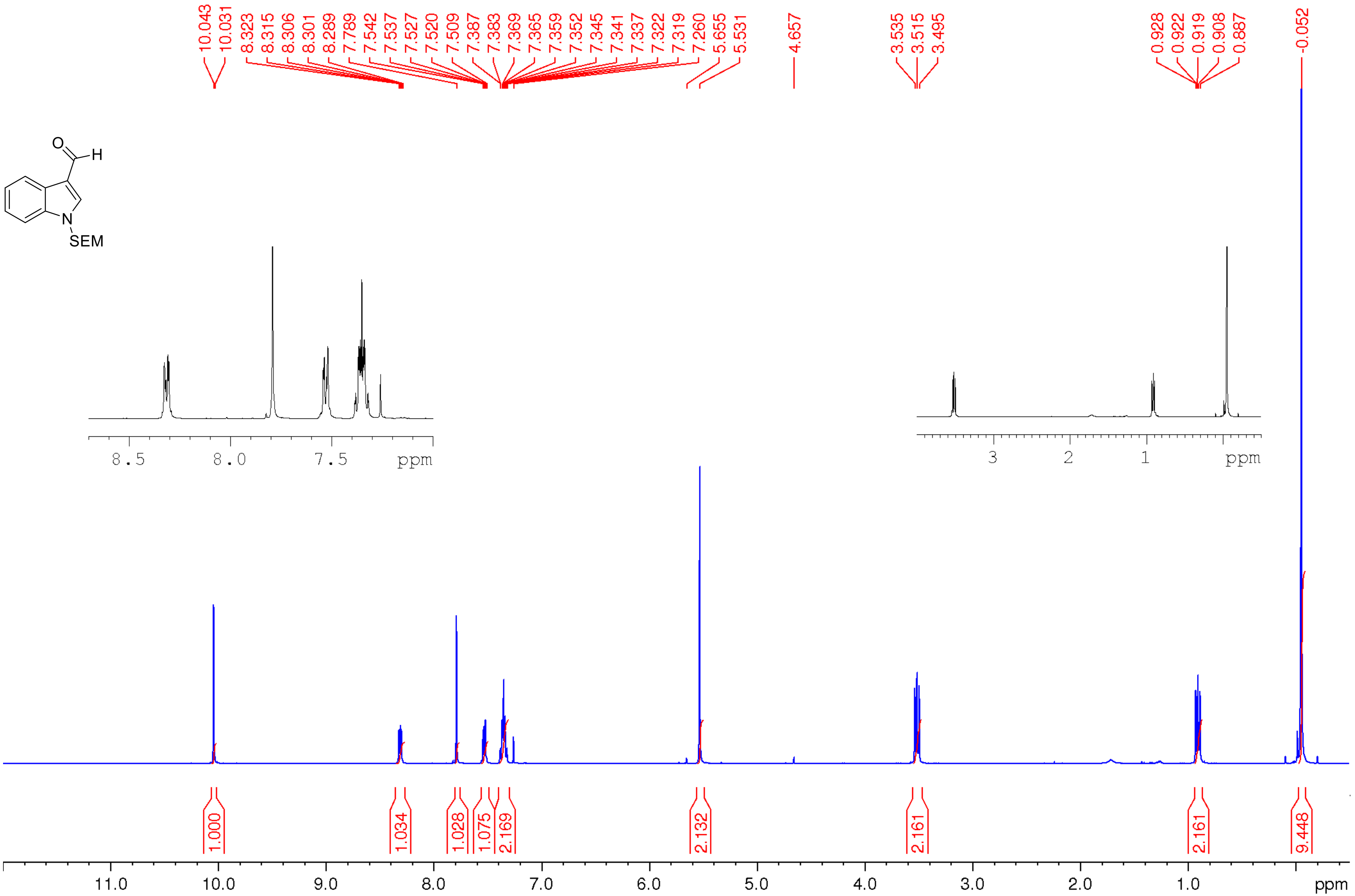

1-tosyl-1H-indole-3-carbaldehyde

^{13}C NMR-spectrum (100.6 MHz) ($CDCl_3$)

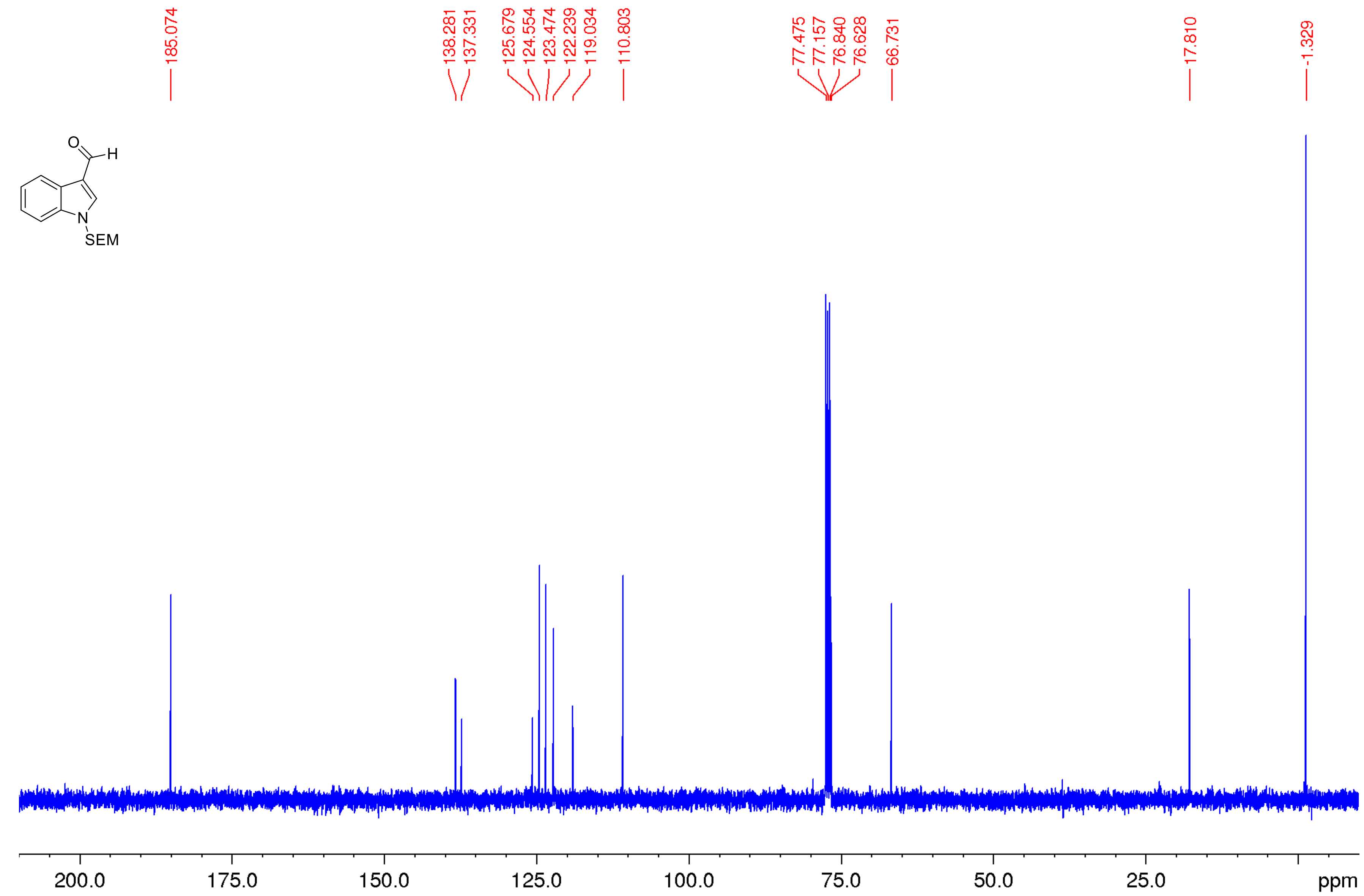

1-tosyl-1H-indole-3-carbaldehyde

DEPT 135 NMR-spectrum ($CDCl_3$)

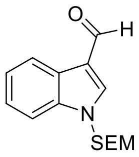

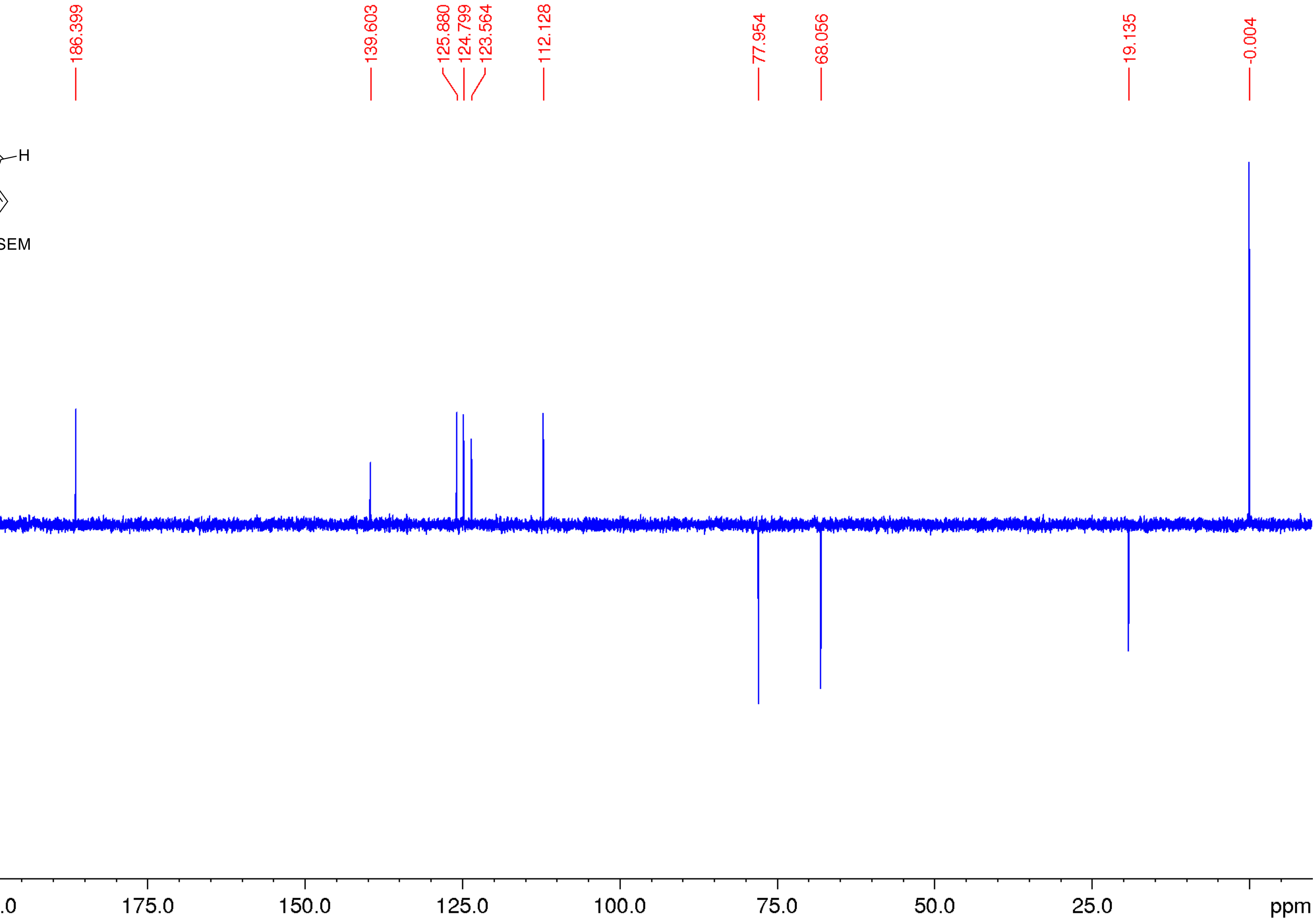

5-methoxy-1-tosyl-1H-indole-3-carbaldehyde

^{1}H NMR-spectrum (400.13 MHz) (DMSO-d_6)

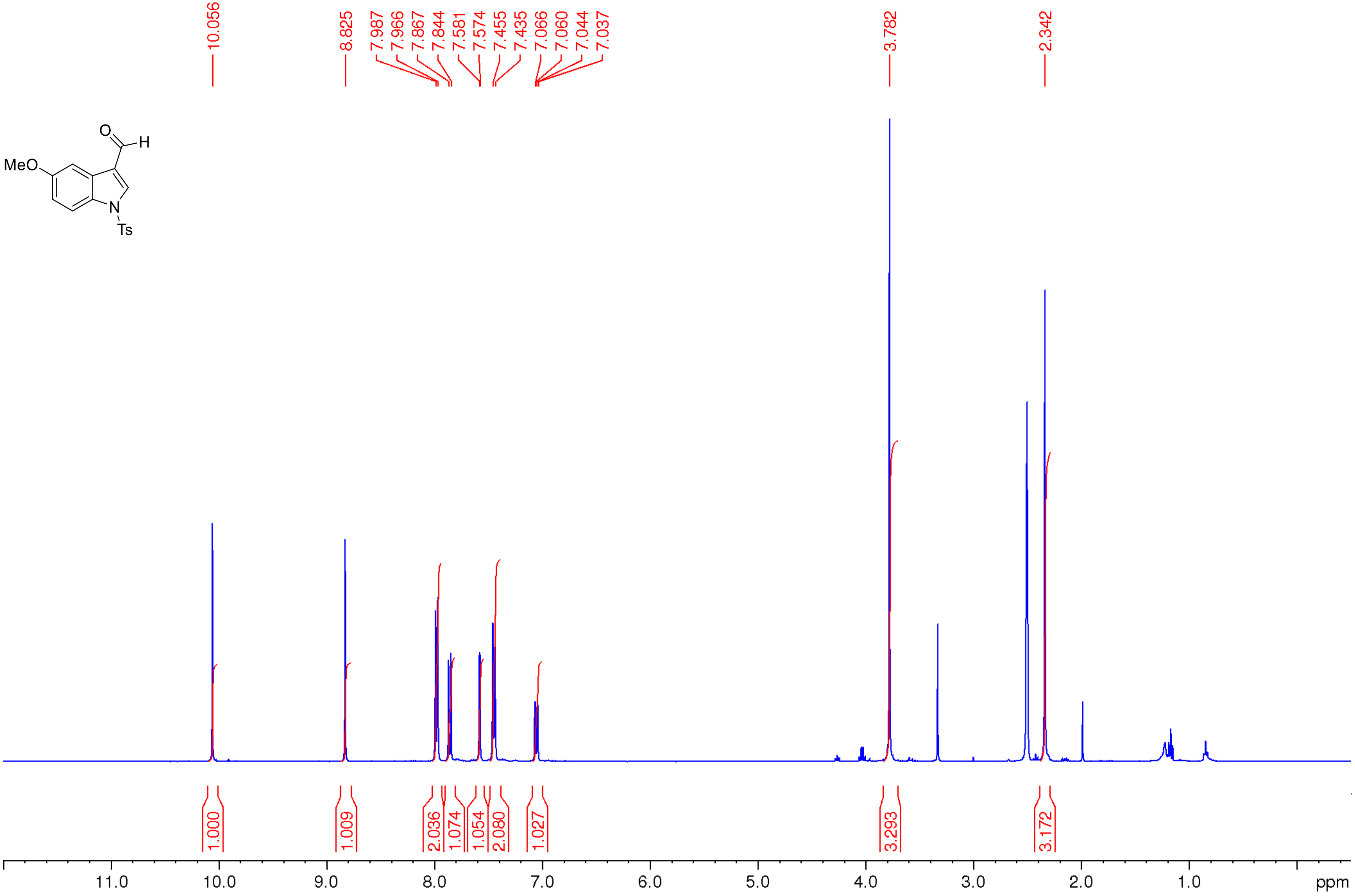

5-methoxy-1-tosyl-1H-indole-3-carbaldehyde

^{13}C NMR-spectrum (100.6 MHz) (DMSO-d_6)

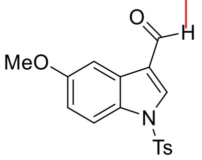

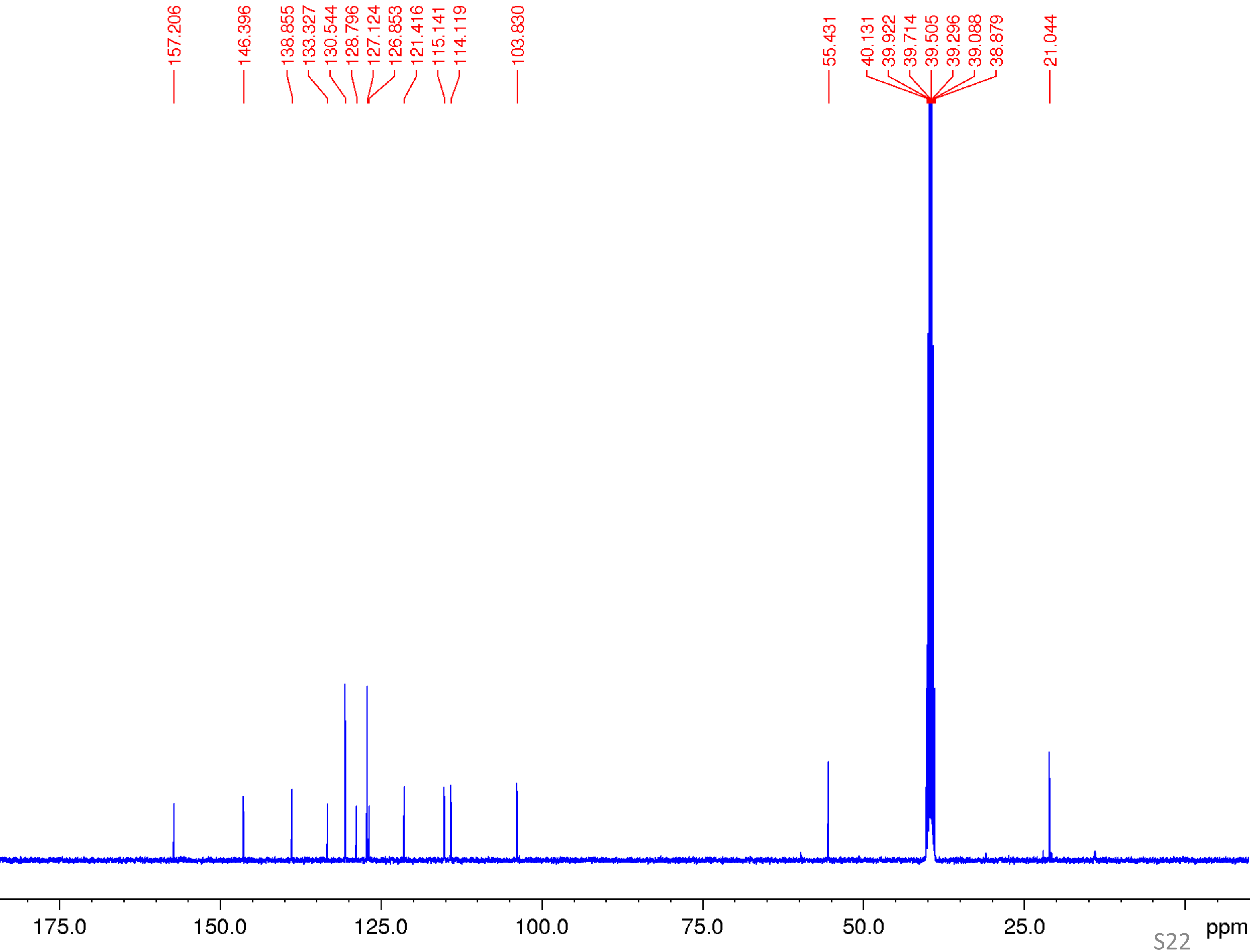

5-methoxy-1-tosyl-1H-indole-3-carbaldehyde

DEPT 135 NMR-spectrum (DMSO-d_6)

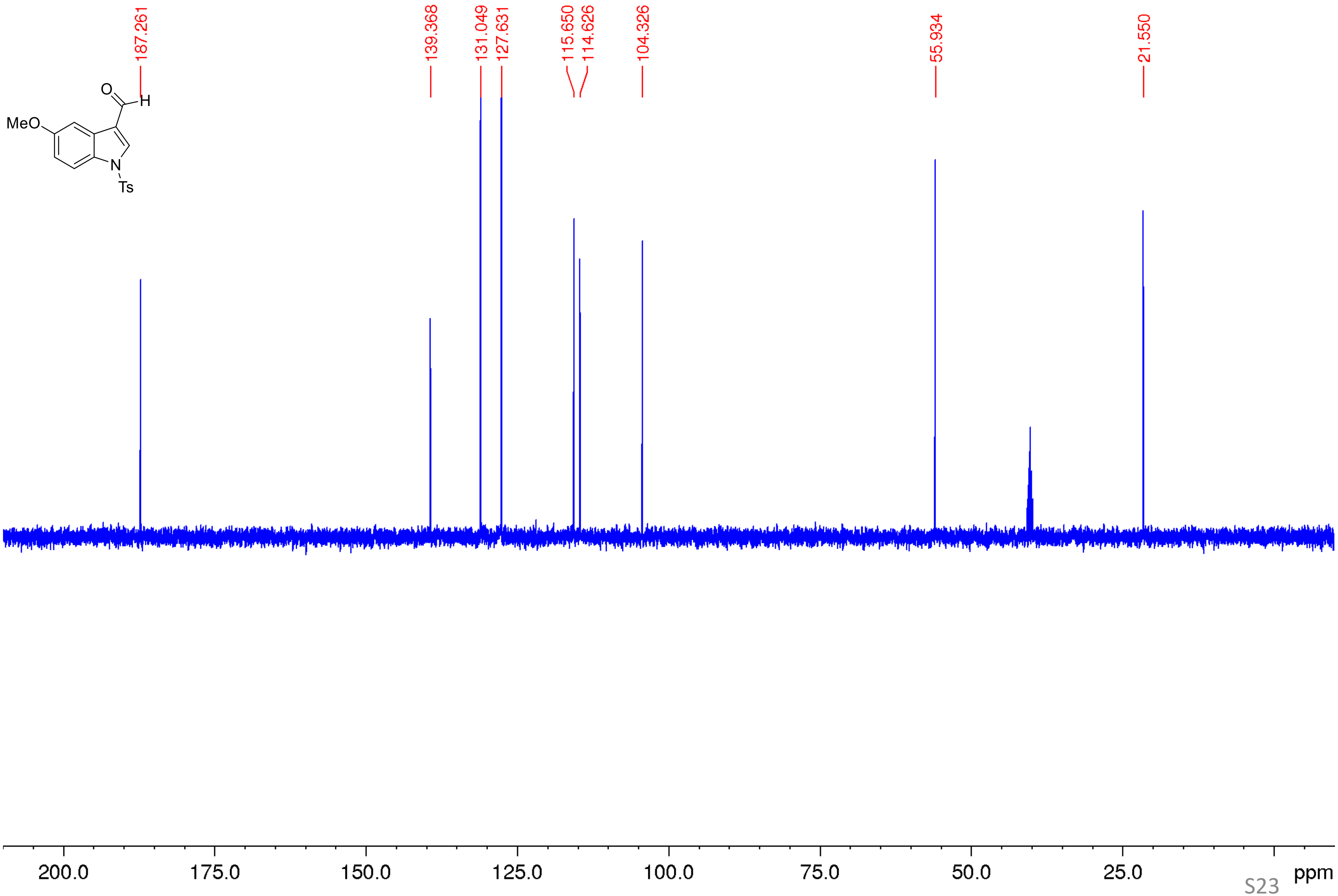

5-phenyl-1H-indole-3-carbaldehyde

^{1}H NMR-spectrum (400.13 MHz) (DMSO-d_6)

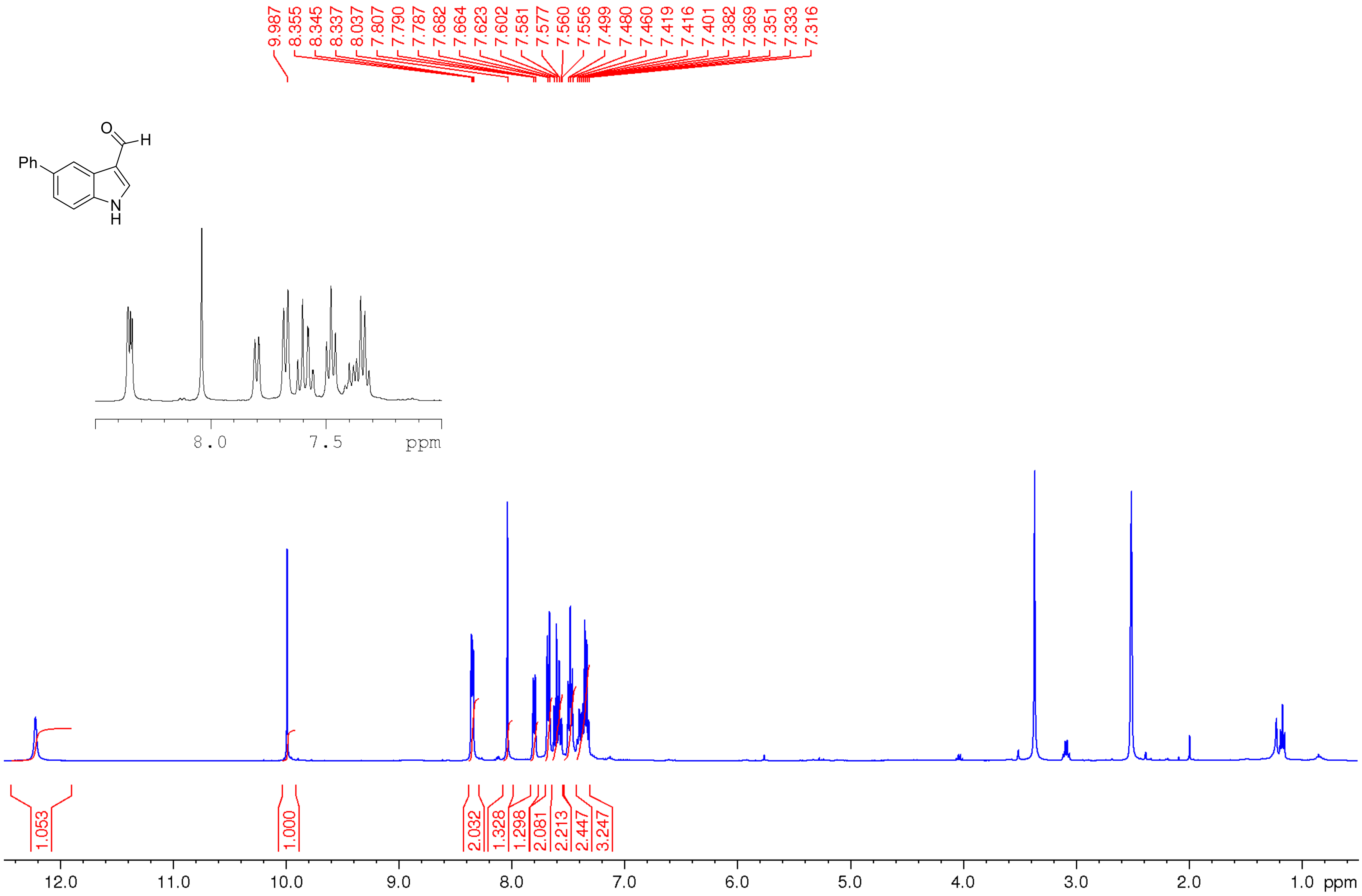

5-phenyl-1H-indole-3-carbaldehyde

^{13}C NMR-spectrum (100.6 MHz) (DMSO-d_6)

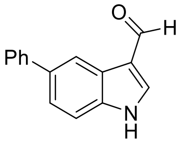

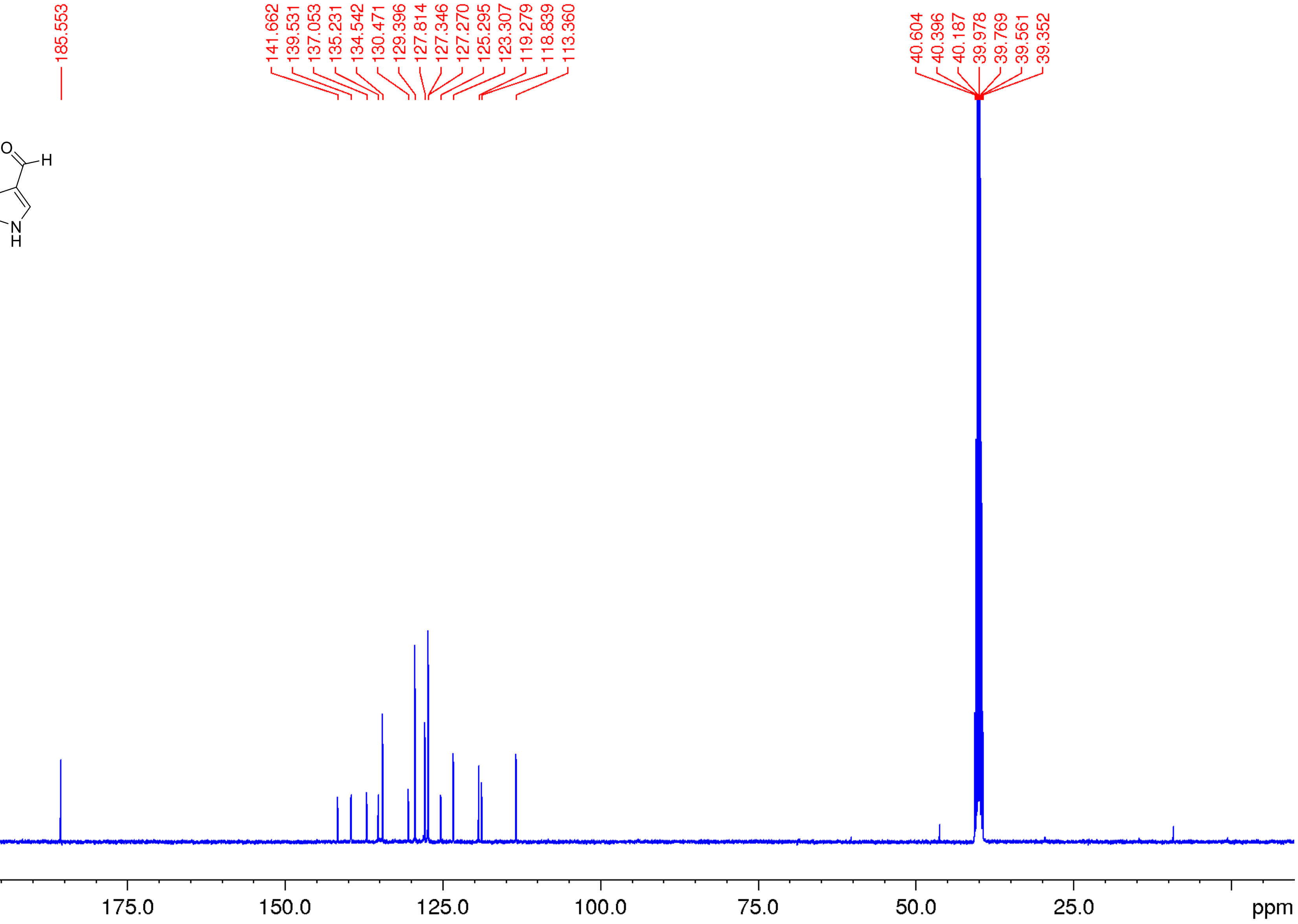

5-phenyl-1H-indole-3-carbaldehyde

DEPT 135 NMR-spectrum (DMSO-d_6)

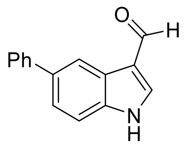

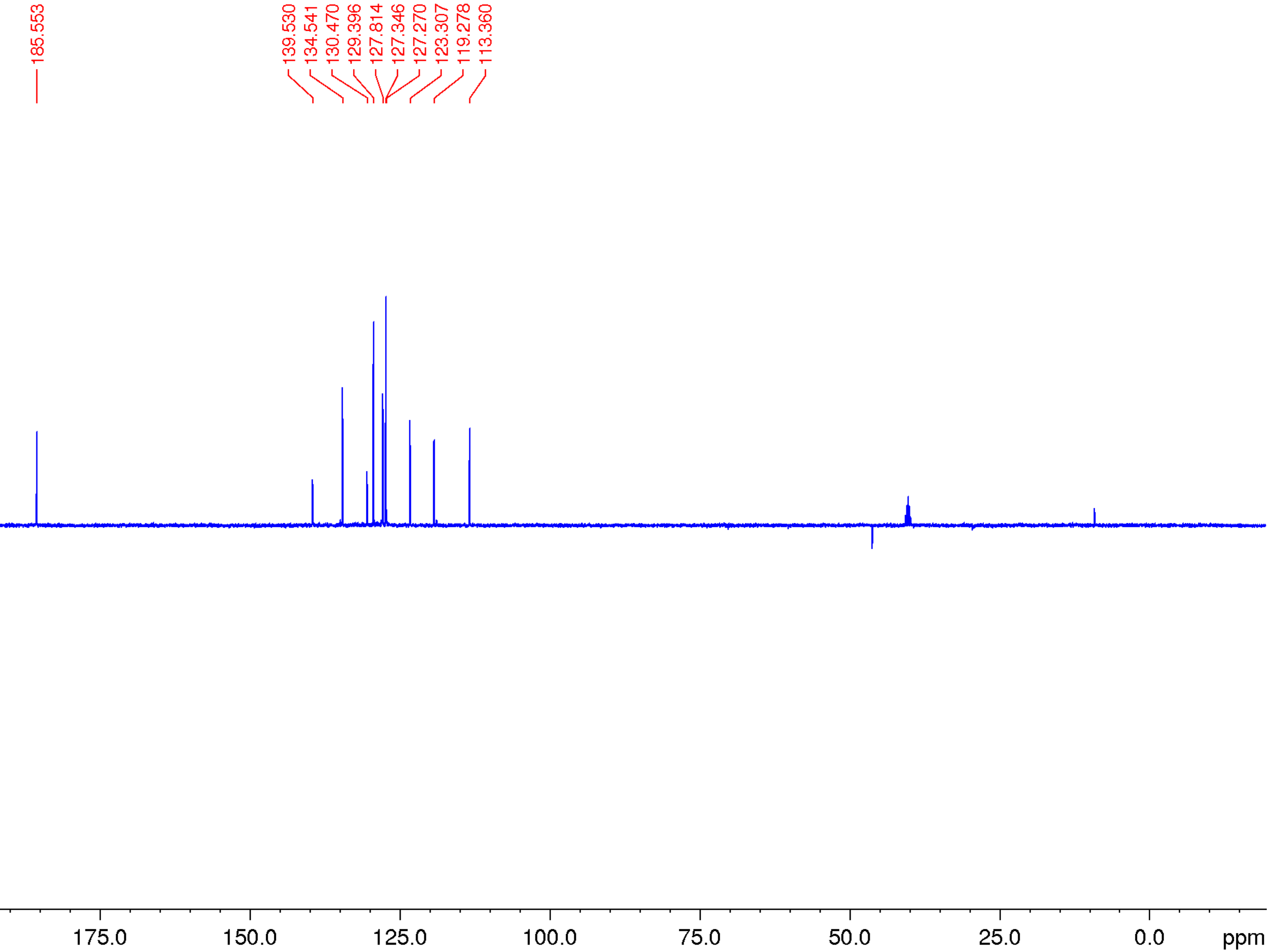

5-phenyl-1-tosyl-1H-indole-3-carbaldehyde

^{1}H NMR-spectrum (400.13 MHz) ($CDCl_3$)

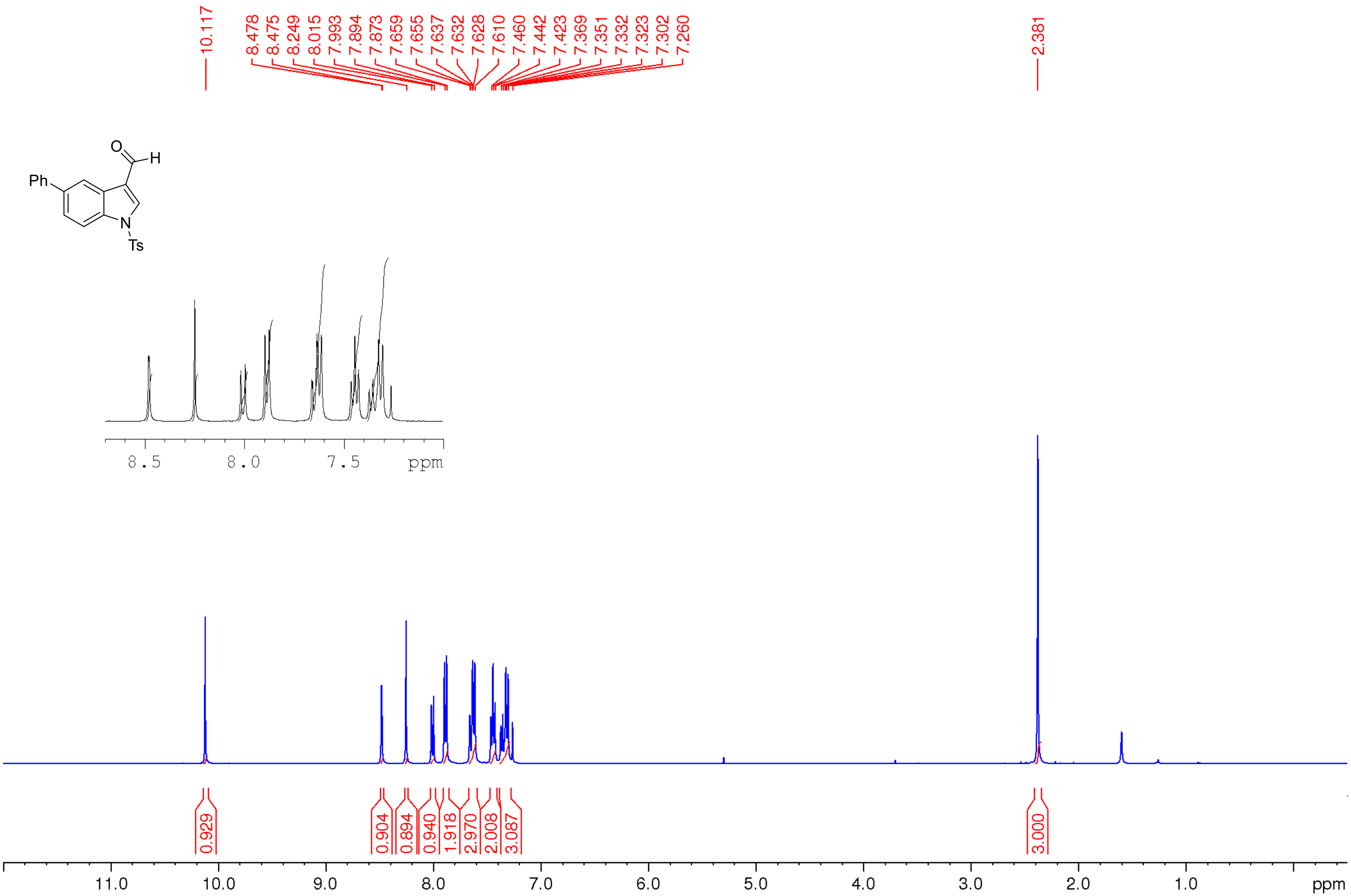

5-phenyl-1-tosyl-1H-indole-3-carbaldehyde

^{13}C NMR-spectrum (100.6 MHz) ($CDCl_3$)

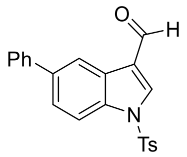

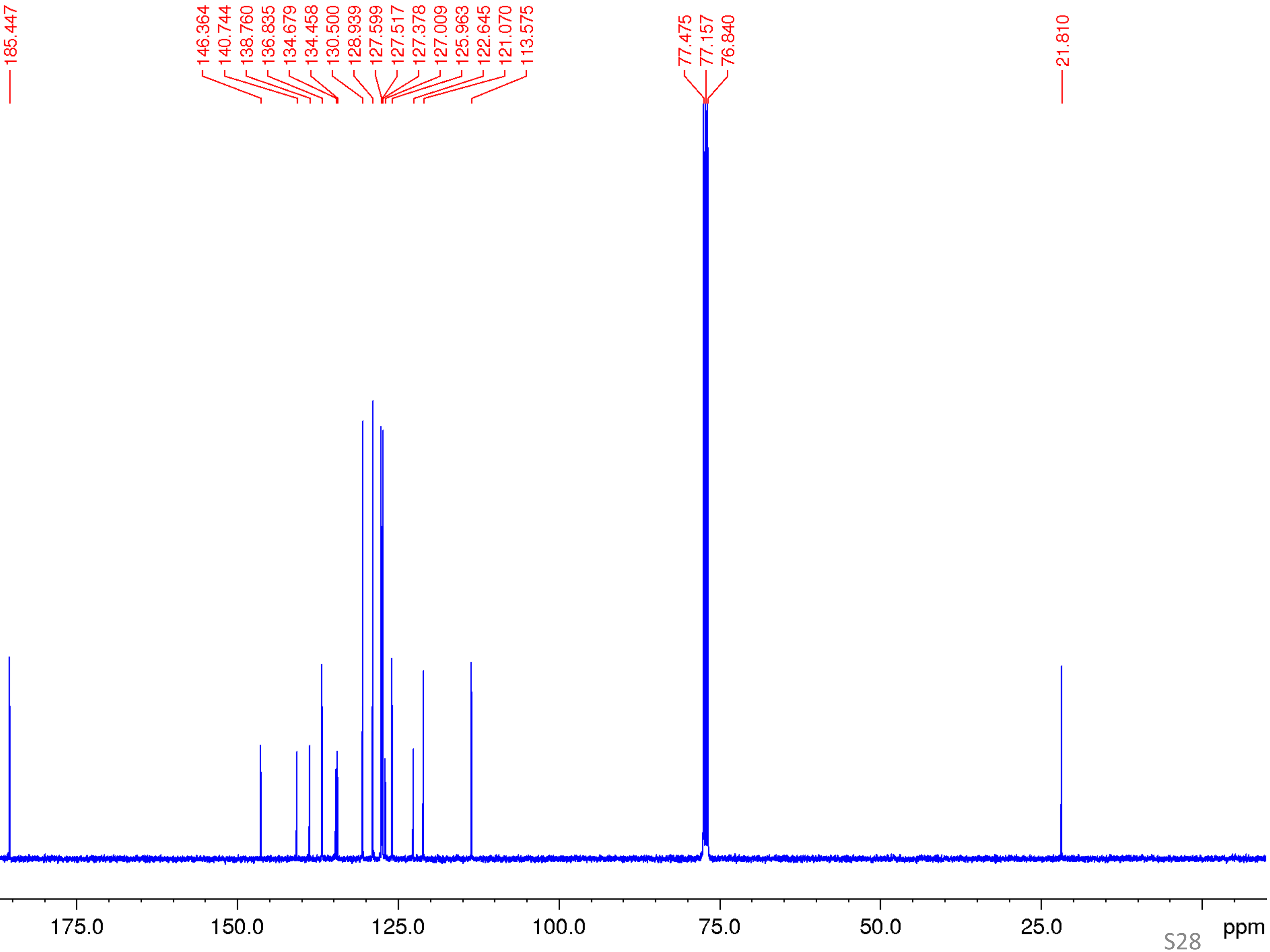

5-phenyl-1-tosyl-1H-indole-3-carbaldehyde

DEPT 135 NMR-spectrum ($CDCl_3$)

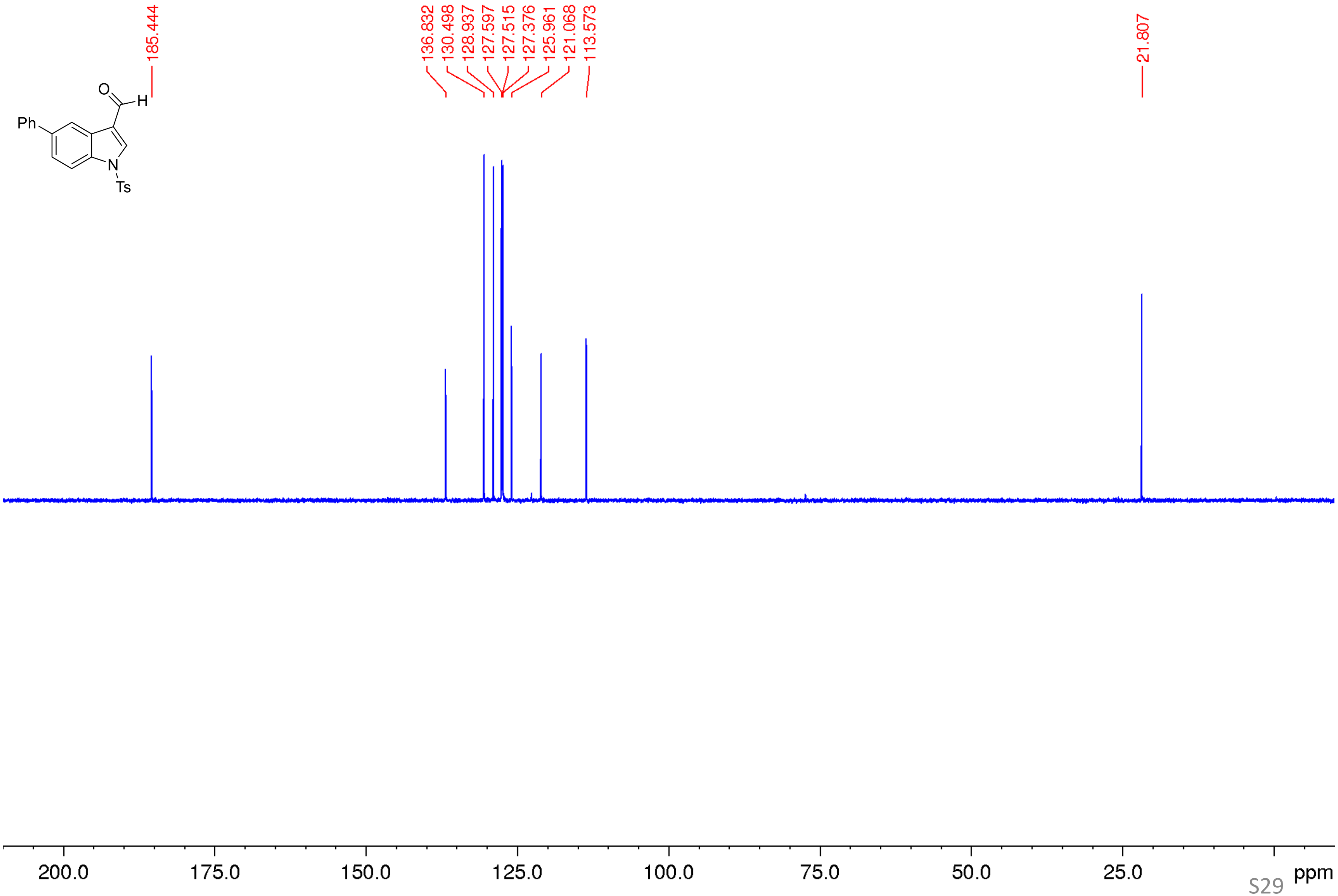

6-chloro-1-tosyl-1H-indole-3-carbaldehyde

^{1}H NMR-spectrum (400.13 MHz) ($CDCl_3$)

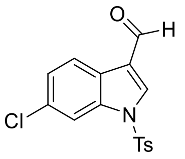

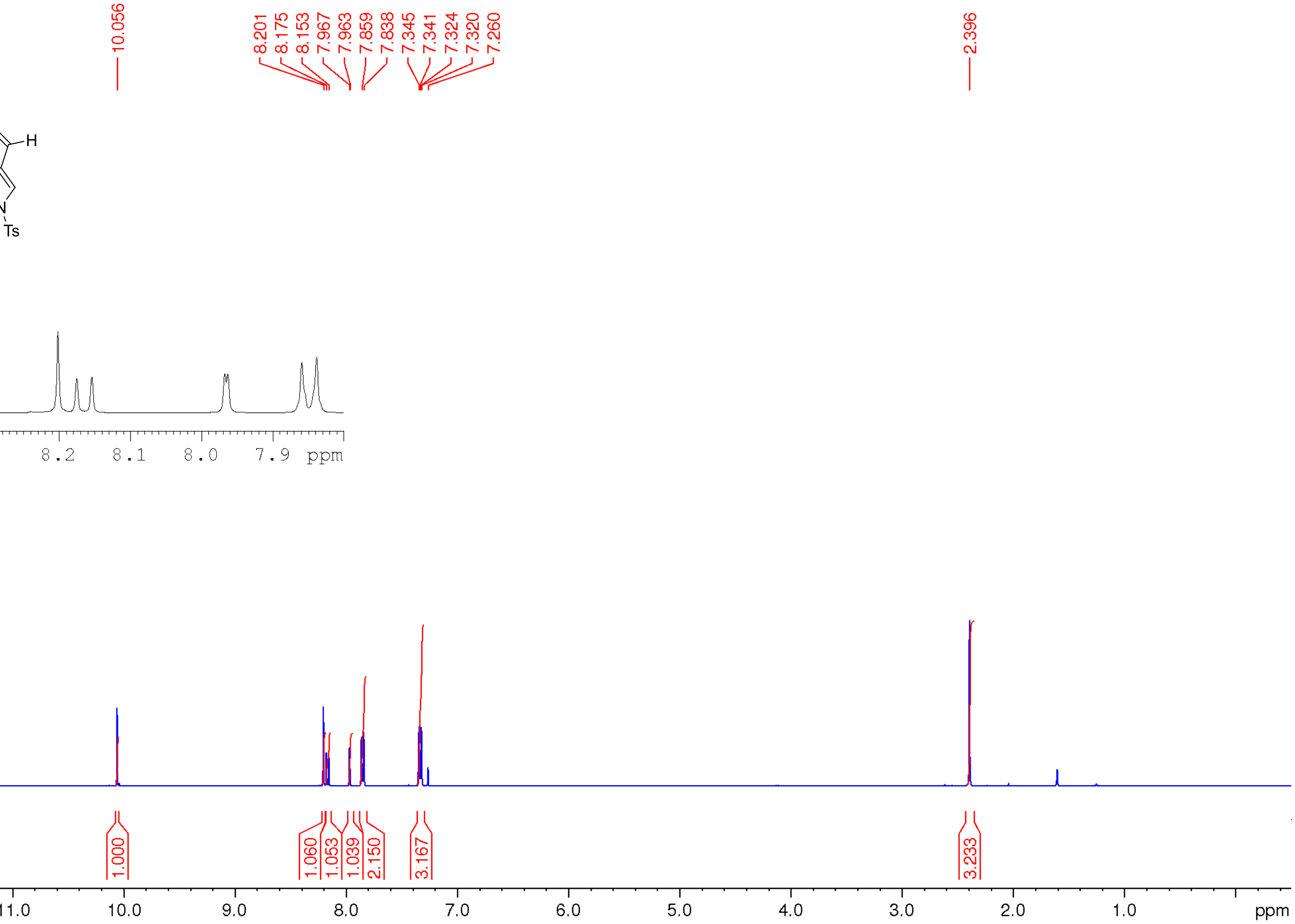

6-chloro-1-tosyl-1H-indole-3-carbaldehyde

^{13}C NMR-spectrum (100.6 MHz) ($CDCl_3$)

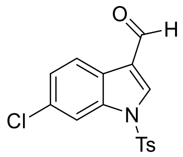

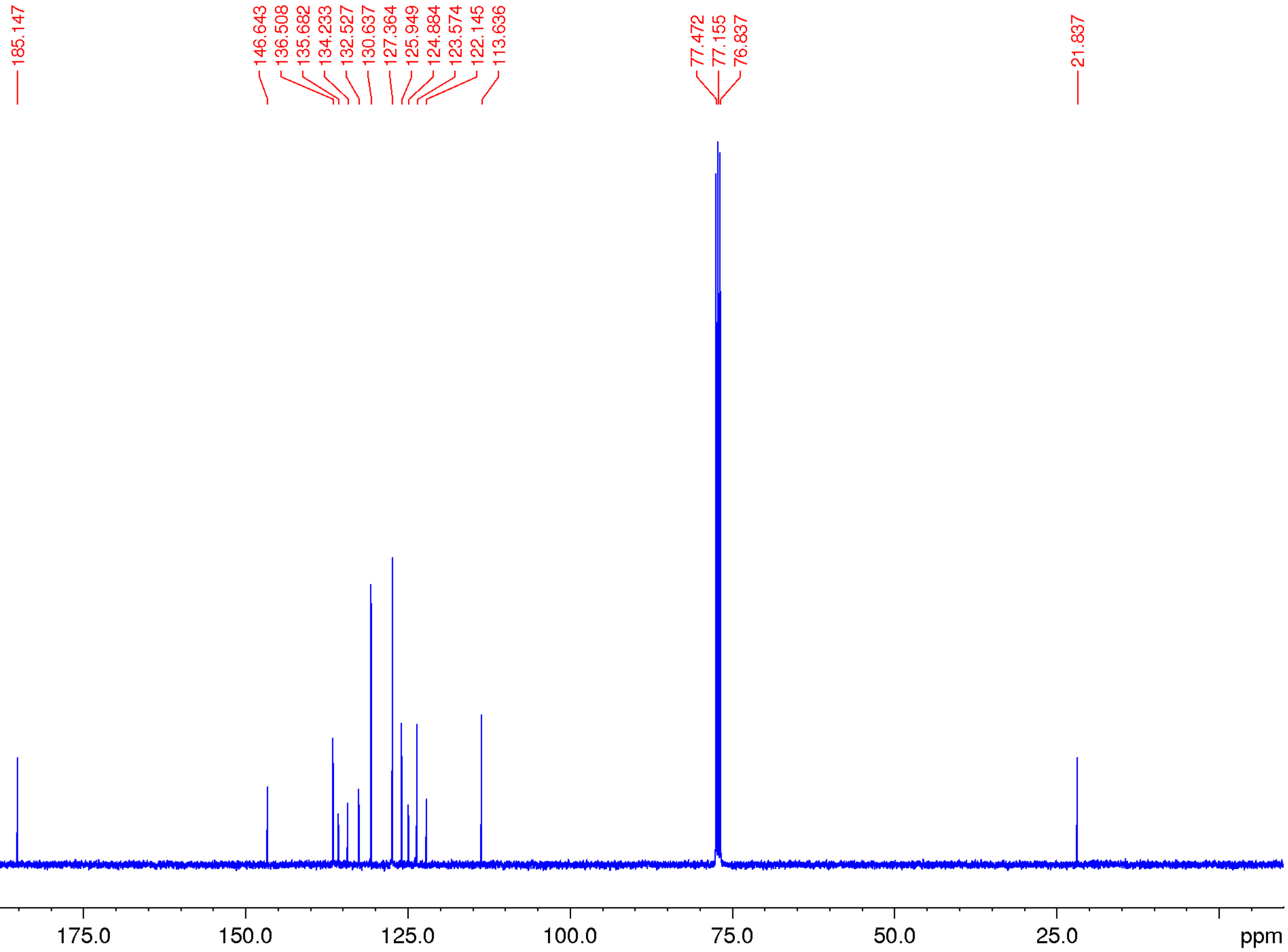

6-chloro-1-tosyl-1H-indole-3-carbaldehyde

DEPT 135 NMR-spectrum ($CDCl_3$)

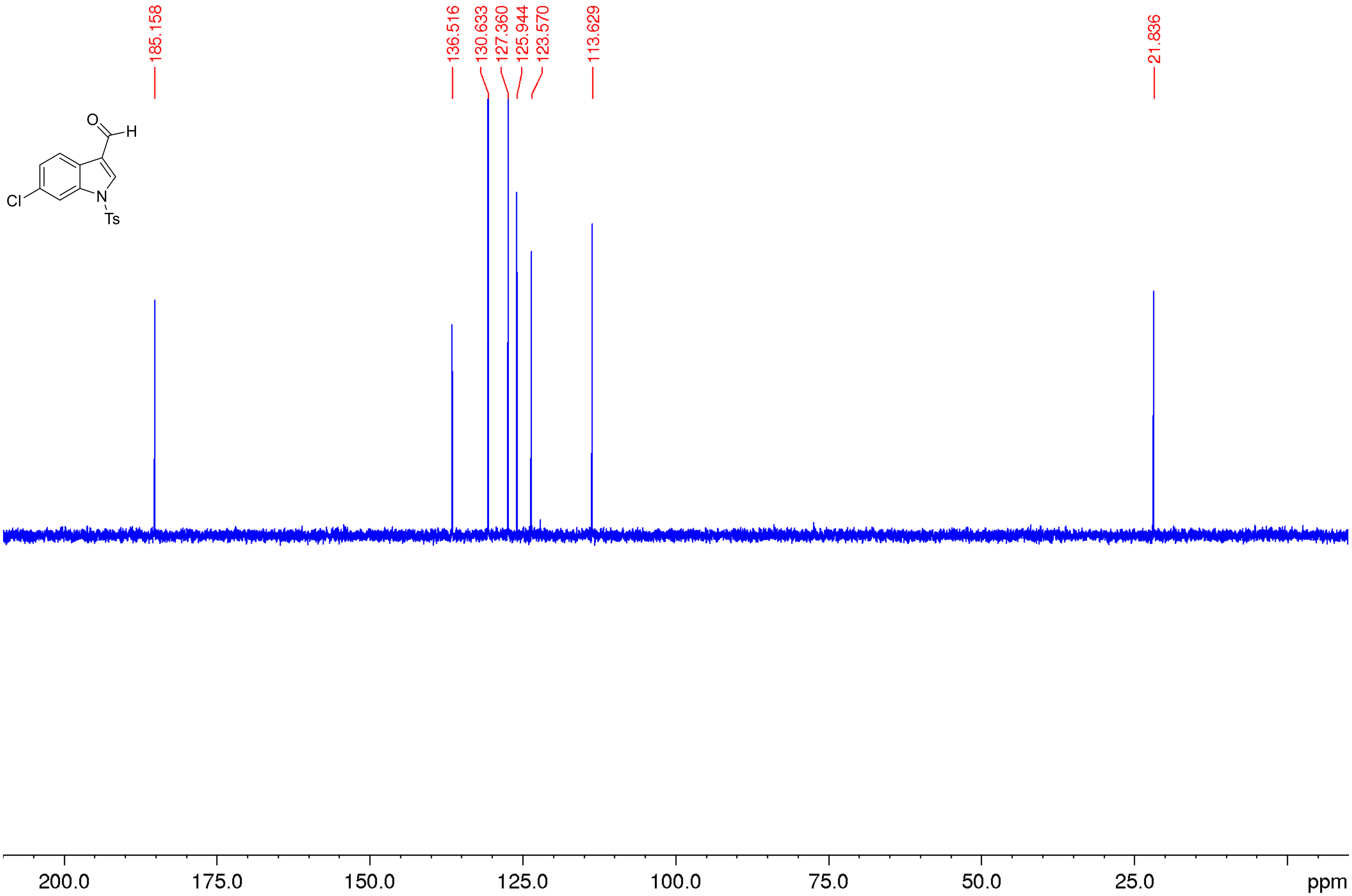

5-nitro-1-tosyl-1H-indole-3-carbaldehyde

^{1}H NMR-spectrum (400.13 MHz) (DMSO-d_6)

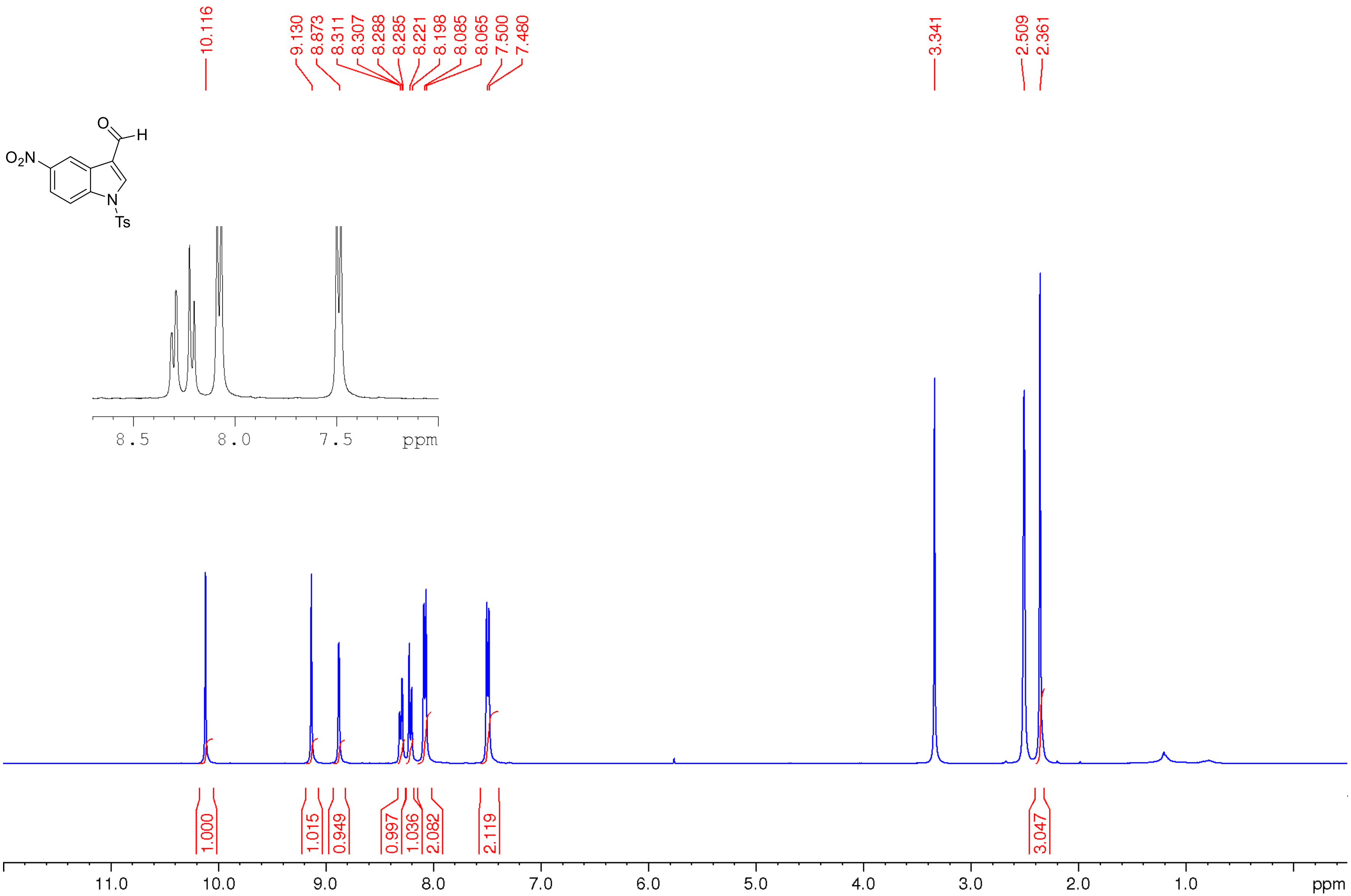

5-nitro-1-tosyl-1H-indole-3-carbaldehyde

^{13}C NMR-spectrum (100.6 MHz) (DMSO-d_6)

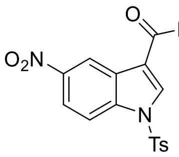

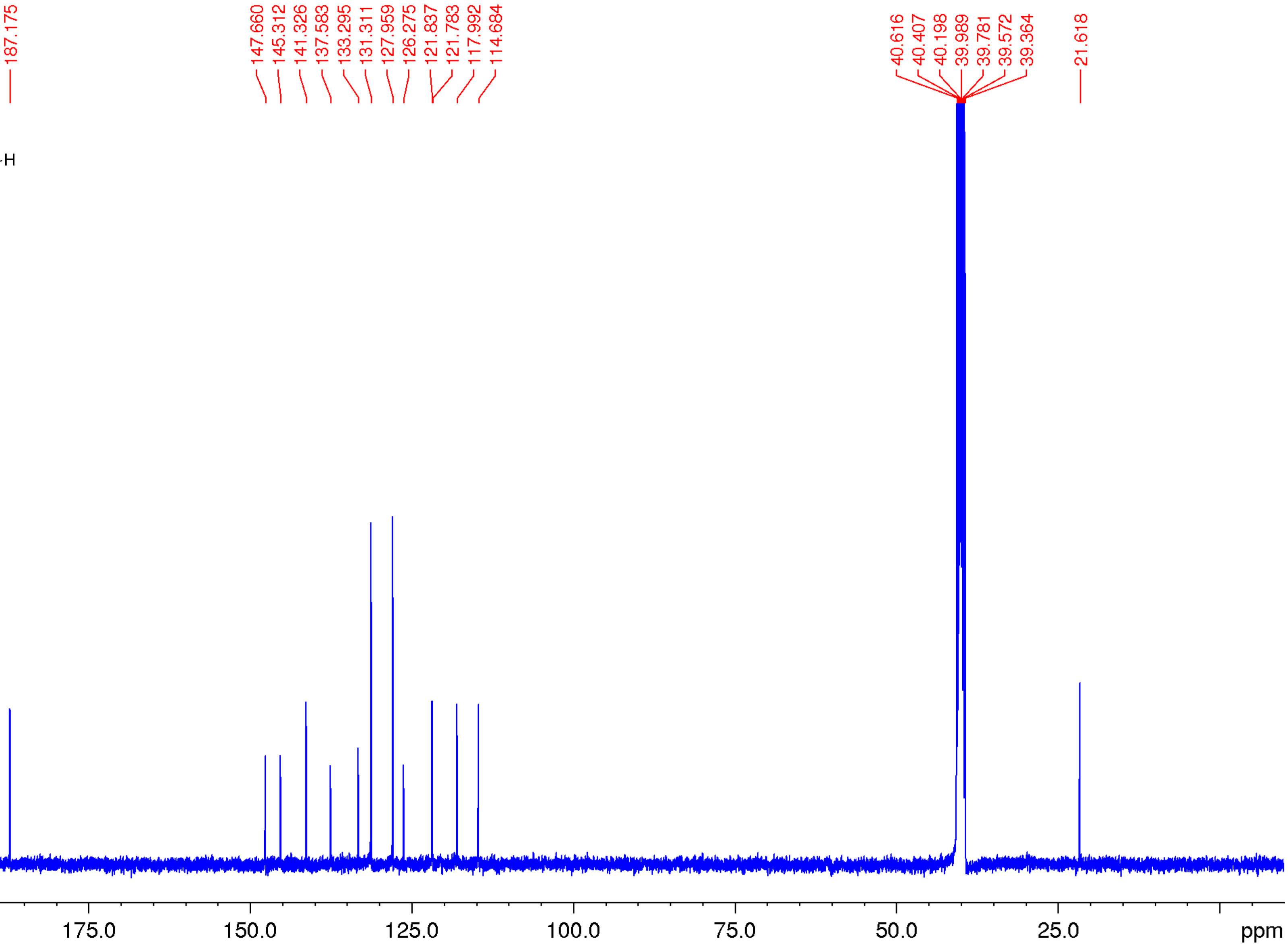

5-nitro-1-tosyl-1H-indole-3-carbaldehyde

DEPT 135 NMR-spectrum (DMSO-d_6)

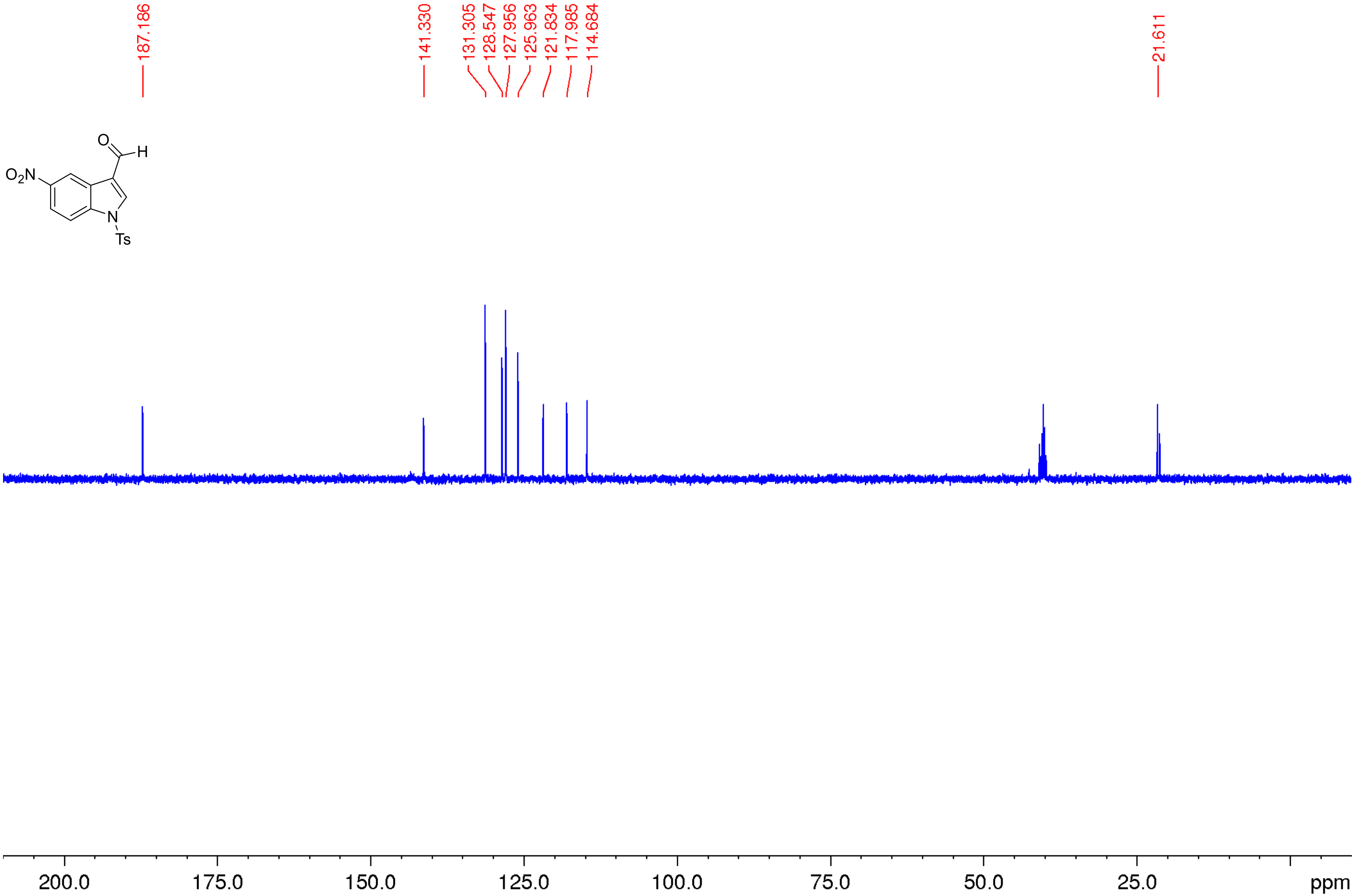

(1-tosyl-1H-indol-3-yl)methanol

^{1}H NMR-spectrum (400.13 MHz) ($CDCl_3$)

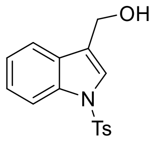

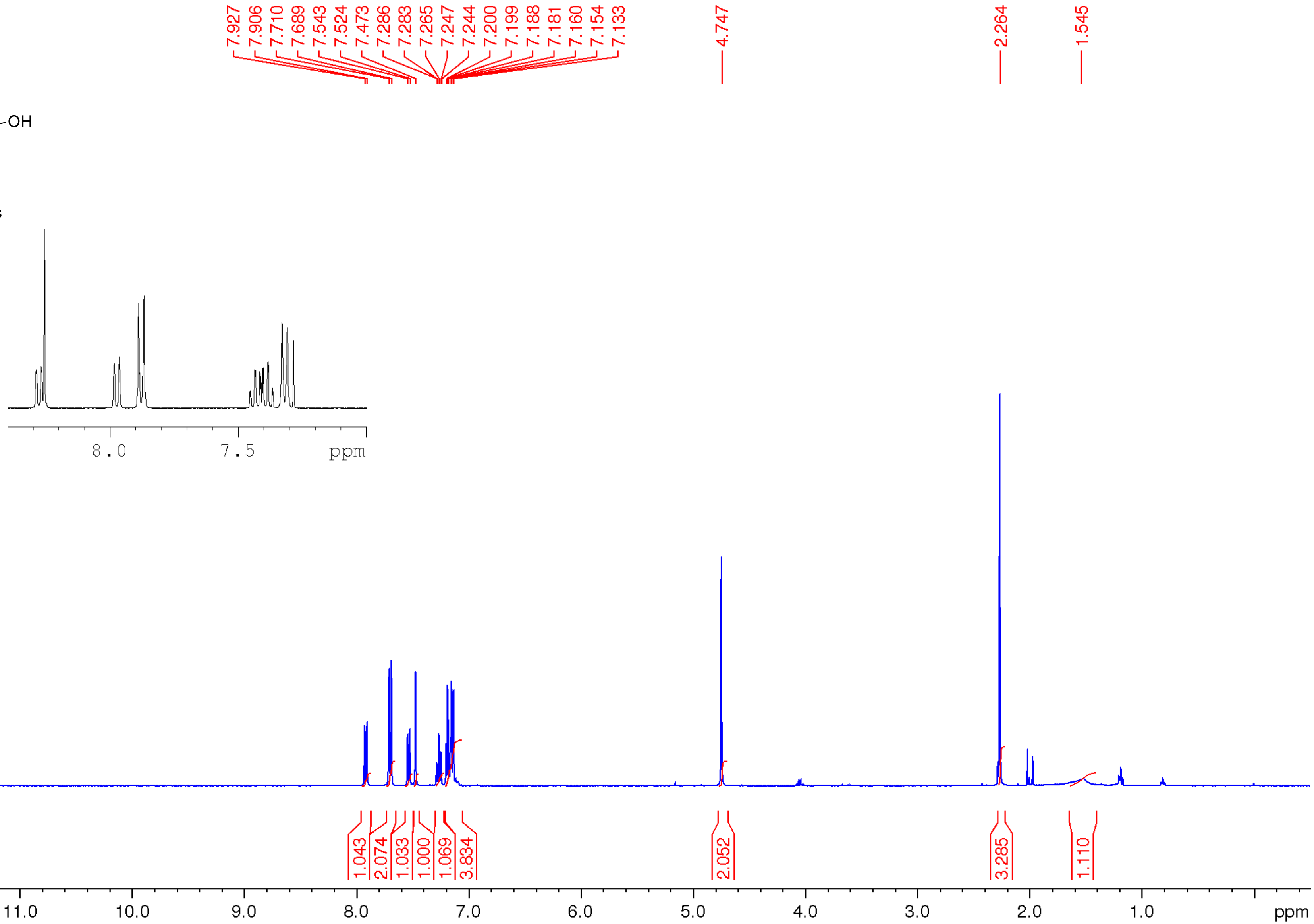

(1-tosyl-1H-indol-3-yl)methanol

^{13}C NMR-spectrum (100.6 MHz) ($CDCl_3$)

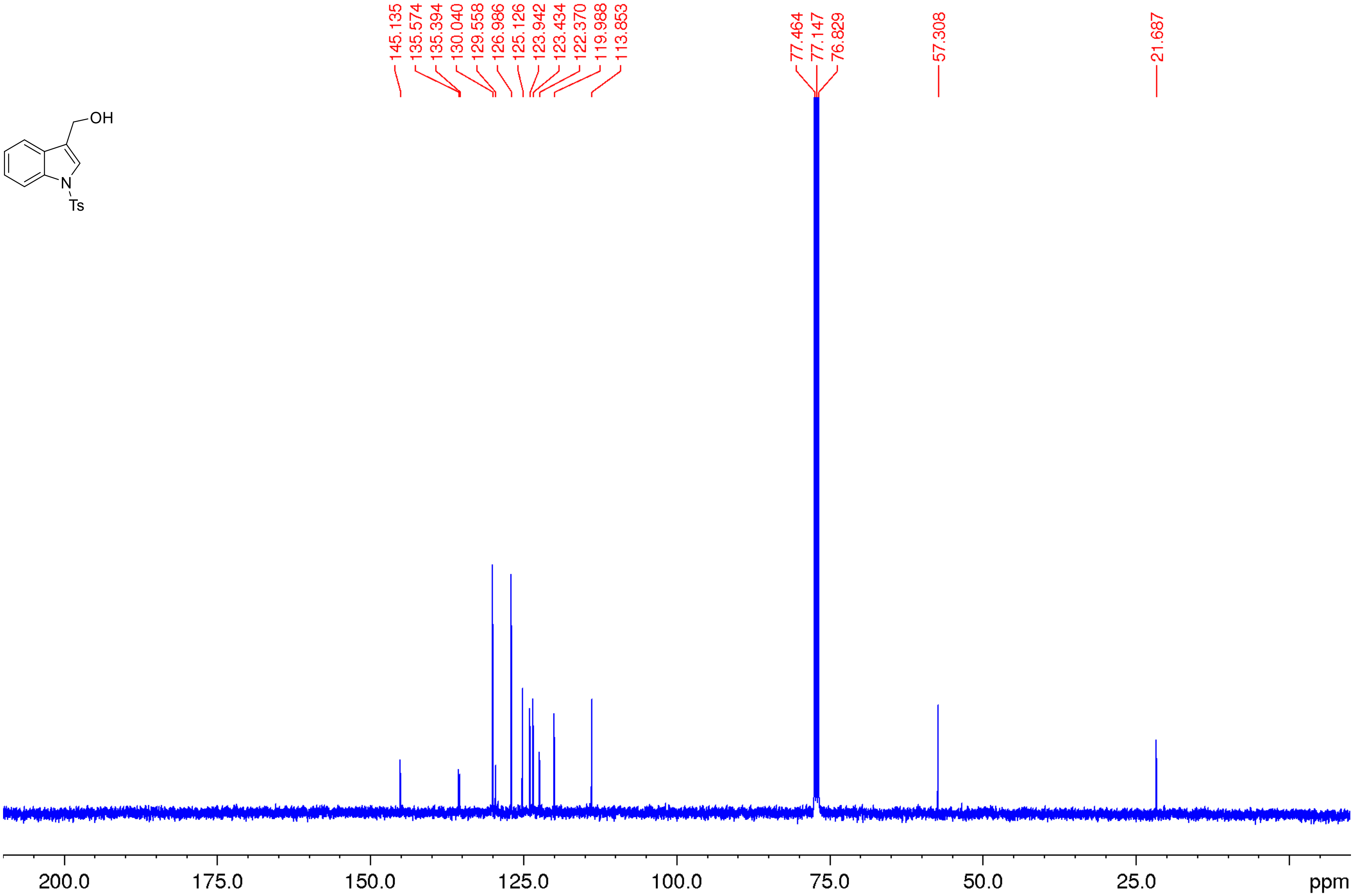

(1-tosyl-1H-indol-3-yl)methanol

DEPT 135 NMR-spectrum ($CDCl_3$)

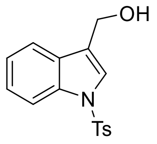

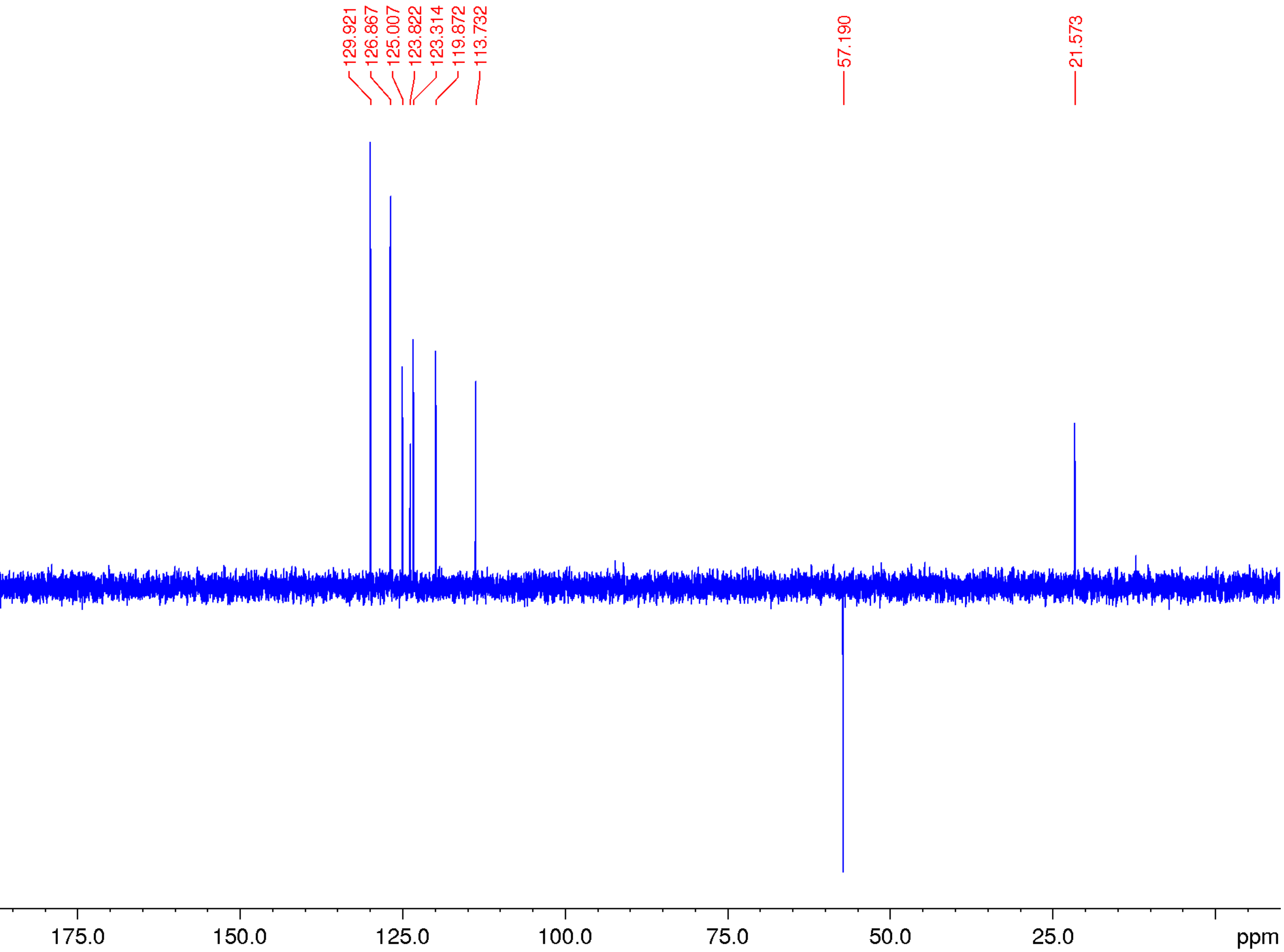

(1-tosyl-1H-indol-3-yl)methanol

^{1}H NMR-spectrum (400.13 MHz) ($CDCl_3$)

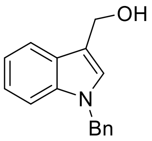

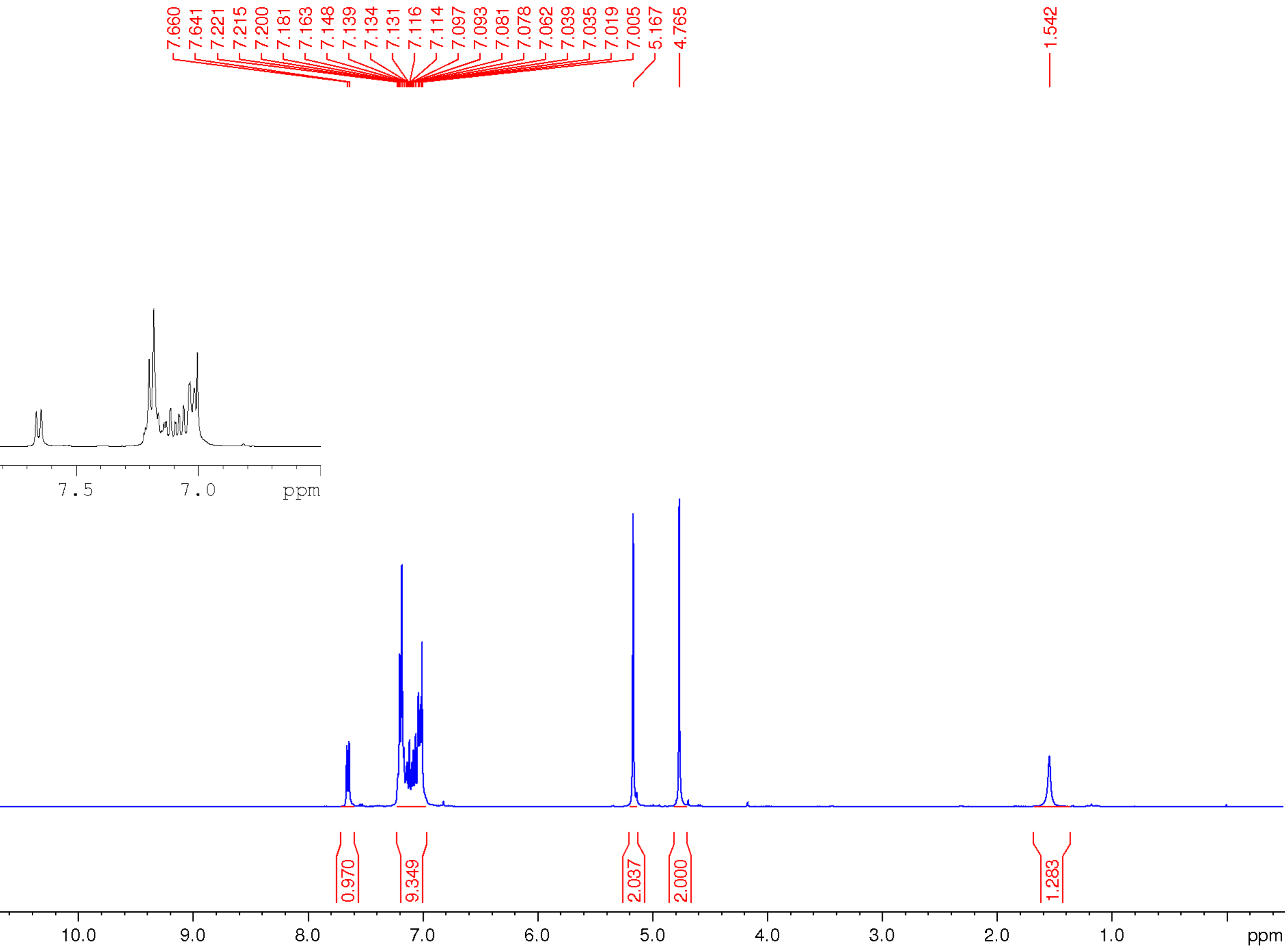

(1-tosyl-1H-indol-3-yl)methanol

^{13}C NMR-spectrum (100.6 MHz) ($CDCl_3$)

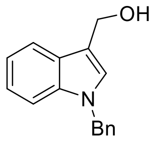

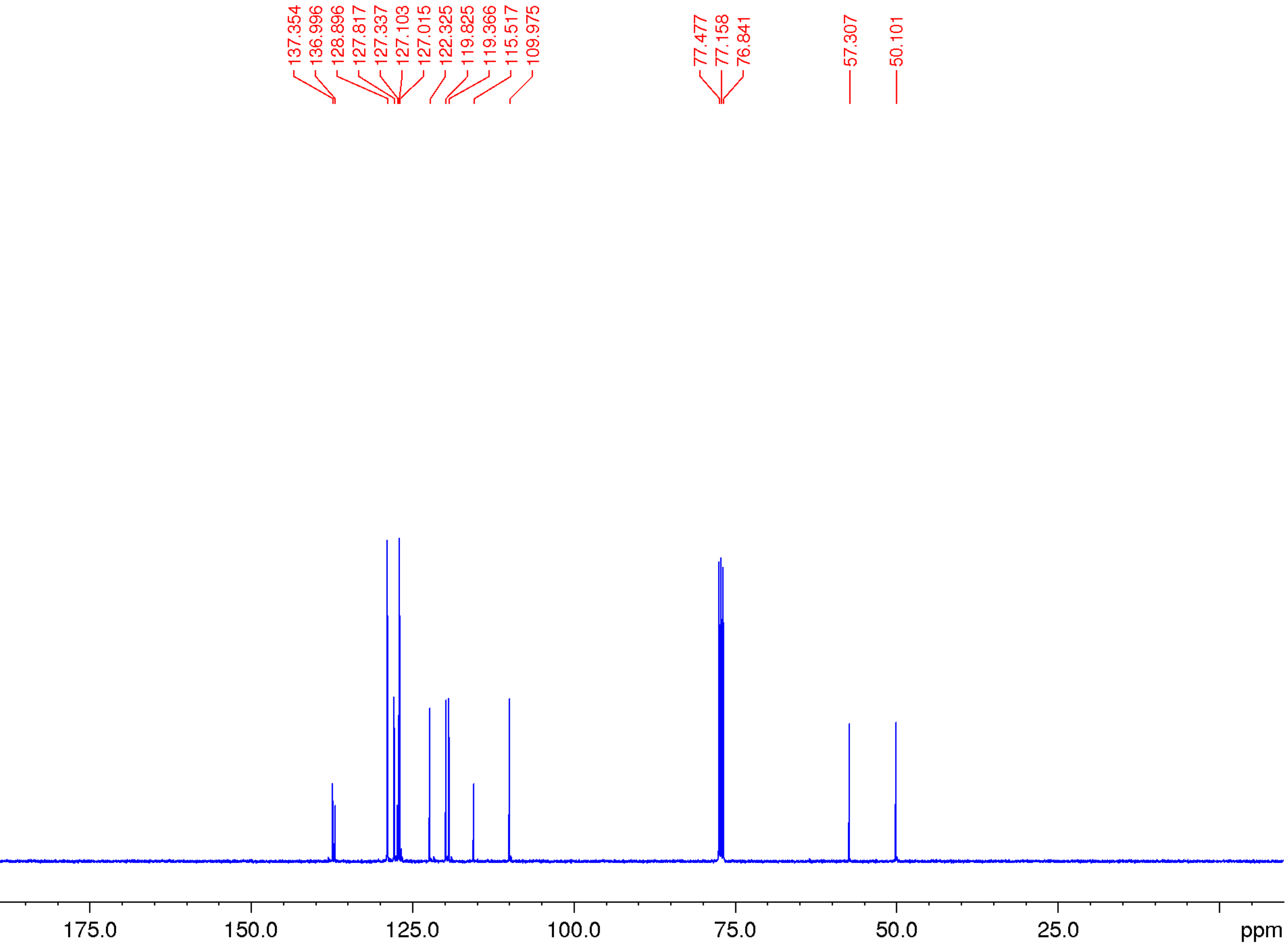

(1-tosyl-1H-indol-3-yl)methanol

DEPT 135 NMR-spectrum ($CDCl_3$)

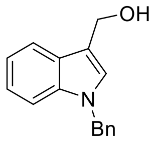

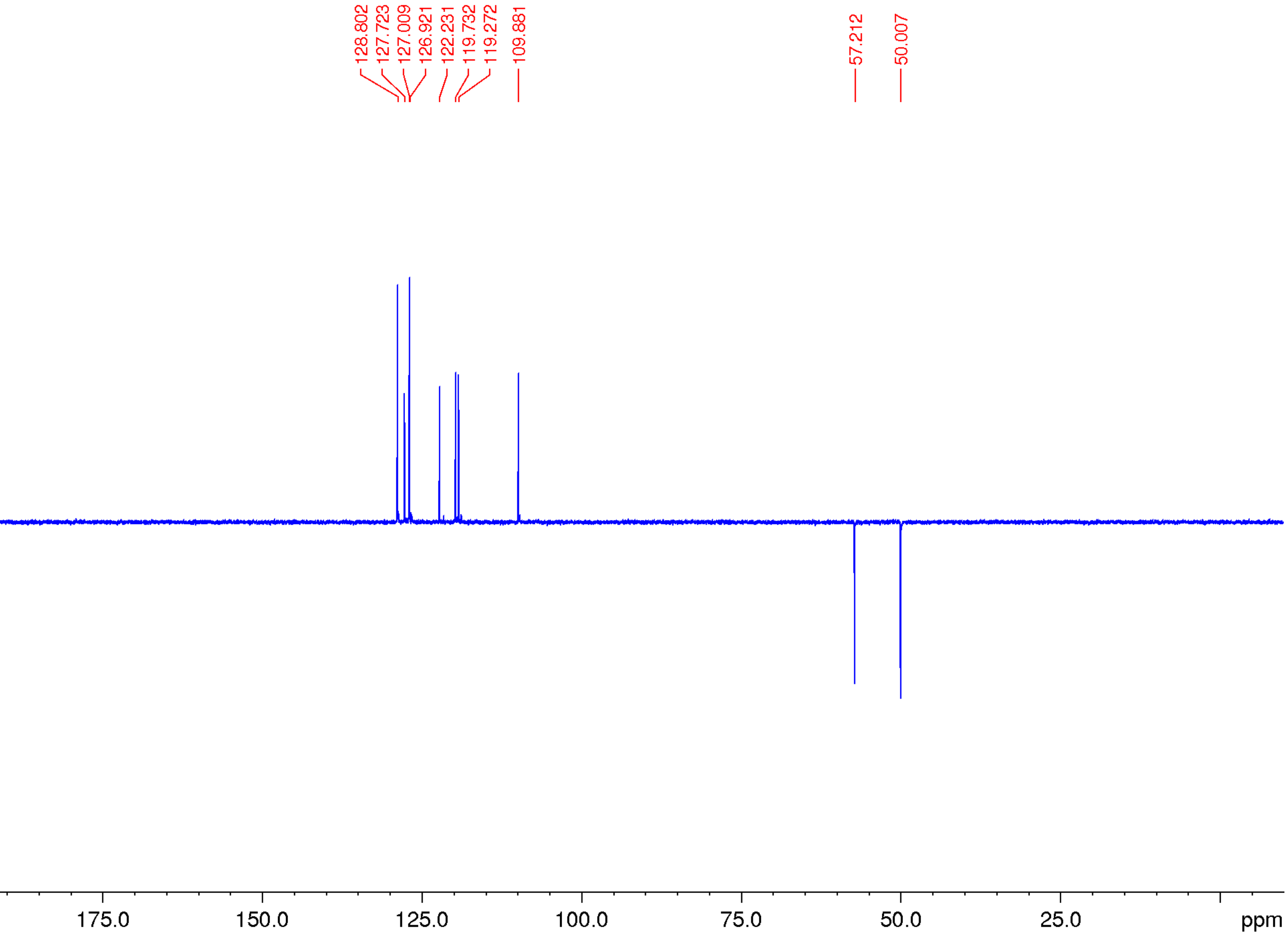

(1-tosyl-1H-indol-3-yl)methanol

^{1}H NMR-spectrum (400.13 MHz) (DMSO-d_6)

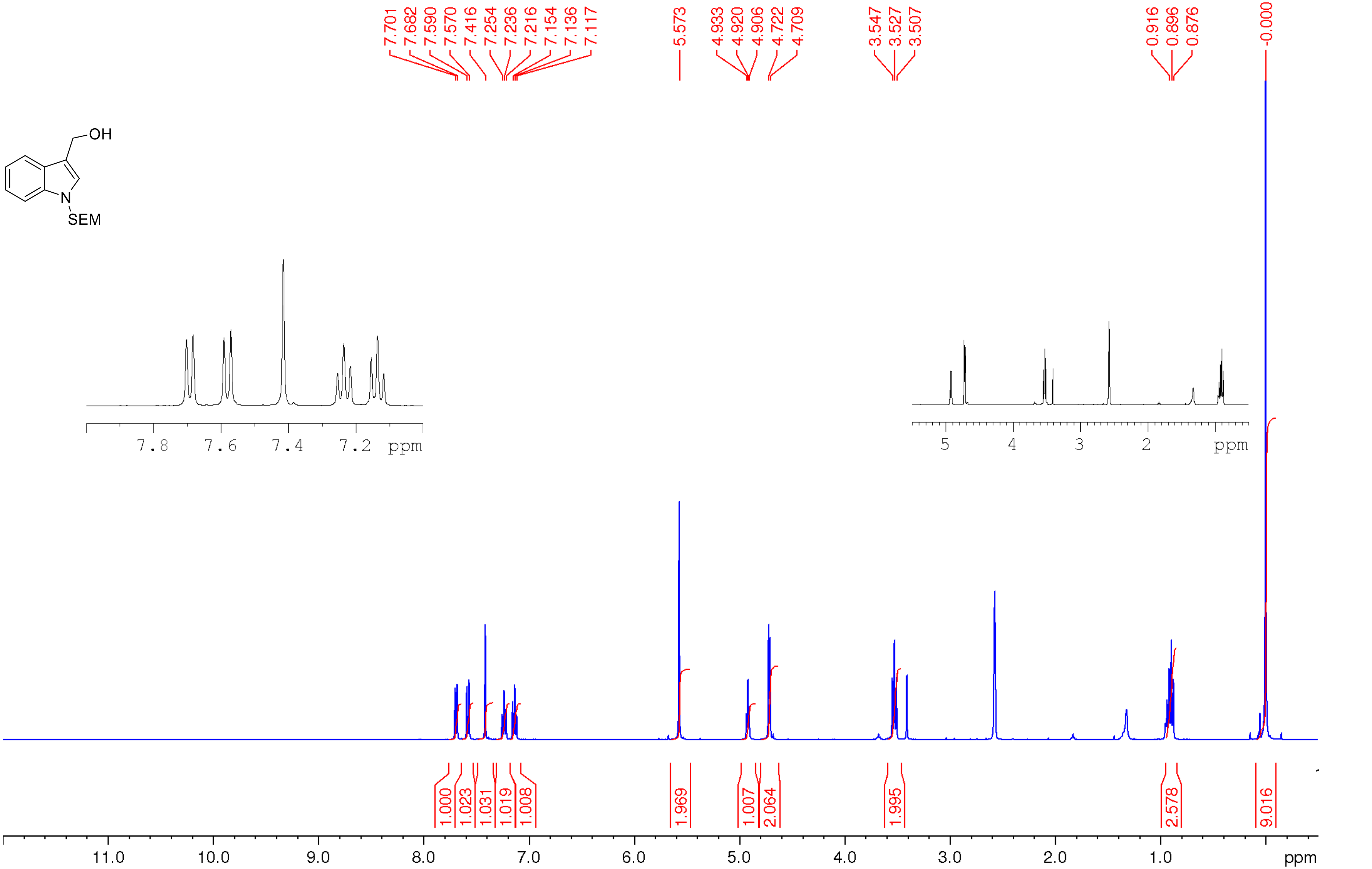

(1-tosyl-1H-indol-3-yl)methanol

^{13}C NMR-spectrum (100.6 MHz) ($CDCl_3$)

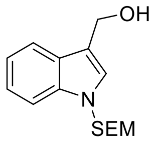

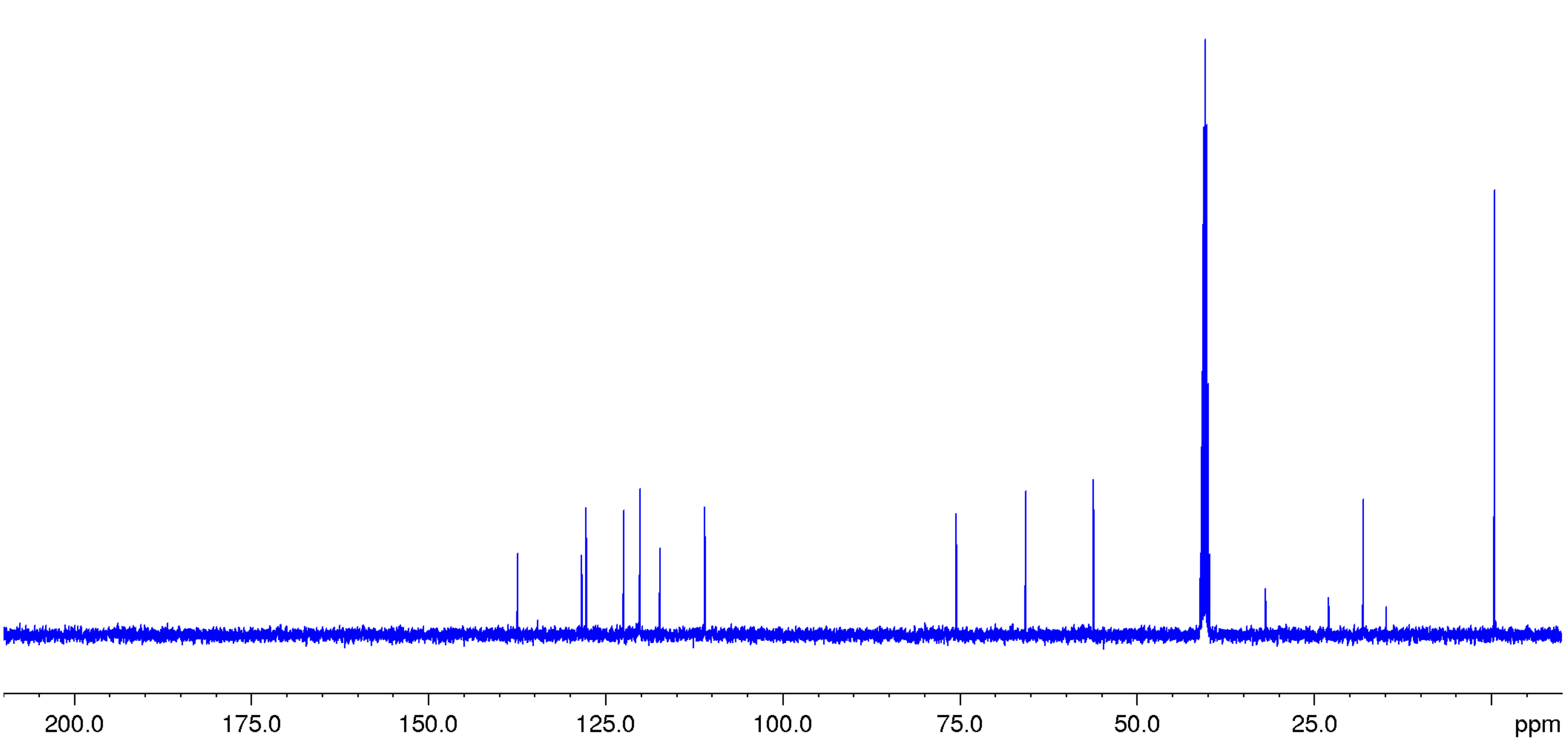

(1-tosyl-1H-indol-3-yl)methanol

DEPT 135 NMR-spectrum ($CDCl_3$)

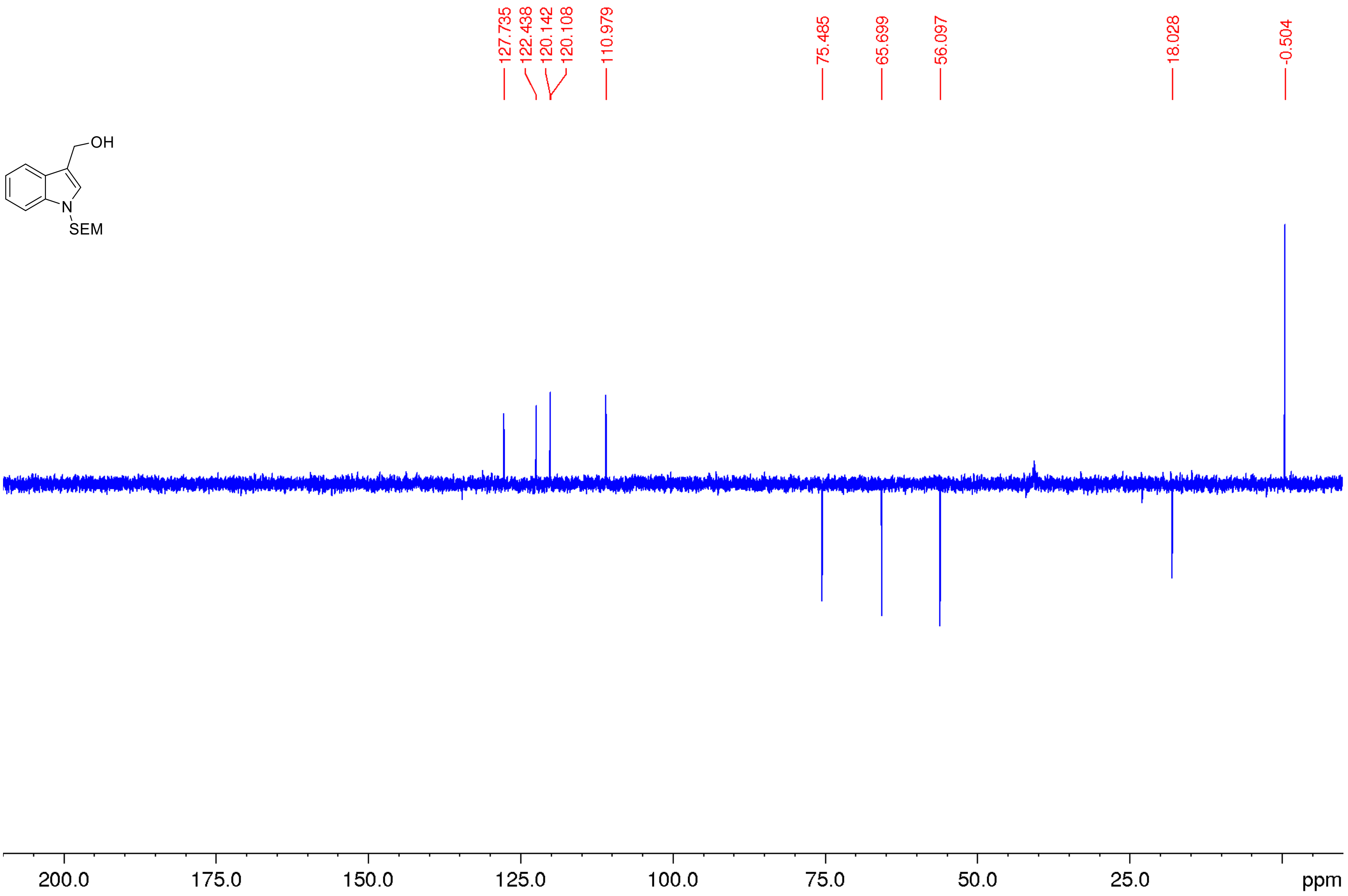

(5-methoxy-1-tosyl-1H-indol-3-yl)methanol

^{1}H NMR-spectrum (400.13 MHz) ($CDCl_3$)

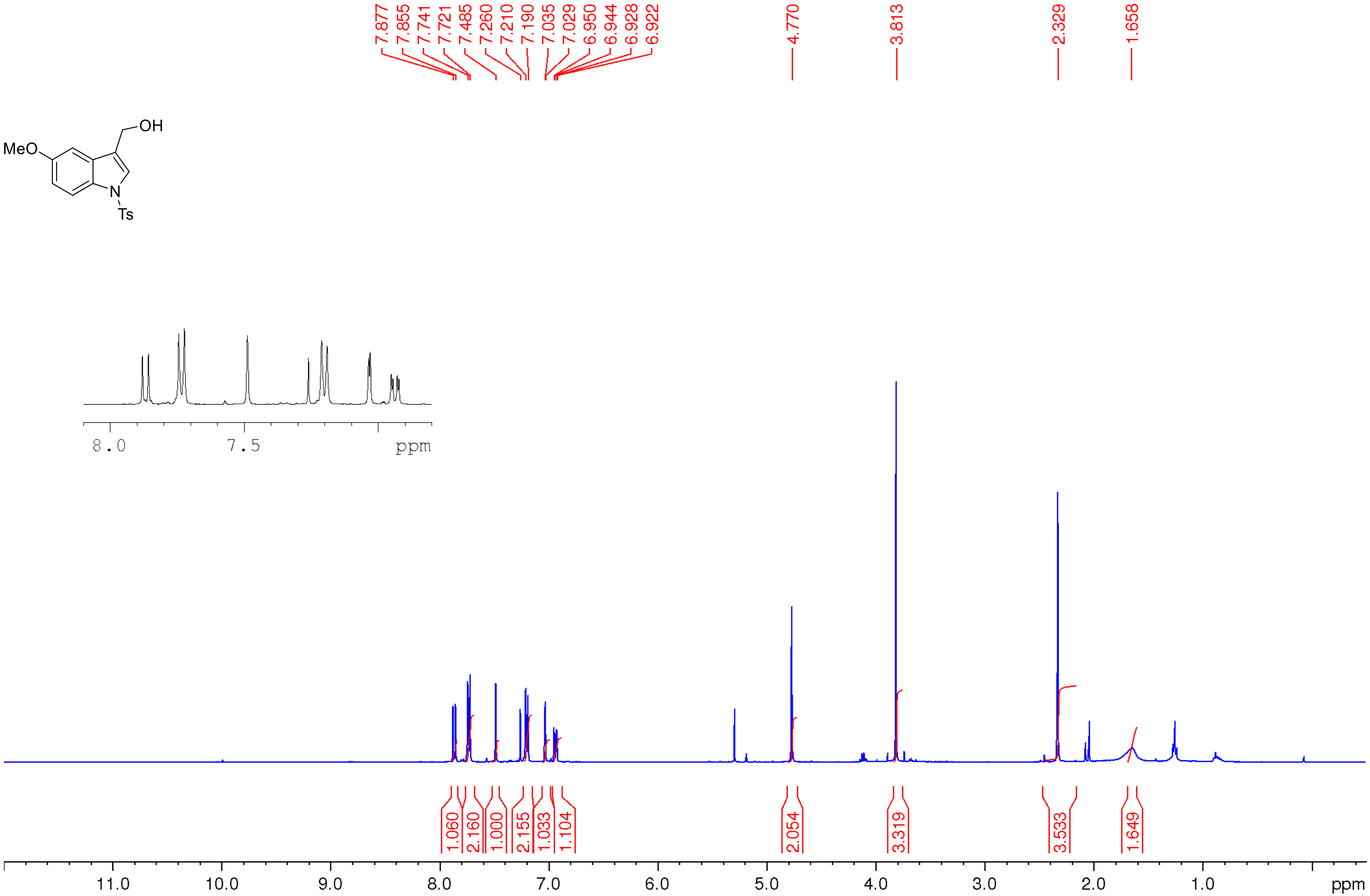

(5-methoxy-1-tosyl-1H-indol-3-yl)methanol

^{13}C NMR-spectrum (100.6 MHz) ($CDCl_3$)

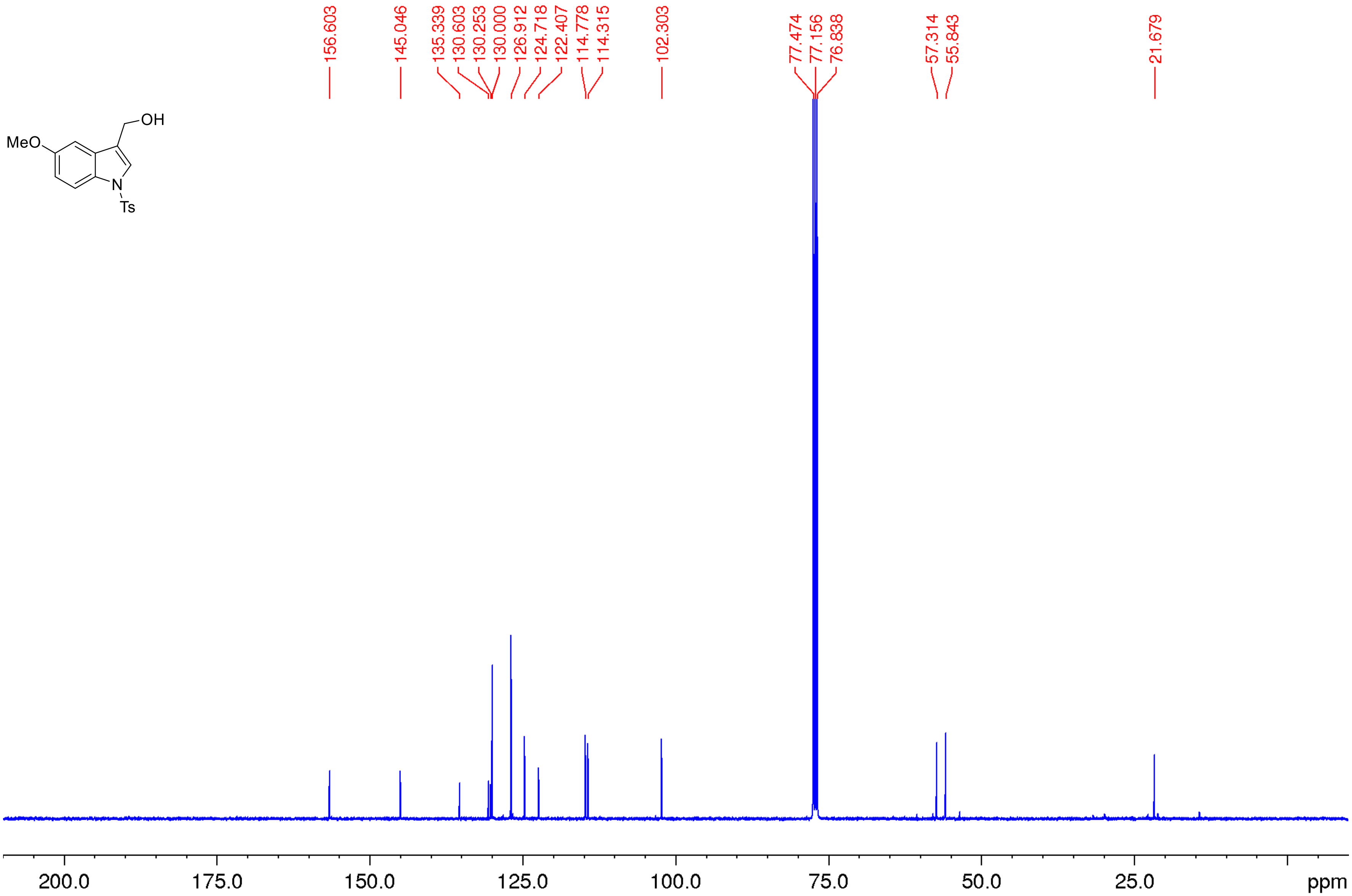

(5-methoxy-1-tosyl-1H-indol-3-yl)methanol

DEPT 135 NMR-spectrum ($CDCl_3$)

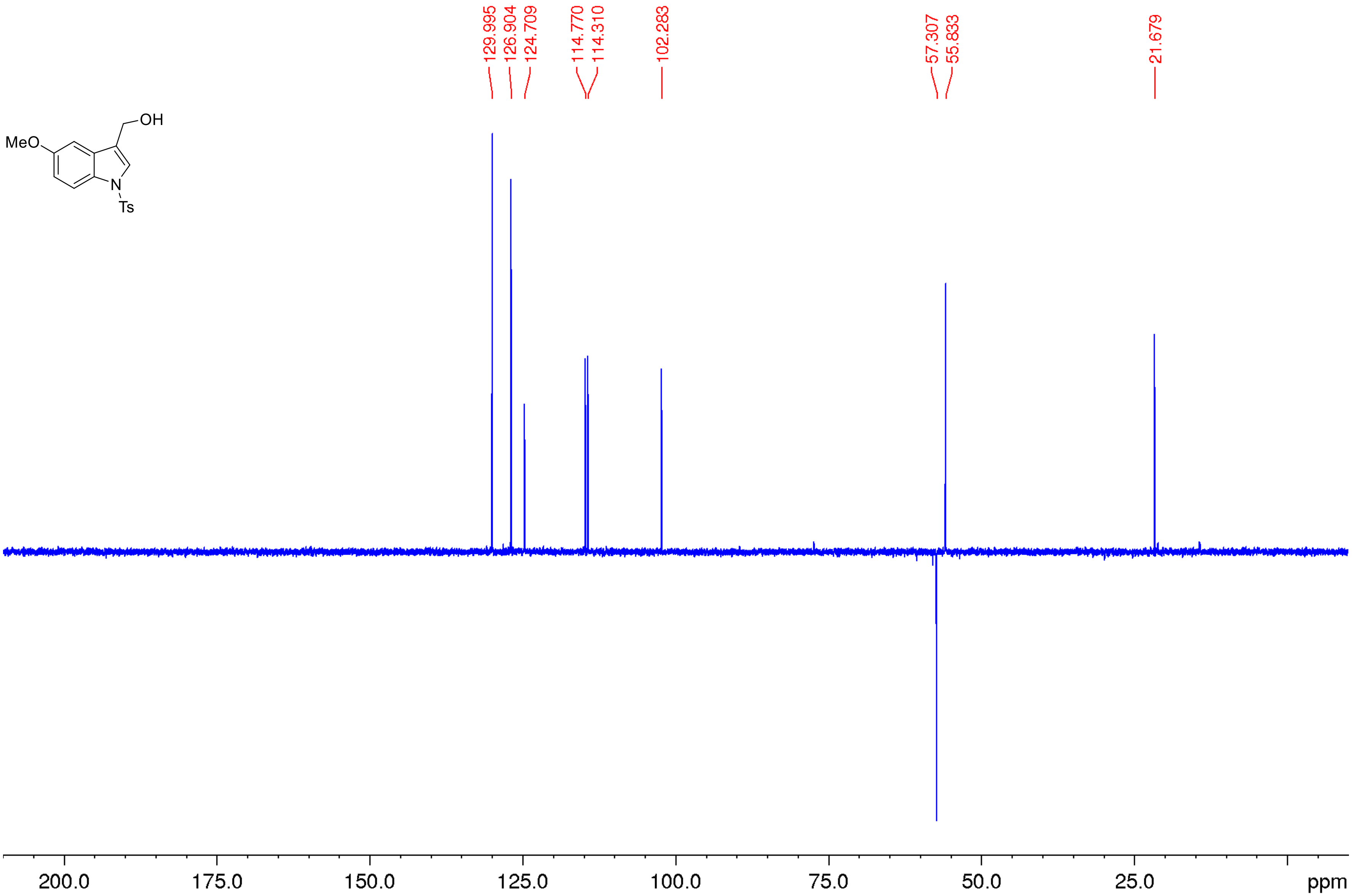

(5-phenyl-1-tosyl-1H-indol-3-yl)methanol

^{1}H NMR-spectrum (400.13 MHz) ($CDCl_3$)

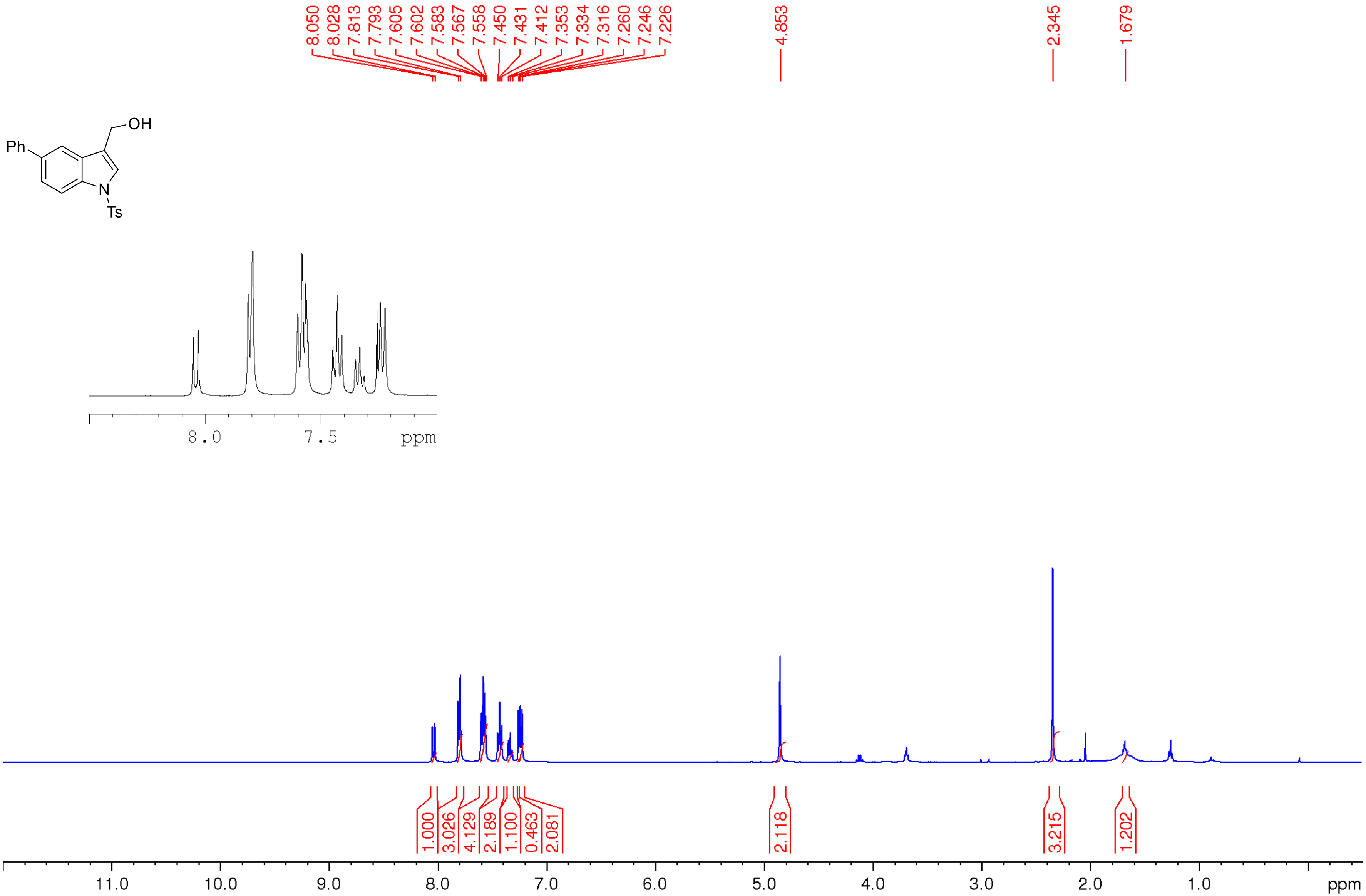

((5-phenyl-1-tosyl-1H-indol-3-yl)methanol

^{13}C NMR-spectrum (100.6 MHz) ($CDCl_3$)

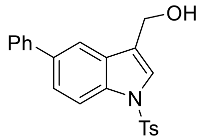

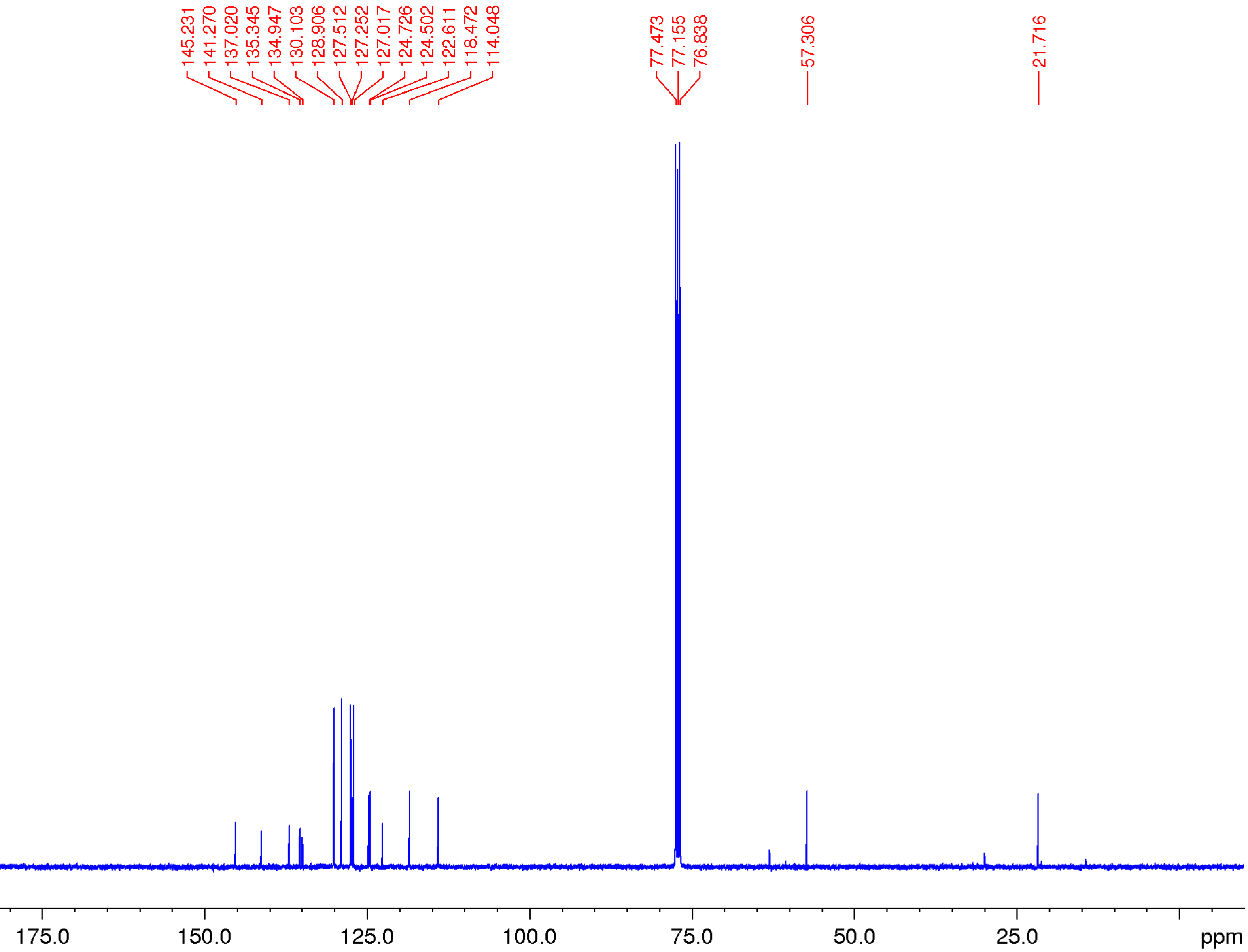

(5-phenyl-1-tosyl-1H-indol-3-yl)methanol

DEPT 135 NMR-spectrum ($CDCl_3$)

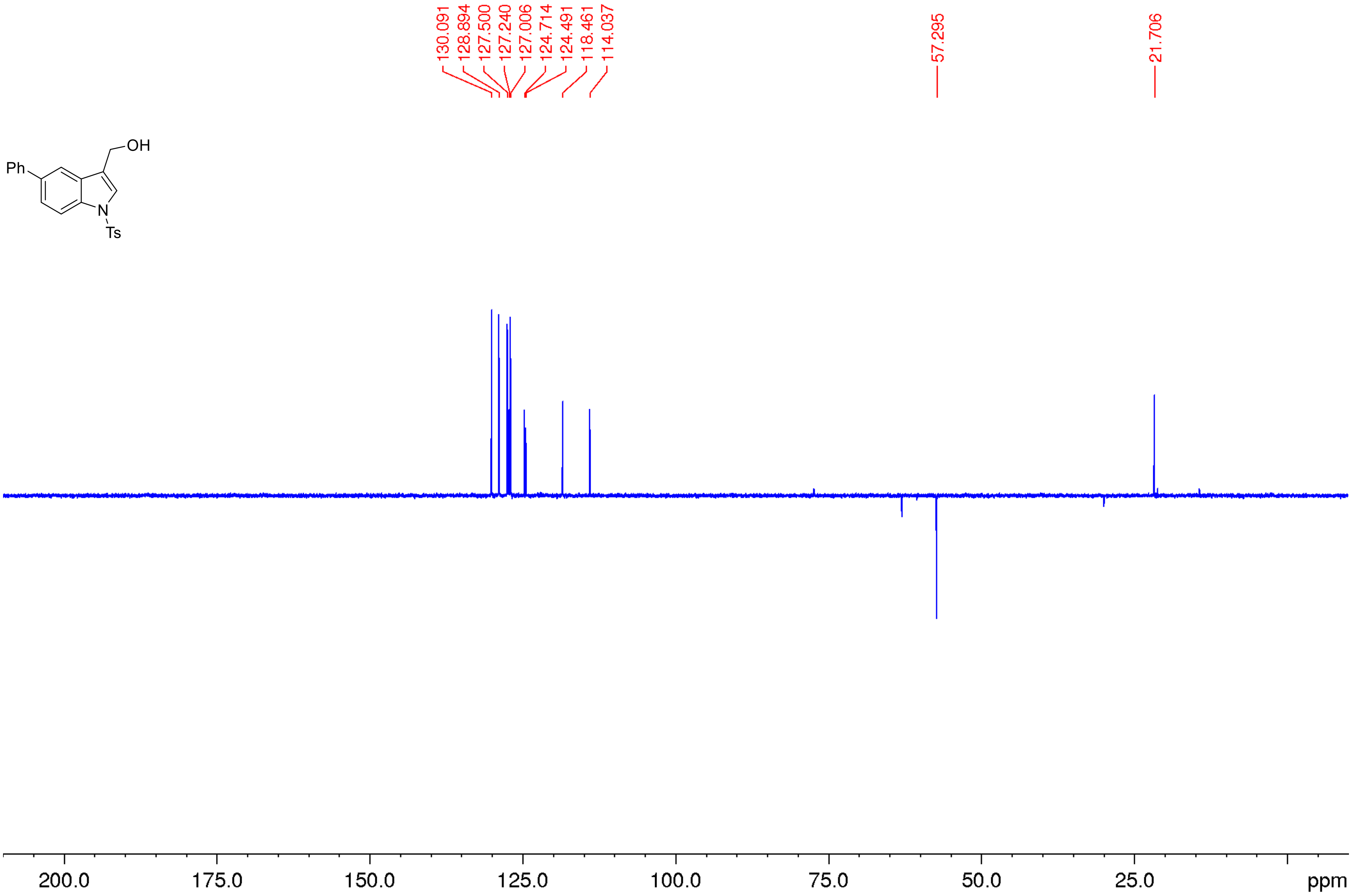

(6-chloro-1-tosyl-1H-indol-3-yl)methanol

^{1}H NMR-spectrum (400.13 MHz) ($CDCl_3$)

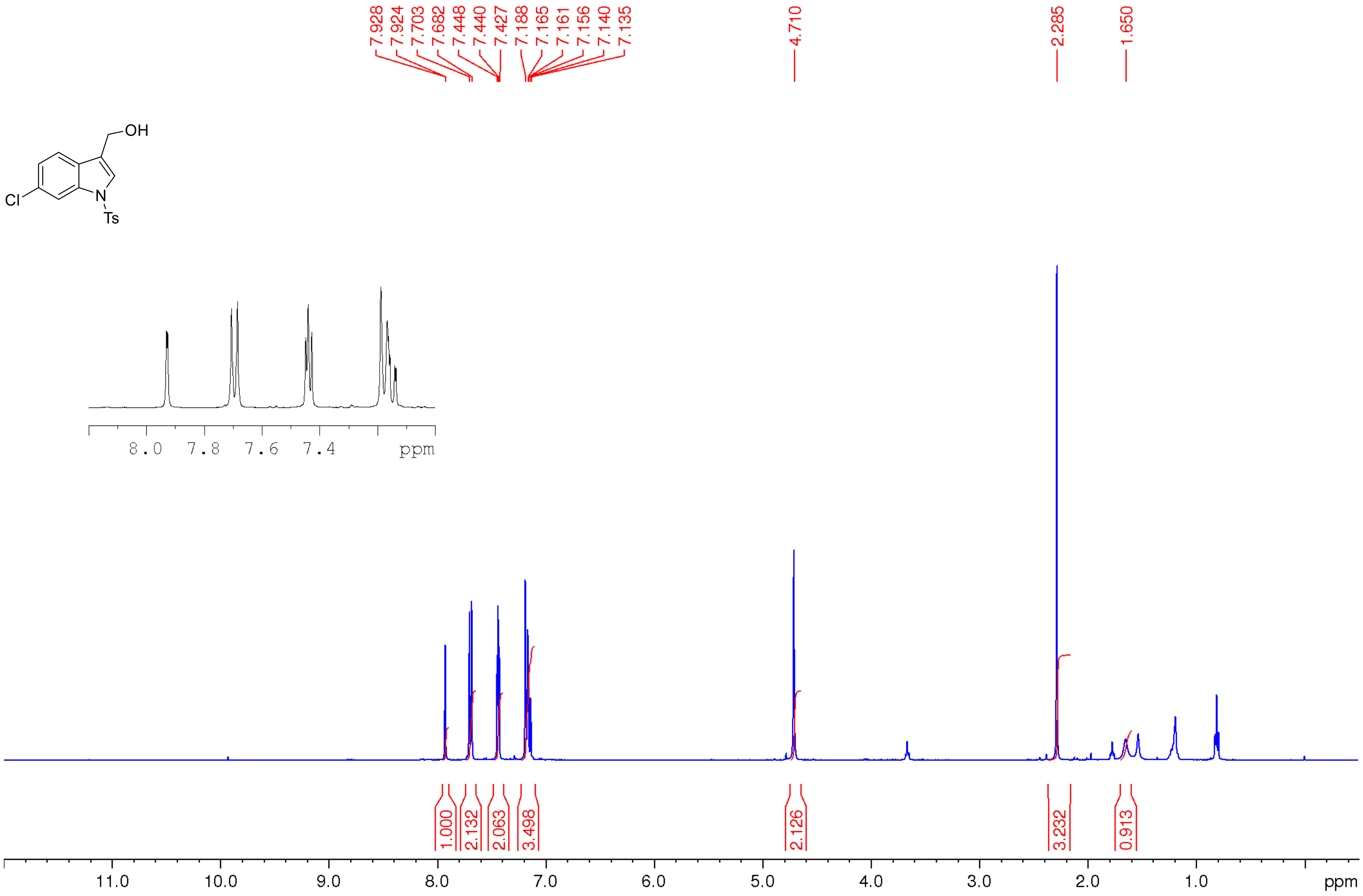

(6-chloro-1-tosyl-1H-indol-3-yl)methanol

^{13}C NMR-spectrum (100.6 MHz) ($CDCl_3$)

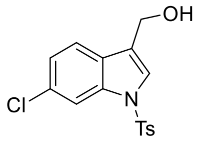

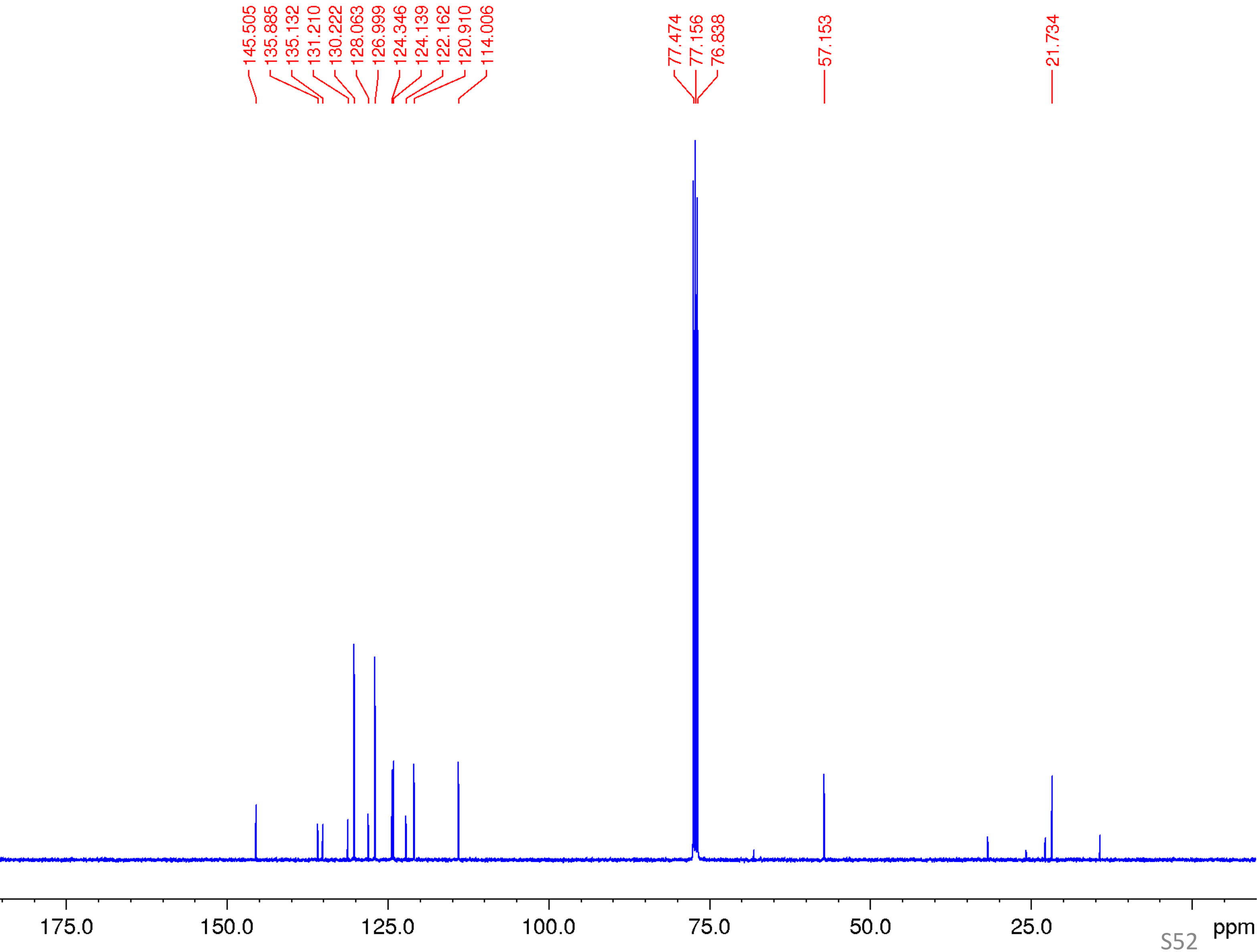

(6-chloro-1-tosyl-1H-indol-3-yl)methanol

DEPT 135 NMR-spectrum ($CDCl_3$)

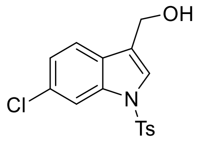

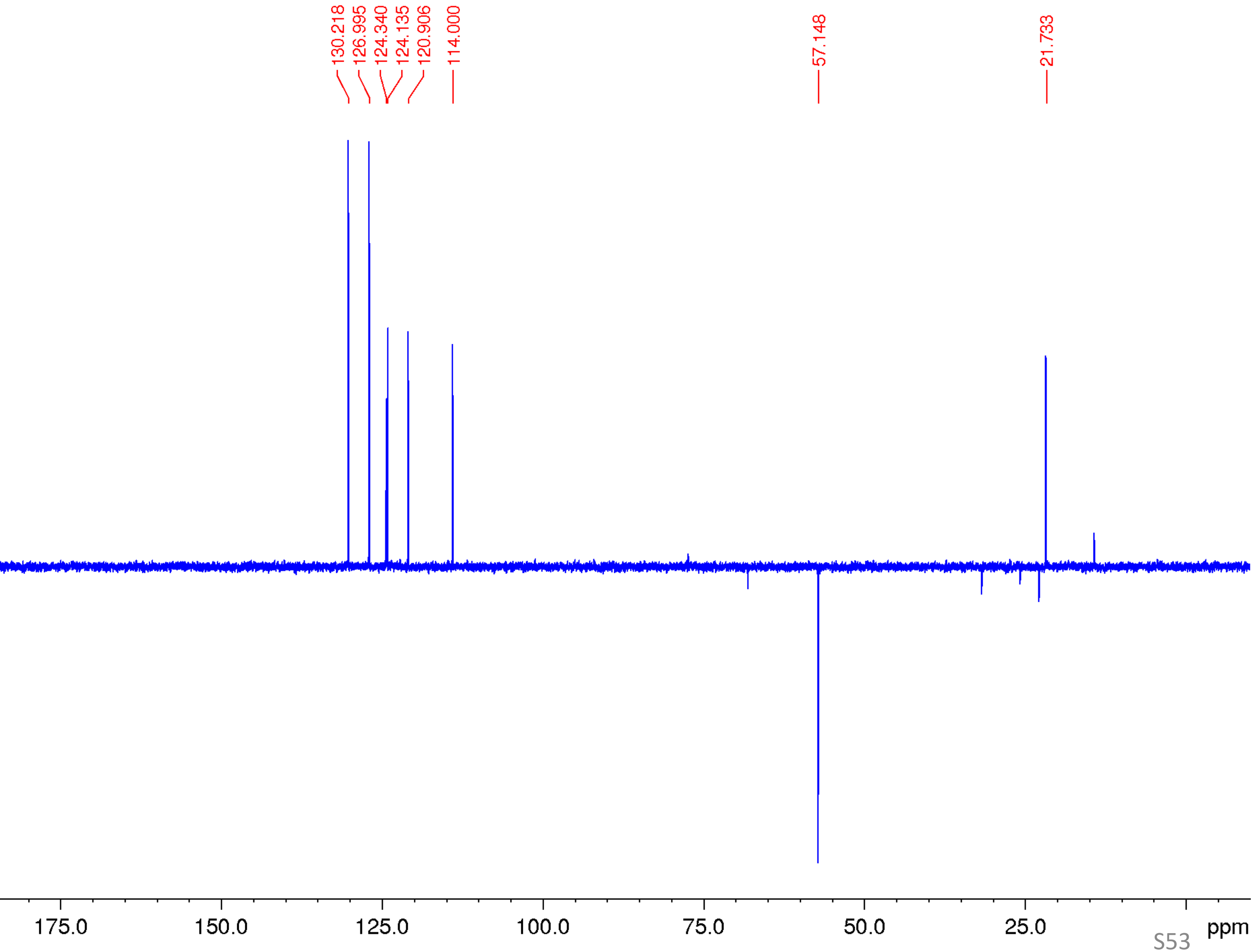

(5-nitro-1-tosyl-1H-indol-3-yl)methanol

^{1}H NMR-spectrum (400.13 MHz) (DMSO-d_6)

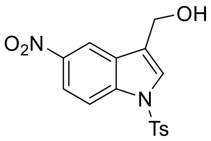

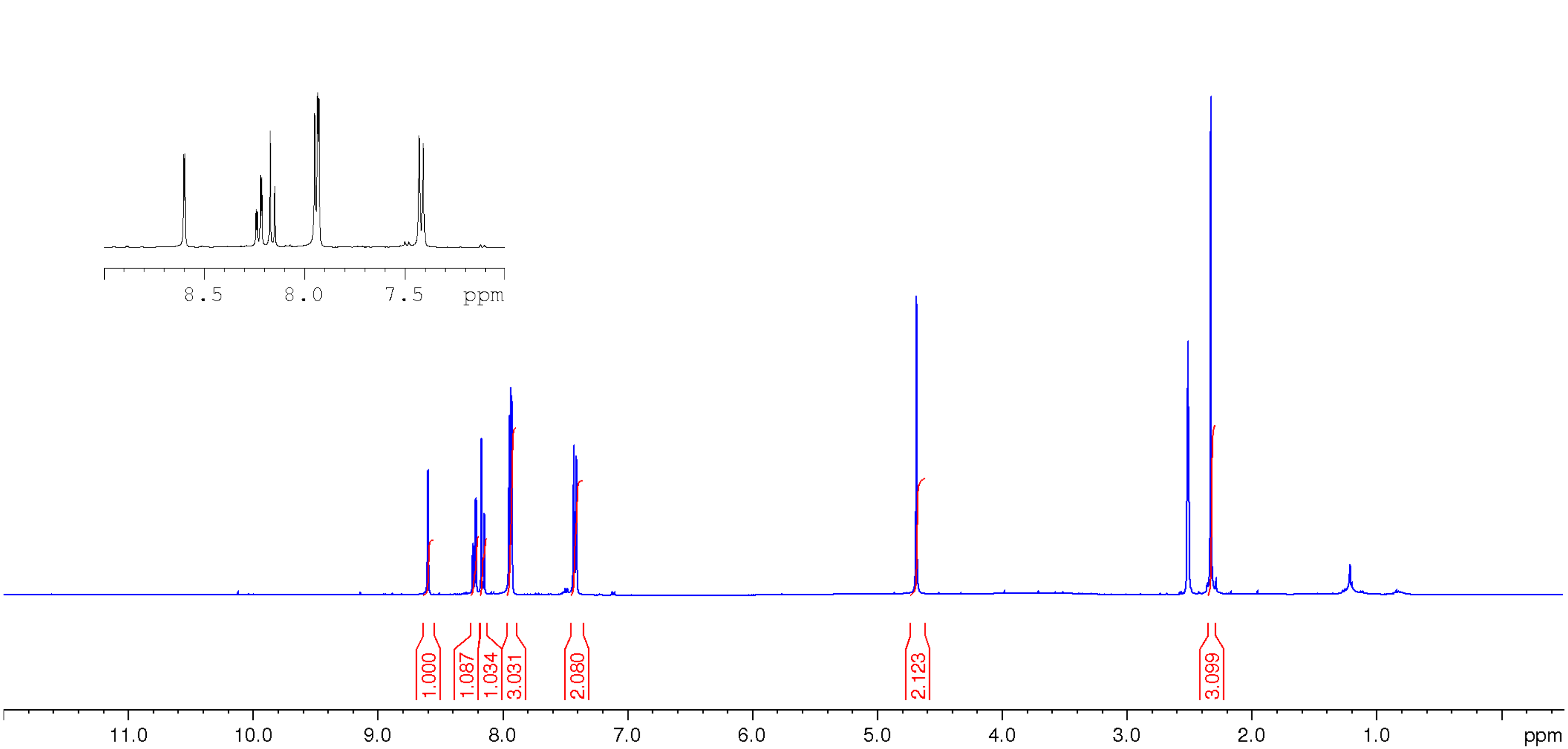

(5-nitro-1-tosyl-1H-indol-3-yl)methanol

^{13}C NMR-spectrum (100.6 MHz) (DMSO-d_6)

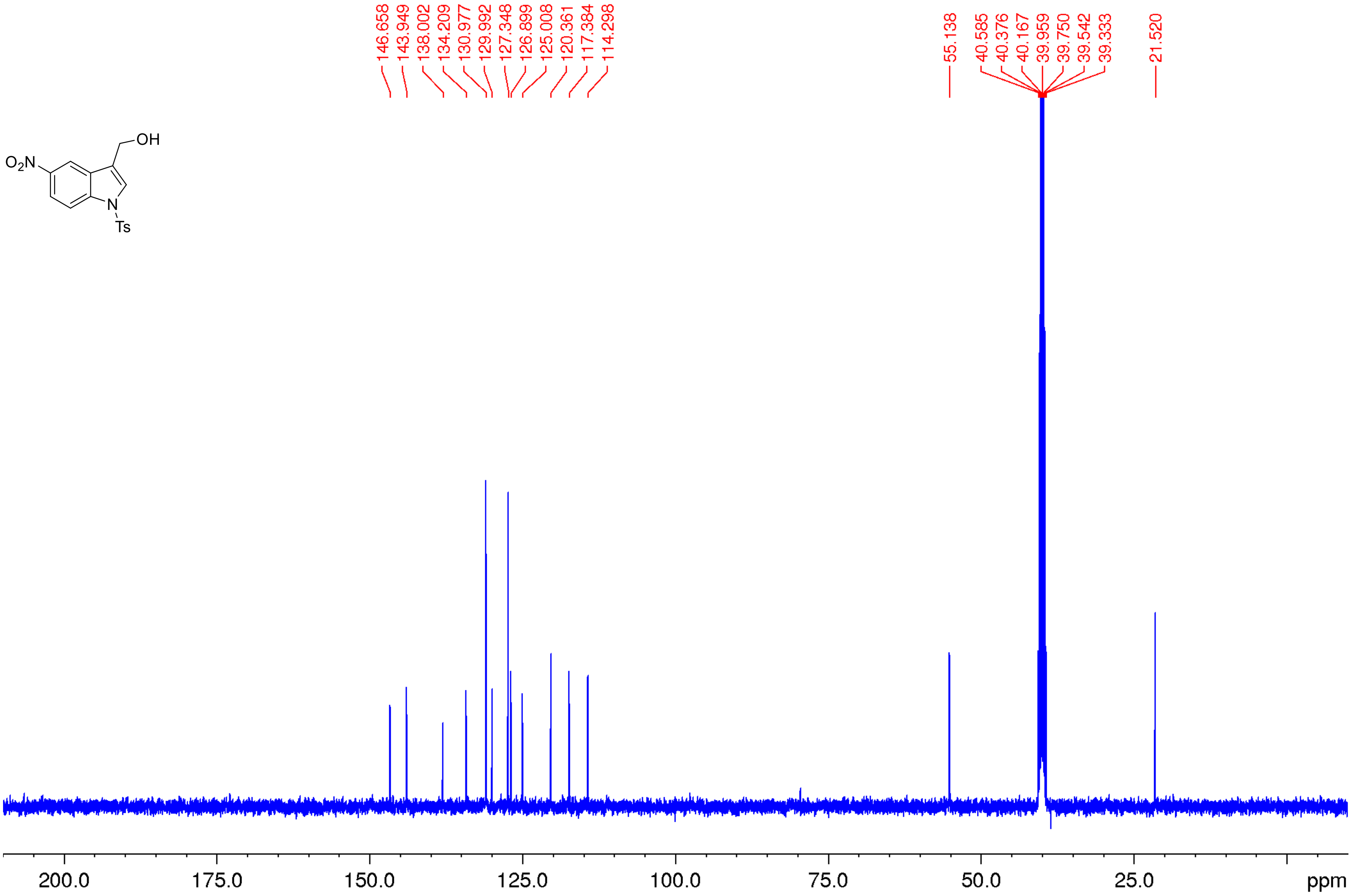

(5-nitro-1-tosyl-1H-indol-3-yl)methanol

DEPT 135 NMR-spectrum (DMSO-d_6)

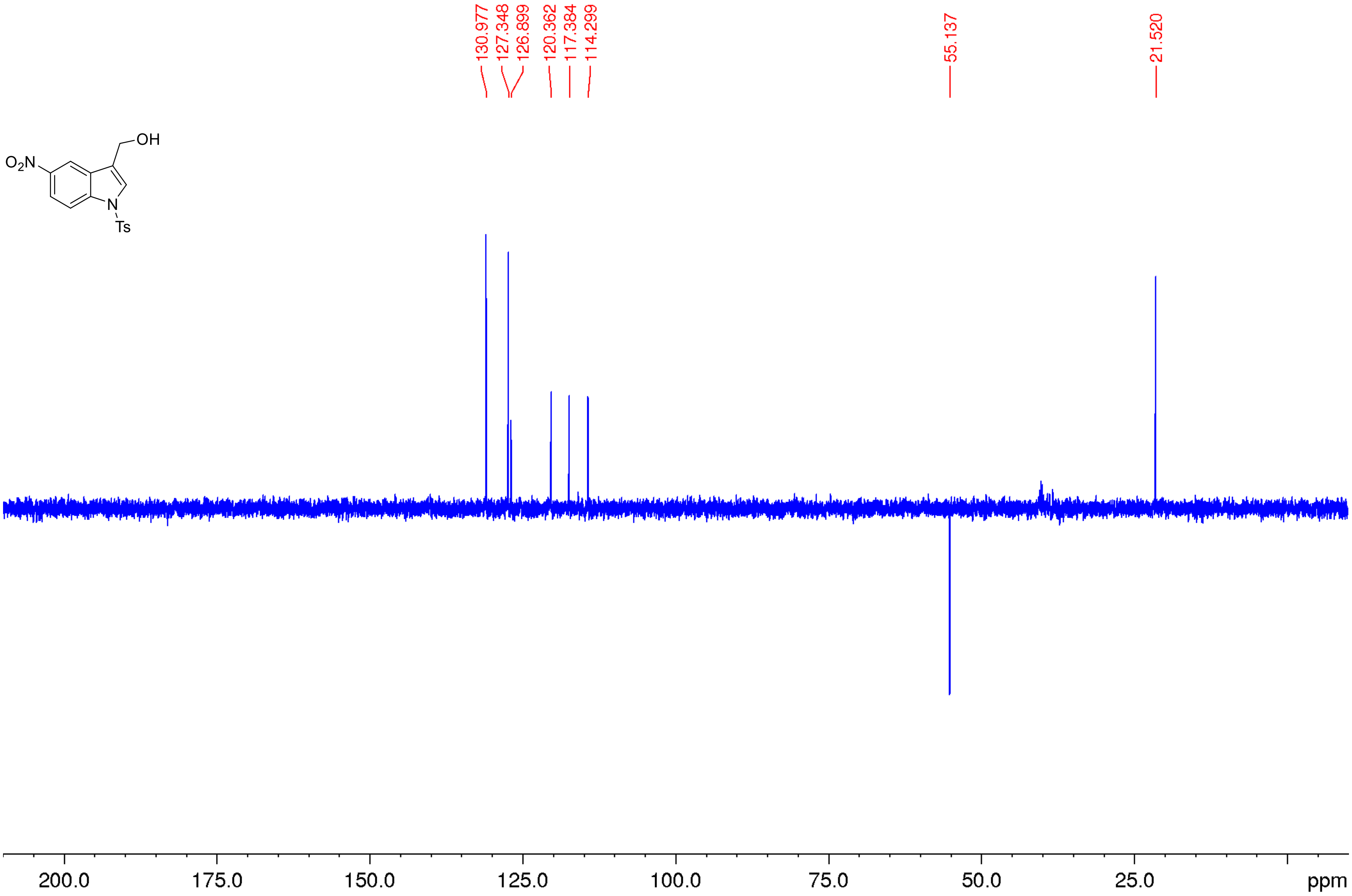

(1-tosyl-1H-indol-3-yl)methyl acetate **3a**

^{1}H NMR-spectrum (400.13 MHz) ($CDCl_3$)

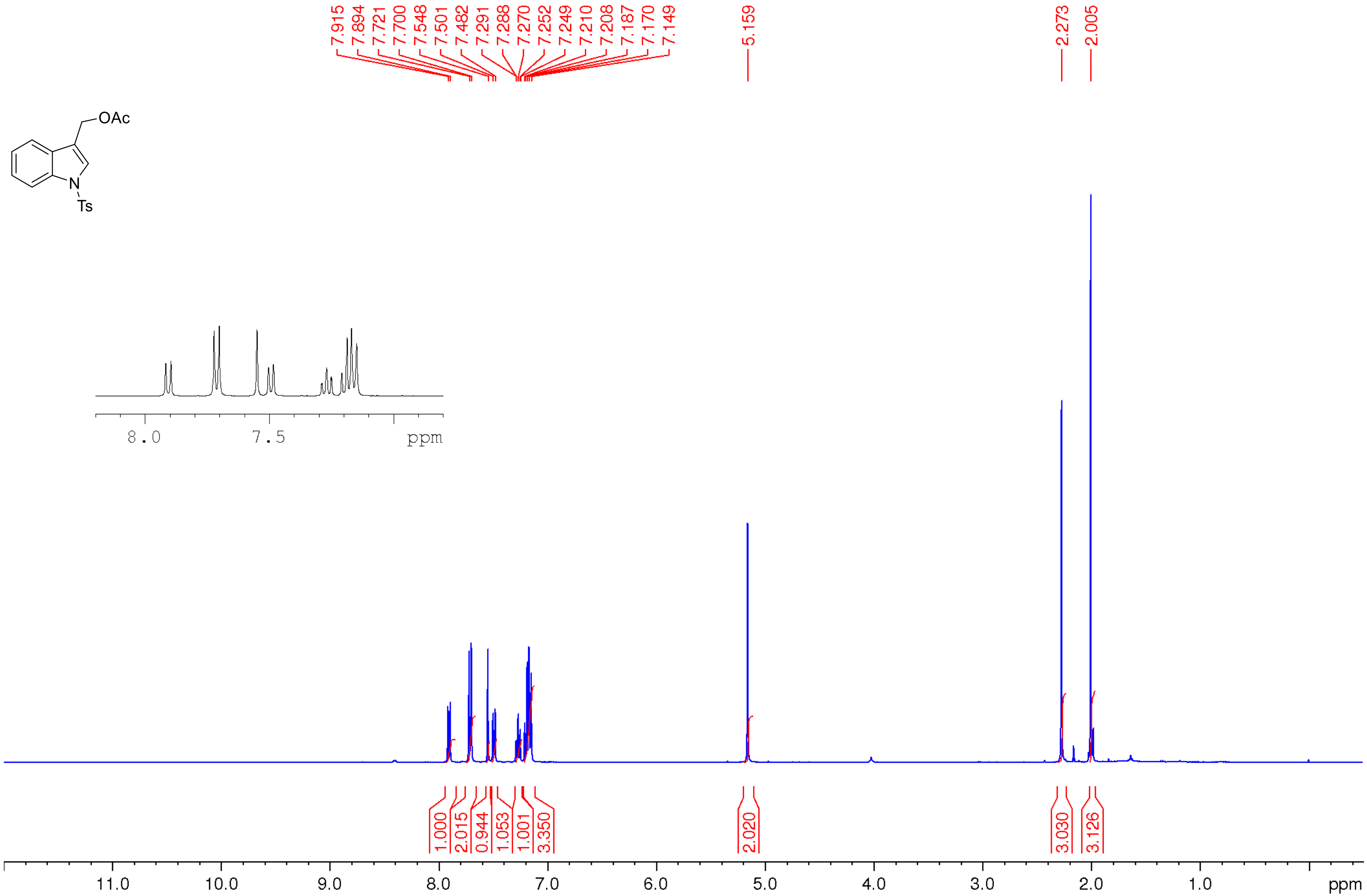

(*1-tosyl-1H-indol-3-yl)methyl acetate* **3a**

^{13}C NMR-spectrum (100.6 MHz) ($CDCl_3$)

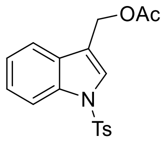

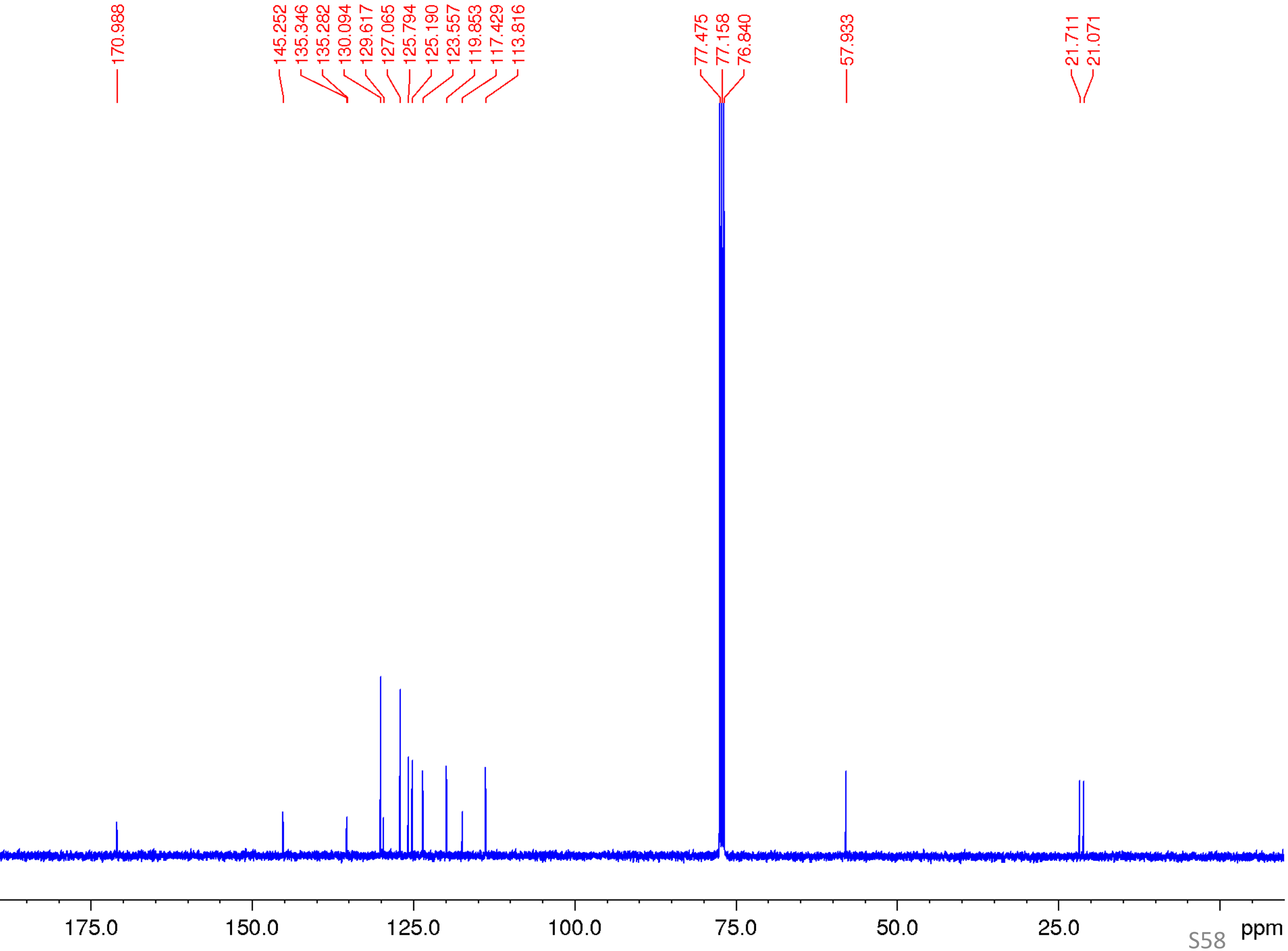

(1-tosyl-1H-indol-3-yl)methyl acetate **3a**

DEPT 135 NMR-spectrum ($CDCl_3$)

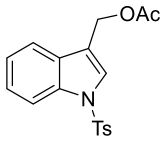

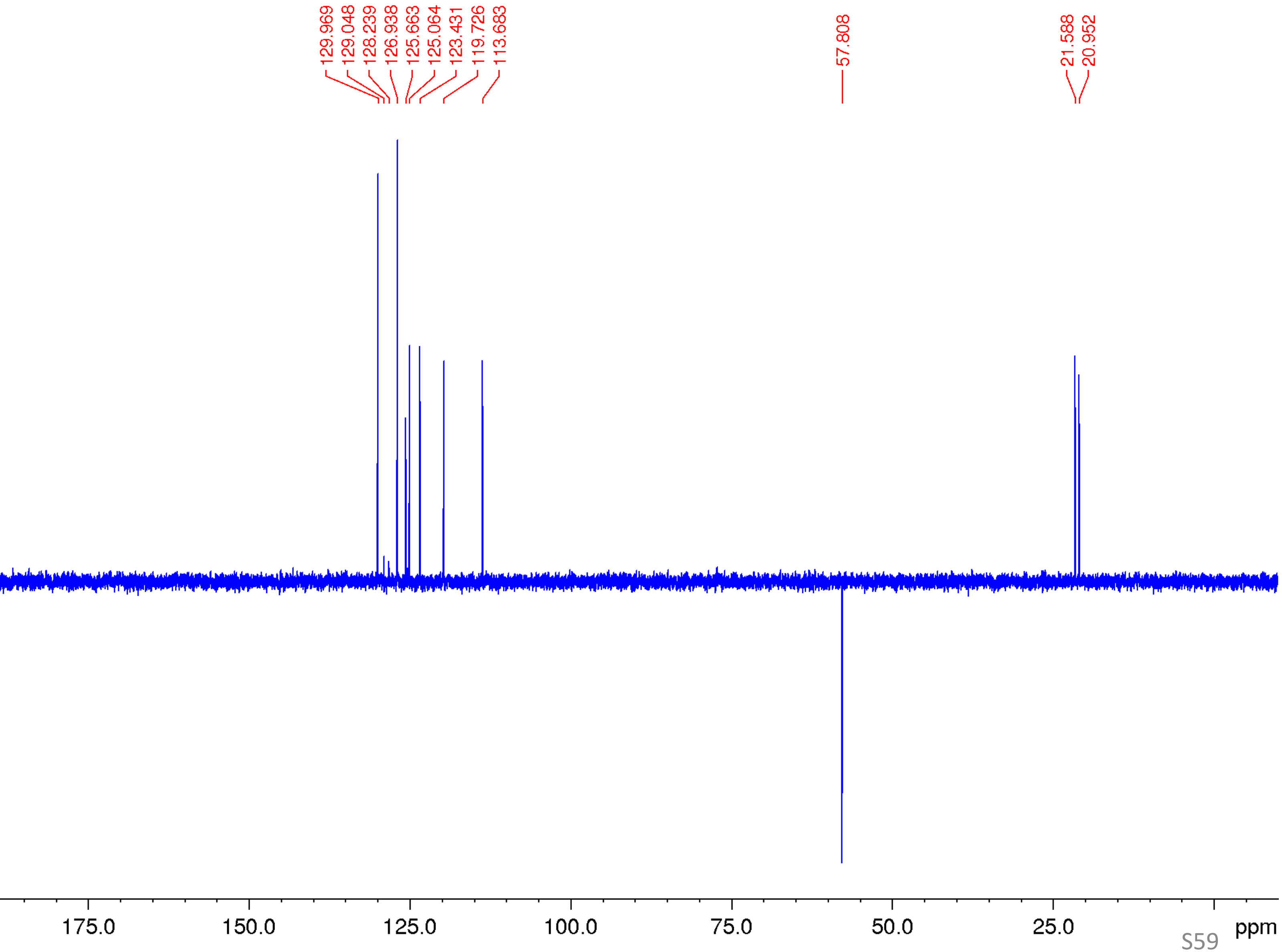

(1-benzyl-1H-indol-3-yl)methyl acetate 3b

^{1}H NMR-spectrum (400.13 MHz) ($CDCl_3$)

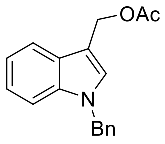

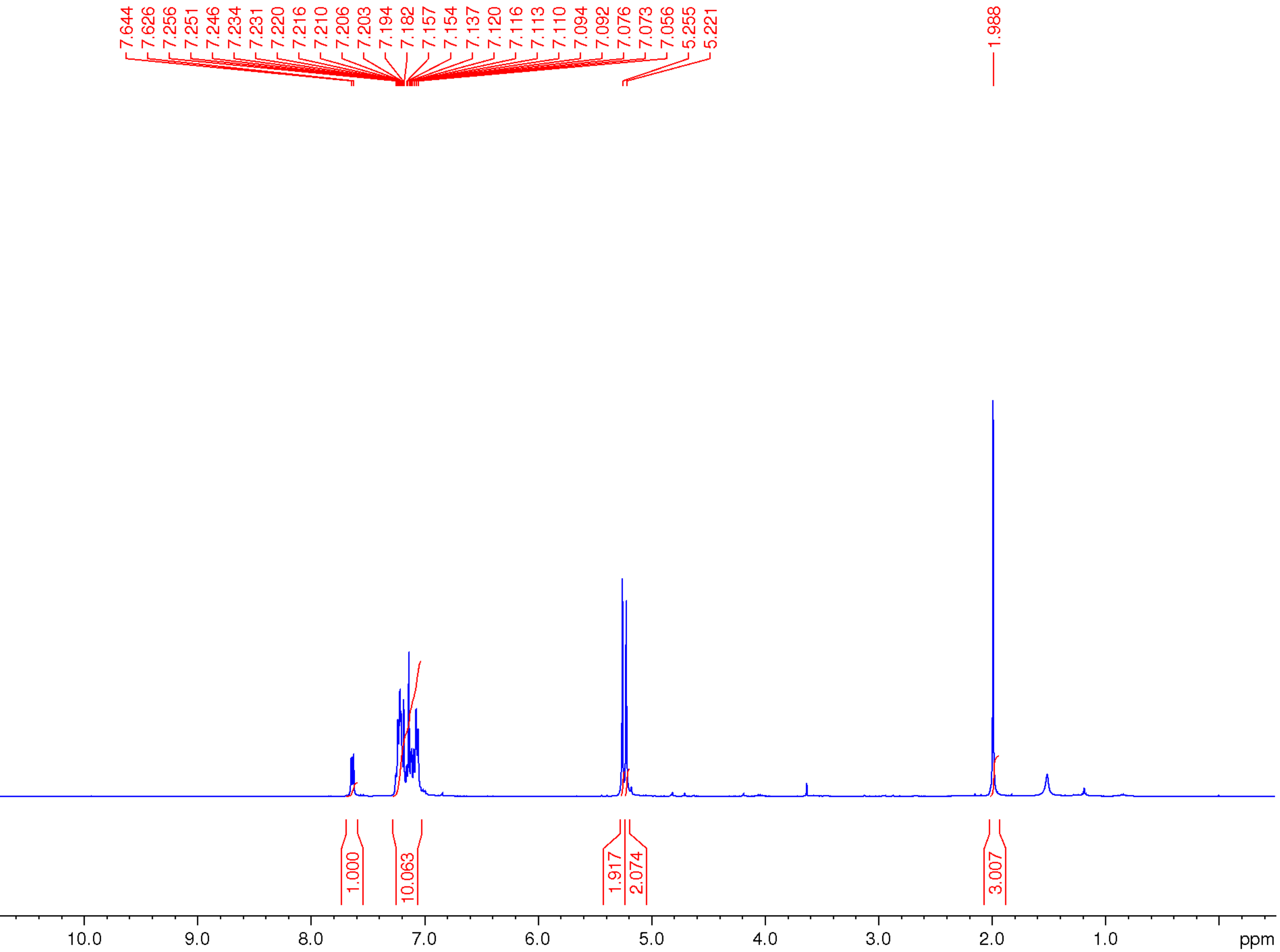

(1-benzyl-1H-indol-3-yl)methyl acetate 3b

^{13}C NMR-spectrum (100.6 MHz) ($CDCl_3$)

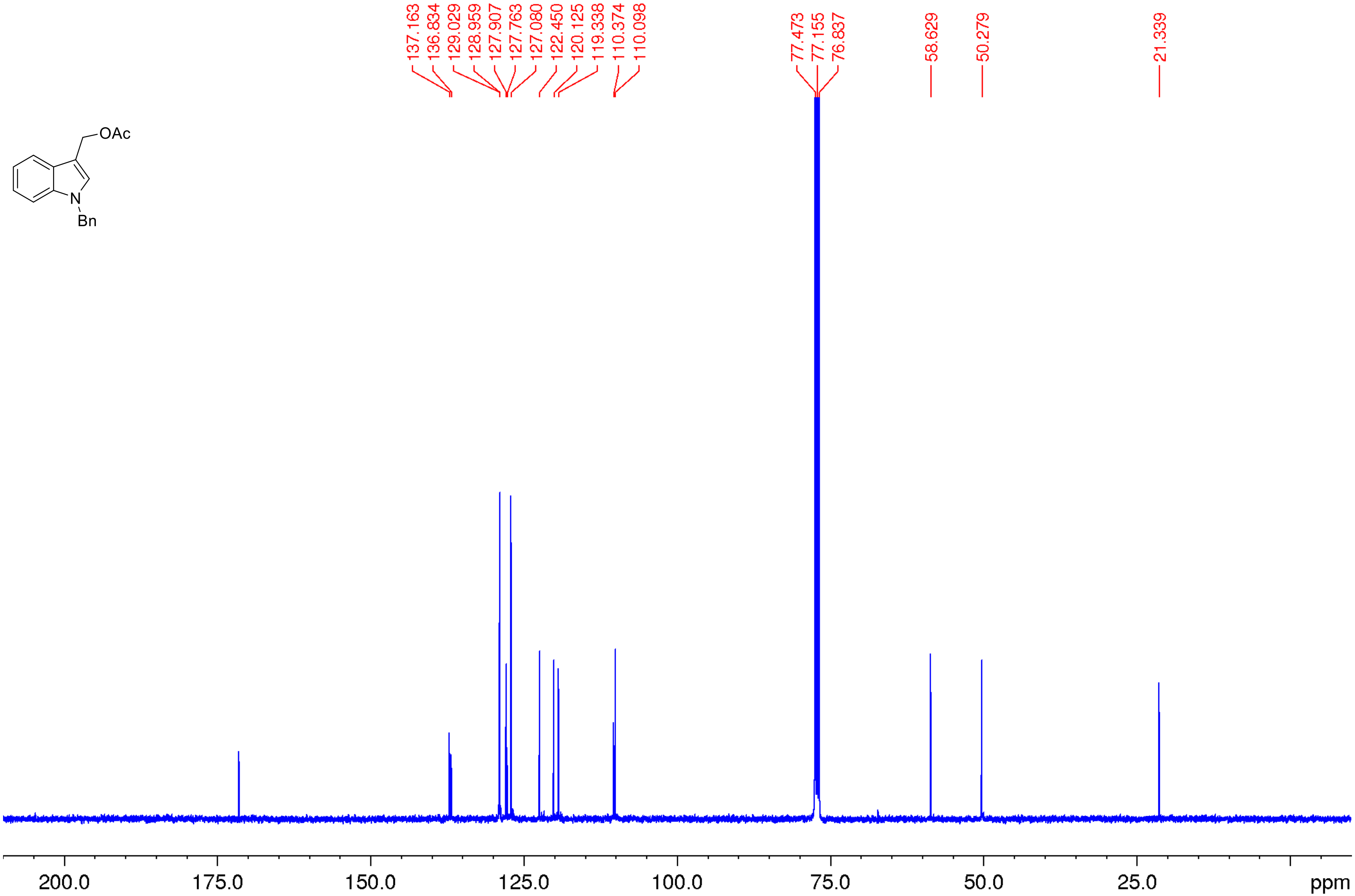

(1-benzyl-1H-indol-3-yl)methyl acetate 3b

DEPT 135 NMR-spectrum ($CDCl_3$)

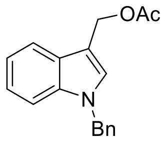

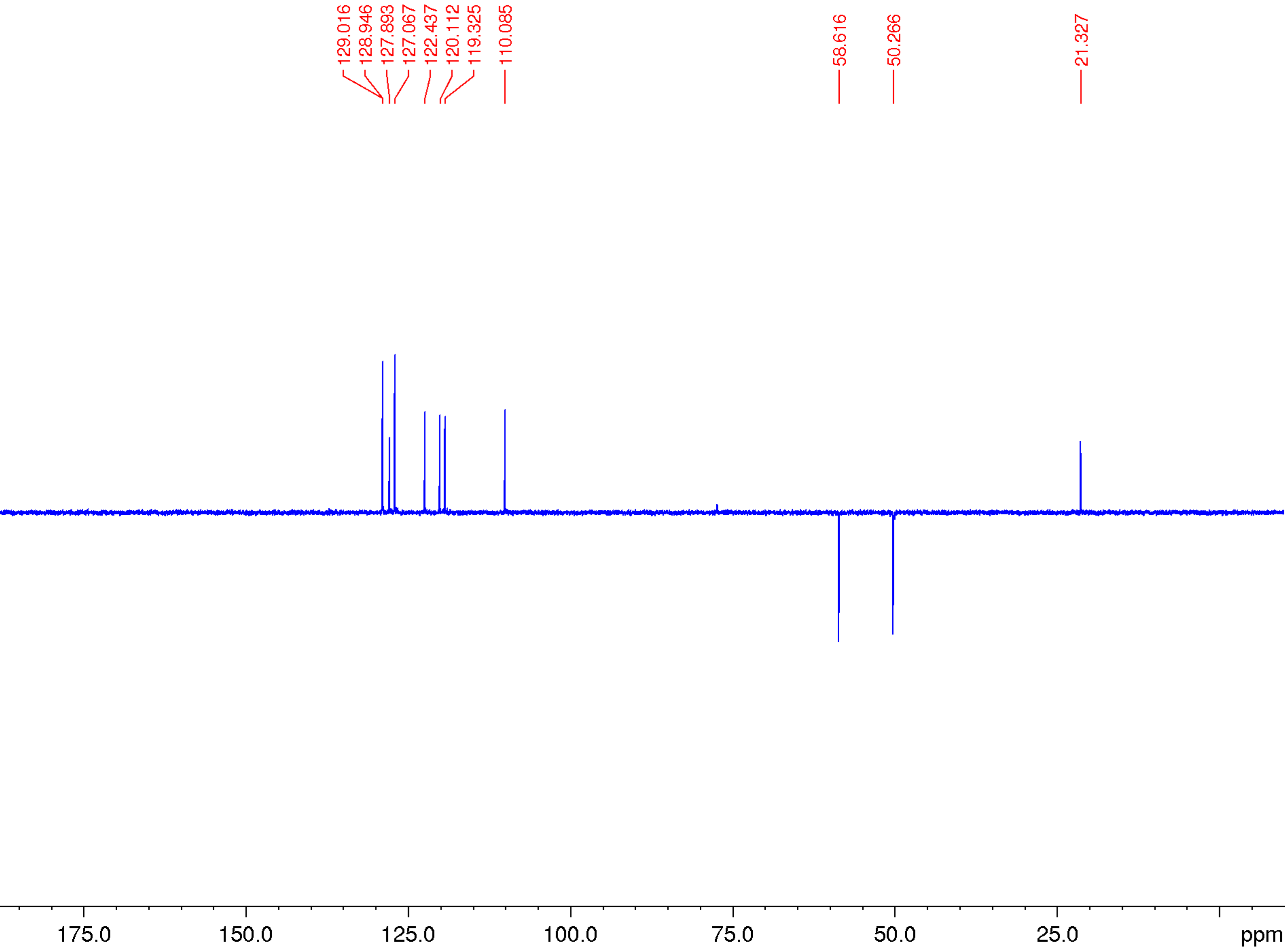

*(1-((2-(trimethylsilyl)ethoxy)methyl)-1H-indol-3-yl)methyl acetate **3c***

^{1}H NMR-spectrum (400.13 MHz) ($CDCl_3$)

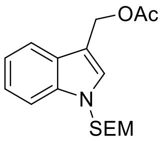

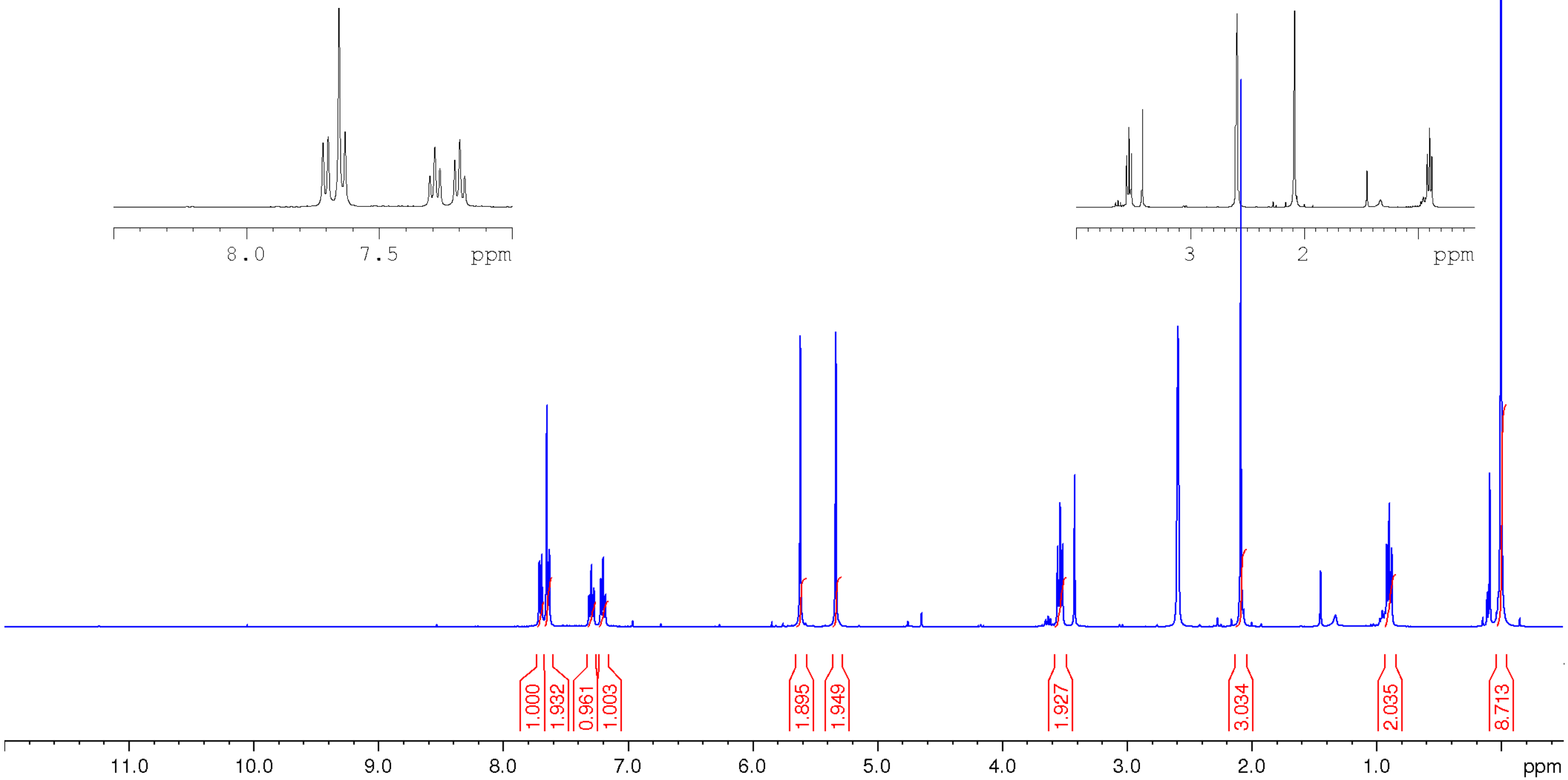

(1-((2-(trimethylsilyl)ethoxy)methyl)-1H-indol-3-yl)methyl acetate ***3c***

^{13}C NMR-spectrum (100.6 MHz) (DMSO-d_6)

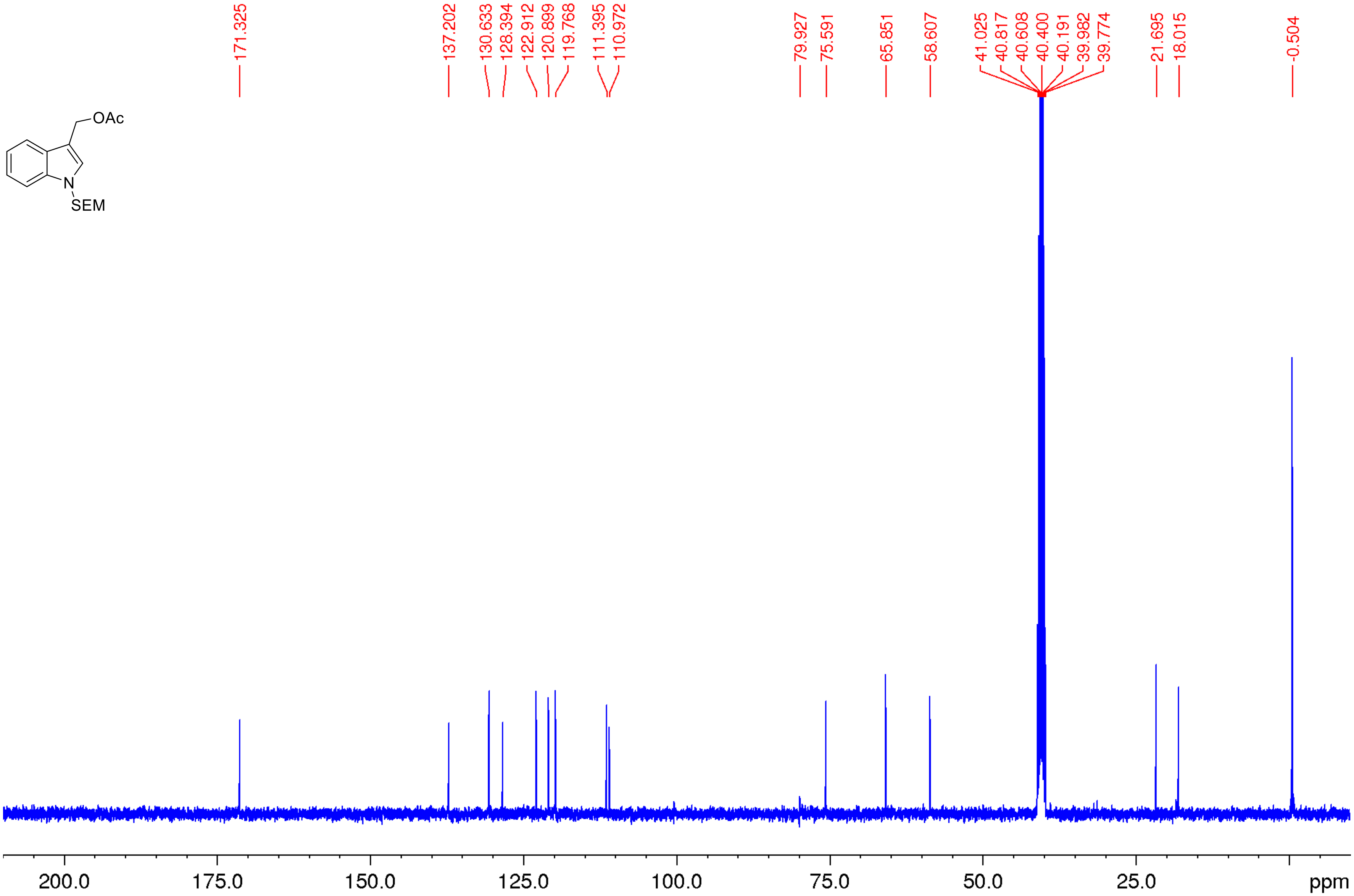

(1-((2-(trimethylsilyl)ethoxy)methyl)-1H-indol-3-yl)methyl acetate ***3c***

DEPT 135 NMR-spectrum (DMSO-d_6)

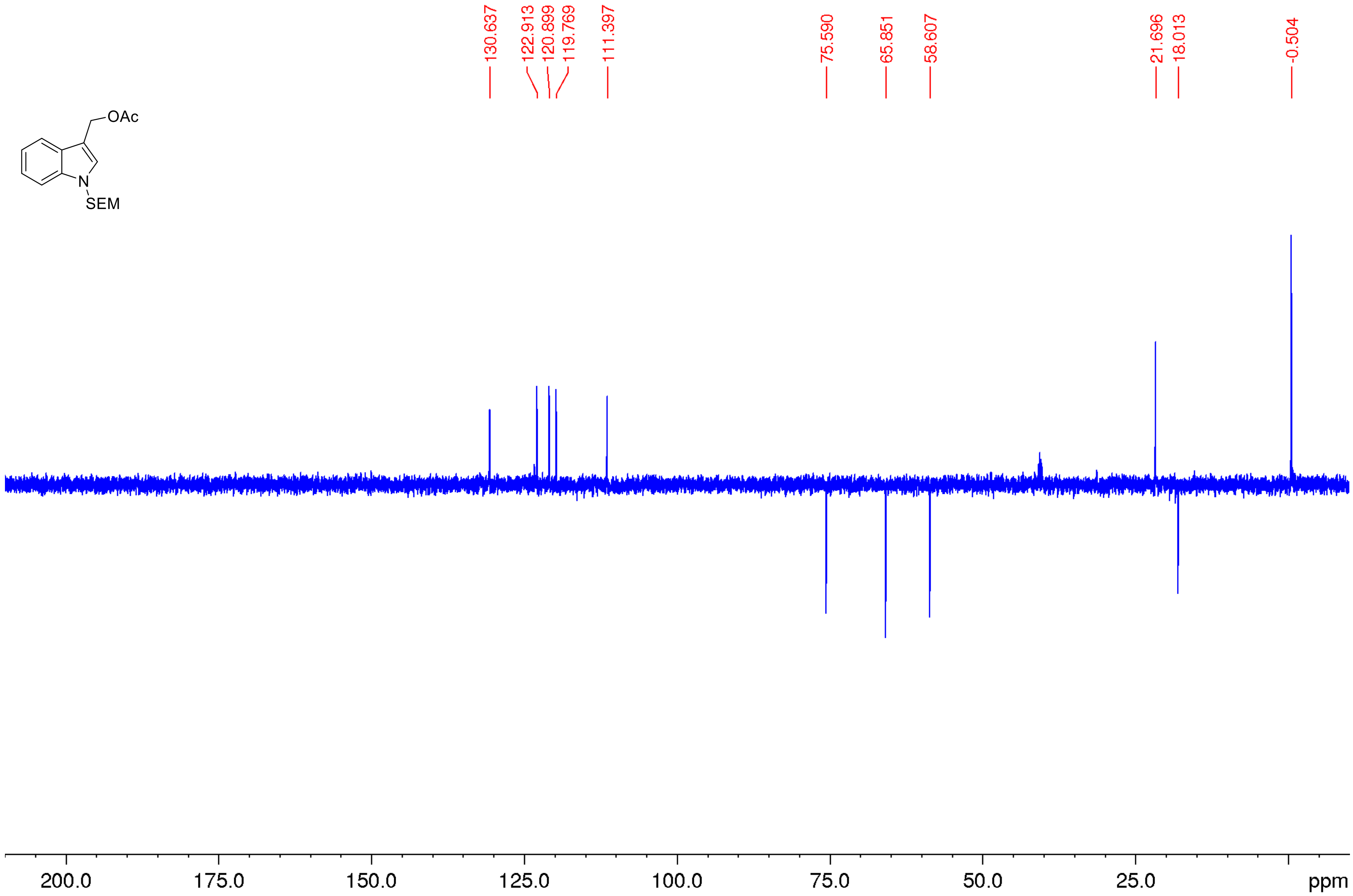

(5-methoxy-1-tosyl-1H-indol-3-yl)methyl acetate ***3d***

^{1}H NMR-spectrum (400.13 MHz) (DMSO-d_6)

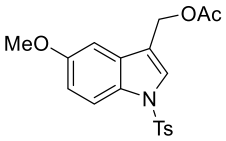

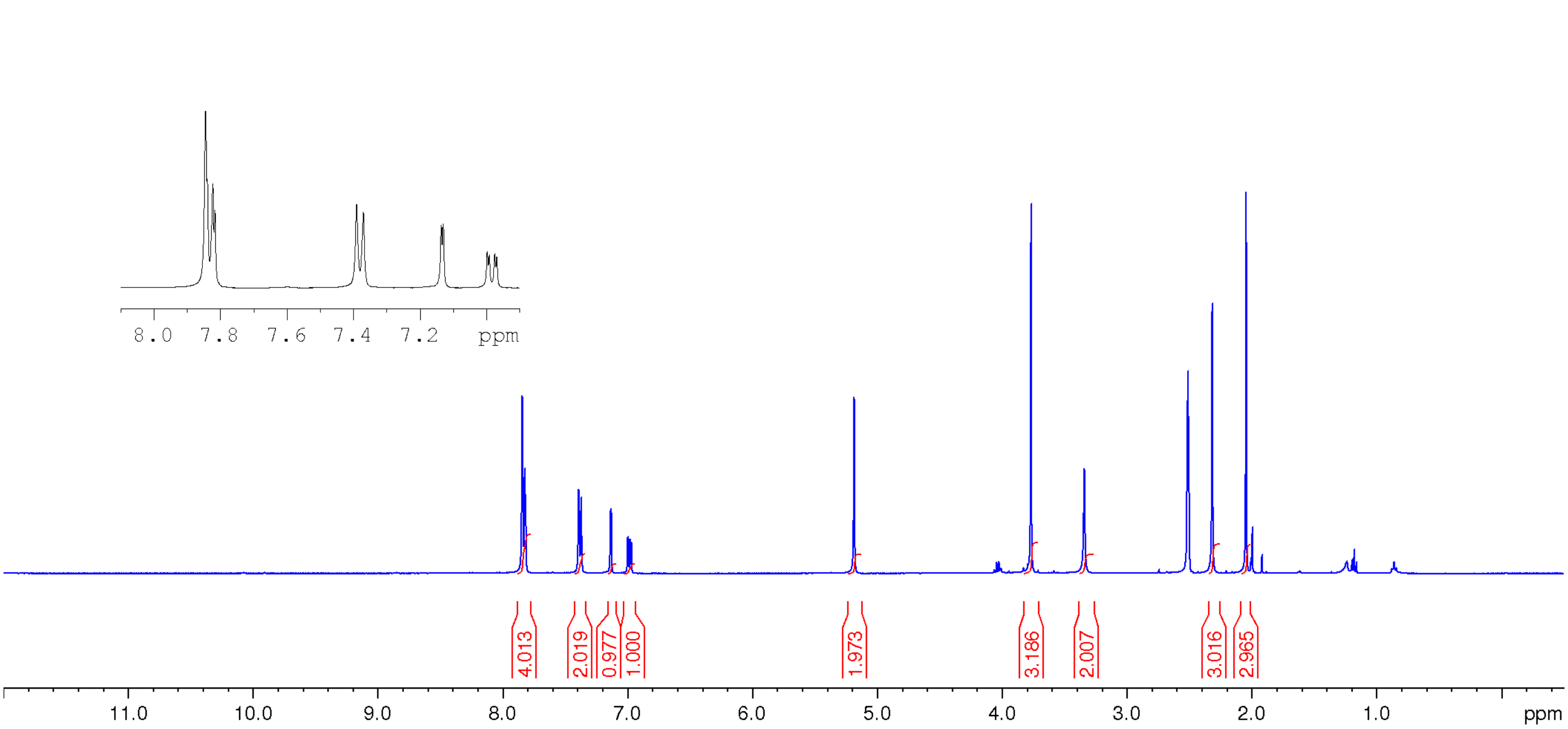

*(5-methoxy-1-tosyl-1H-indol-3-yl)methyl acetate **3d***

^{13}C NMR-spectrum (100.6 MHz) (DMSO-d_6)

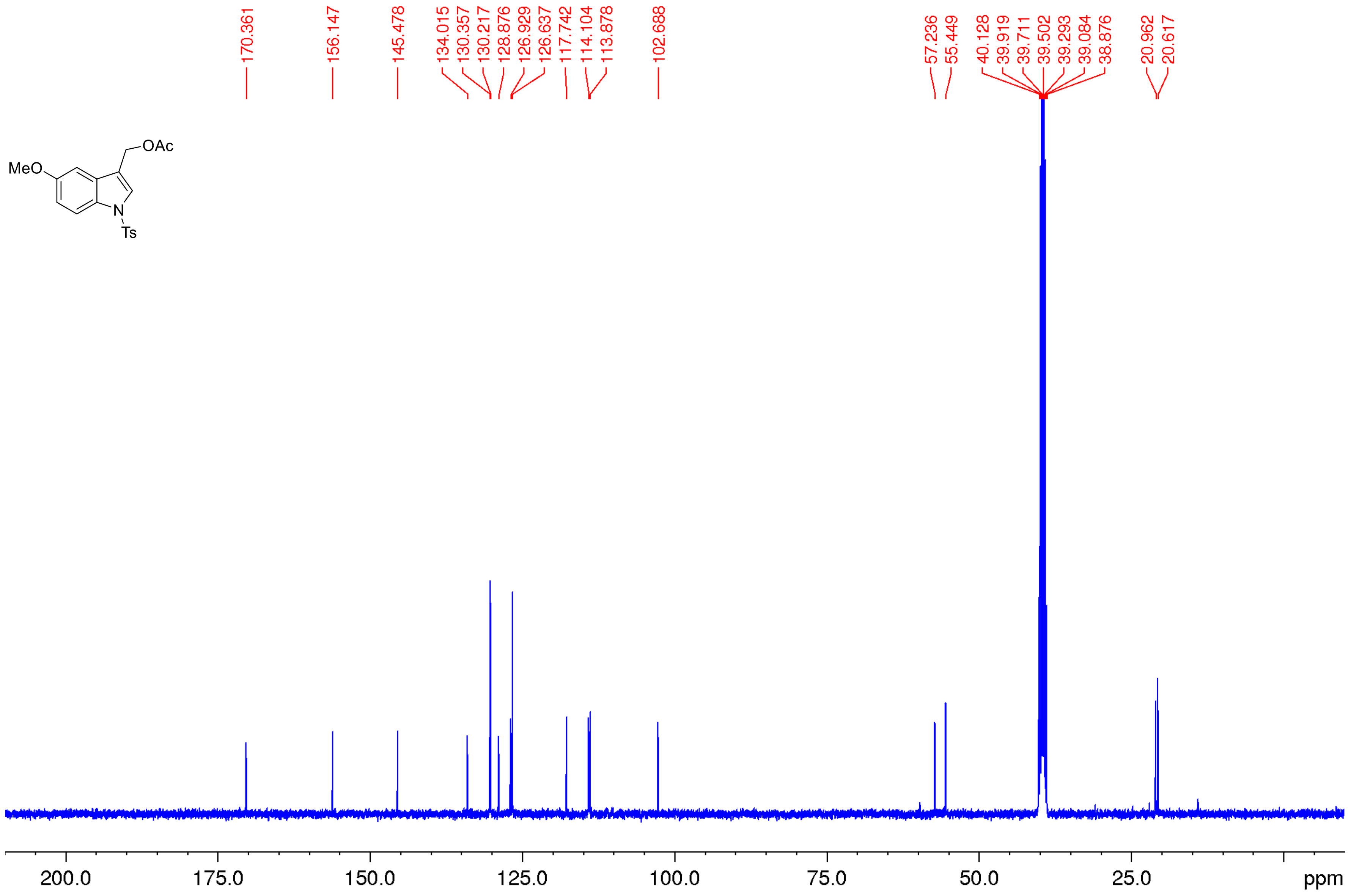

(5-methoxy-1-tosyl-1H-indol-3-yl)methyl acetate ***3d***

DEPT 135 NMR-spectrum (DMSO-d_6)

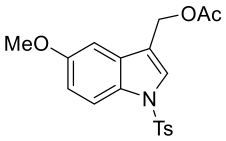

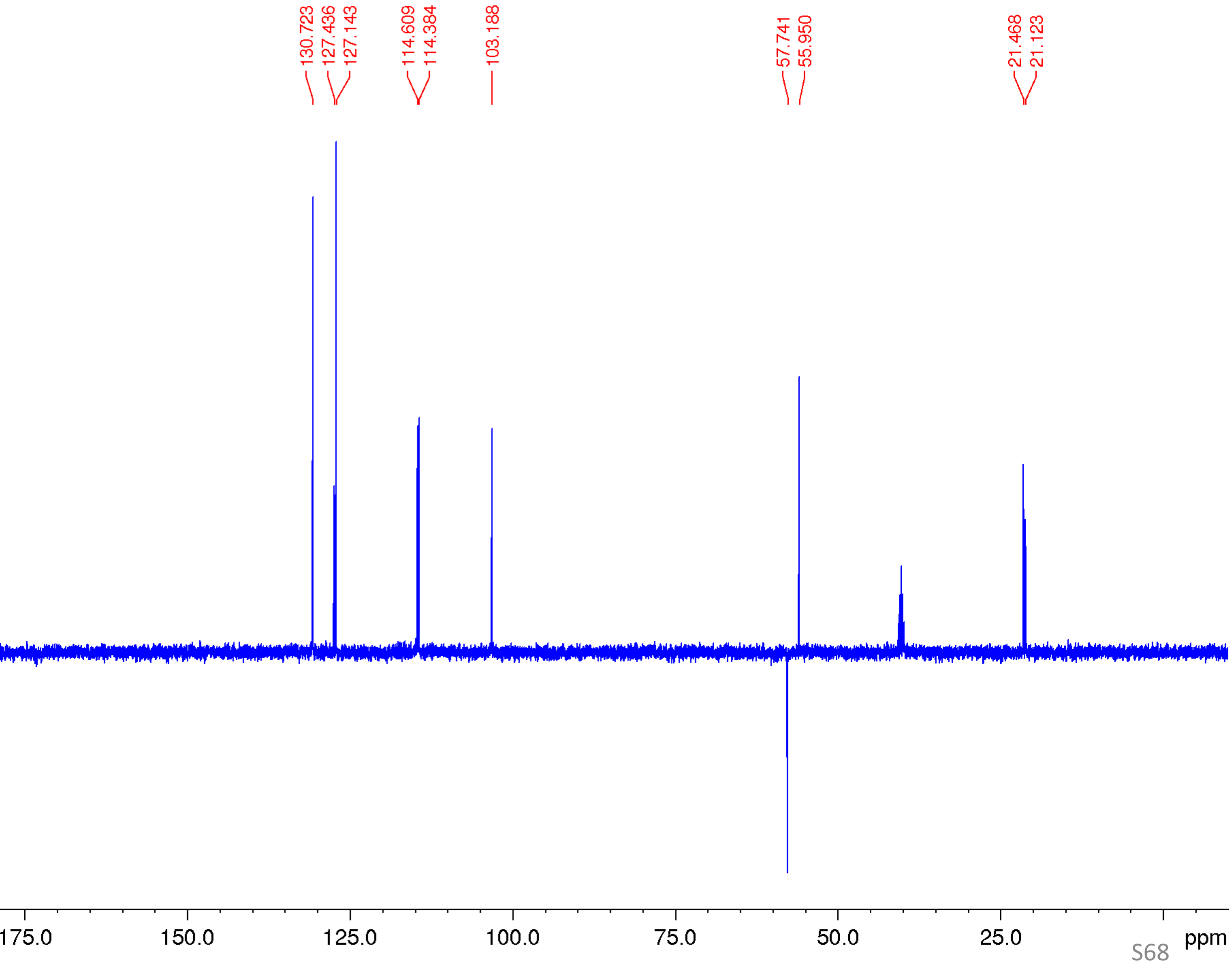

(5-phenyl-1-tosyl-1H-indol-3-yl)methyl acetate ***3e***

^{1}H NMR-spectrum (400.13 MHz) ($CDCl_3$)

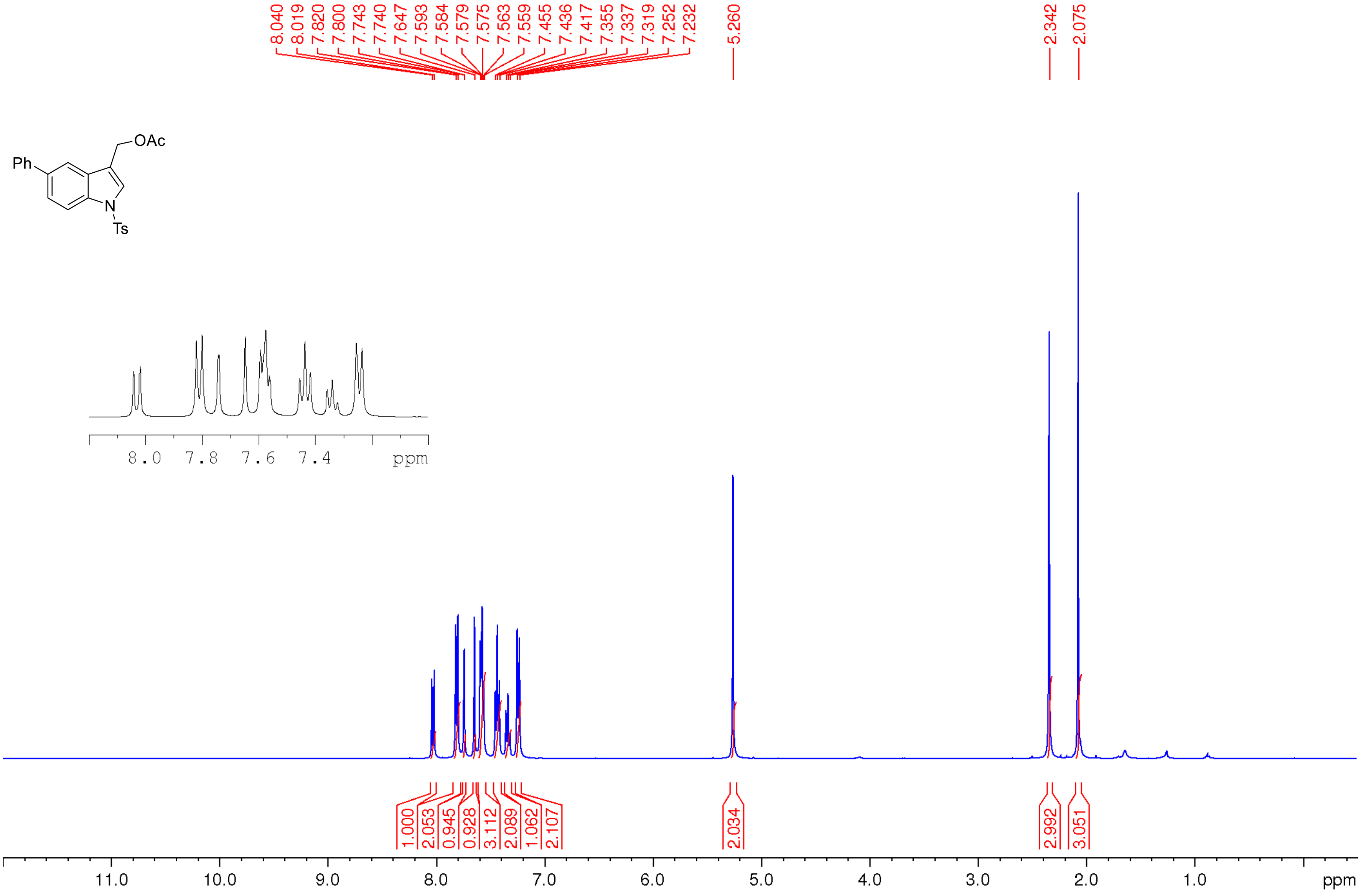

(5-phenyl-1-tosyl-1H-indol-3-yl)methyl acetate ***3e***

^{13}C NMR-spectrum (100.6 MHz) ($CDCl_3$)

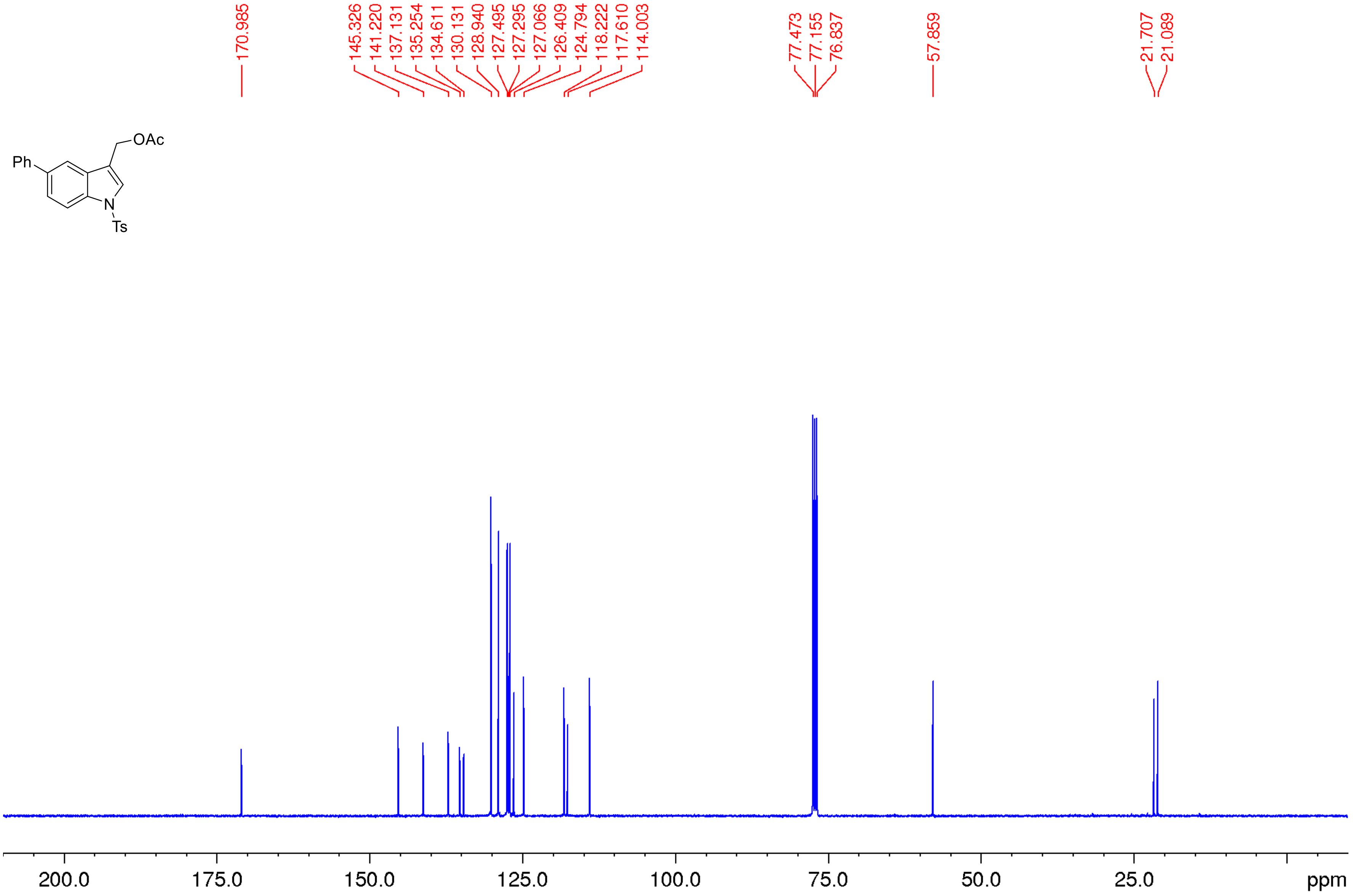

(5-phenyl-1-tosyl-1H-indol-3-yl)methyl acetate ***3e***

DEPT 135 NMR-spectrum ($CDCl_3$)

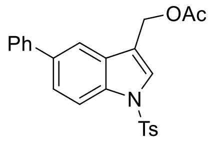

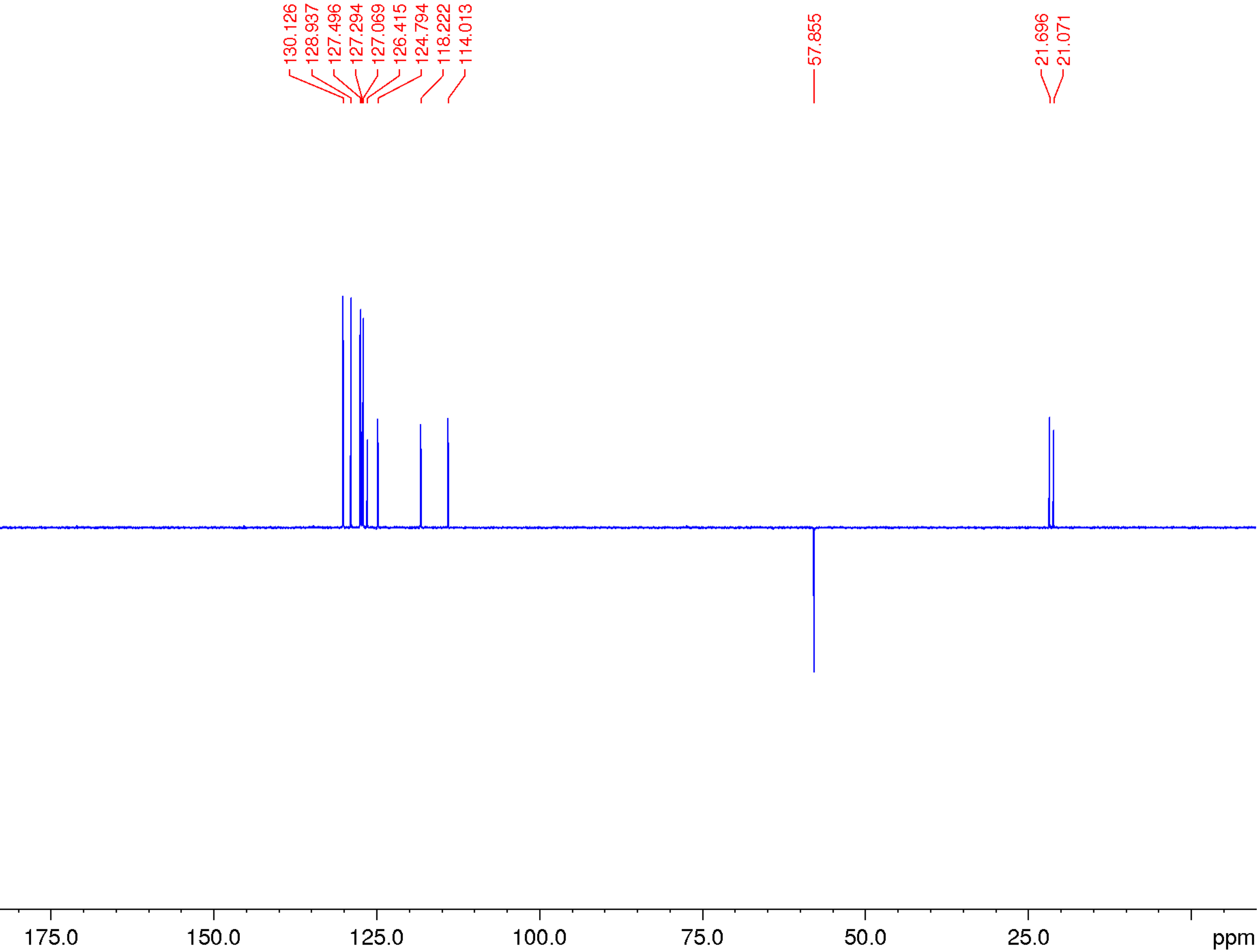

(6-chloro-1-tosyl-1H-indol-3-yl)methyl acetate ***3f***

^{1}H NMR-spectrum (400.13 MHz) ($CDCl_3$)

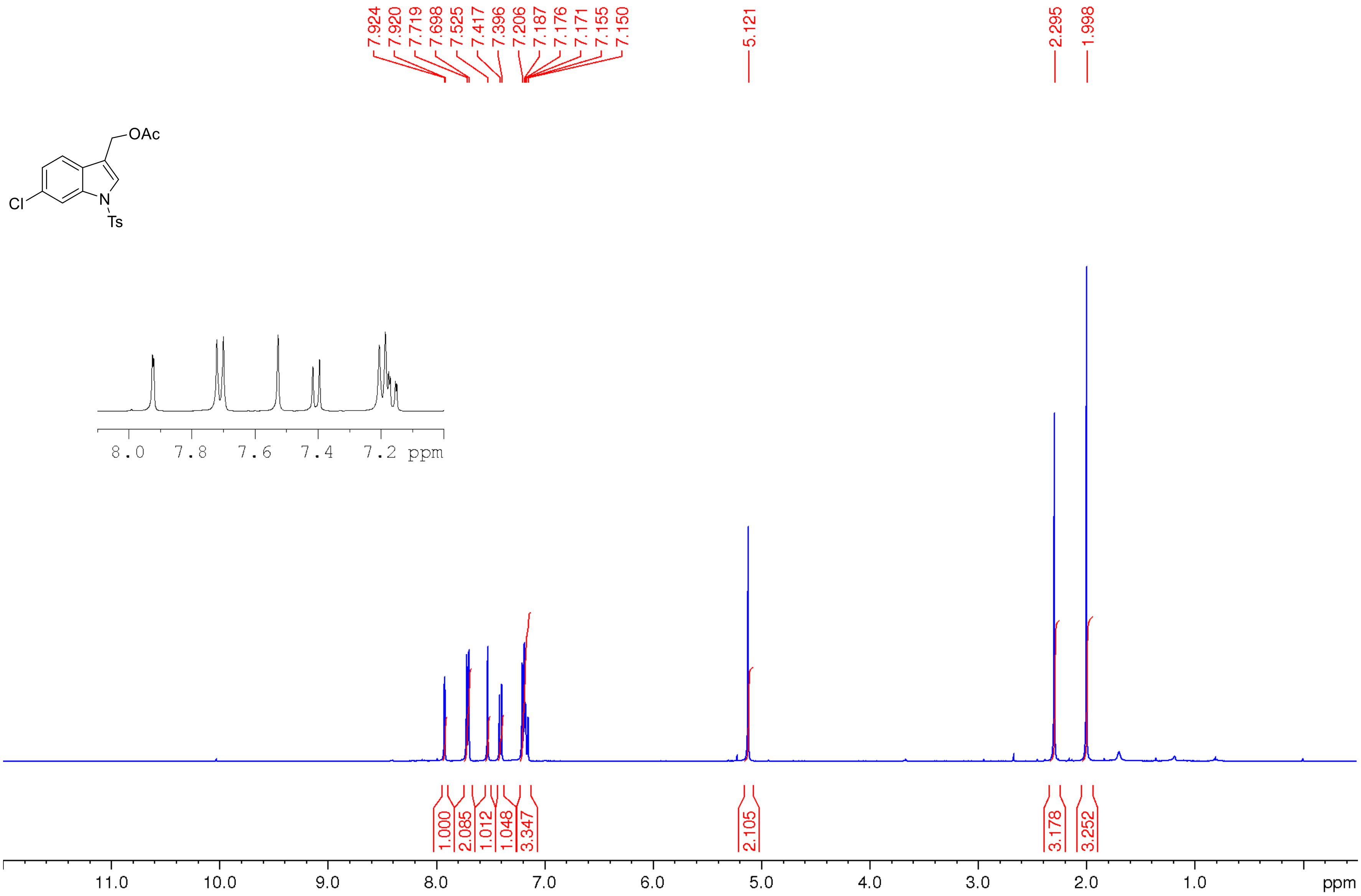

*(6-chloro-1-tosyl-1H-indol-3-yl)methyl acetate **3f***

^{13}C NMR-spectrum (100.6 MHz) ($CDCl_3$)

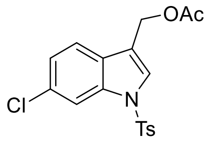

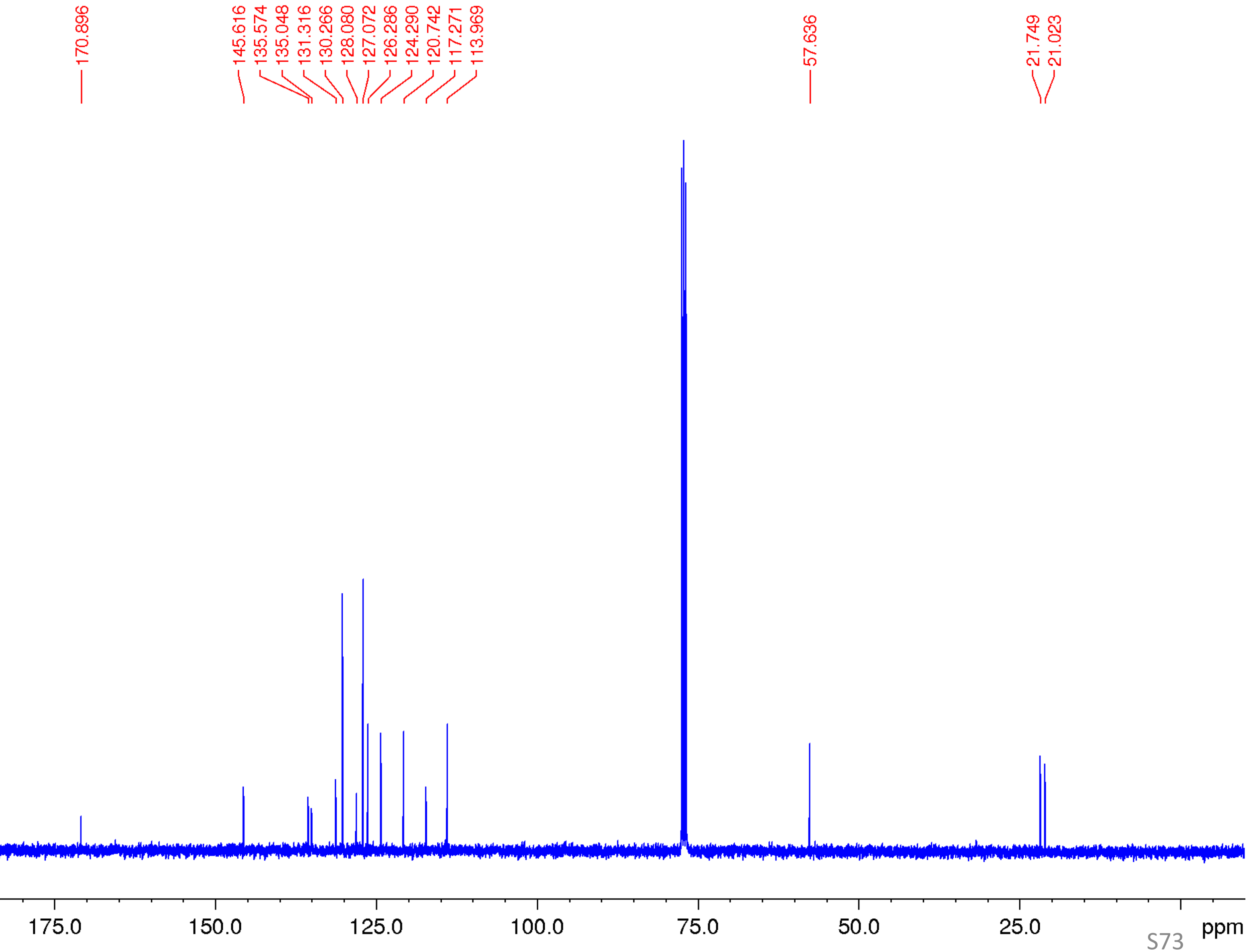

(6-chloro-1-tosyl-1H-indol-3-yl)methyl acetate ***3f***

DEPT 135 NMR-spectrum ($CDCl_3$)

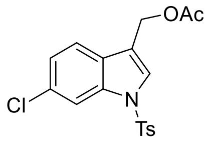

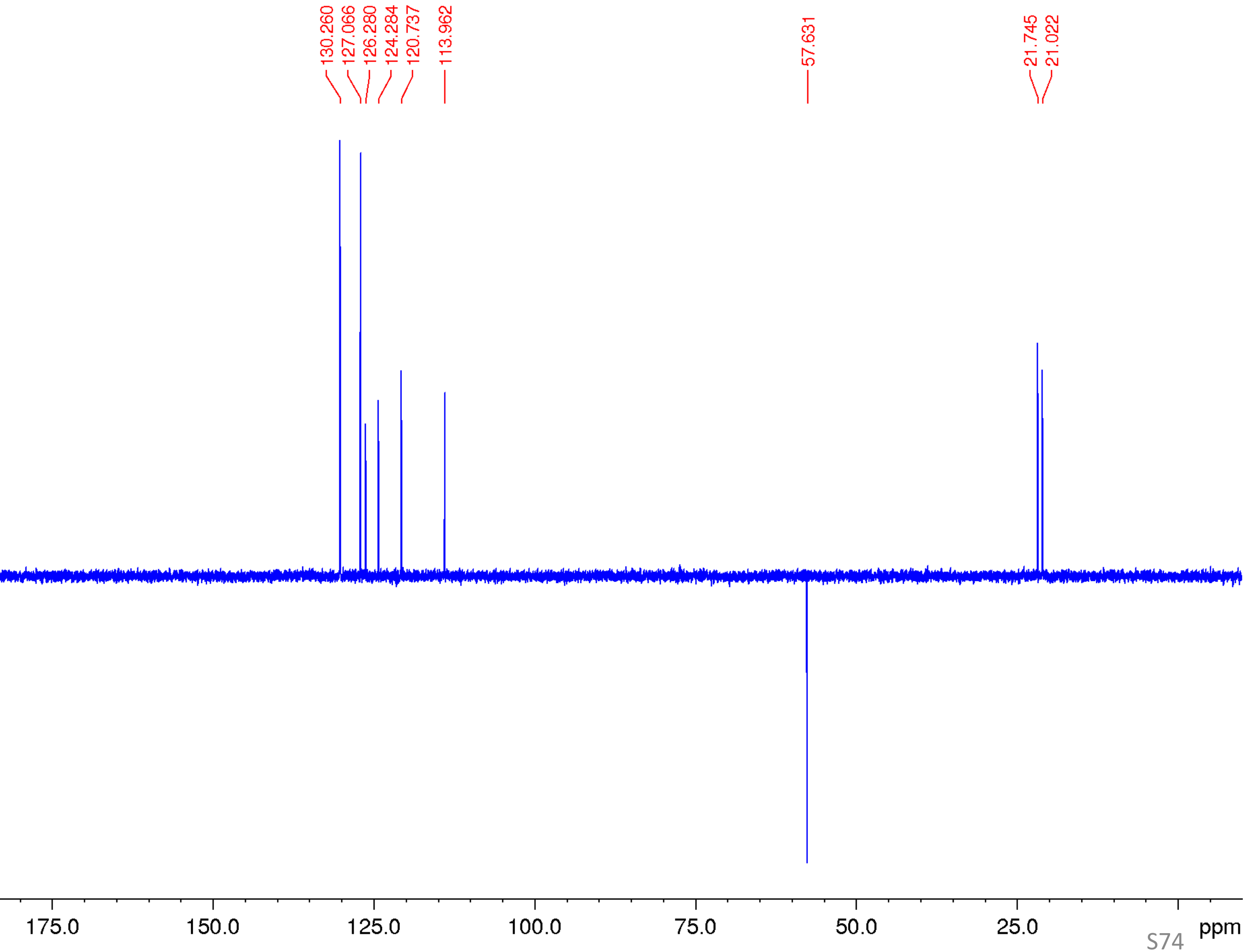

(*5-nitro-1-tosyl-1H-indol-3-yl*)*methyl acetate* ***3g***

^{1}H NMR-spectrum (400.13 MHz) (DMSO-d_6)

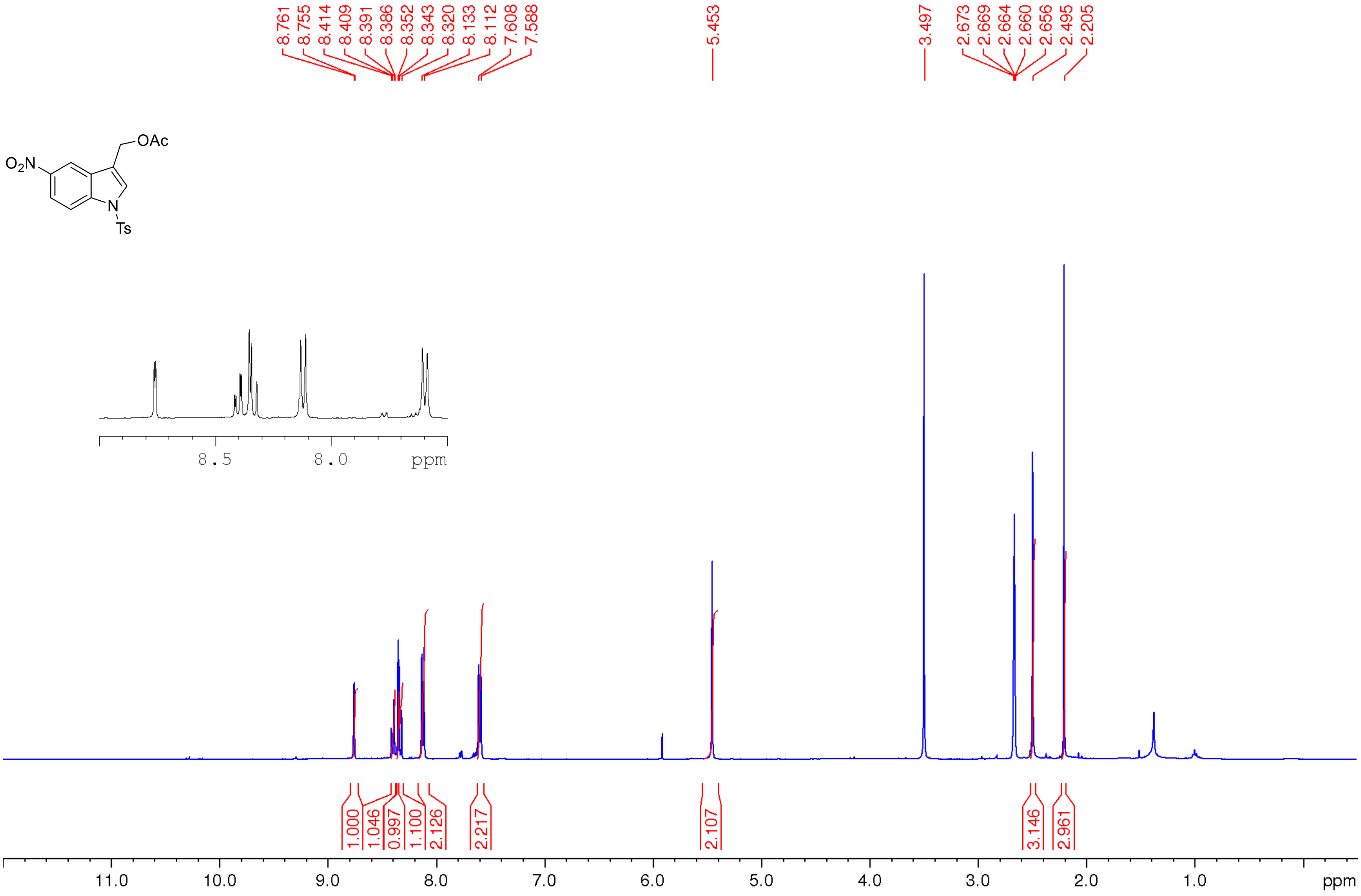

(5-nitro-1-tosyl-1H-indol-3-yl)methyl acetate **3g**

^{13}C NMR-spectrum (100.6 MHz) (DMSO-d_6)

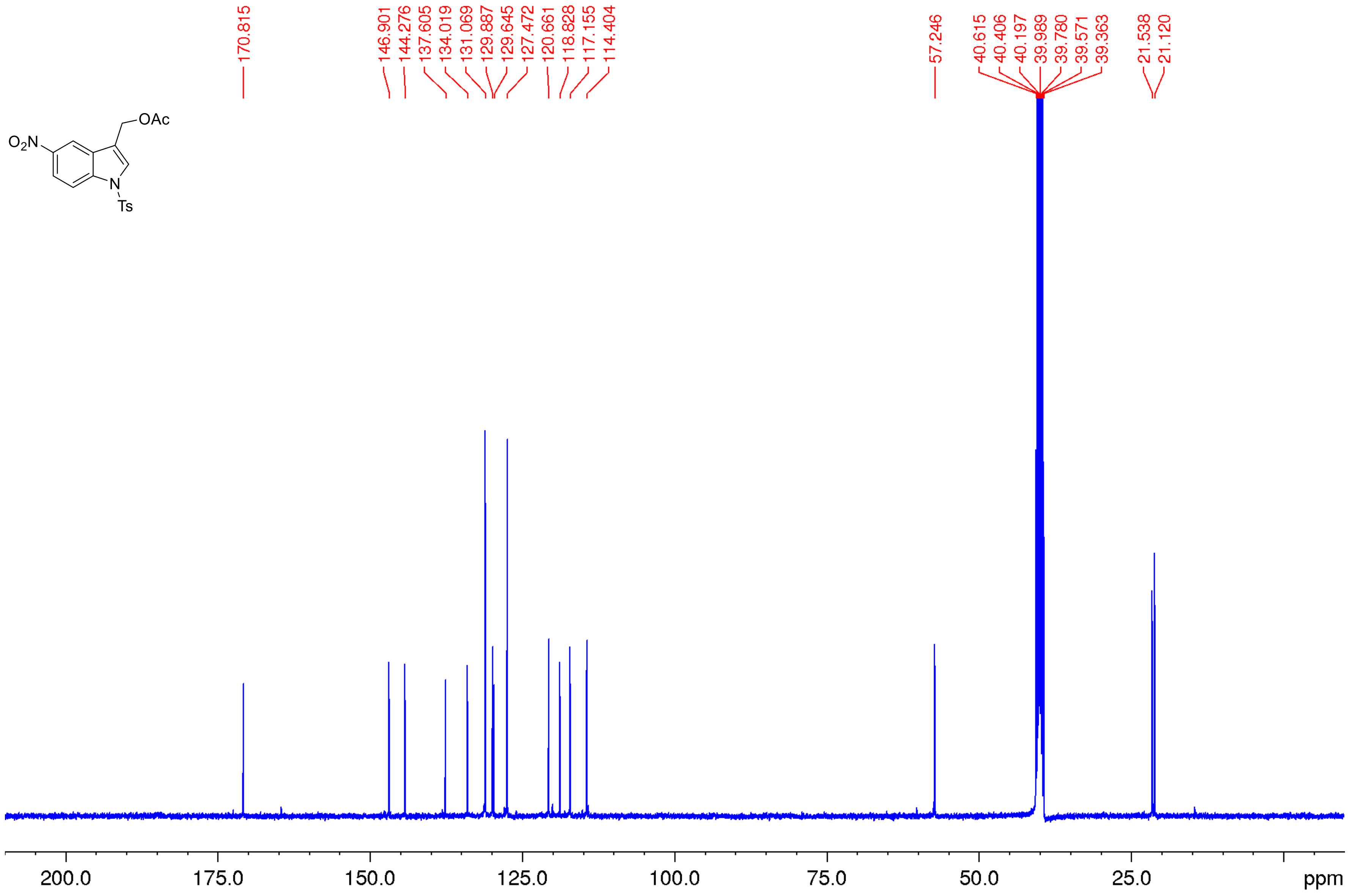

(5-nitro-1-tosyl-1H-indol-3-yl)methyl acetate **3g**

DEPT 135 NMR-spectrum (DMSO-d_6)

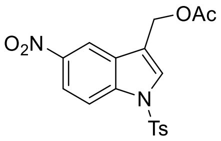

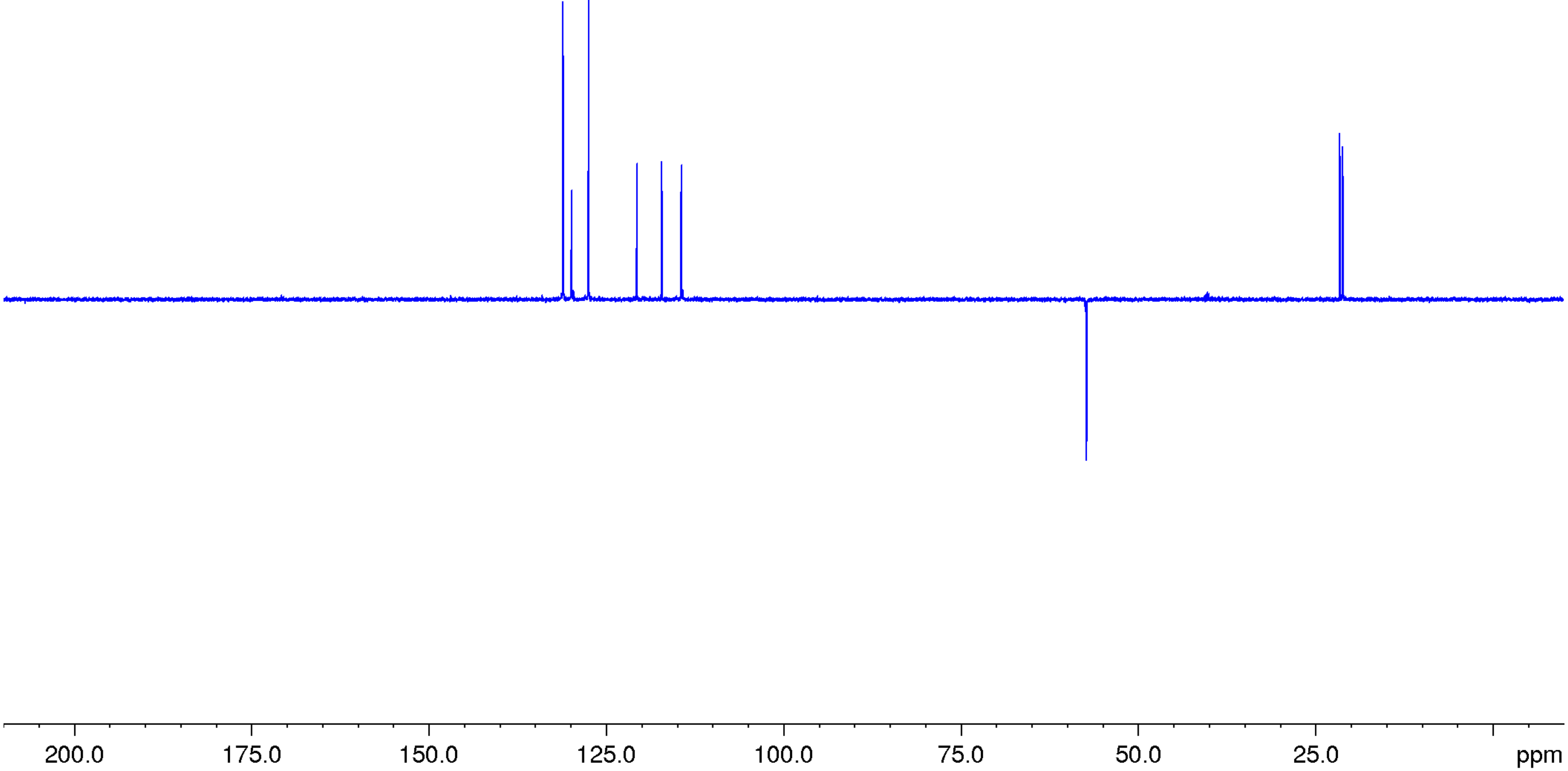

3-((4-methoxyphenoxy)methyl)-1-tosyl-1H-indole ***4aa***

^{1}H NMR-spectrum (400.13 MHz) ($CDCl_3$)

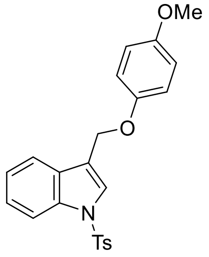

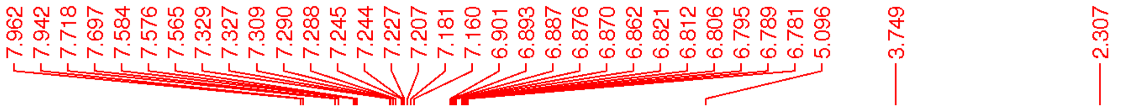

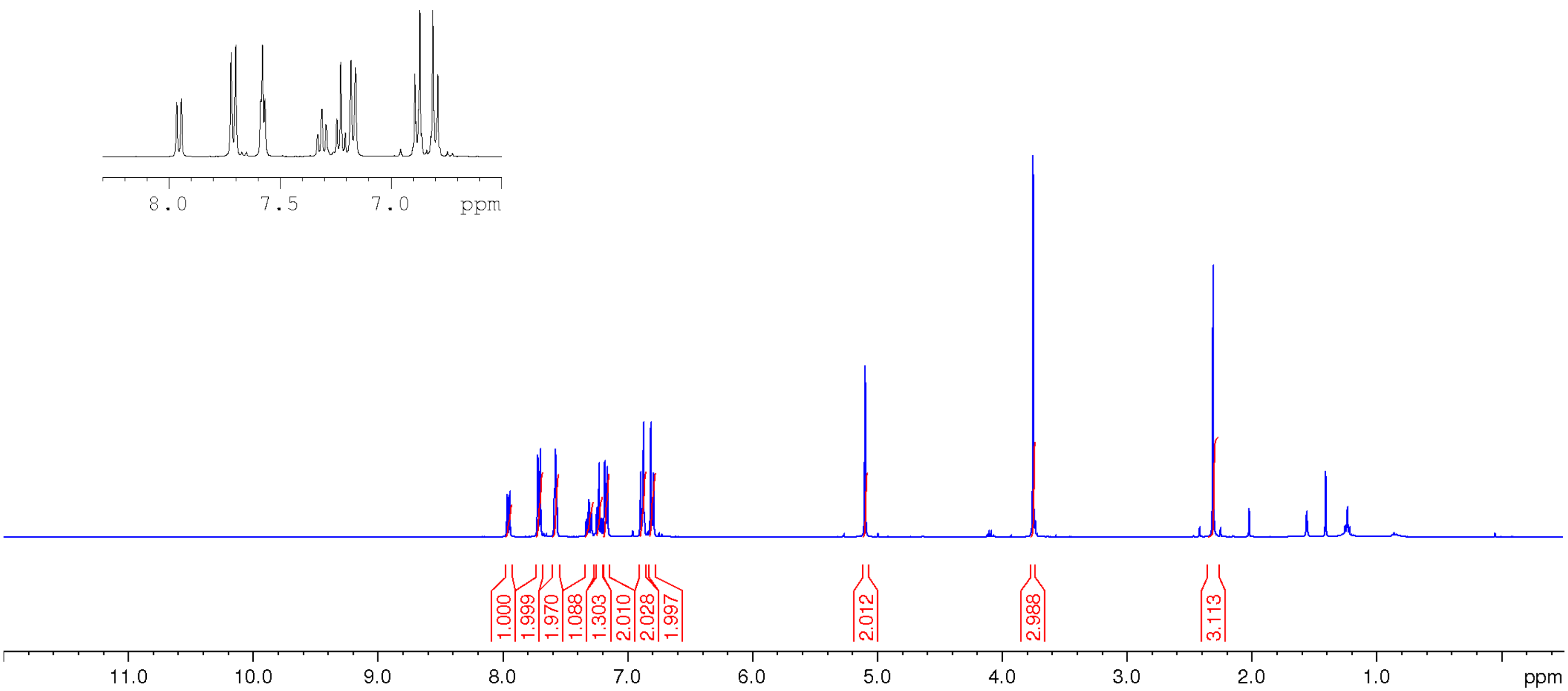

3-((4-methoxyphenoxy)methyl)-1-tosyl-1H-indole ***4aa***

^{13}C NMR-spectrum (100.6 MHz) ($CDCl_3$)

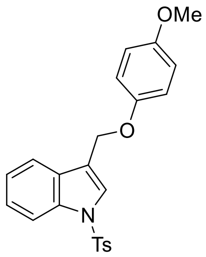

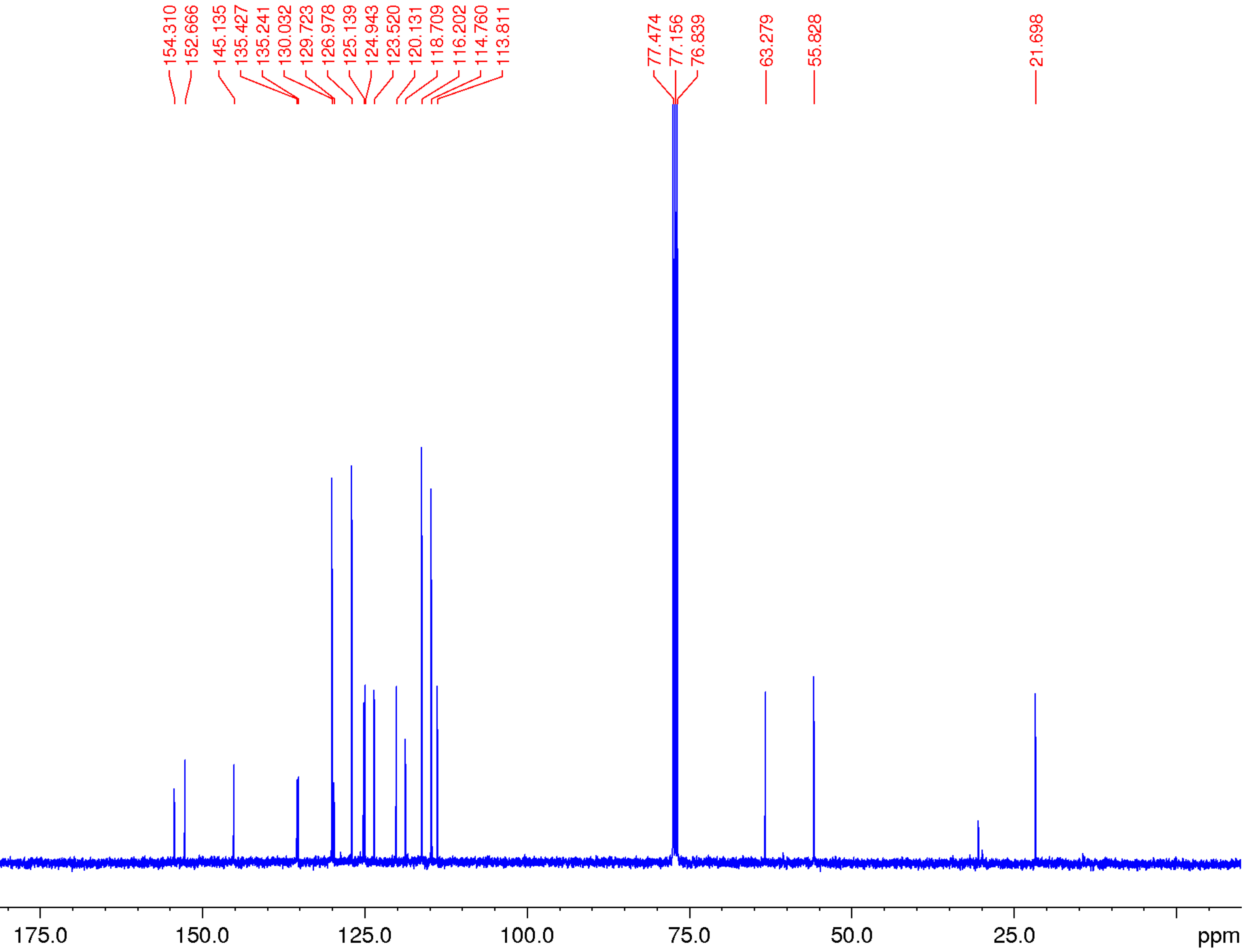

3-((4-methoxyphenoxy)methyl)-1-tosyl-1H-indole ***4aa***

DEPT 135 NMR-spectrum ($CDCl_3$)

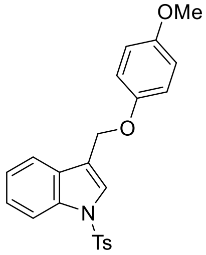

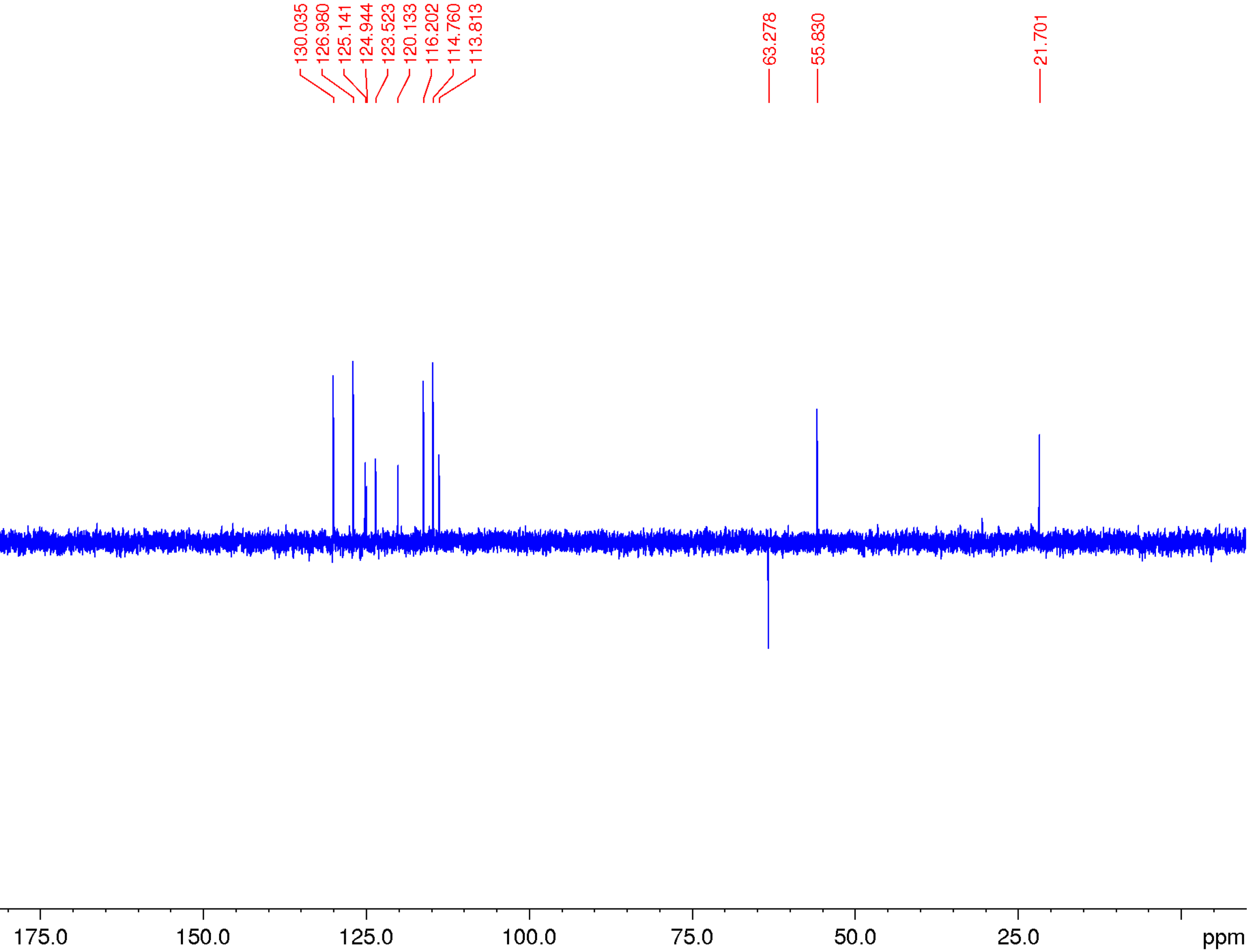

1-benzyl-3-((4-methoxyphenoxy)methyl)-1H-indole ***4ba***

^{1}H NMR-spectrum (400.13 MHz) ($CDCl_3$)

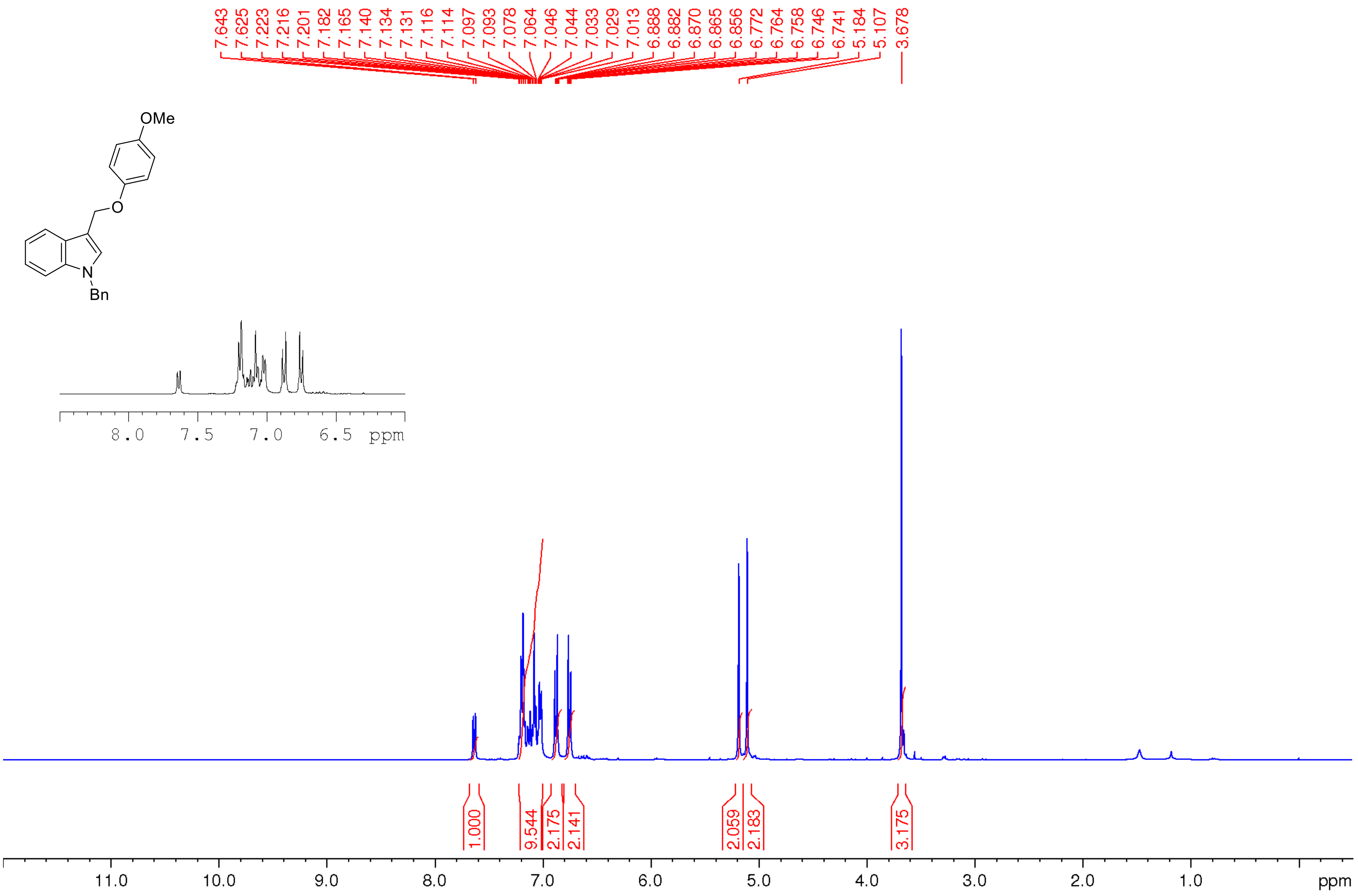

1-benzyl-3-((4-methoxyphenoxy)methyl)-1H-indole ***4ba***

^{13}C NMR-spectrum (100.6 MHz) ($CDCl_3$)

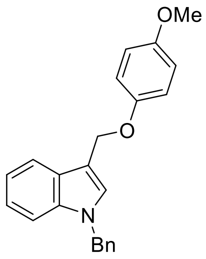

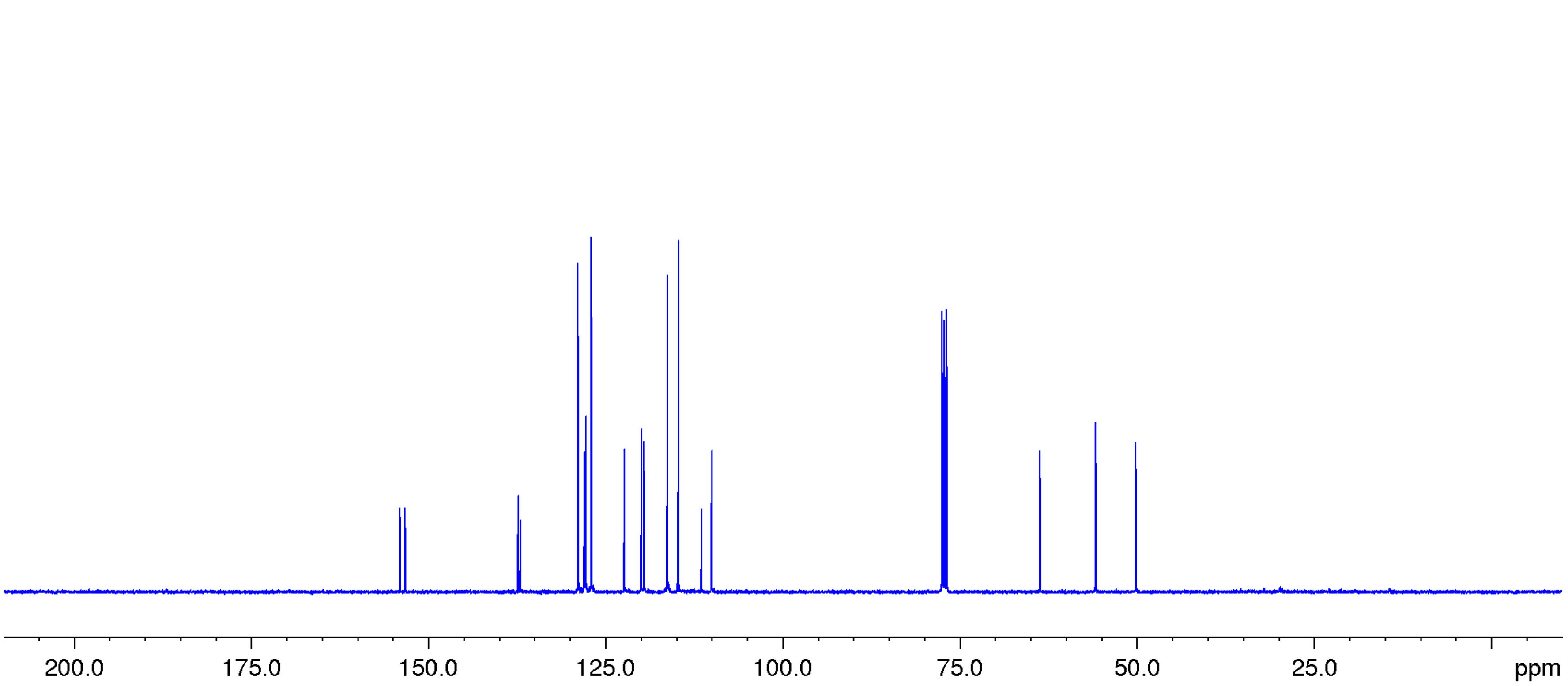

1-benzyl-3-((4-methoxyphenoxy)methyl)-1H-indole ***4ba***

DEPT 135 NMR-spectrum ($CDCl_3$)

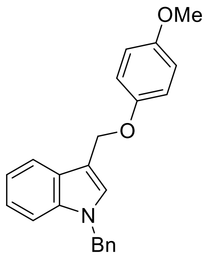

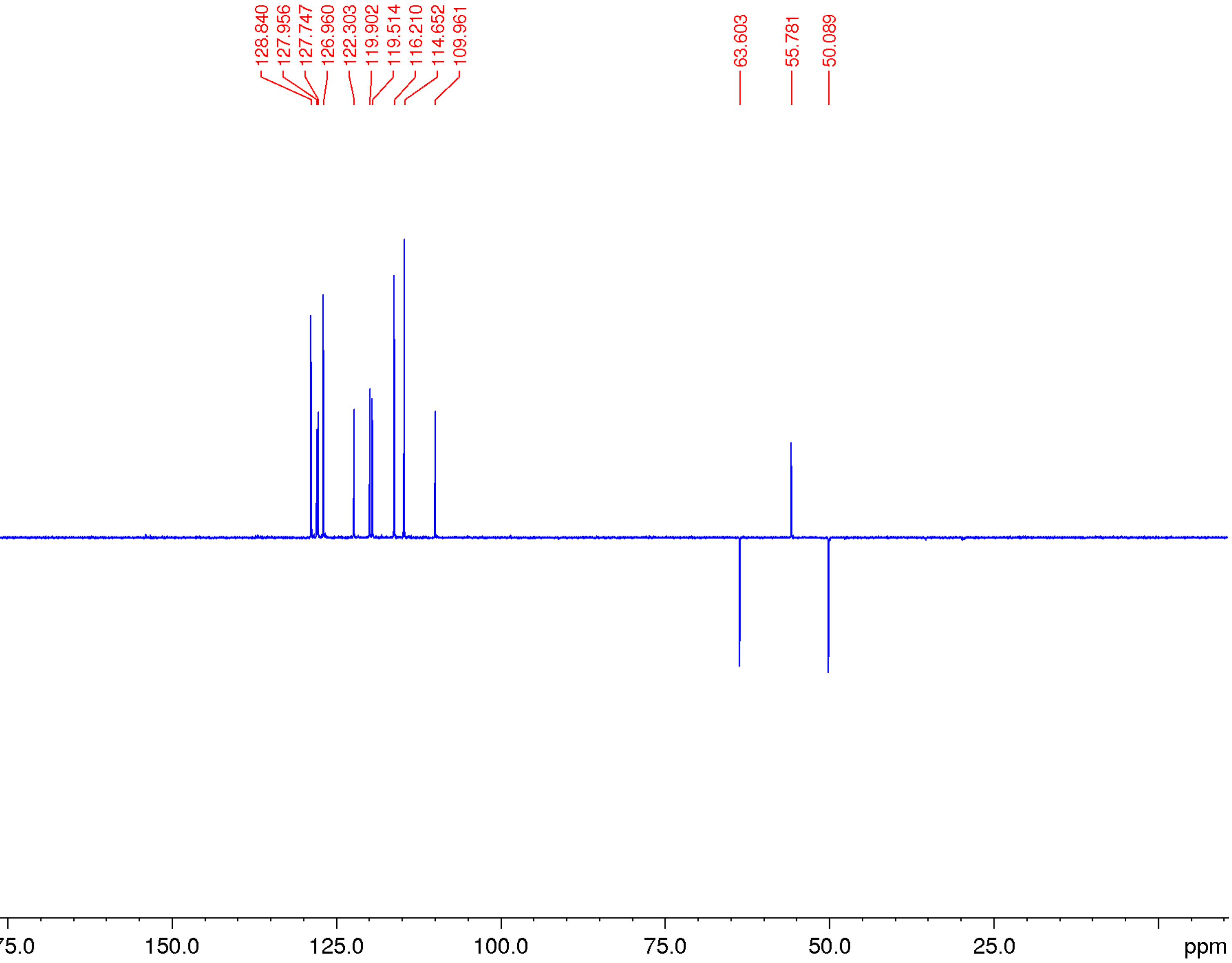

2-((1-benzyl-1H-indol-3-yl)methyl)-4-methoxyphenol ***7ba***

^{1}H NMR-spectrum (400.13 MHz) ($CDCl_3$)

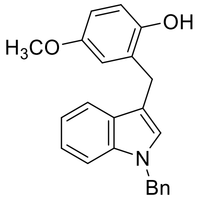

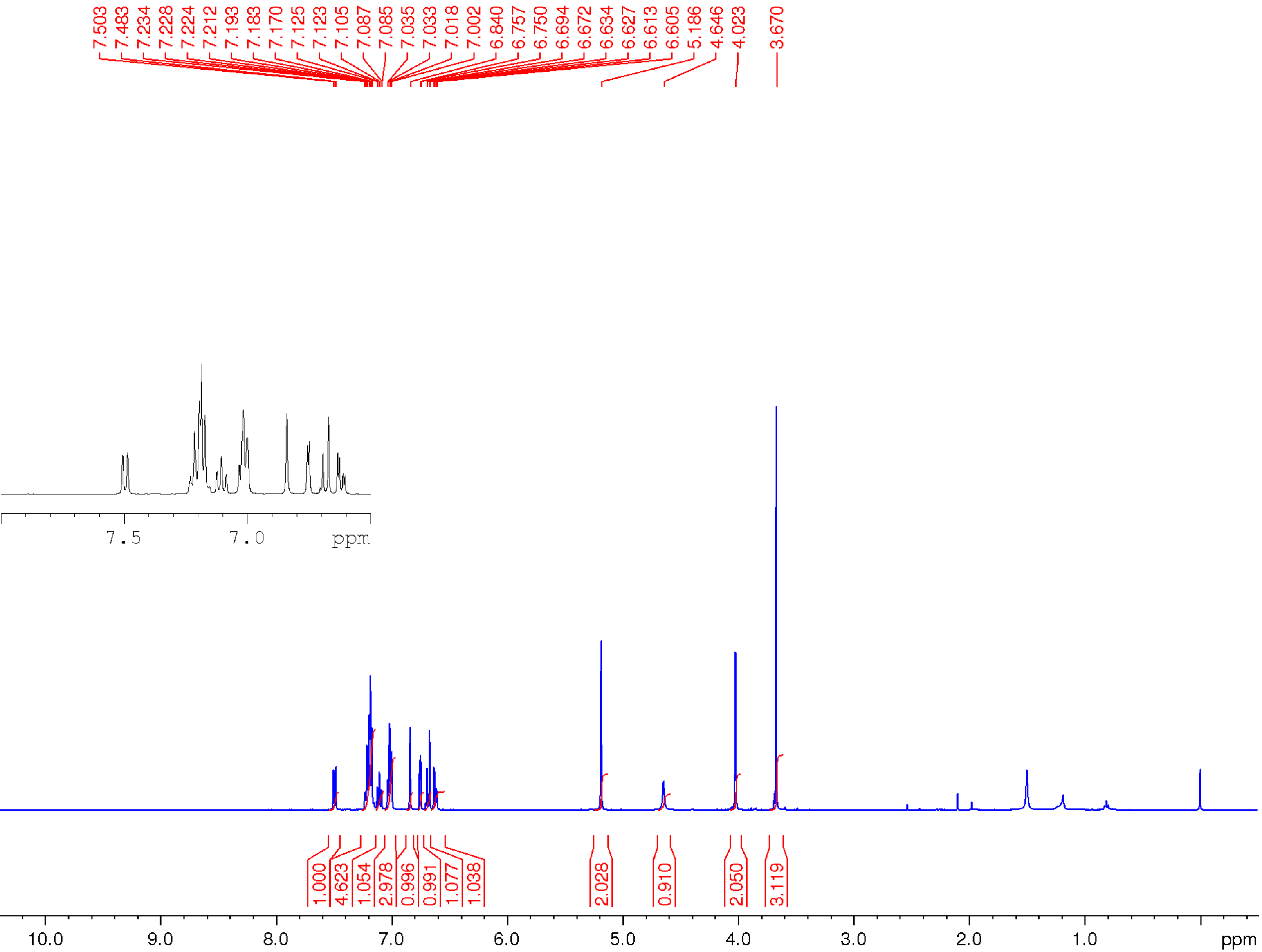

2-((1-benzyl-1H-indol-3-yl)methyl)-4-methoxyphenol ***7ba***

^{13}C NMR-spectrum (100.6 MHz) ($CDCl_3$)

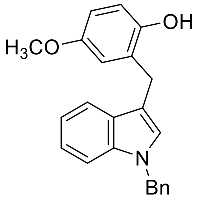

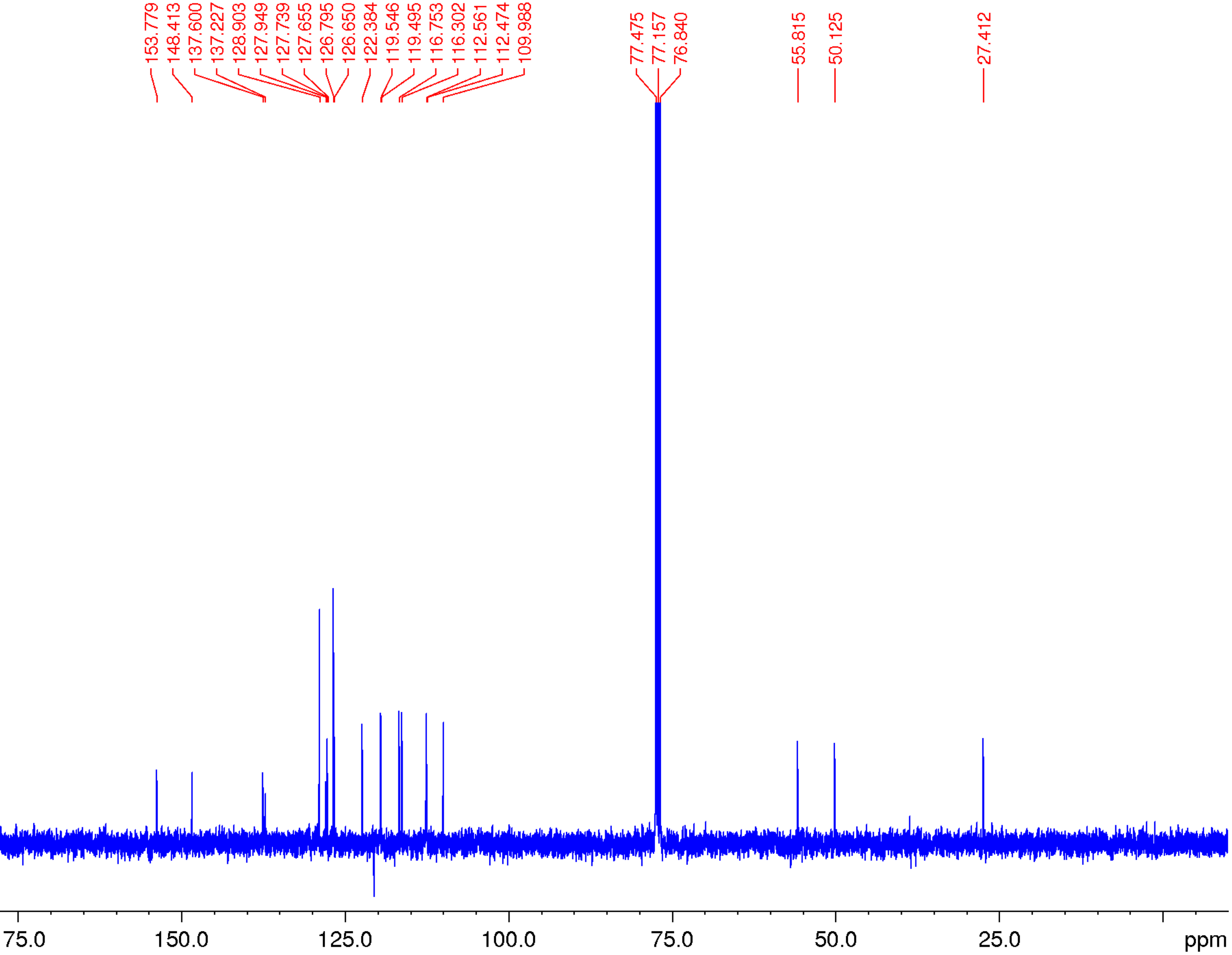

2-((1-benzyl-1H-indol-3-yl)methyl)-4-methoxyphenol ***7ba***

DEPT 135 NMR-spectrum ($CDCl_3$)

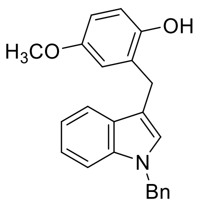

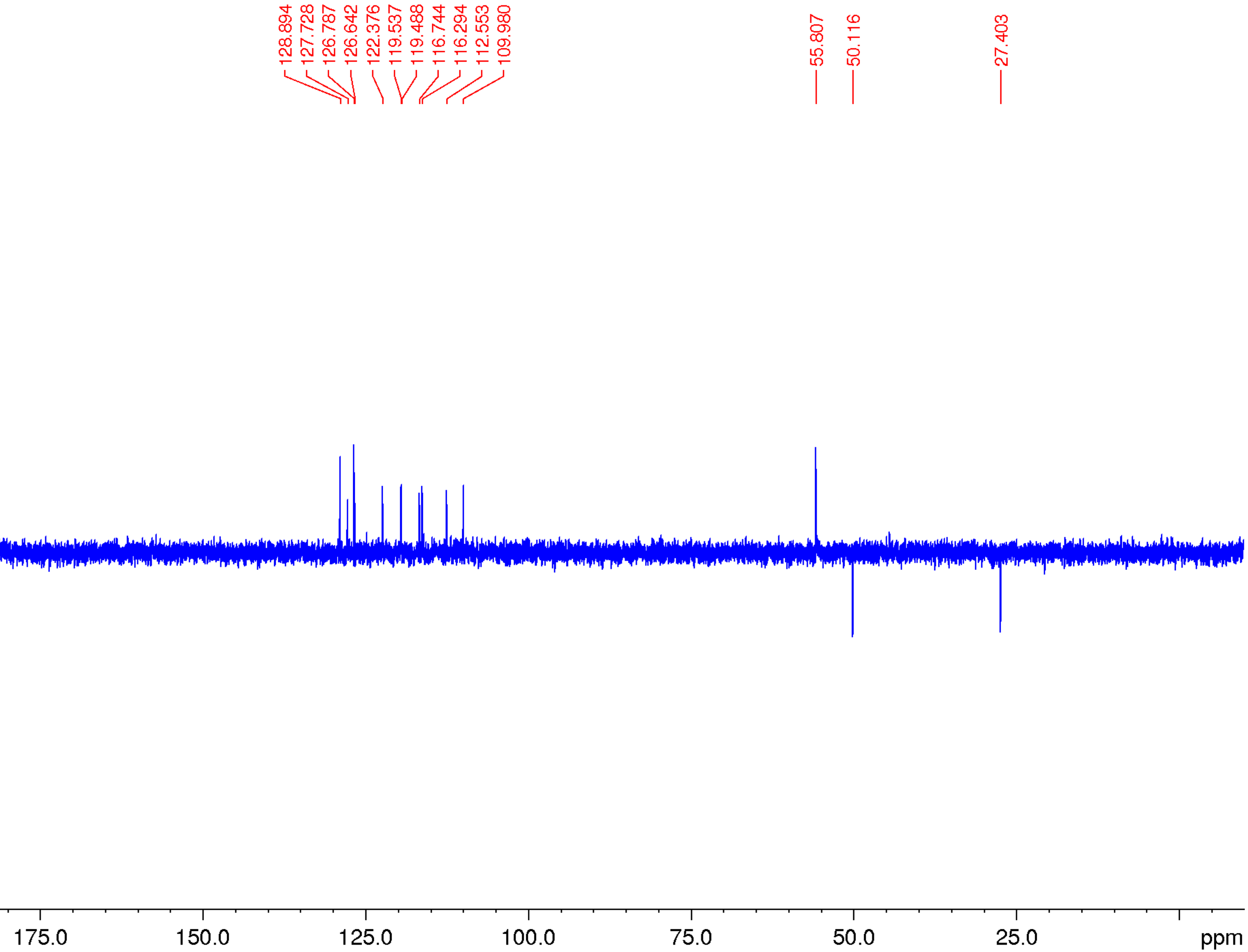

3-((4-methoxyphenoxy)methyl)-1-((2-(trimethylsilyl)ethoxy)methyl)-1H-indole ***4ca***

^{1}H NMR-spectrum (400.13 MHz) ($CDCl_3$)

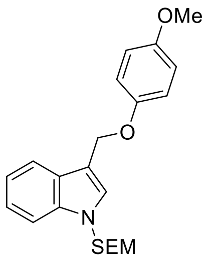

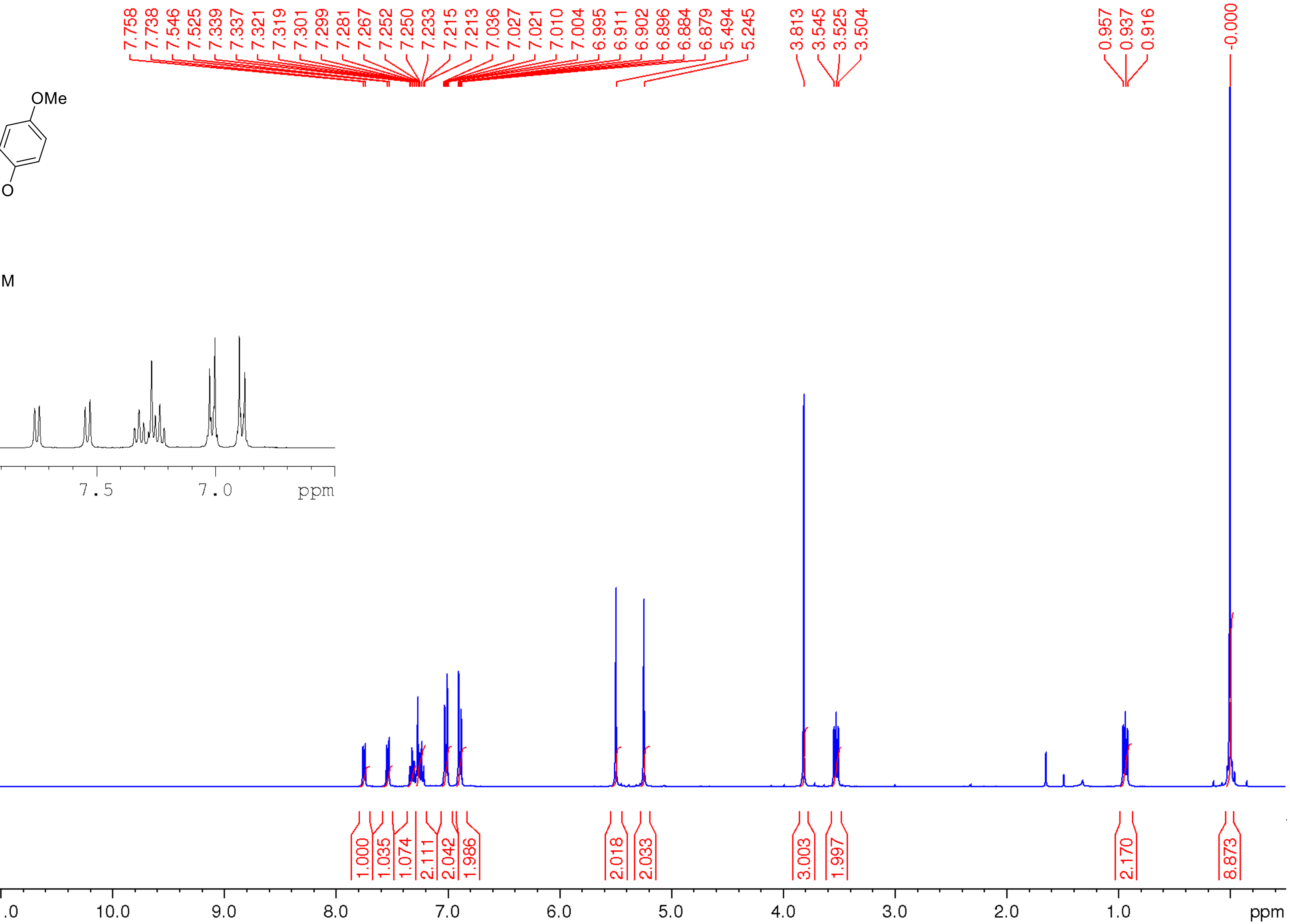

3-((4-methoxyphenoxy)methyl)-1-((2-(trimethylsilyl)ethoxy)methyl)-1H-indole ***4ca***

^{13}C NMR-spectrum (100.6 MHz) ($CDCl_3$)

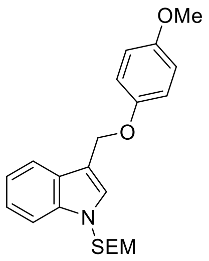

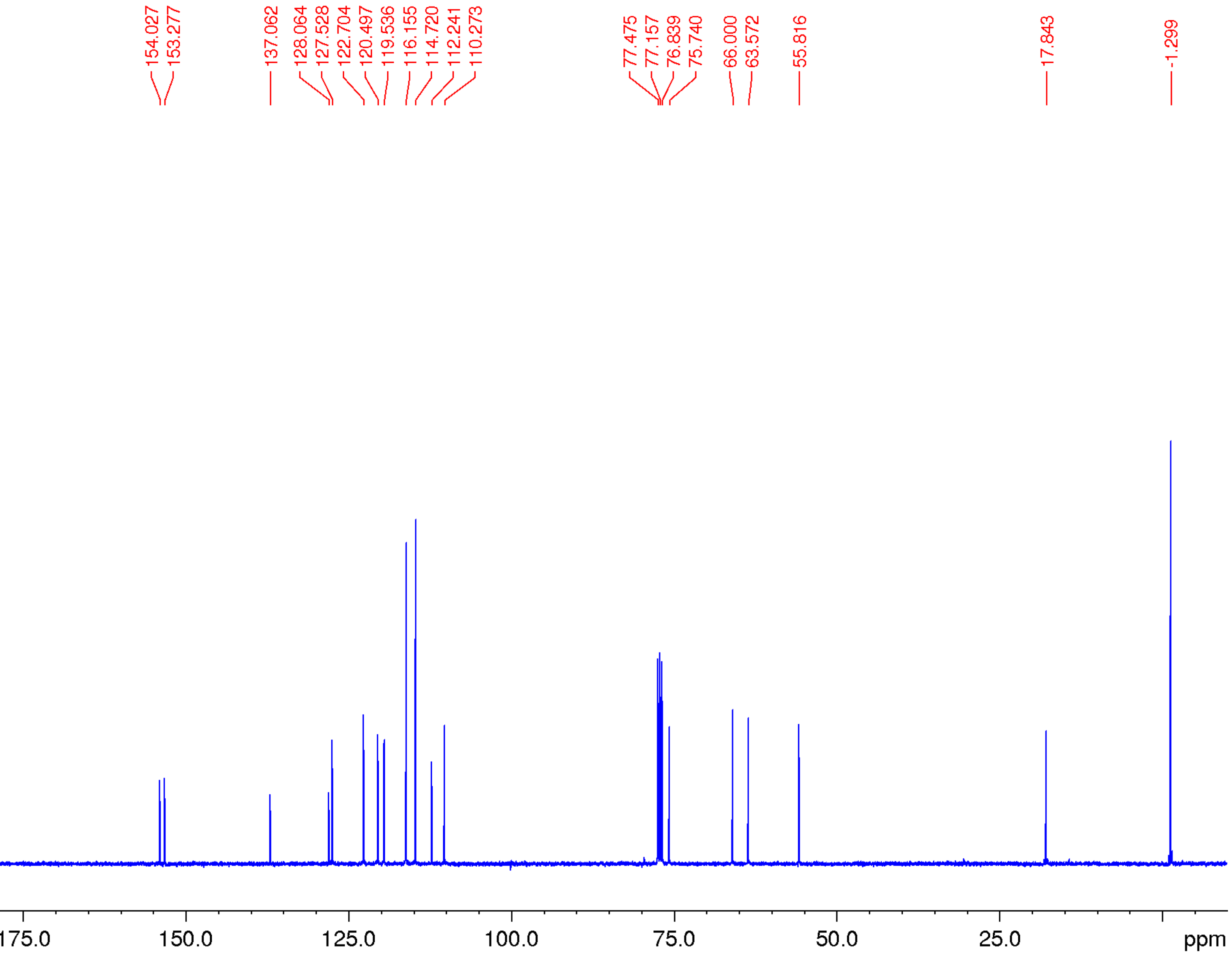

3-((4-methoxyphenoxy)methyl)-1-((2-(trimethylsilyl)ethoxy)methyl)-1H-indole ***4ca***

DEPT 135 NMR-spectrum ($CDCl_3$)

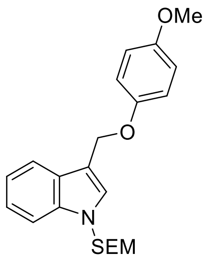

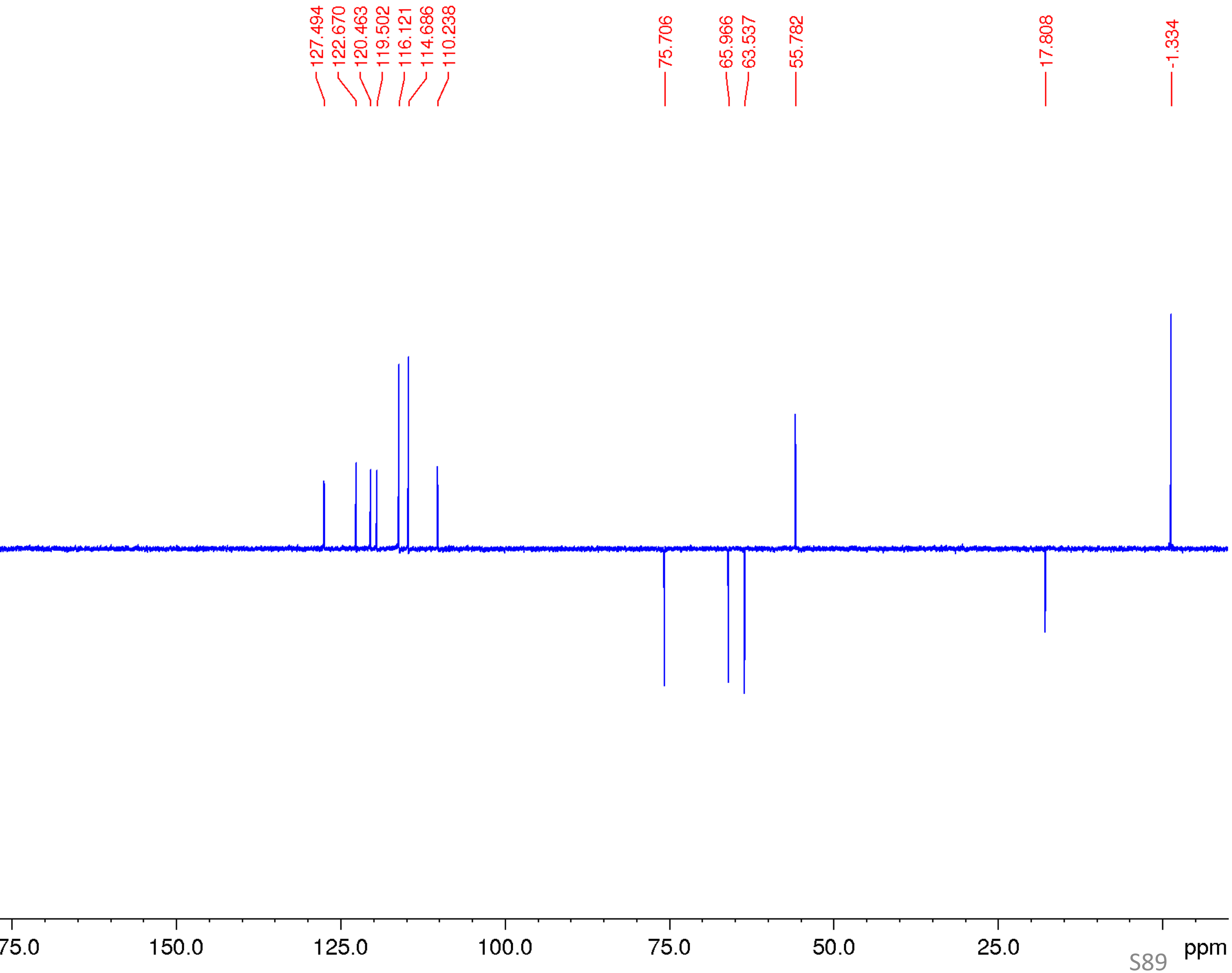

4-methoxy-2-((1-((2-(trimethylsilyl)ethoxy)methyl)-1H-indol-3-yl)methyl)phenol ***7ca***

^{1}H NMR-spectrum (400.13 MHz) ($CDCl_3$)

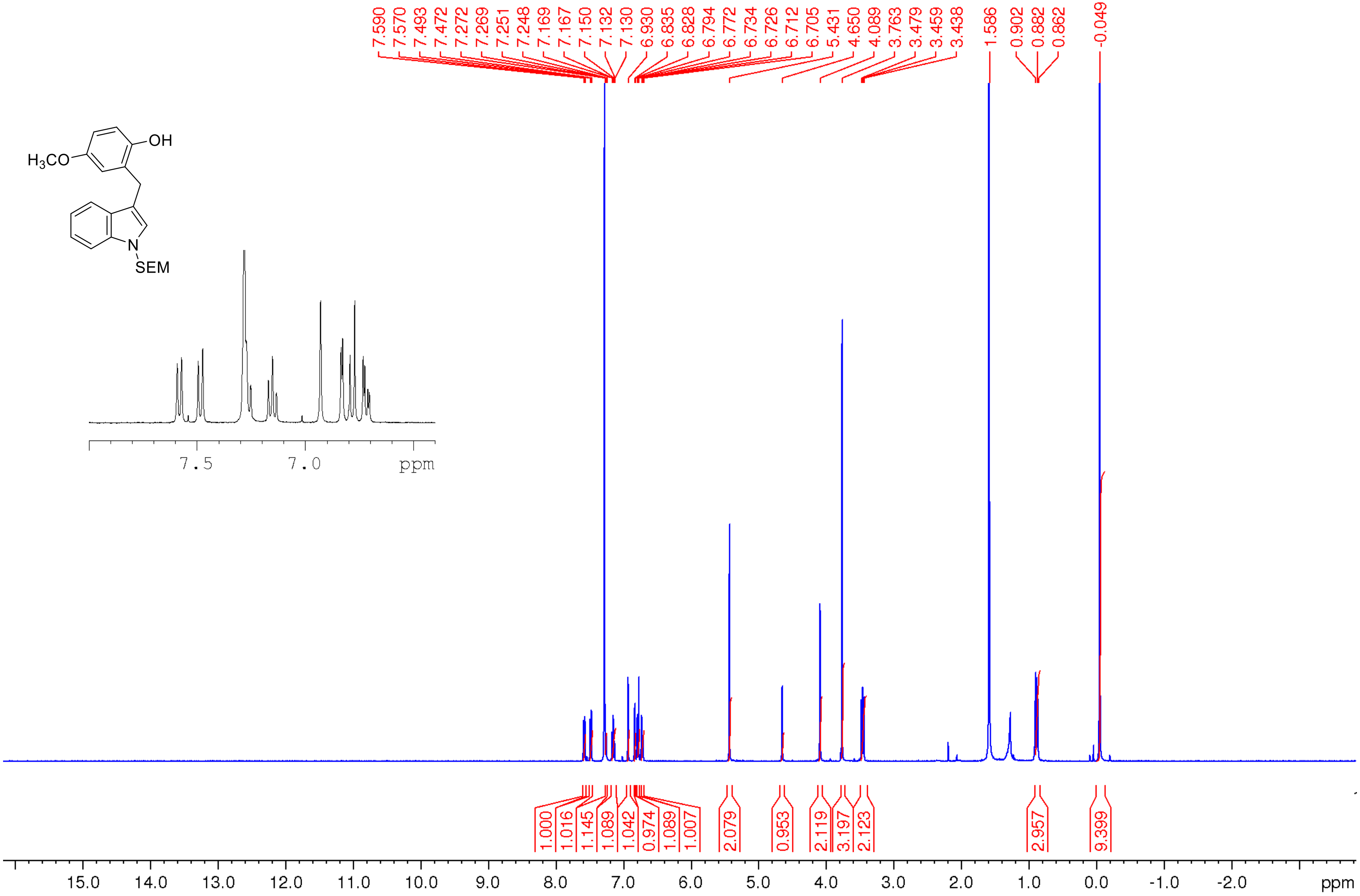

4-methoxy-2-((1-((2-(trimethylsilyl)ethoxy)methyl)-1H-indol-3-yl)methyl)phenol ***7ca***

^{13}C NMR-spectrum (100.6 MHz) ($CDCl_3$)

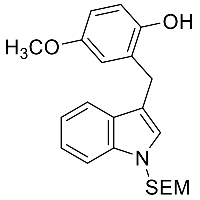

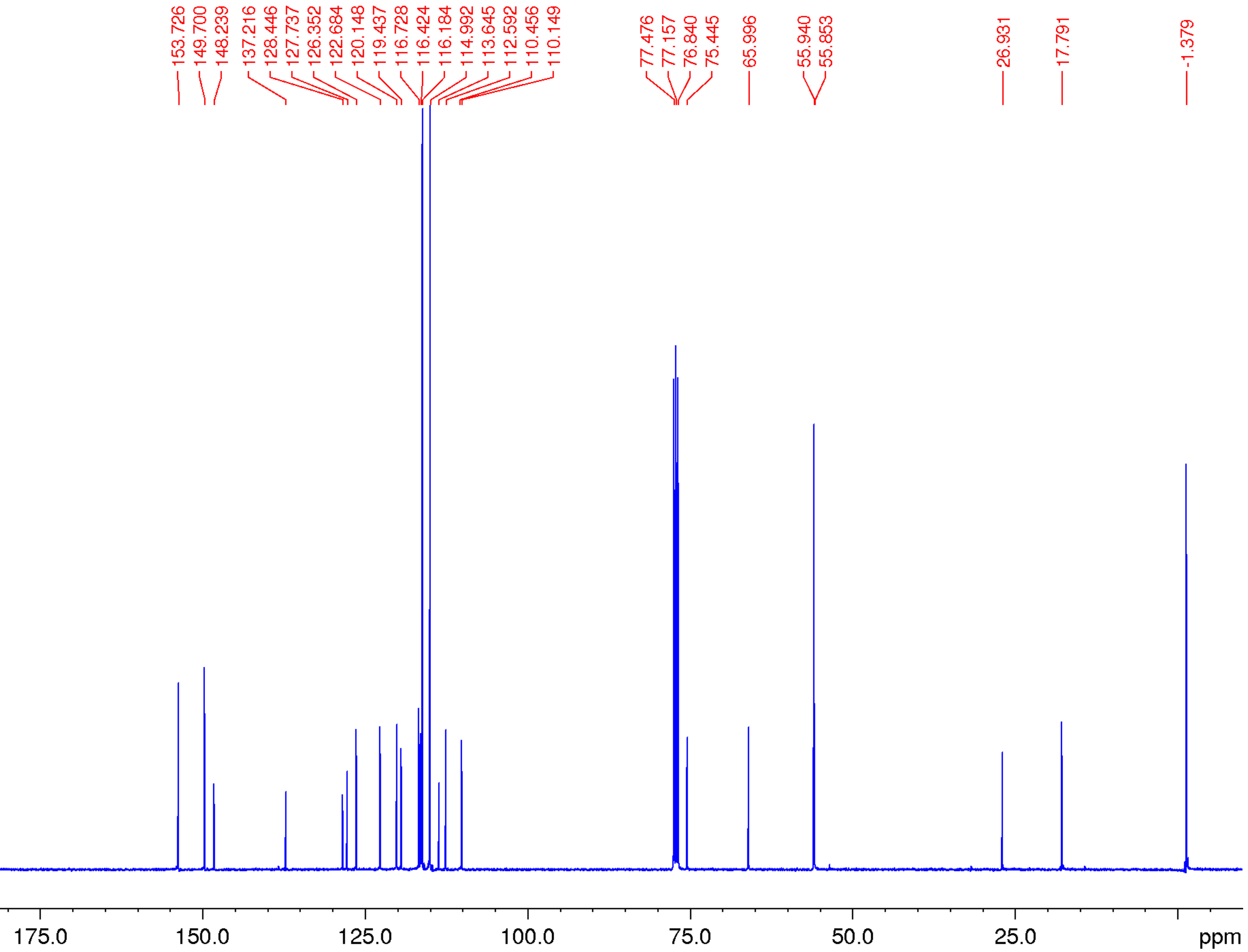

4-methoxy-2-((1-((2-(trimethylsilyl)ethoxy)methyl)-1H-indol-3-yl)methyl)phenol ***7ca***

DEPT 135 NMR-spectrum ($CDCl_3$)

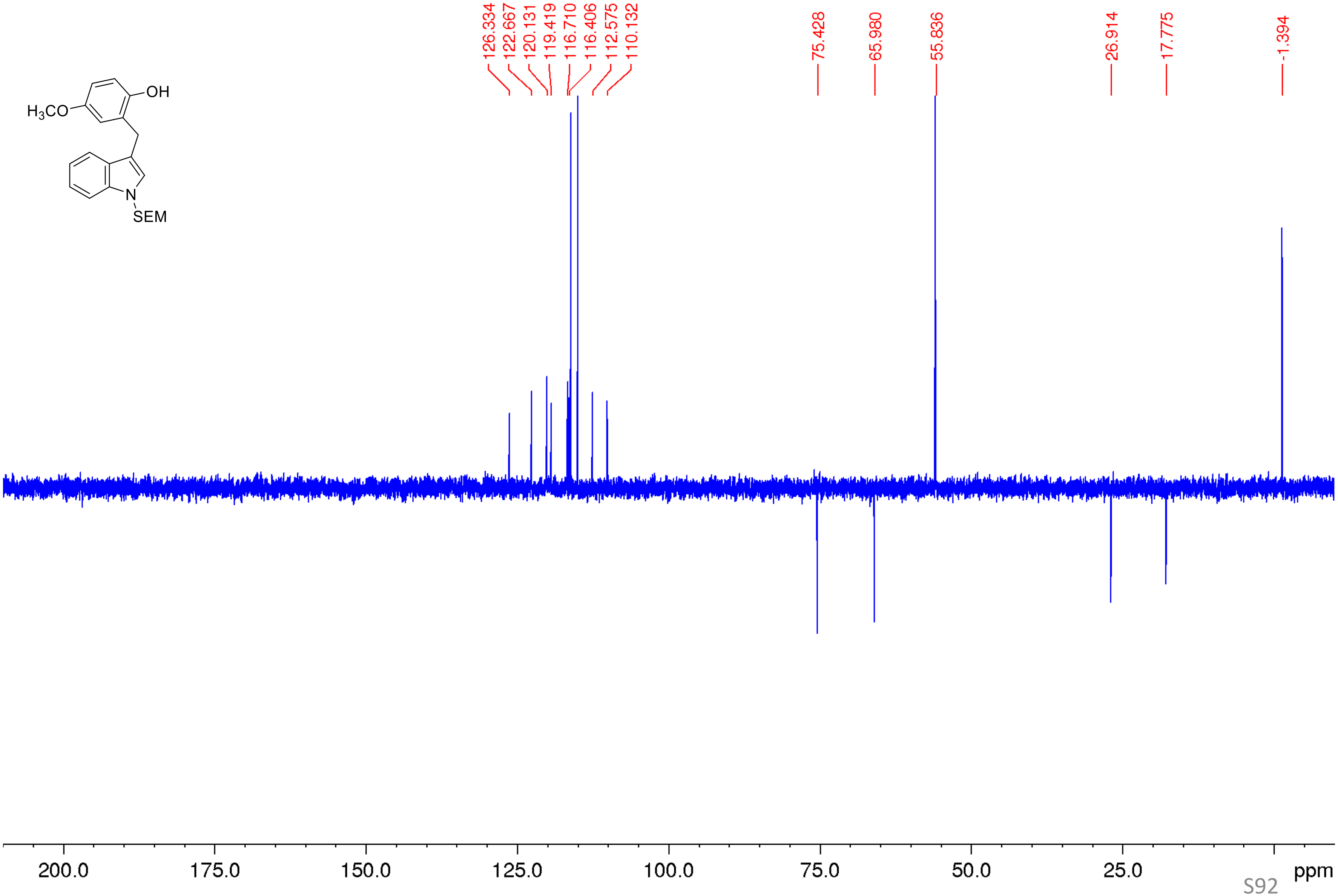

1-tosyl-3-(tosylmethyl)-1*H*-indole **9aa**

^{1}H NMR-spectrum (400.13 MHz) ($CDCl_3$)

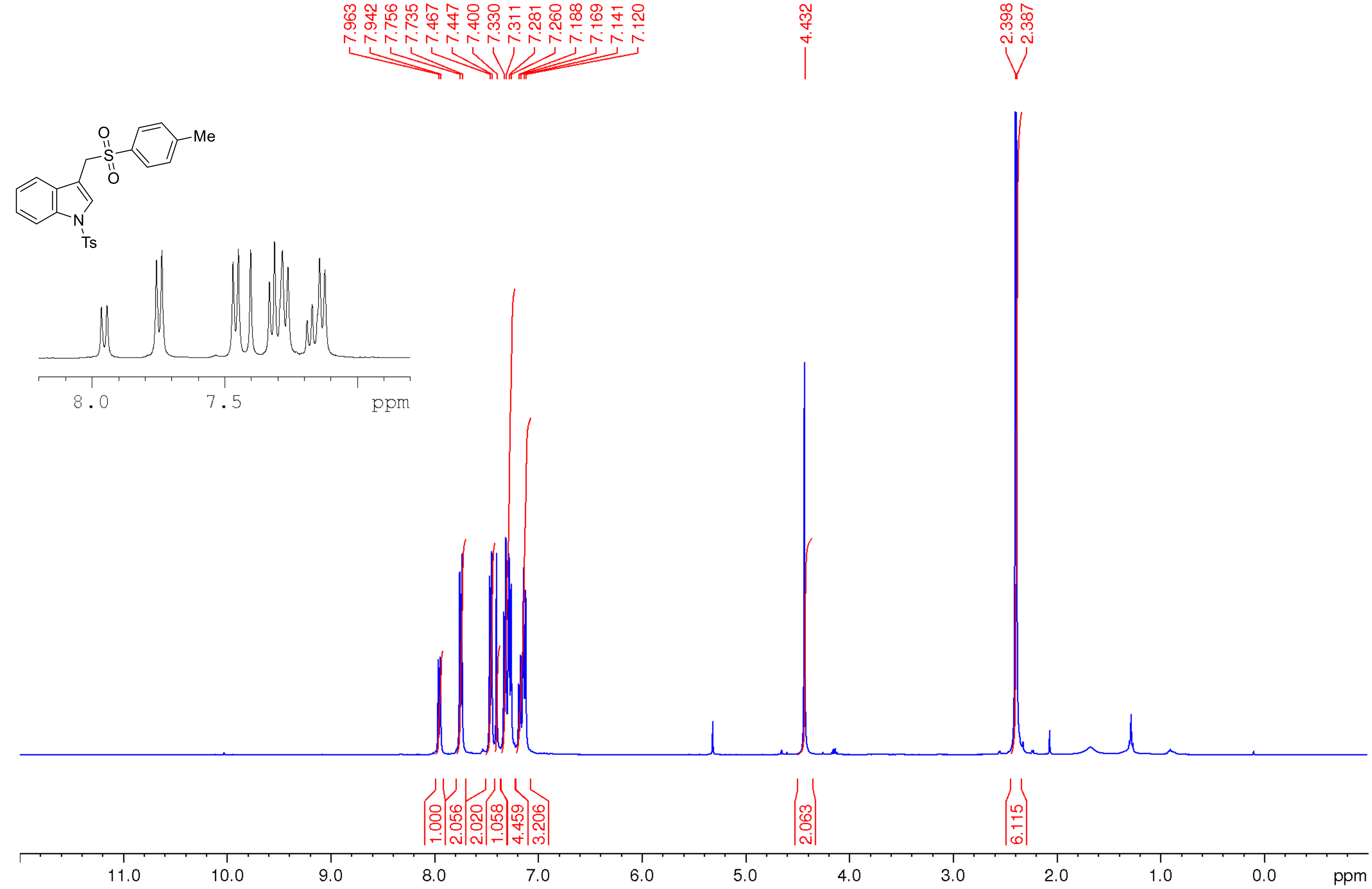

1-tosyl-3-(tosylmethyl)-1*H*-indole **9aa**

^{13}C NMR-spectrum (100.6 MHz) ($CDCl_3$)

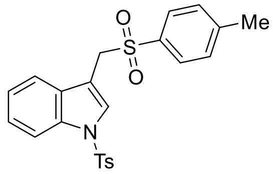

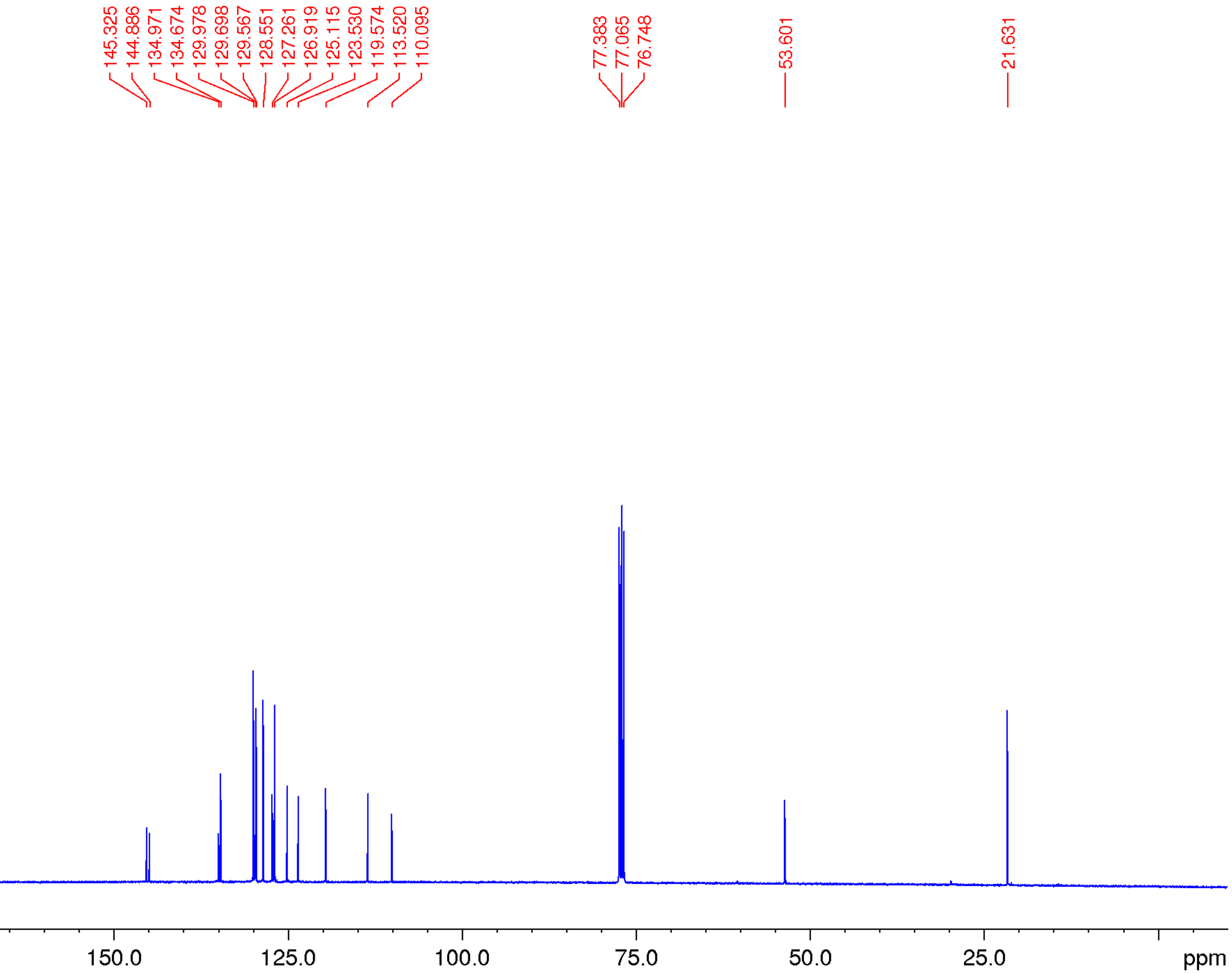

1-tosyl-3-(tosylmethyl)-1*H*-indole **9aa**

DEPT 135 NMR-spectrum ($CDCl_3$)

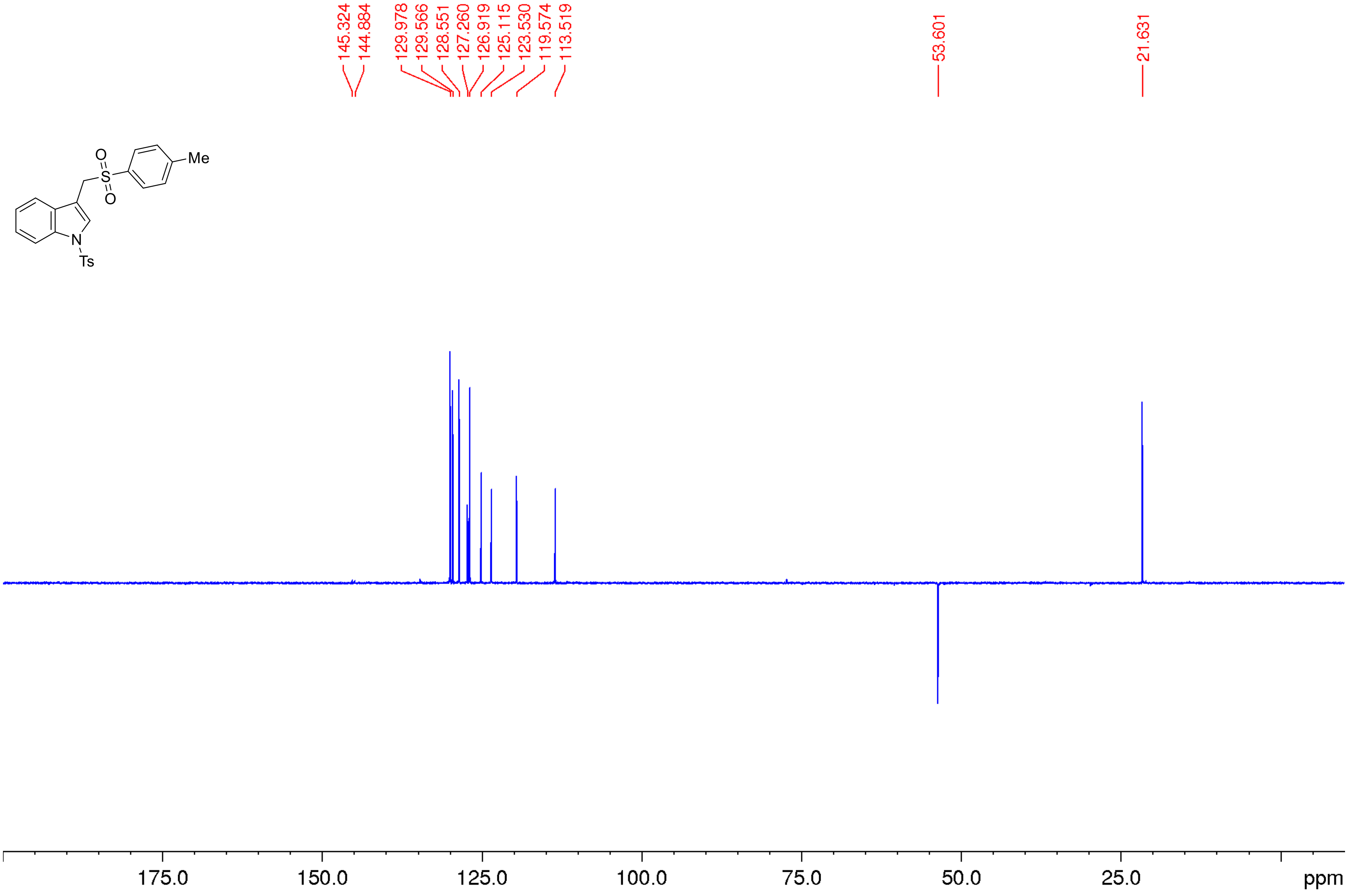

3-((4-(tert-butyl)phenoxy)methyl)-1-tosyl-1H-indole **4ab**

^{1}H NMR-spectrum (400.13 MHz) ($CDCl_3$)

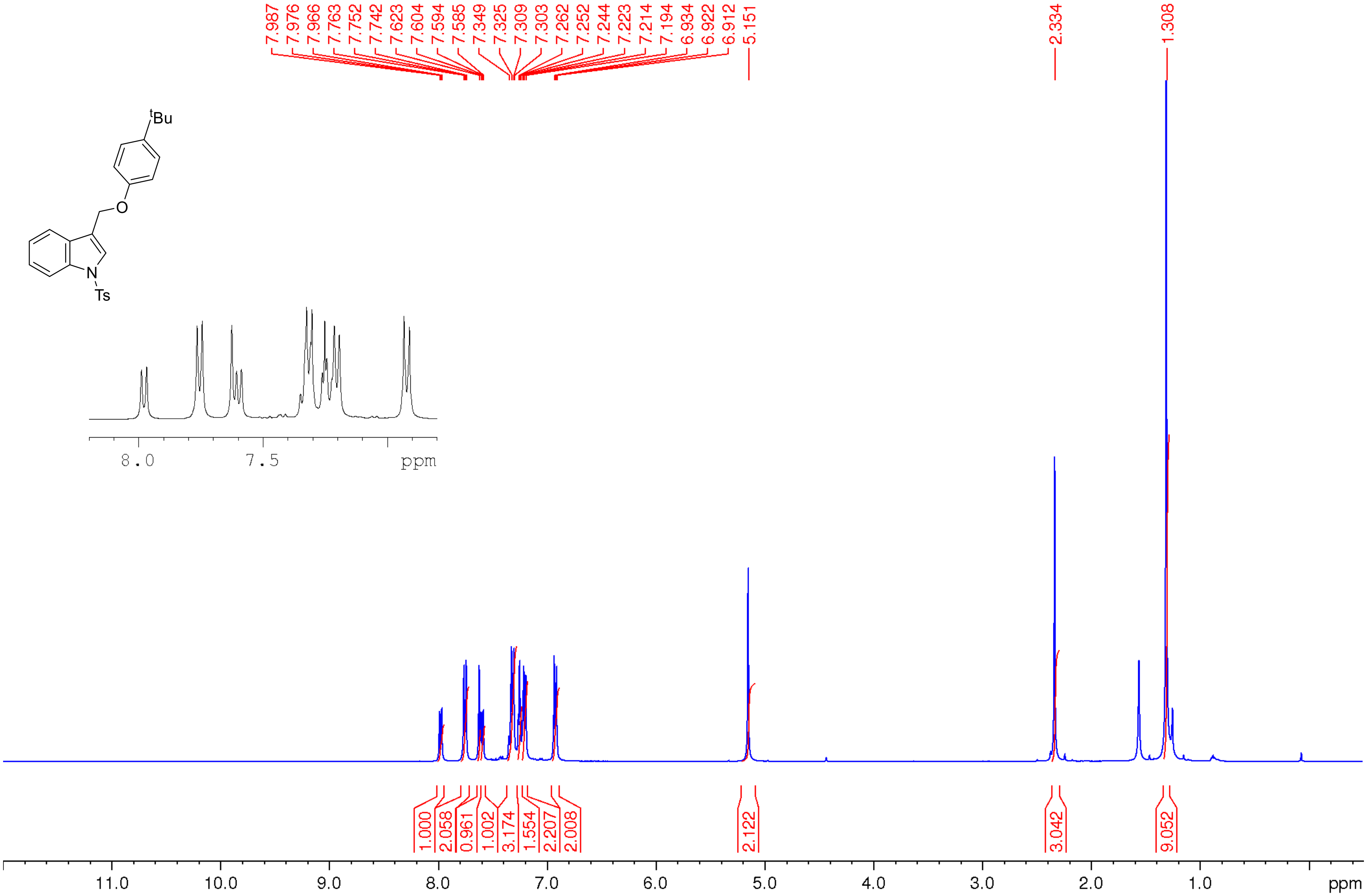

3-((4-(tert-butyl)phenoxy)methyl)-1-tosyl-1H-indole ***4ab***

^{13}C NMR-spectrum (100.6 MHz) ($CDCl_3$)

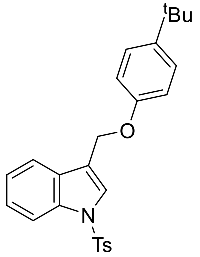

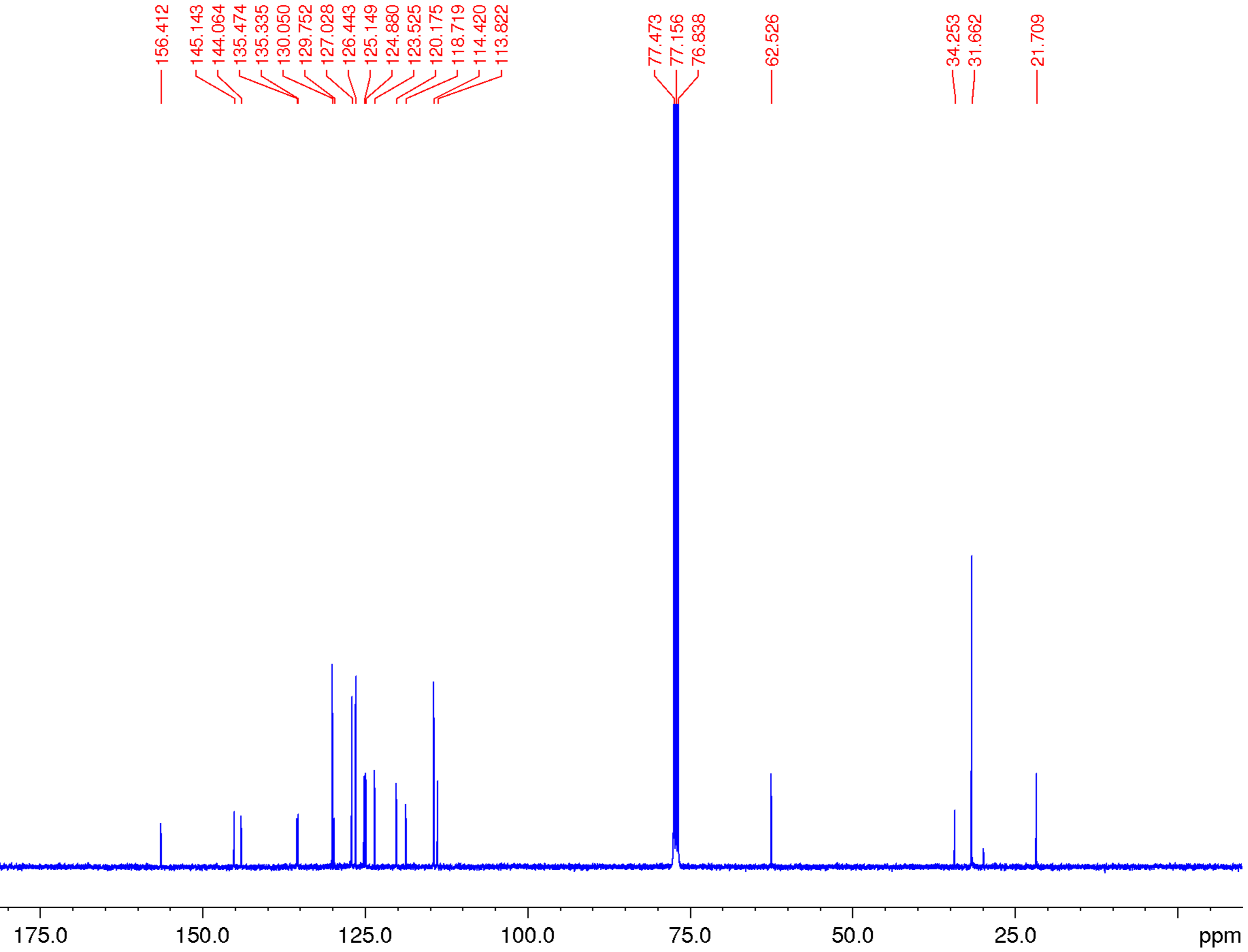

3-((4-(tert-butyl)phenoxy)methyl)-1-tosyl-1H-indole ***4ab***

DEPT 135 NMR-spectrum ($CDCl_3$)

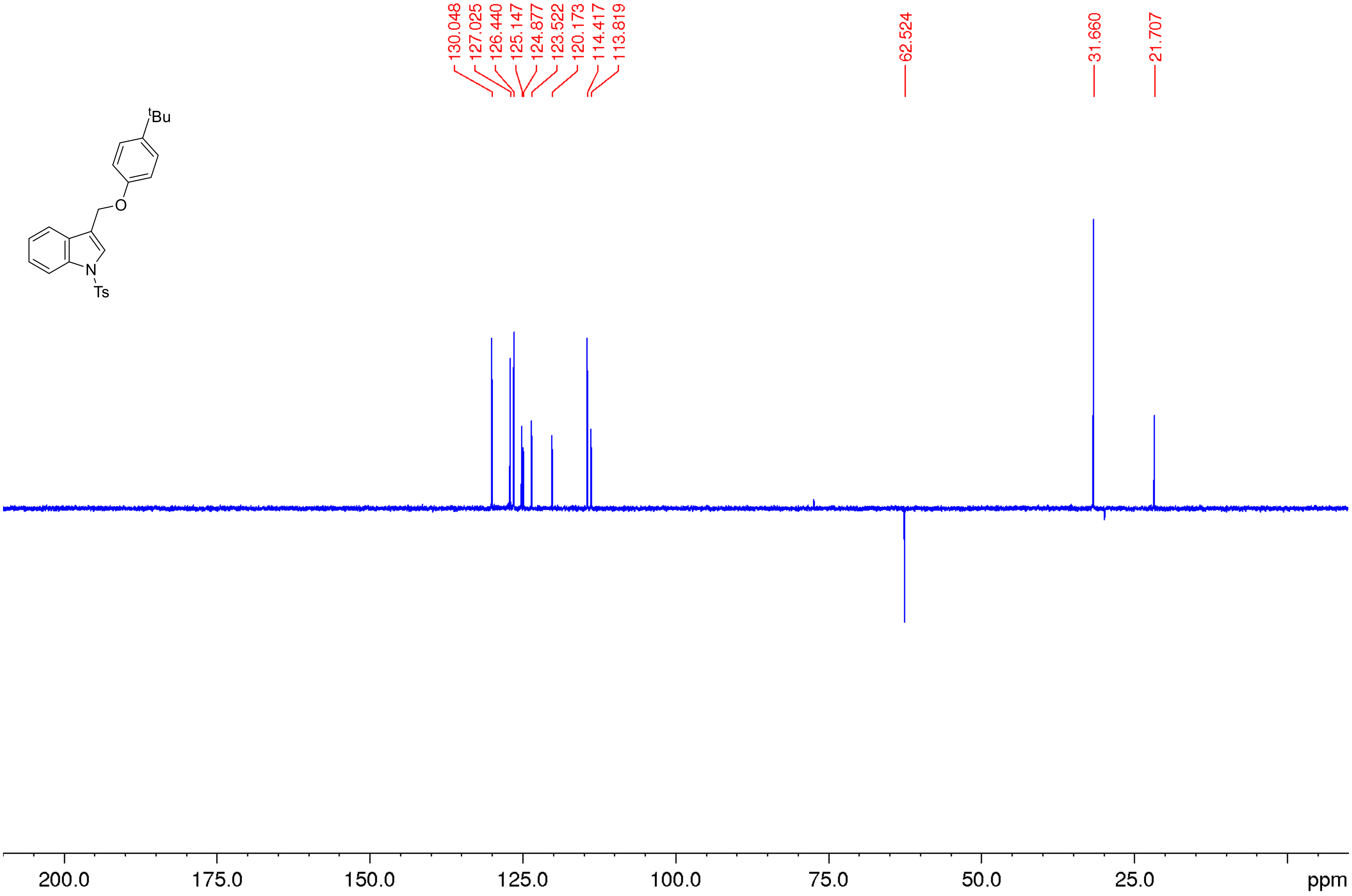

3-((4-fluorophenoxy)methyl)-1-tosyl-1H-indole ***4ac***

^{1}H NMR-spectrum (400.13 MHz) ($CDCl_3$)

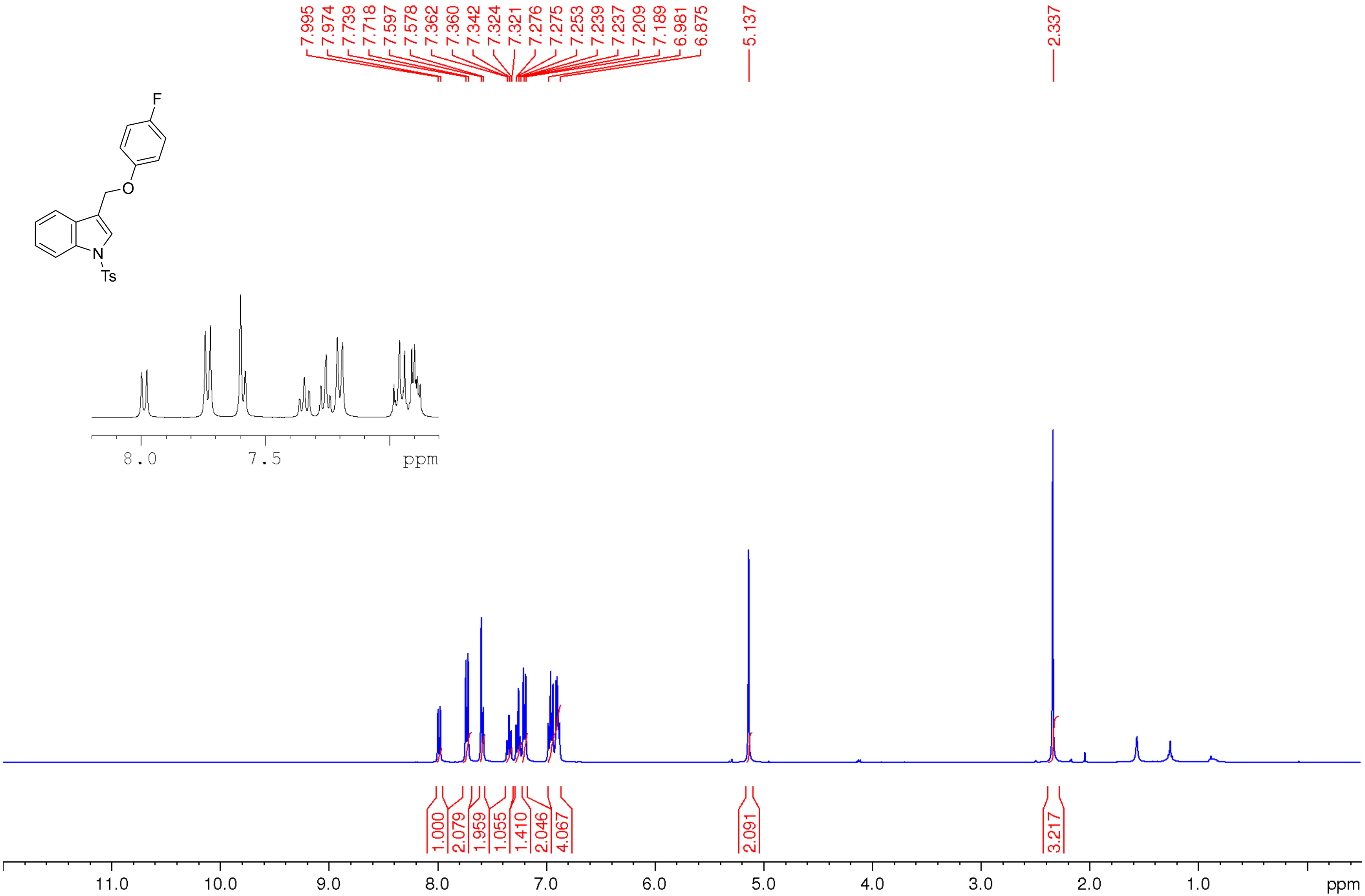

3-((4-fluorophenoxy)methyl)-1-tosyl-1H-indole ***4ac***

^{13}C NMR-spectrum (100.6 MHz) ($CDCl_3$)

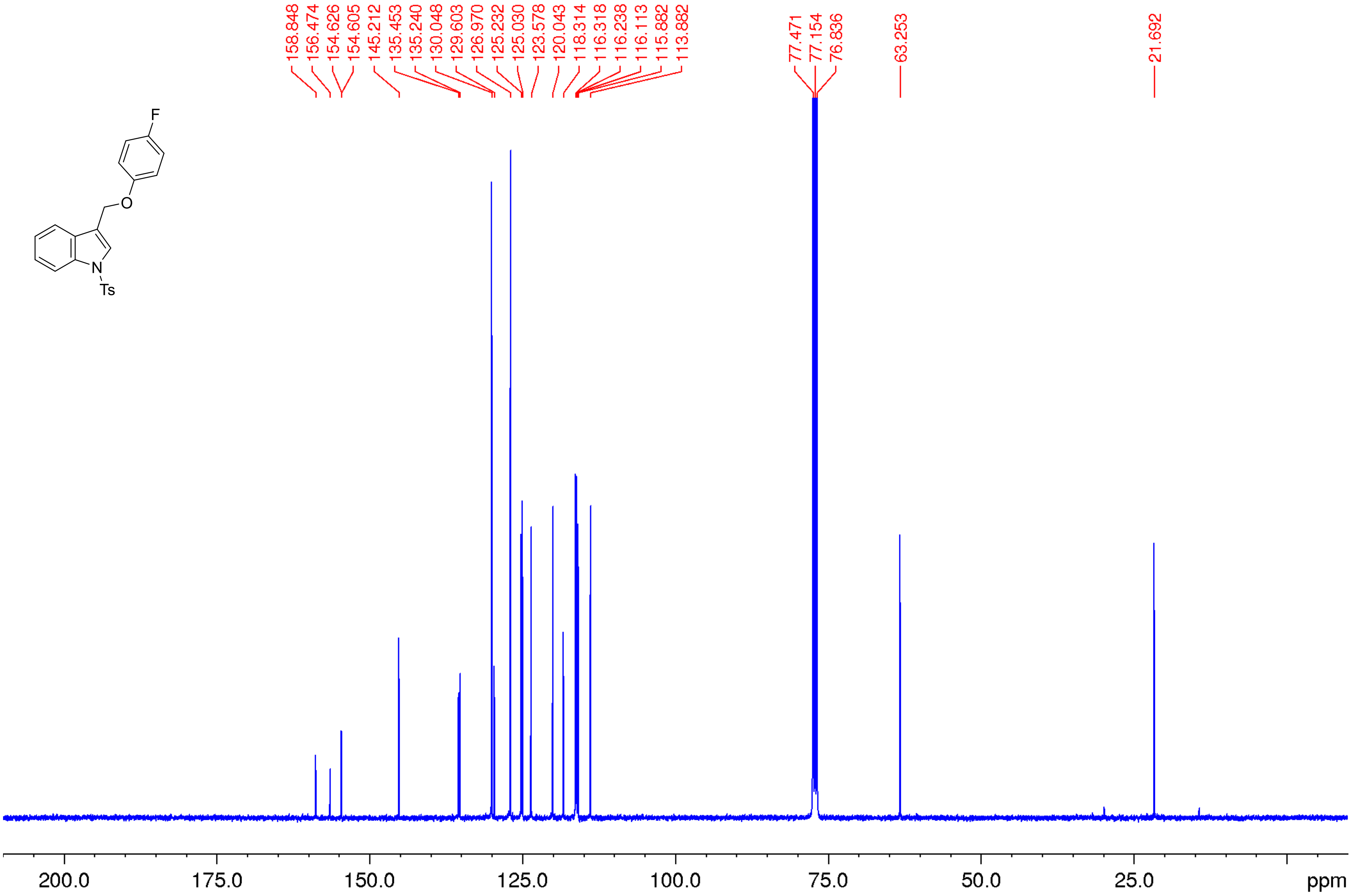

3-((4-fluorophenoxy)methyl)-1-tosyl-1H-indole ***4ac***

DEPT 135 NMR-spectrum ($CDCl_3$)

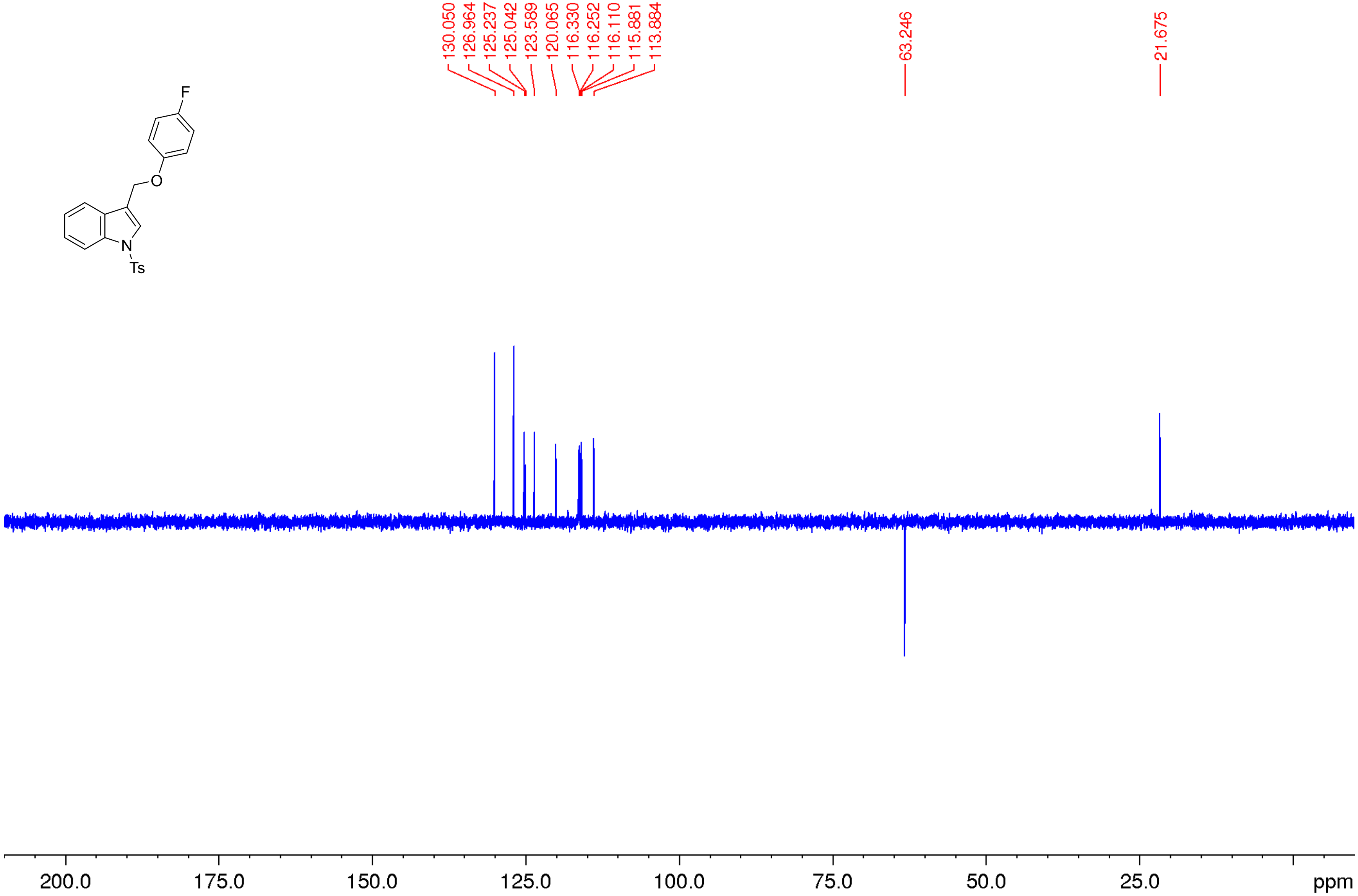

3-(([1,1'-biphenyl]-2-yloxy)methyl)-1-tosyl-1H-indole ***4ad***

^{1}H NMR-spectrum (400.13 MHz) ($CDCl_3$)

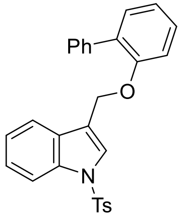

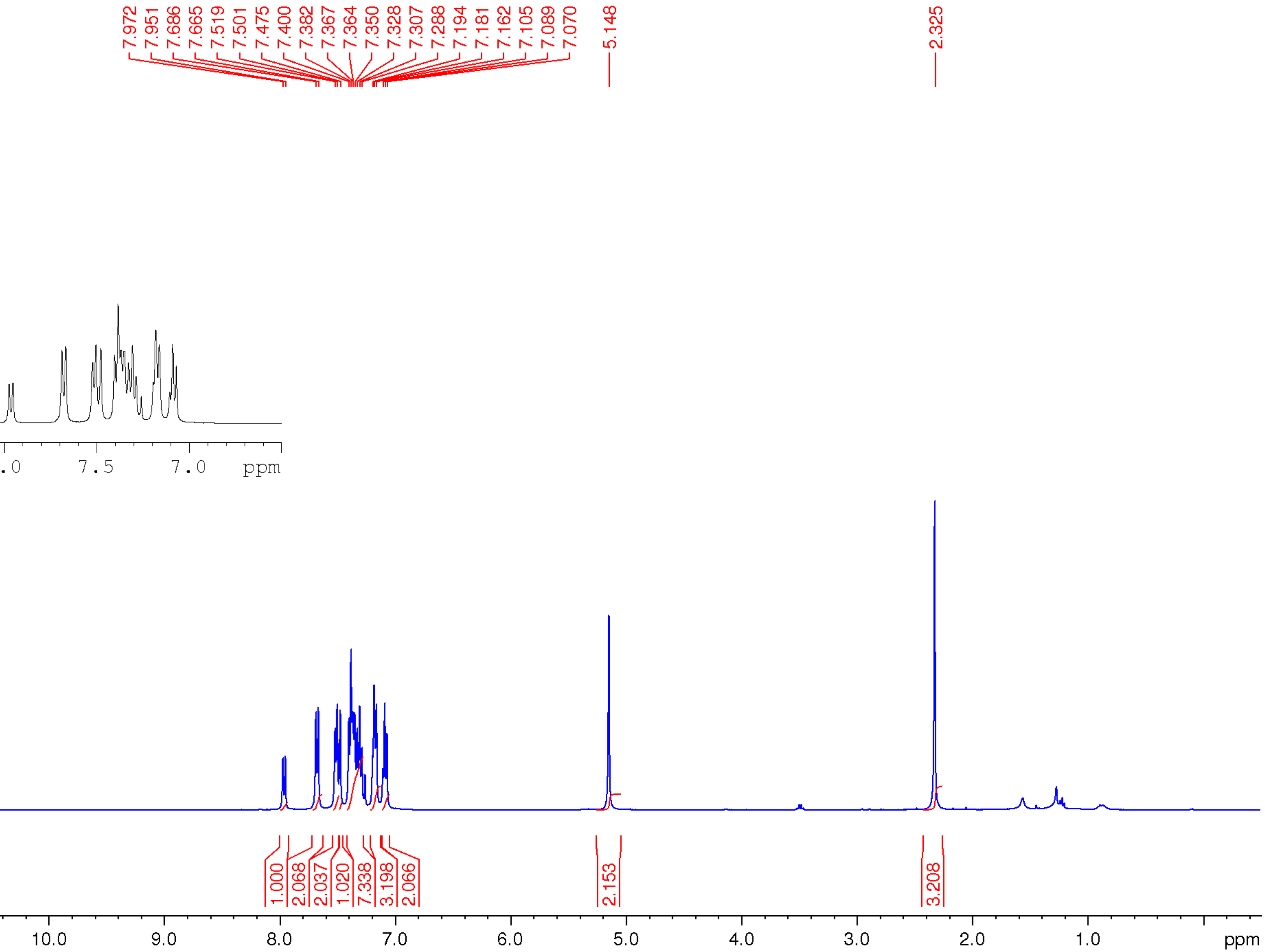

3-(([1,1'-biphenyl]-2-yloxy)methyl)-1-tosyl-1H-indole ***4ad***

^{13}C NMR-spectrum (100.6 MHz) ($CDCl_3$)

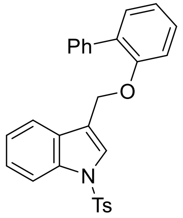

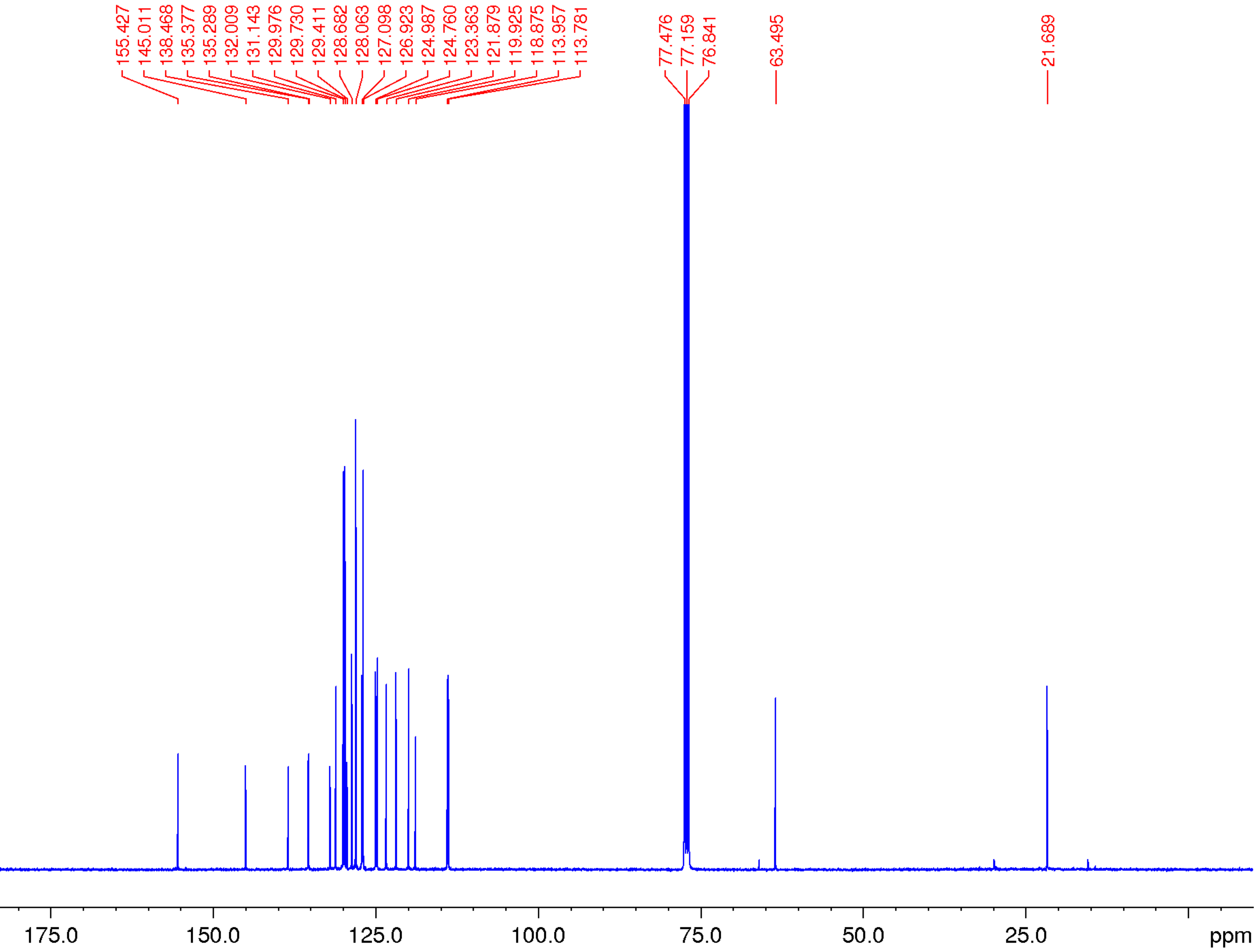

3-(([1,1'-biphenyl]-2-yloxy)methyl)-1-tosyl-1H-indole **4ad**

DEPT 135 NMR-spectrum ($CDCl_3$)

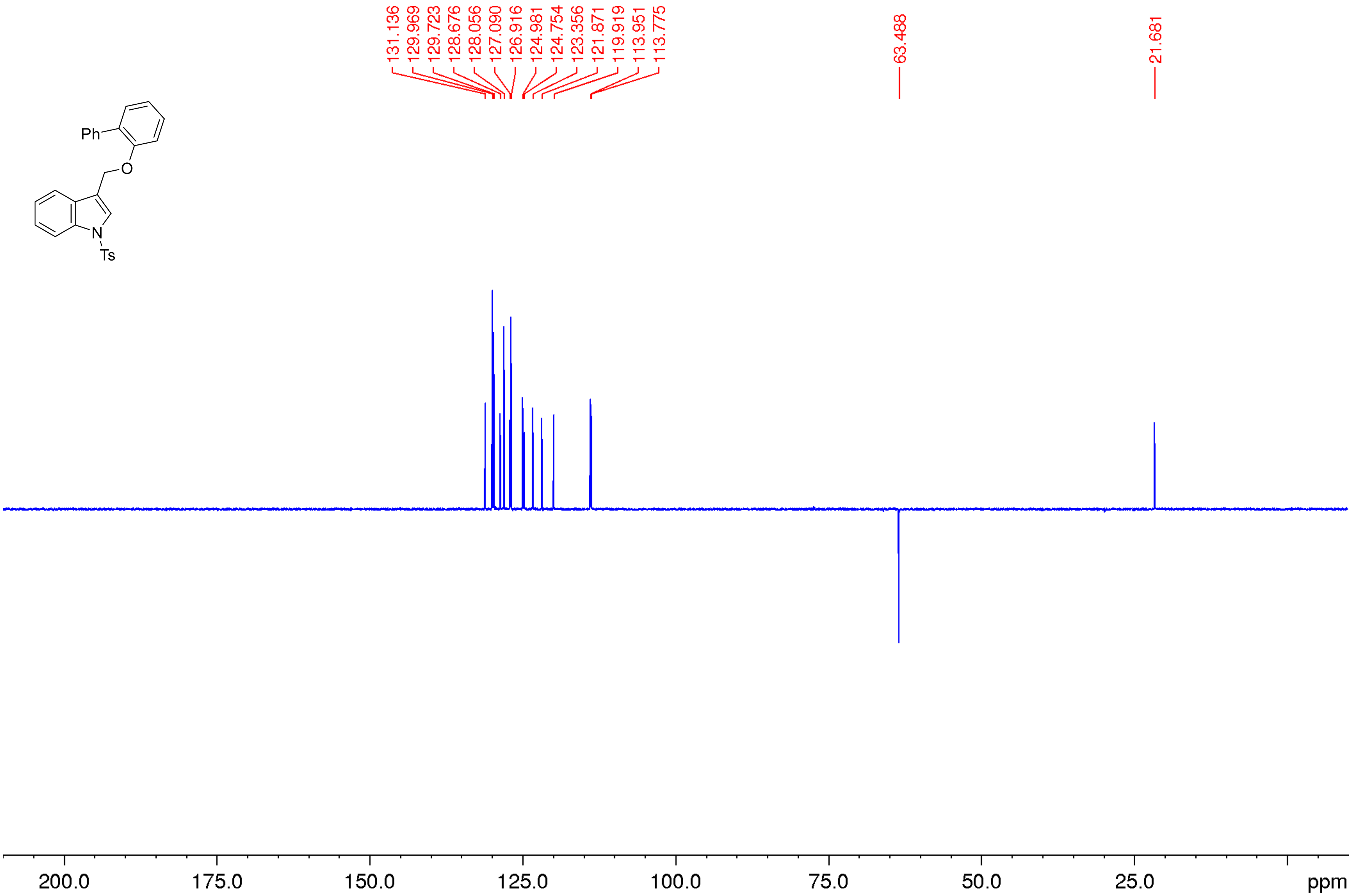

methyl 3-((1-tosyl-1H-indol-3-yl)methoxy)benzoate ***4ae***

^{1}H NMR-spectrum (400.13 MHz) ($CDCl_3$)

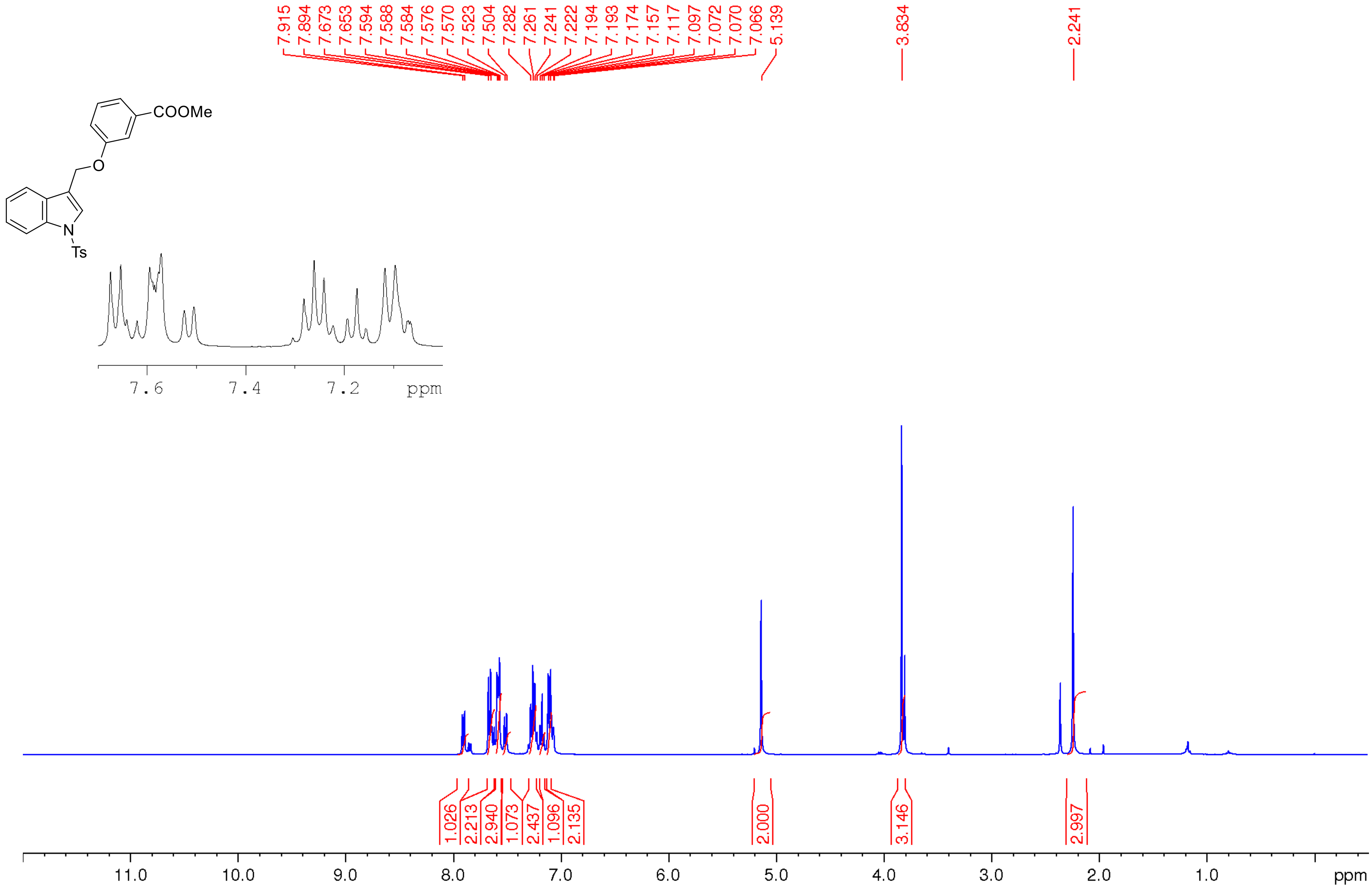

methyl 3-((1-tosyl-1H-indol-3-yl)methoxy)benzoate ***4ae***

^{13}C NMR-spectrum (100.6 MHz) ($CDCl_3$)

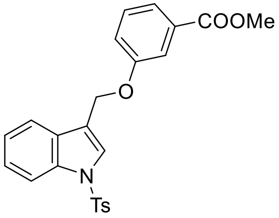

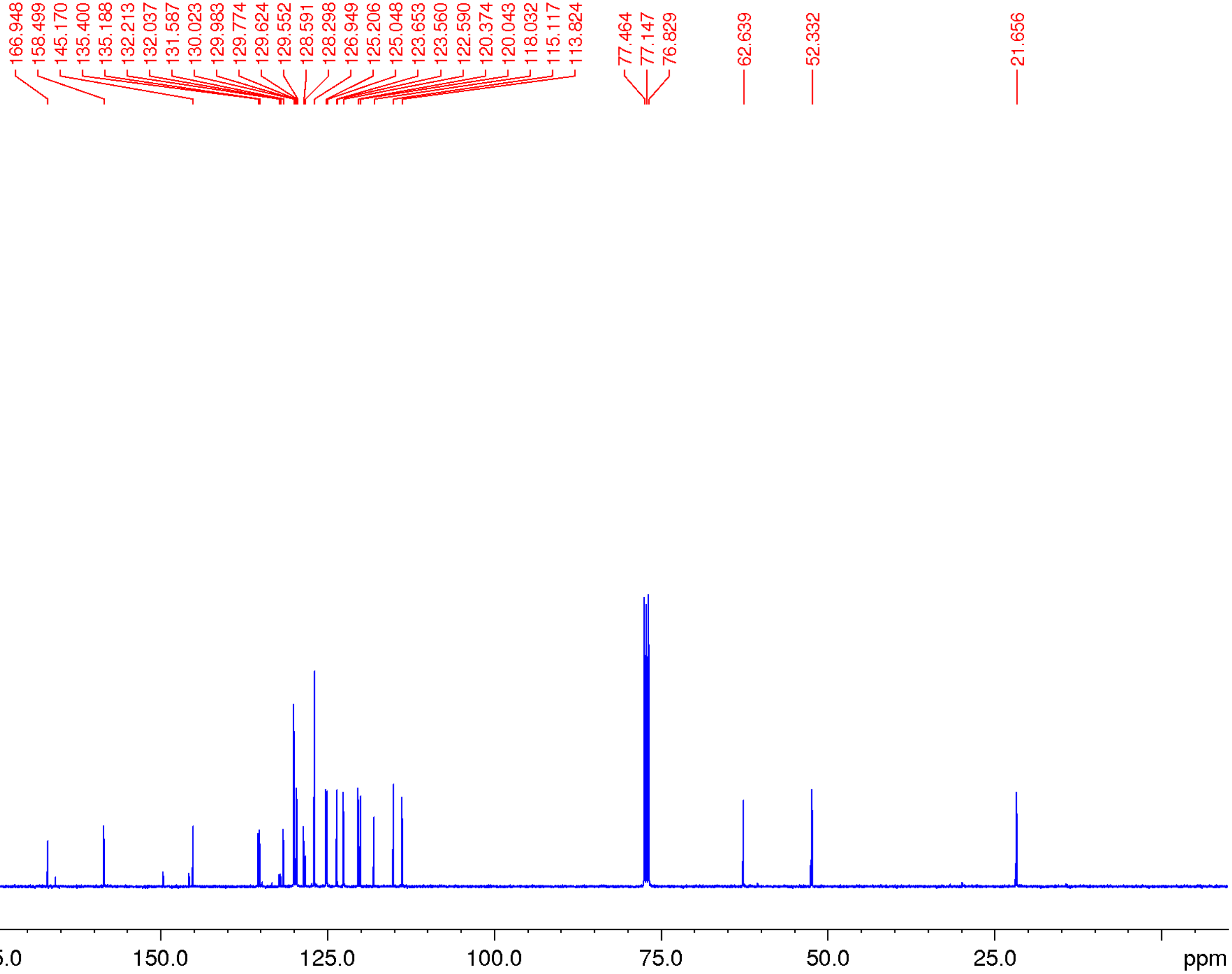

methyl 3-((1-tosyl-1H-indol-3-yl)methoxy)benzoate ***4ae***

DEPT 135 NMR-spectrum ($CDCl_3$)

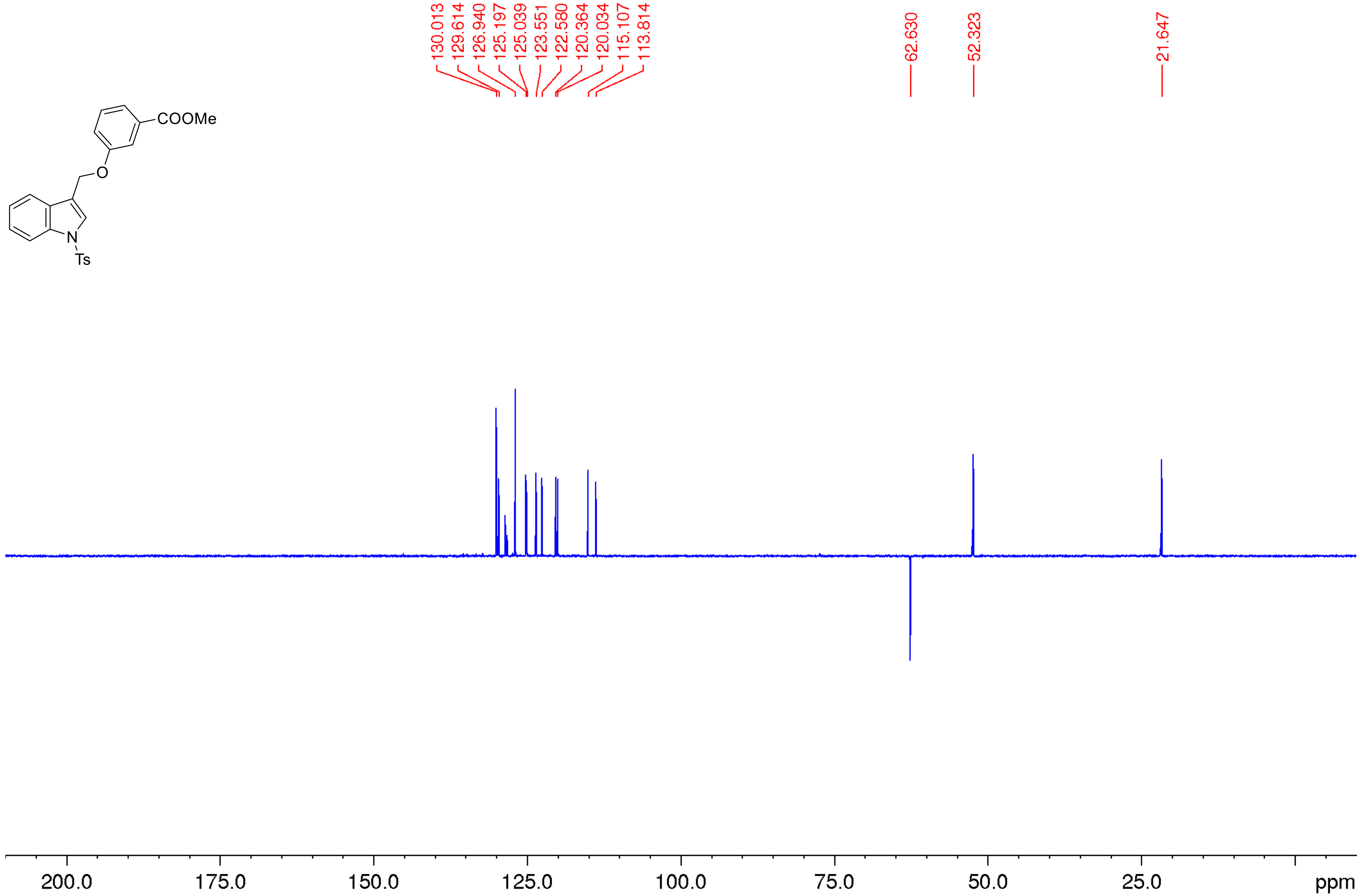

*4-((1-tosyl-1H-indol-3-yl)methoxy)benzonitrile **4af***

^{1}H NMR-spectrum (400.13 MHz) ($CDCl_3$)

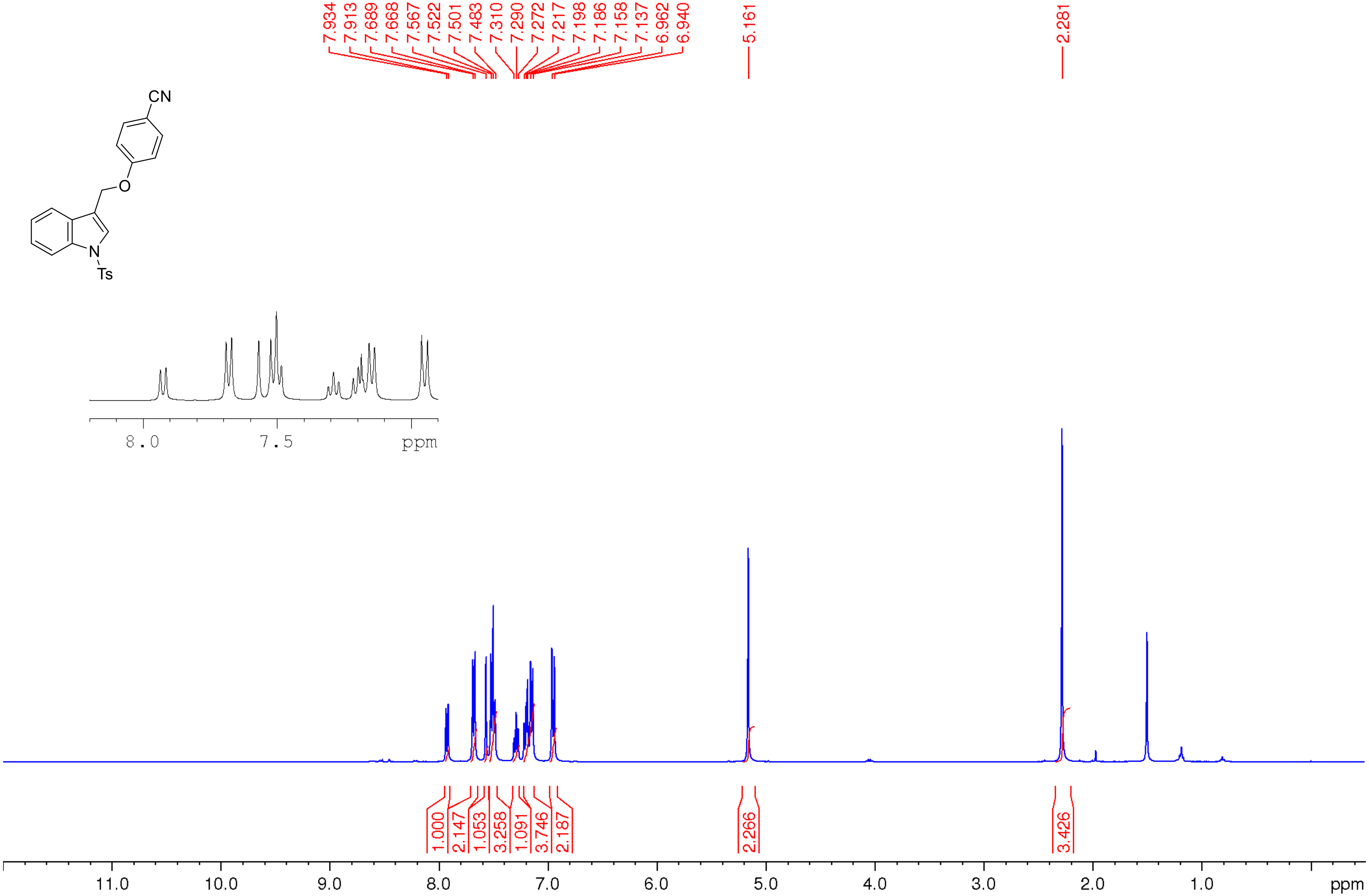

4-((1-tosyl-1H-indol-3-yl)methoxy)benzonitrile ***4af***

^{13}C NMR-spectrum (100.6 MHz) ($CDCl_3$)

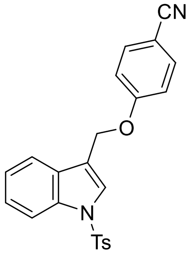

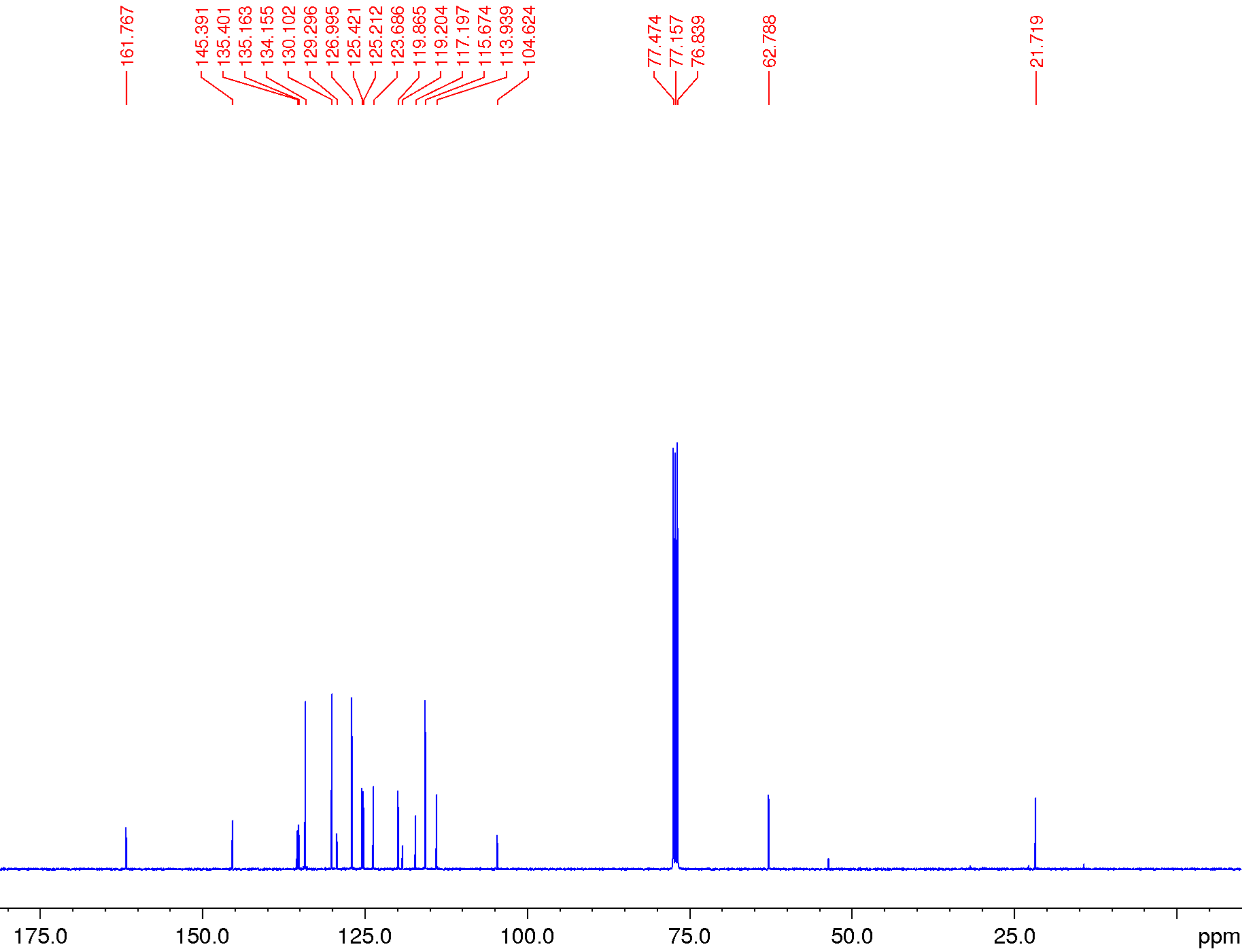

4-((1-tosyl-1H-indol-3-yl)methoxy)benzonitrile ***4af***

DEPT 135 NMR-spectrum ($CDCl_3$)

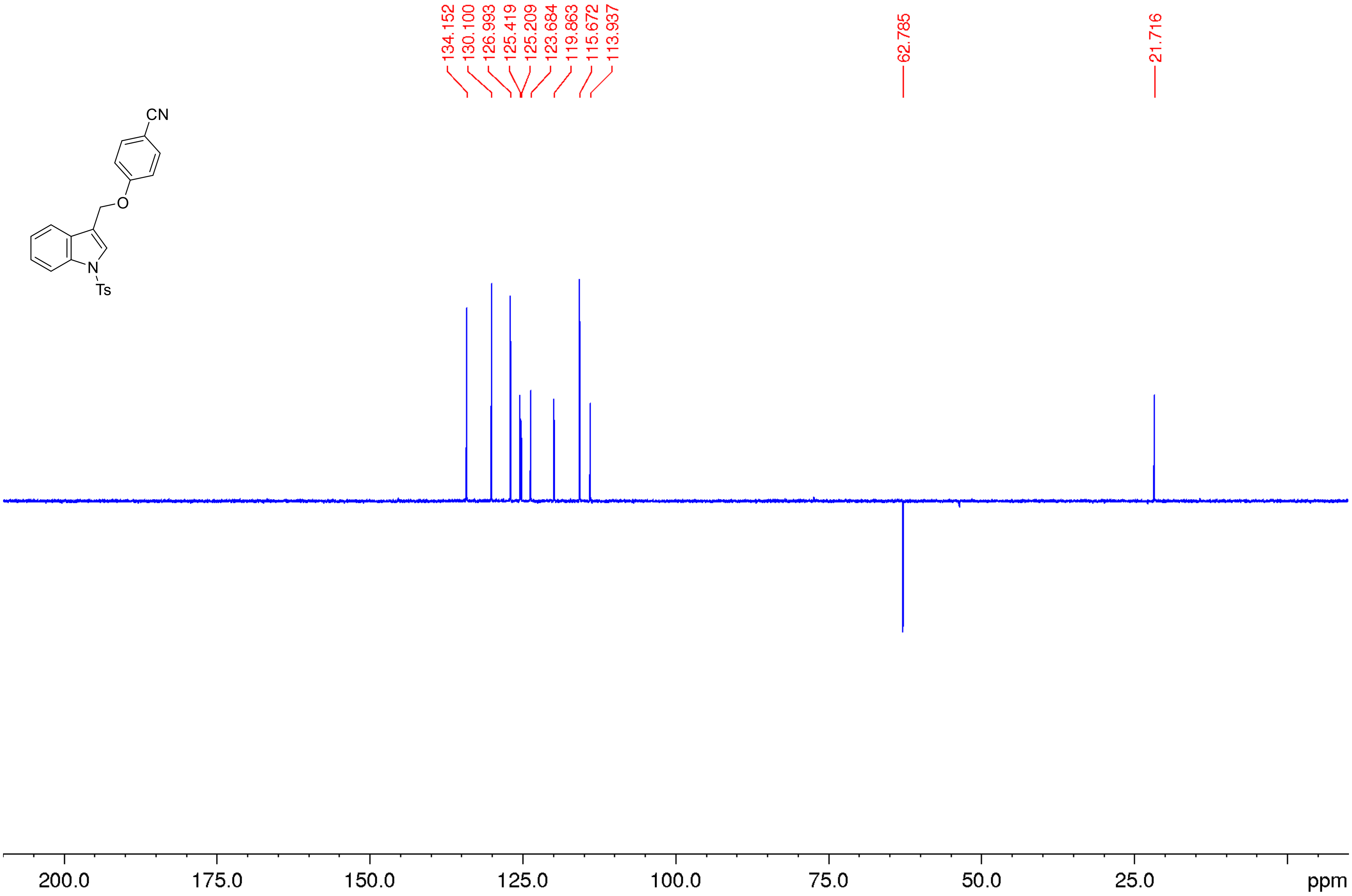

5-methoxy-3-((4-methoxyphenoxy)methyl)-1-tosyl-1H-indole ***4da***

^{1}H NMR-spectrum (400.13 MHz) ($CDCl_3$)

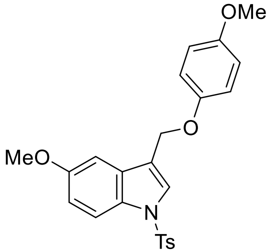

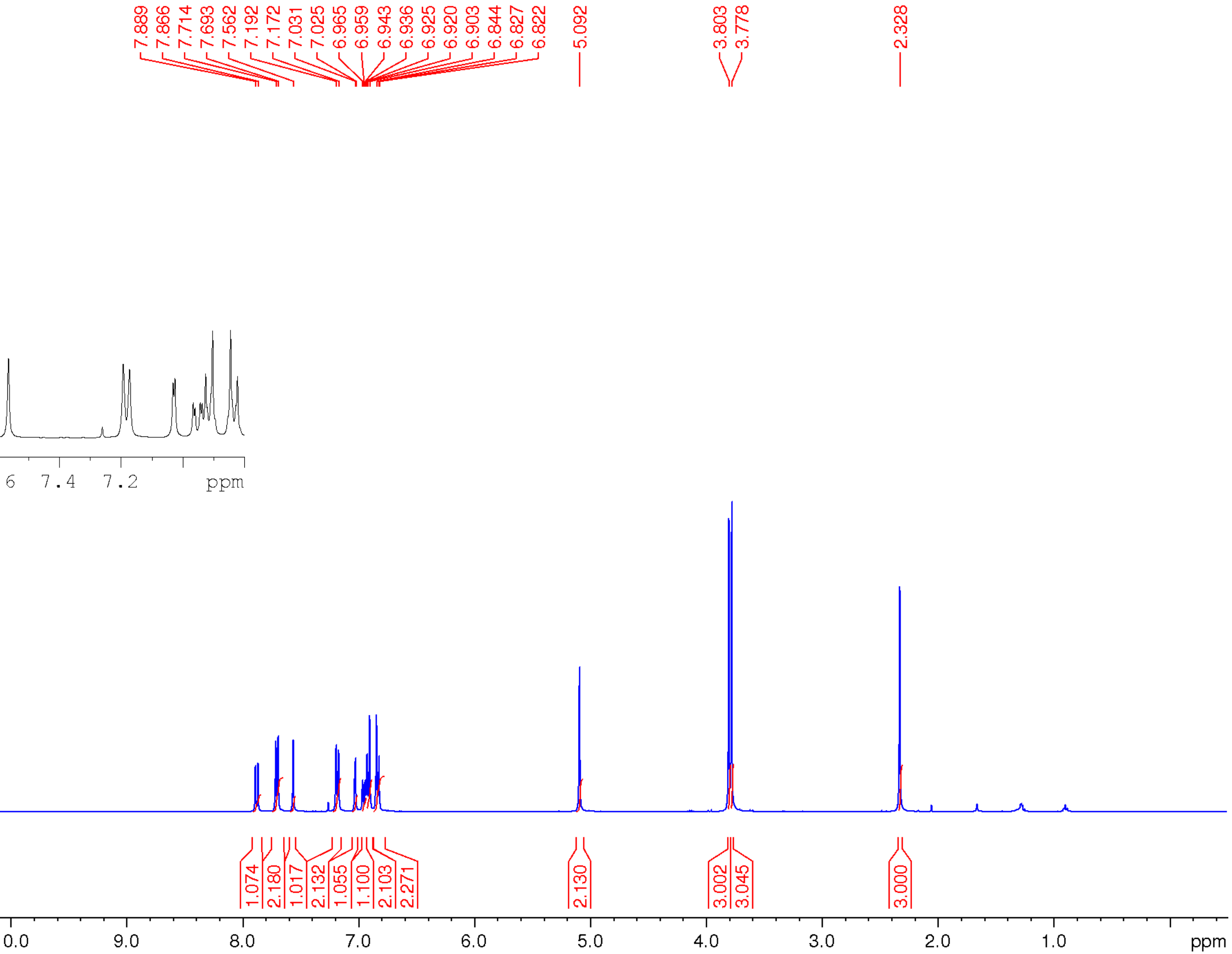

5-methoxy-3-((4-methoxyphenoxy)methyl)-1-tosyl-1H-indole ***4da***

^{13}C NMR-spectrum (100.6 MHz) ($CDCl_3$)

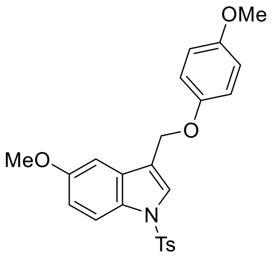

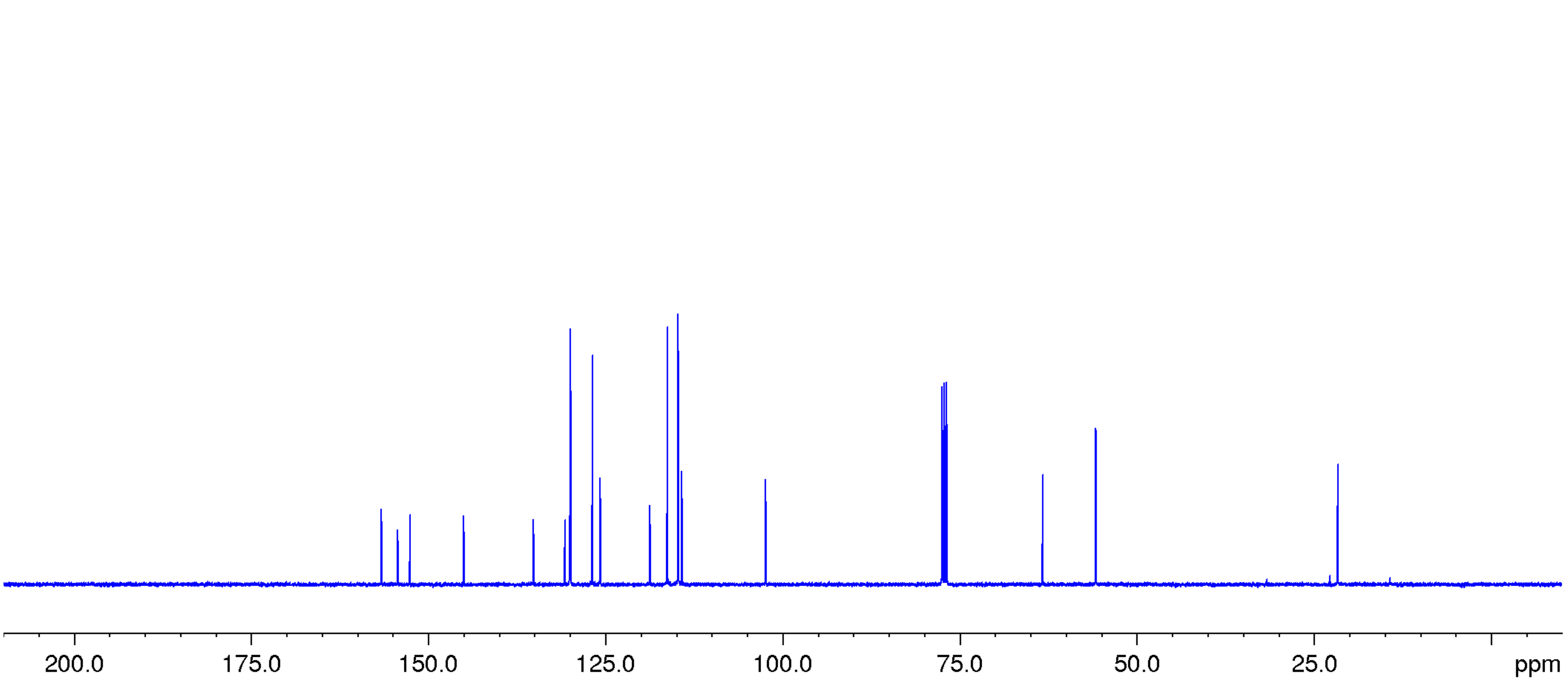

*5-methoxy-3-((4-methoxyphenoxy)methyl)-1-tosyl-1H-indole **4da***

DEPT 135 NMR-spectrum ($CDCl_3$)

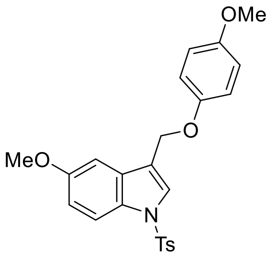

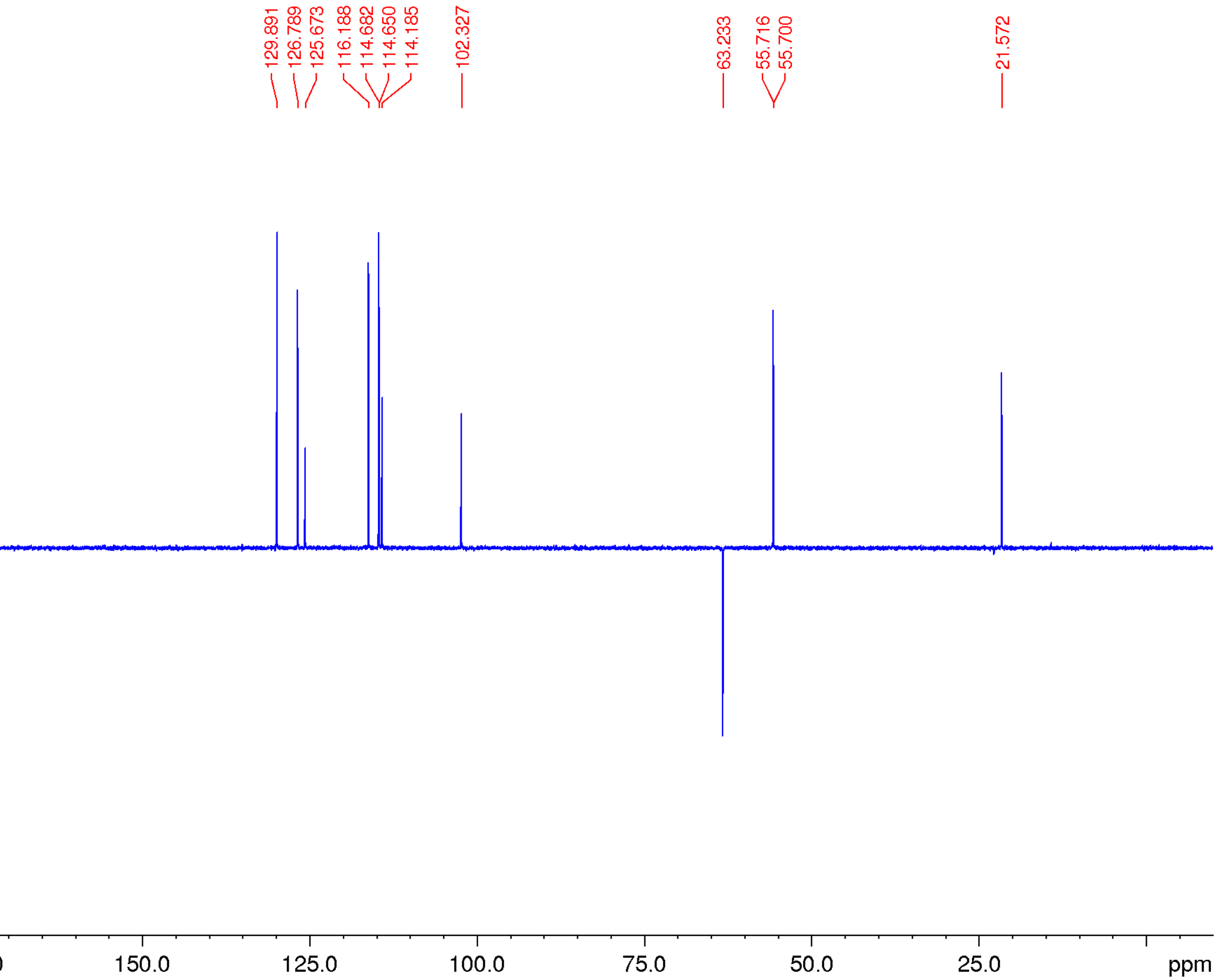

3-((4-(tert-butyl)phenoxy)methyl)-5-methoxy-1-tosyl-1H-indole **4db** ^{1}H NMR-spectrum (400.13 MHz) ($CDCl_3$)

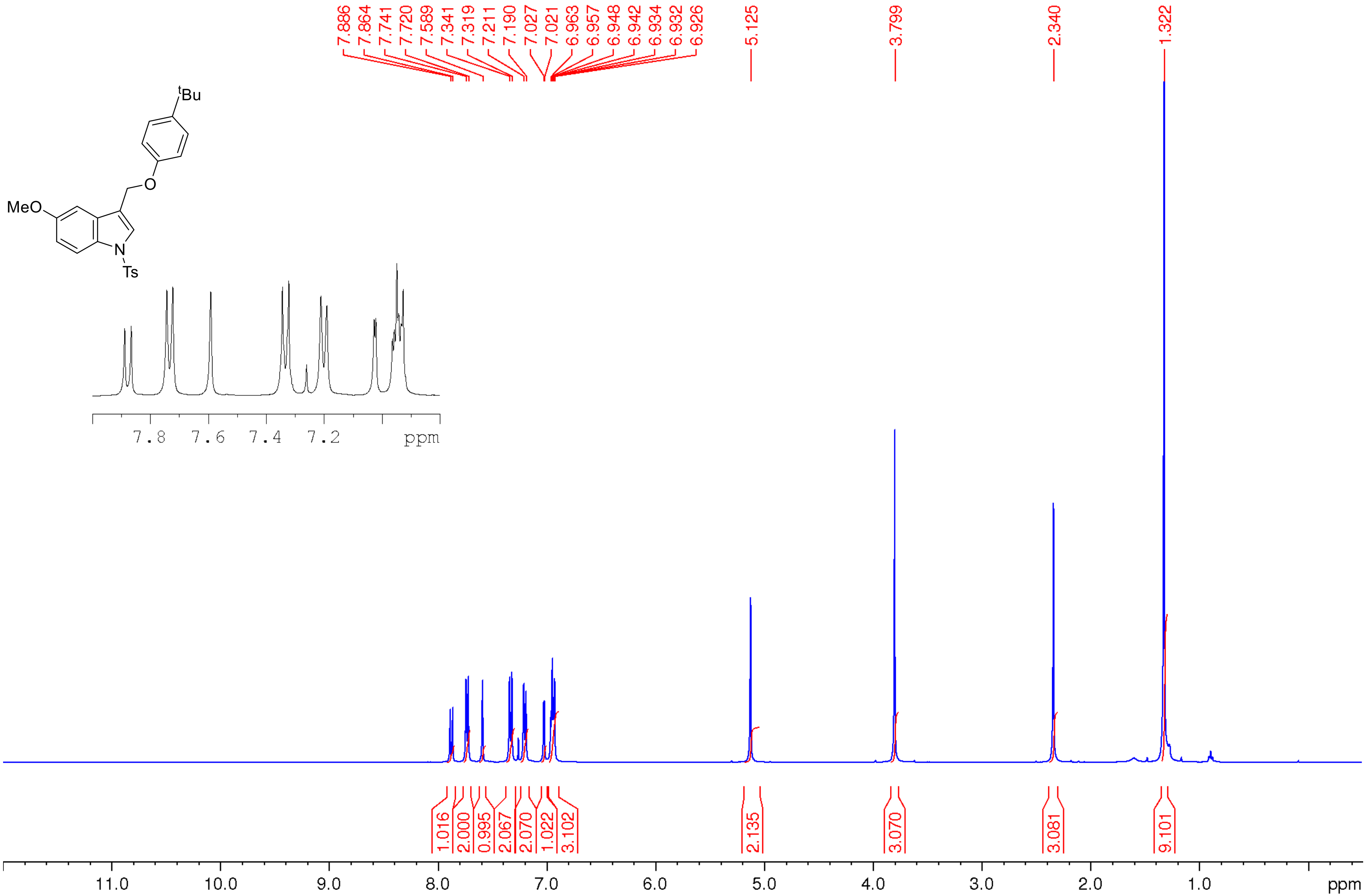

3-((4-(tert-butyl)phenoxy)methyl)-5-methoxy-1-tosyl-1H-indole ***4db*** ^{13}C NMR-spectrum (100.6 MHz) ($CDCl_3$)

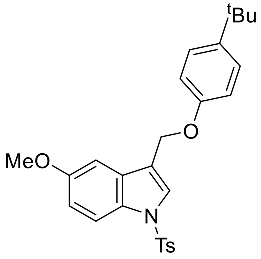

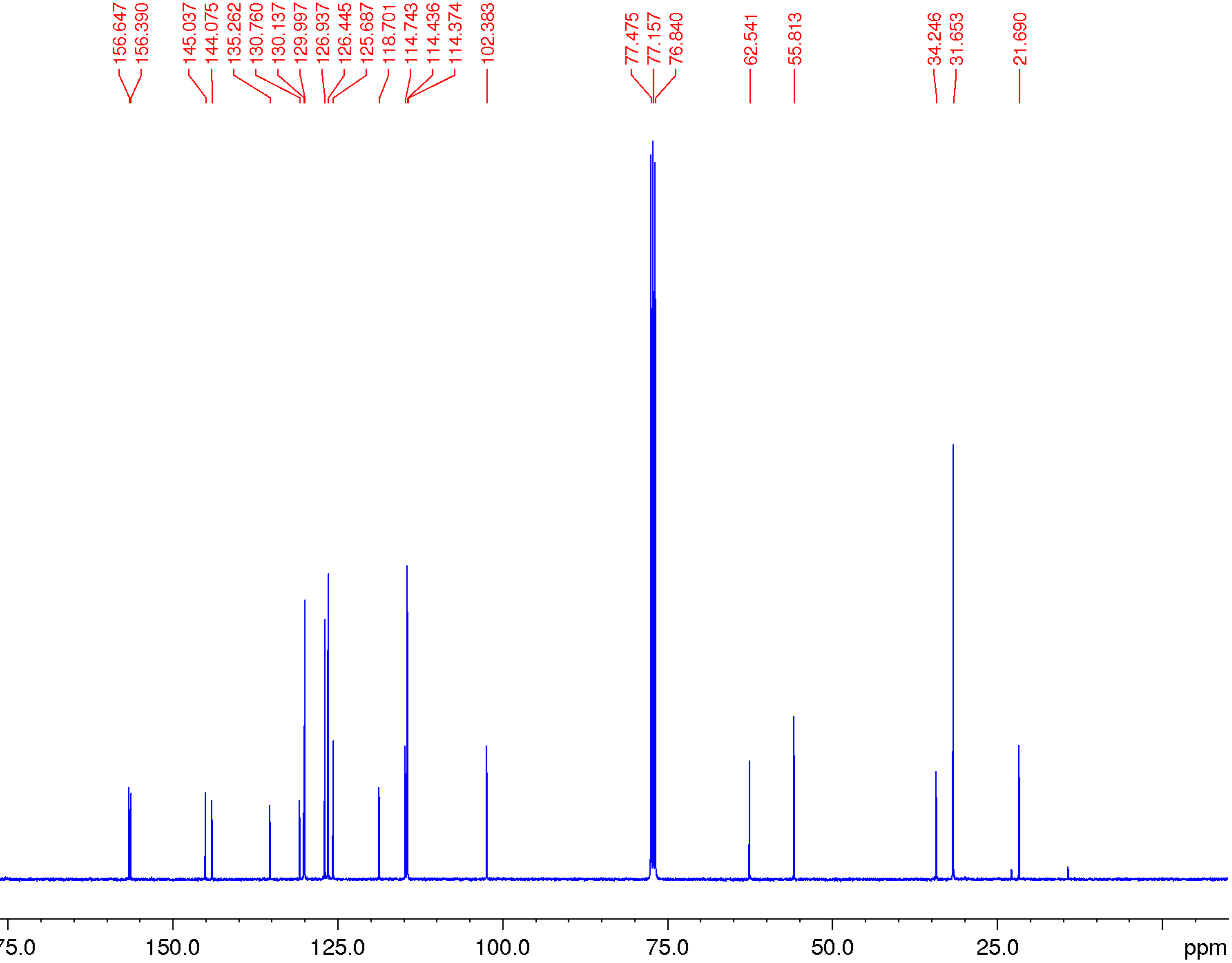

3-((4-(tert-butyl)phenoxy)methyl)-5-methoxy-1-tosyl-1H-indole ***4db*** DEPT 135 NMR-spectrum ($CDCl_3$)

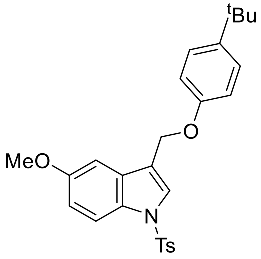

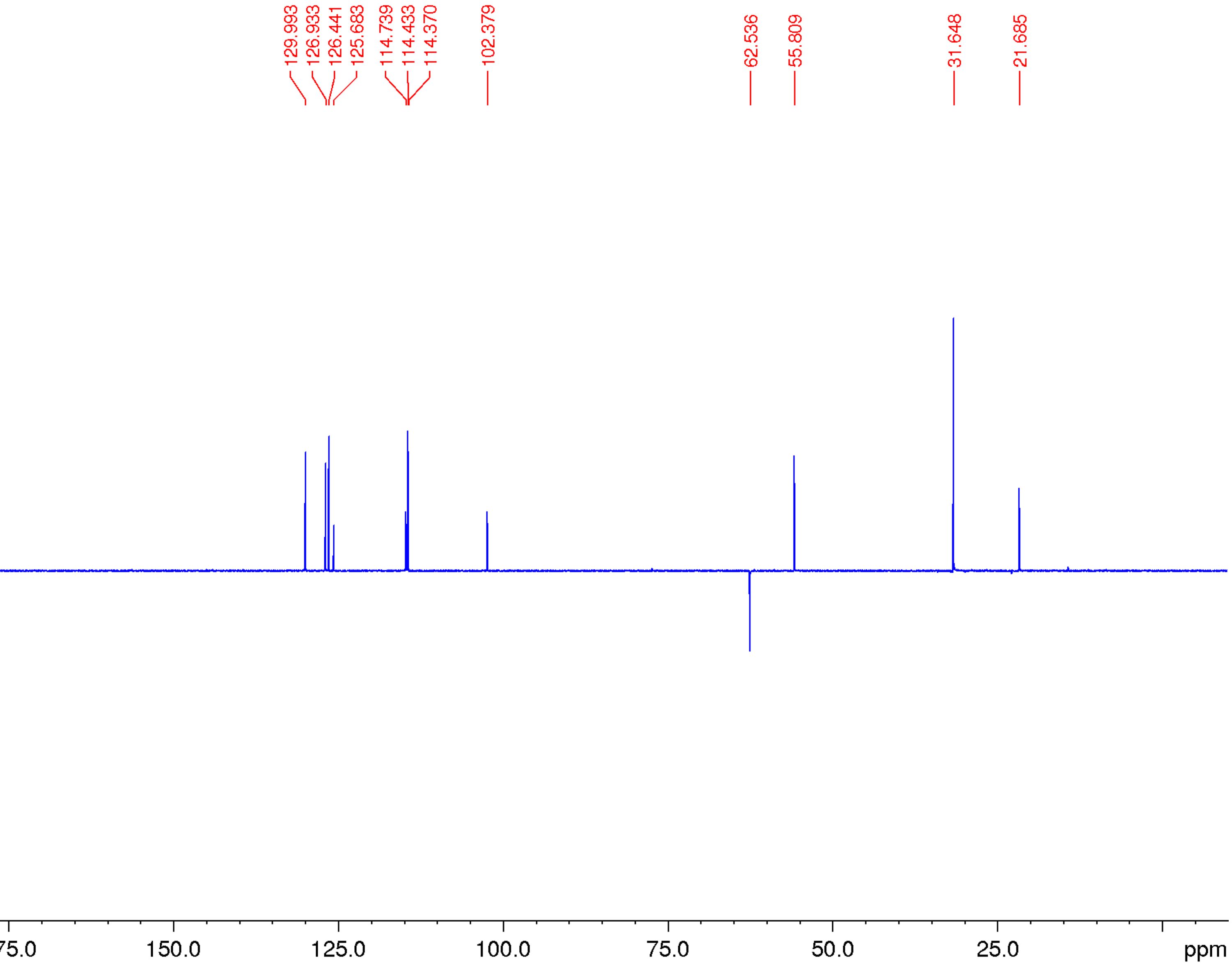

3-(([1,1'-biphenyl]-2-yloxy)methyl)-5-methoxy-1-tosyl-1H-indole ***4dc*** ^{1}H NMR-spectrum (400.13 MHz) ($CDCl_3$)

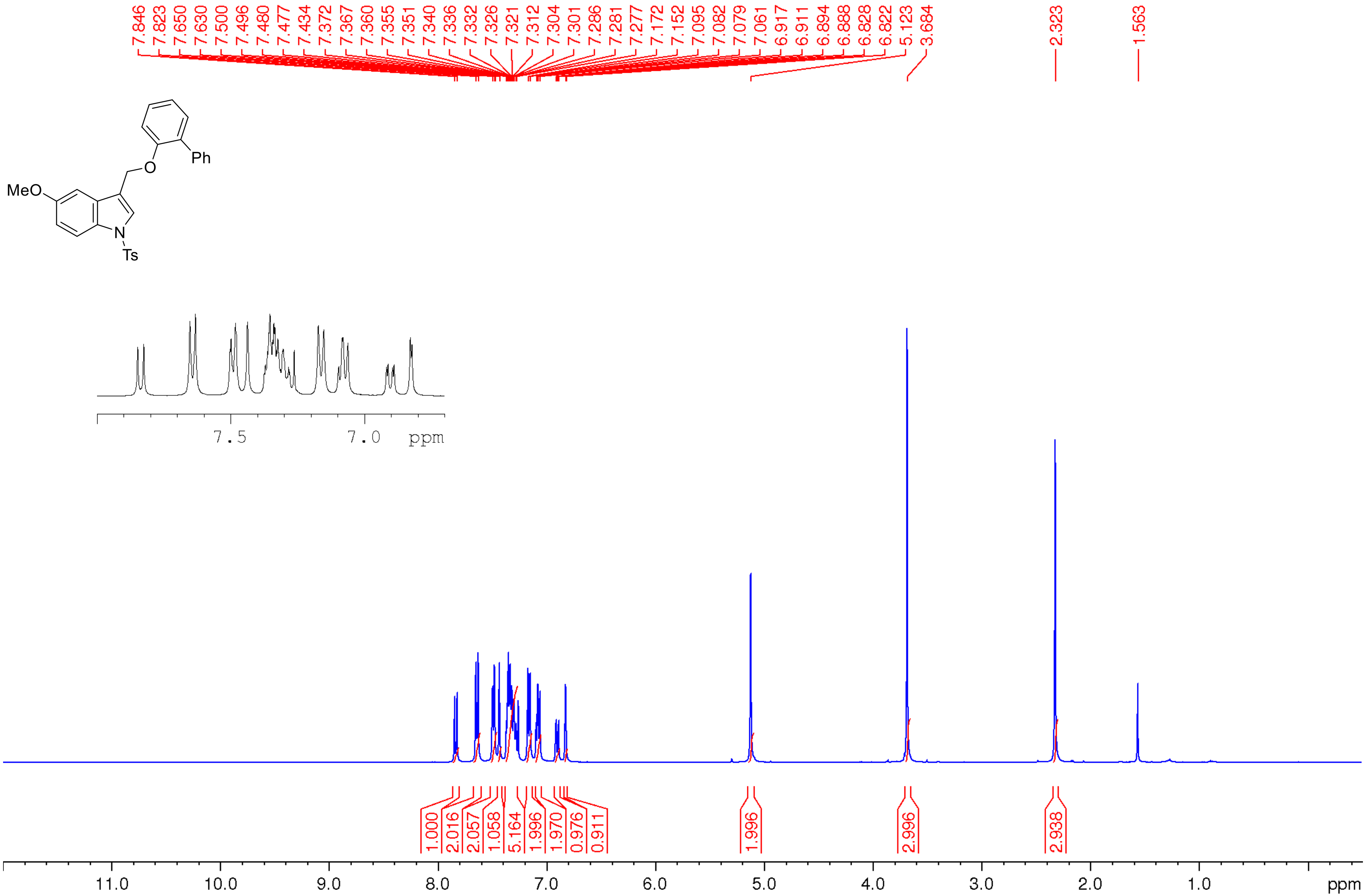

3-(([1,1'-biphenyl]-2-yloxy)methyl)-5-methoxy-1-tosyl-1H-indole ***4dc*** ^{13}C NMR-spectrum (100.6 MHz) ($CDCl_3$)

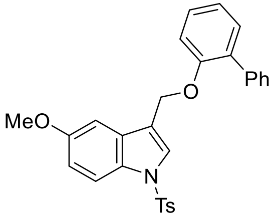

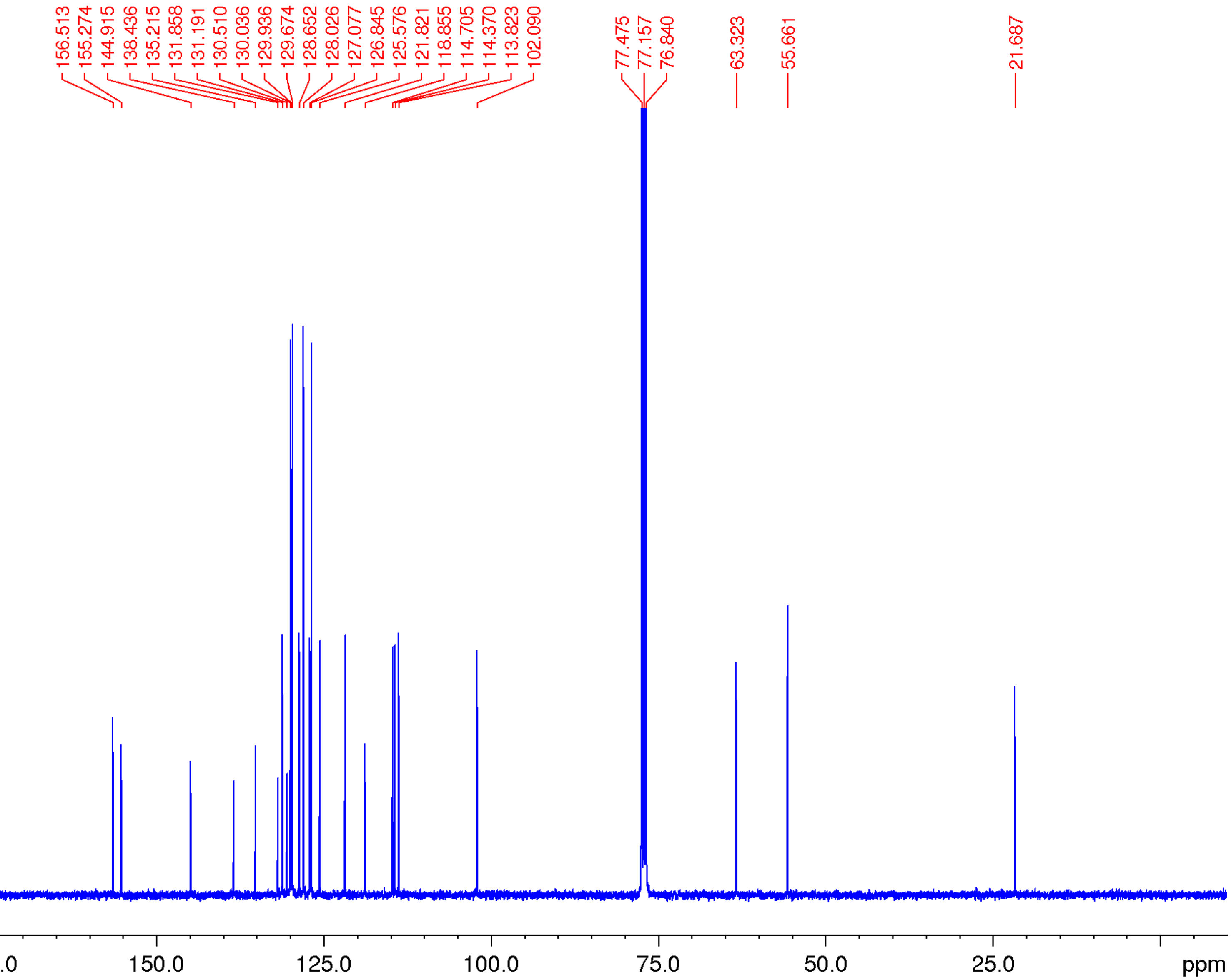

3-(([1,1'-biphenyl]-2-yloxy)methyl)-5-methoxy-1-tosyl-1H-indole ***4dc*** DEPT 135 NMR-spectrum ($CDCl_3$)

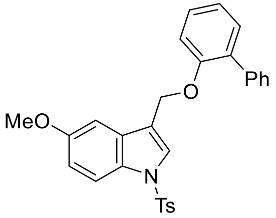

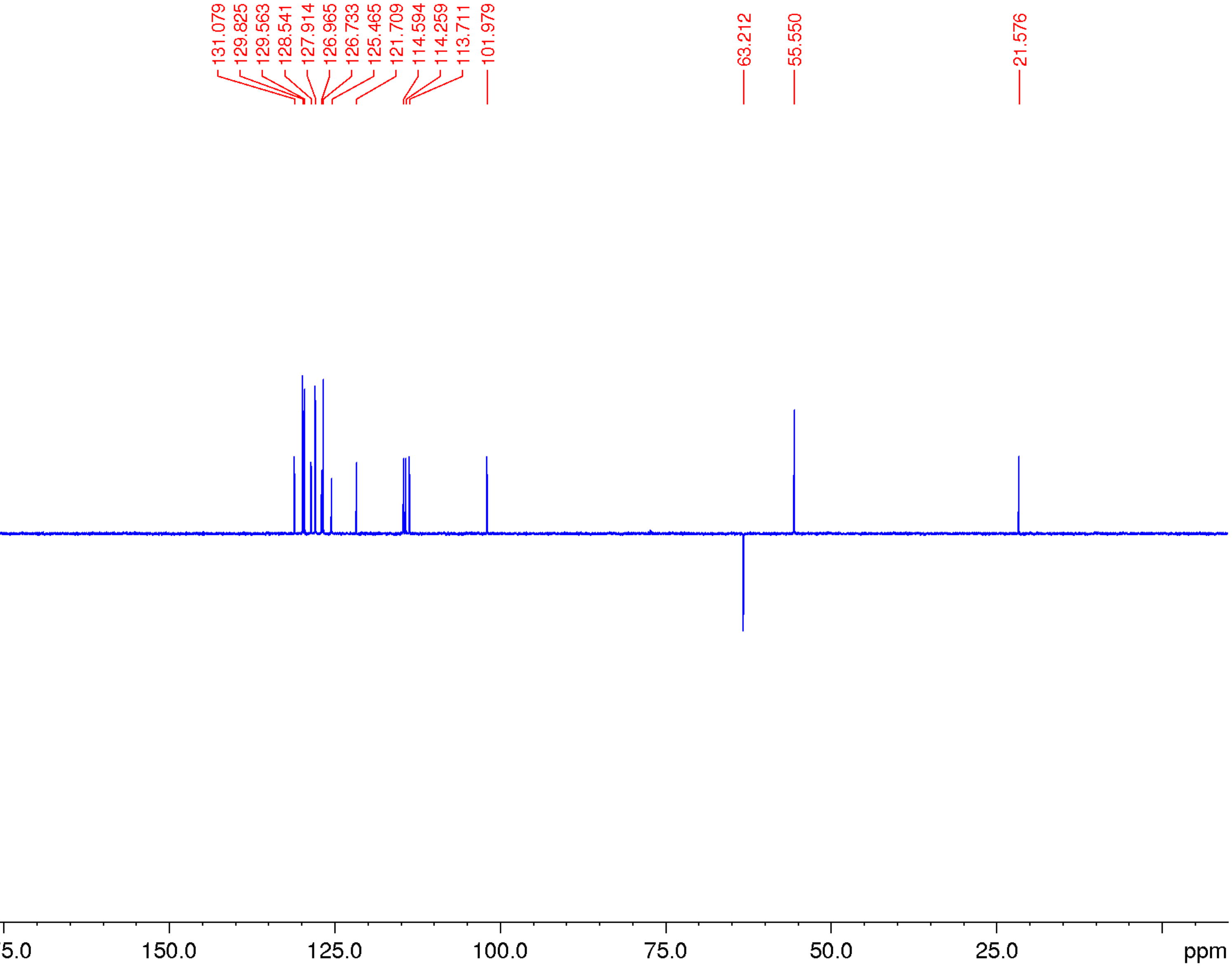

methyl 3-((5-methoxy-1-tosyl-1H-indol-3-yl)methoxy)benzoate ***4de***

^{1}H NMR-spectrum (400.13 MHz) ($CDCl_3$)

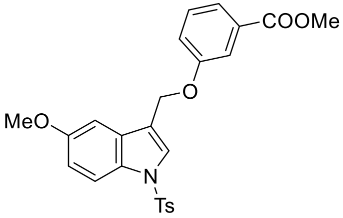

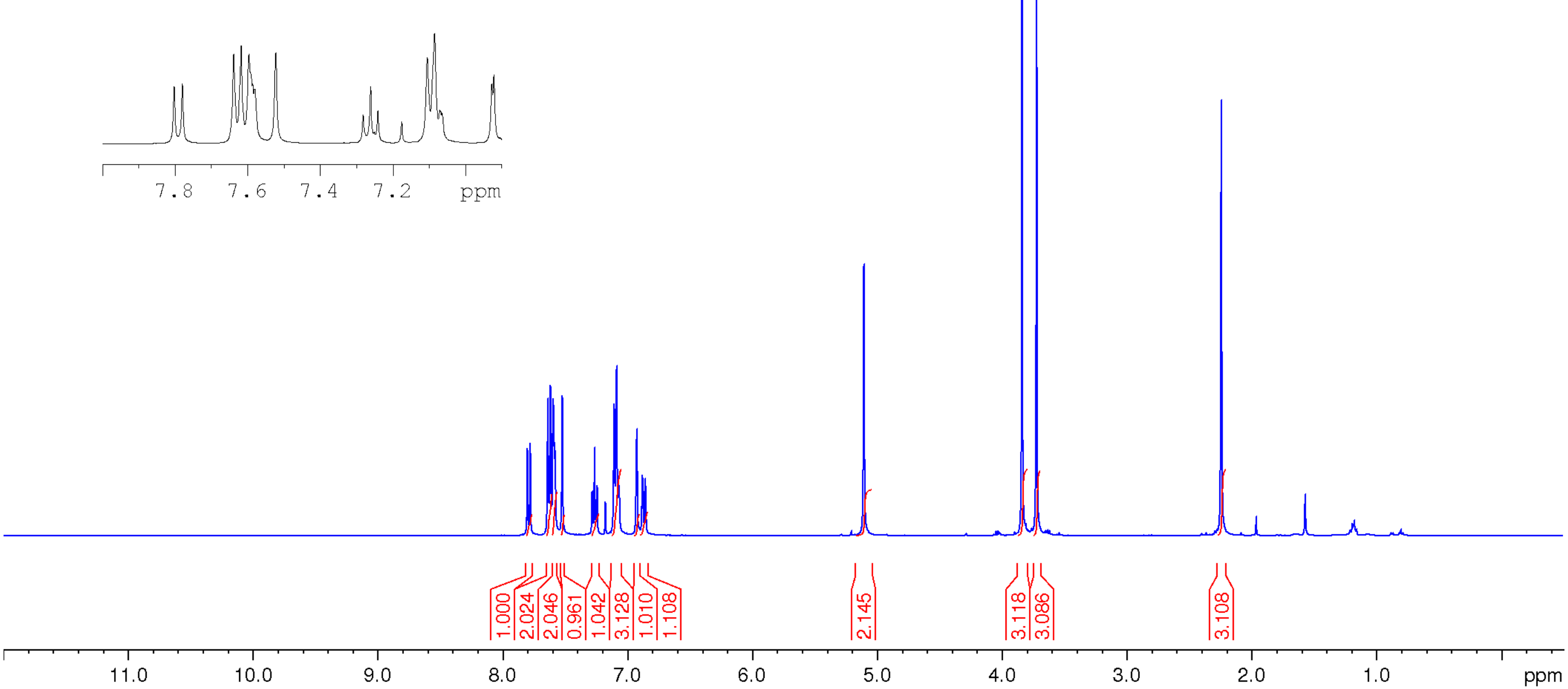

methyl 3-((5-methoxy-1-tosyl-1H-indol-3-yl)methoxy)benzoate ***4de***

^{13}C NMR-spectrum (100.6 MHz) ($CDCl_3$)

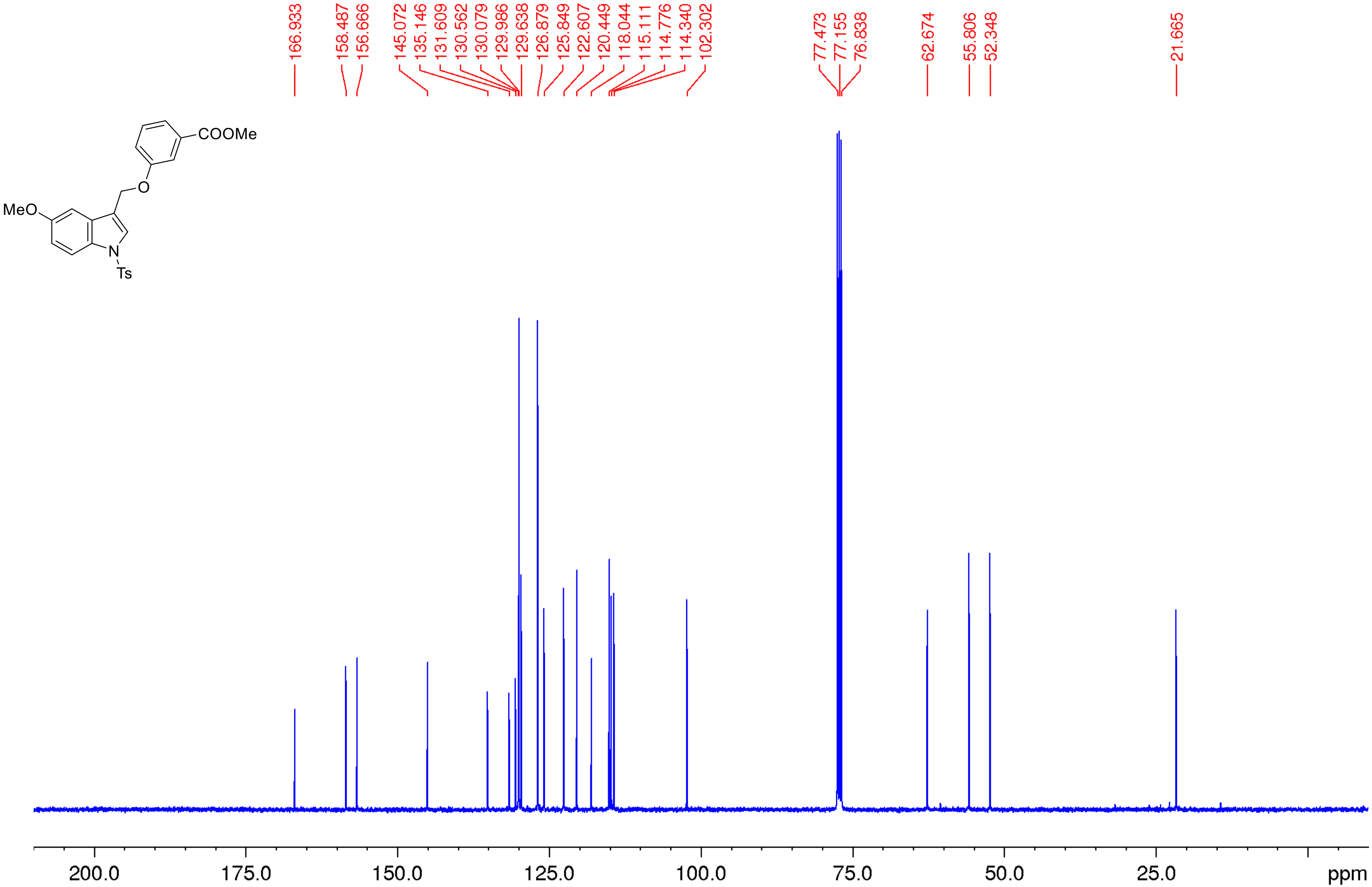

methyl 3-((5-methoxy-1-tosyl-1H-indol-3-yl)methoxy)benzoate ***4de*** DEPT 135 NMR-spectrum ($CDCl_3$)

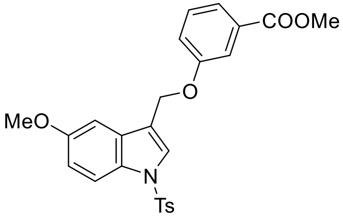

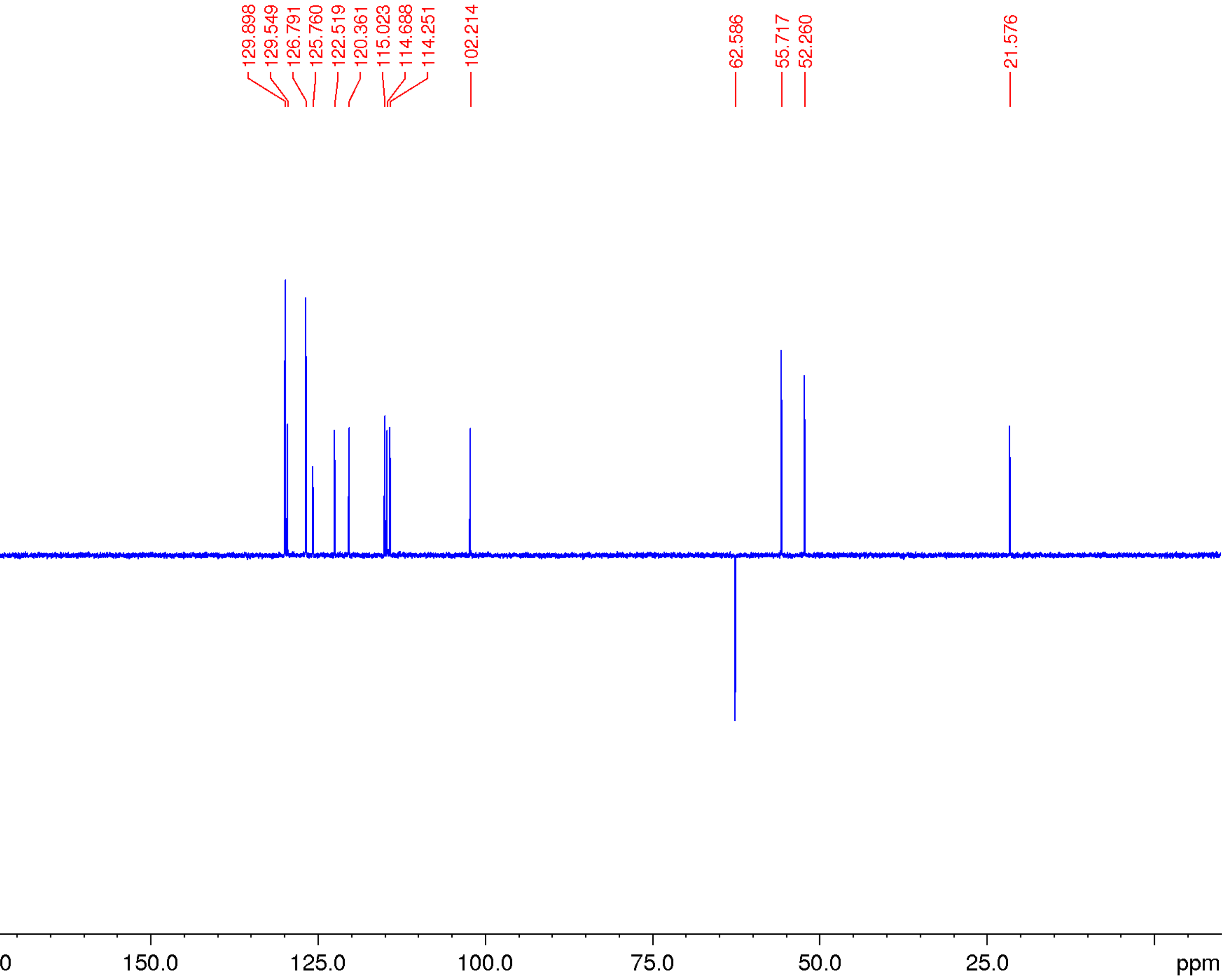

3-(([1,1'-biphenyl]-2-yloxy)methyl)-5-phenyl-1-tosyl-1H-indole ***4ed***

^{1}H NMR-spectrum (400.13 MHz) ($CDCl_3$)

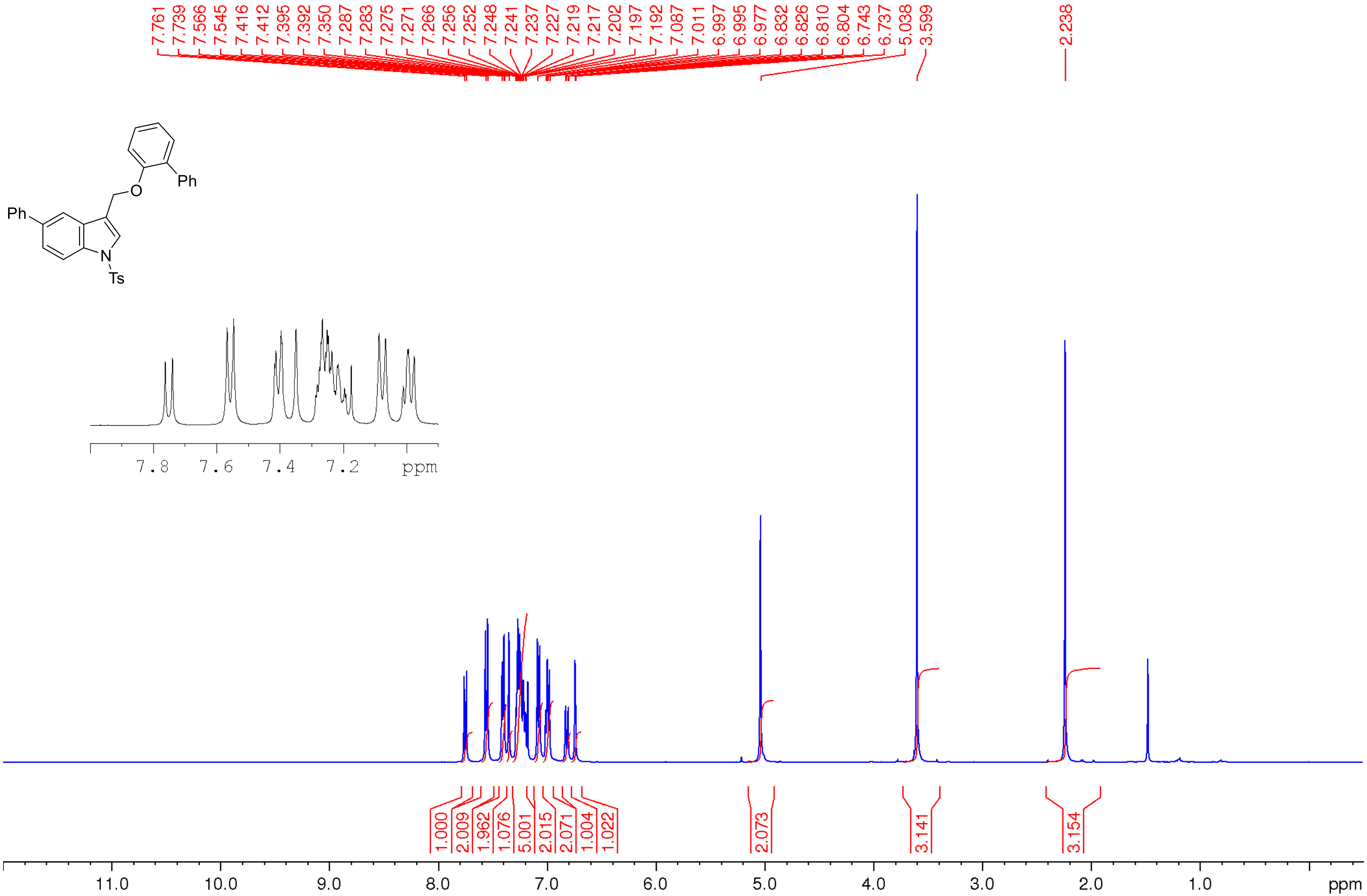

3-(([1,1'-biphenyl]-2-yloxy)methyl)-5-phenyl-1-tosyl-1H-indole ***4ed***

^{13}C NMR-spectrum (100.6 MHz) ($CDCl_3$)

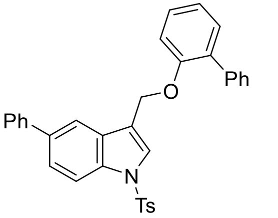

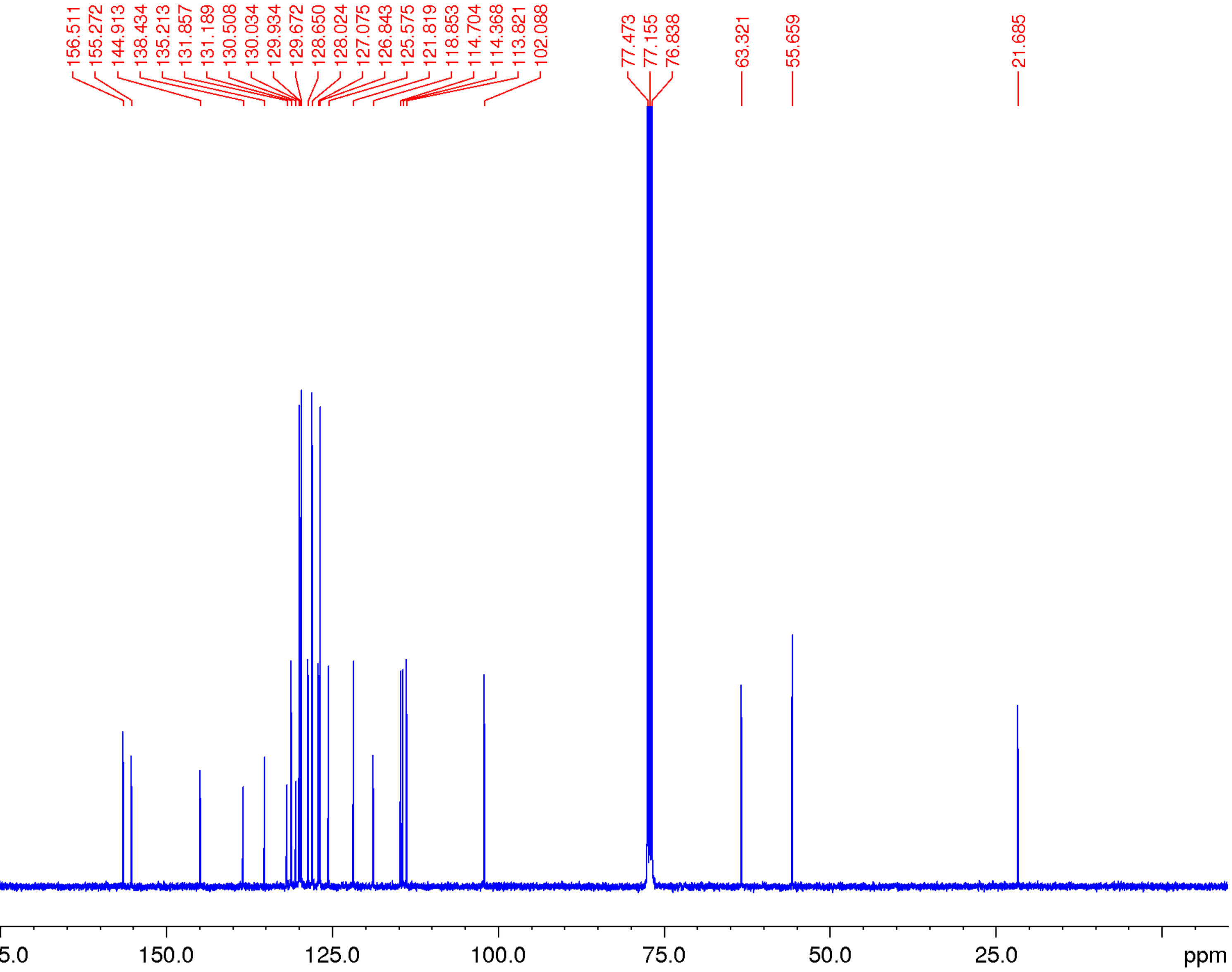

3-(([1,1'-biphenyl]-2-yloxy)methyl)-5-phenyl-1-tosyl-1H-indole ***4ed*** DEPT 135 NMR-spectrum ($CDCl_3$)

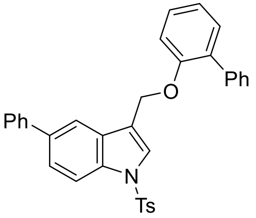

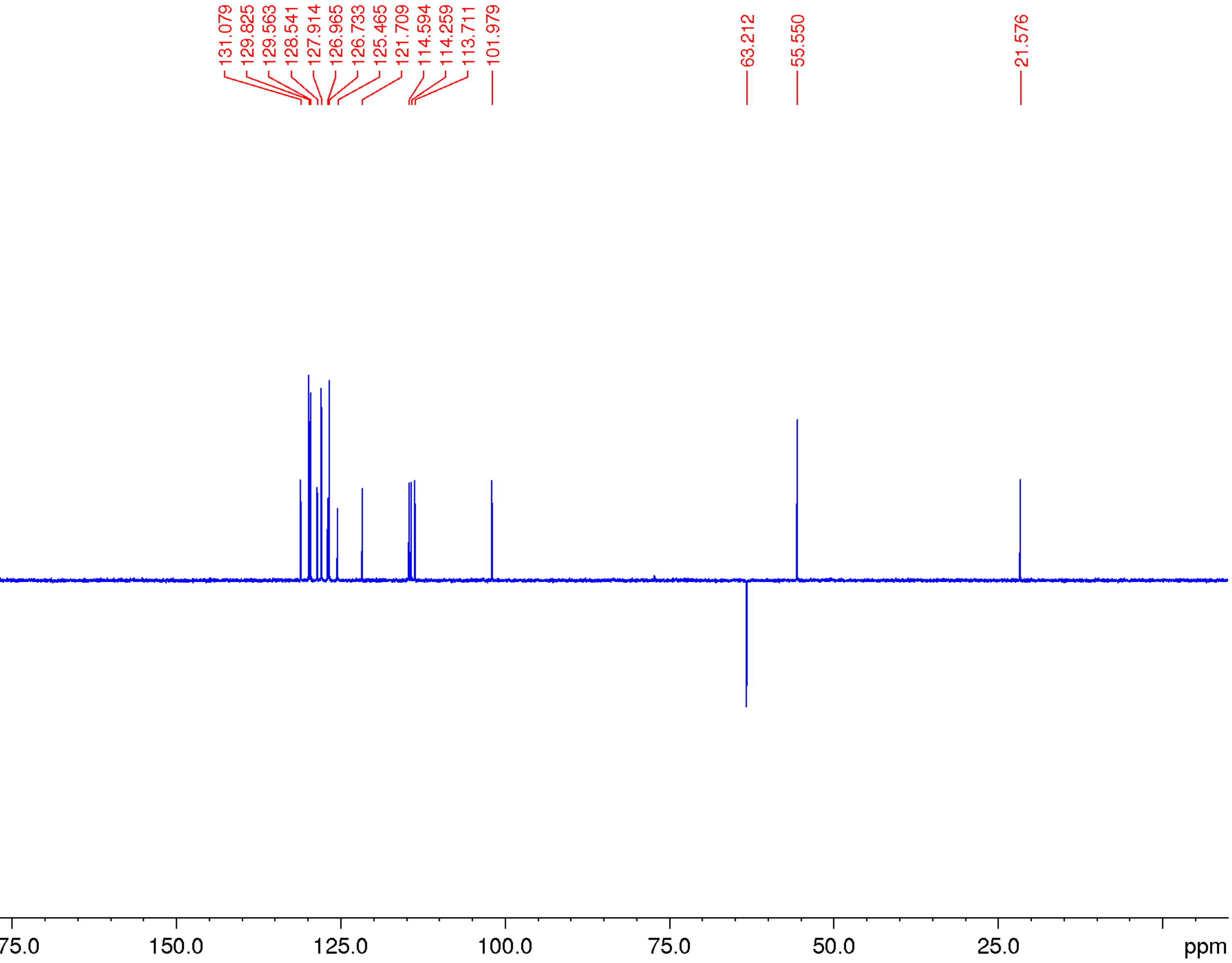

3-((phenylsulfonyl)methyl)-1-tosyl-1*H*-indole **9ab**

^{1}H NMR-spectrum (400.13 MHz) (DMSO-d_6)

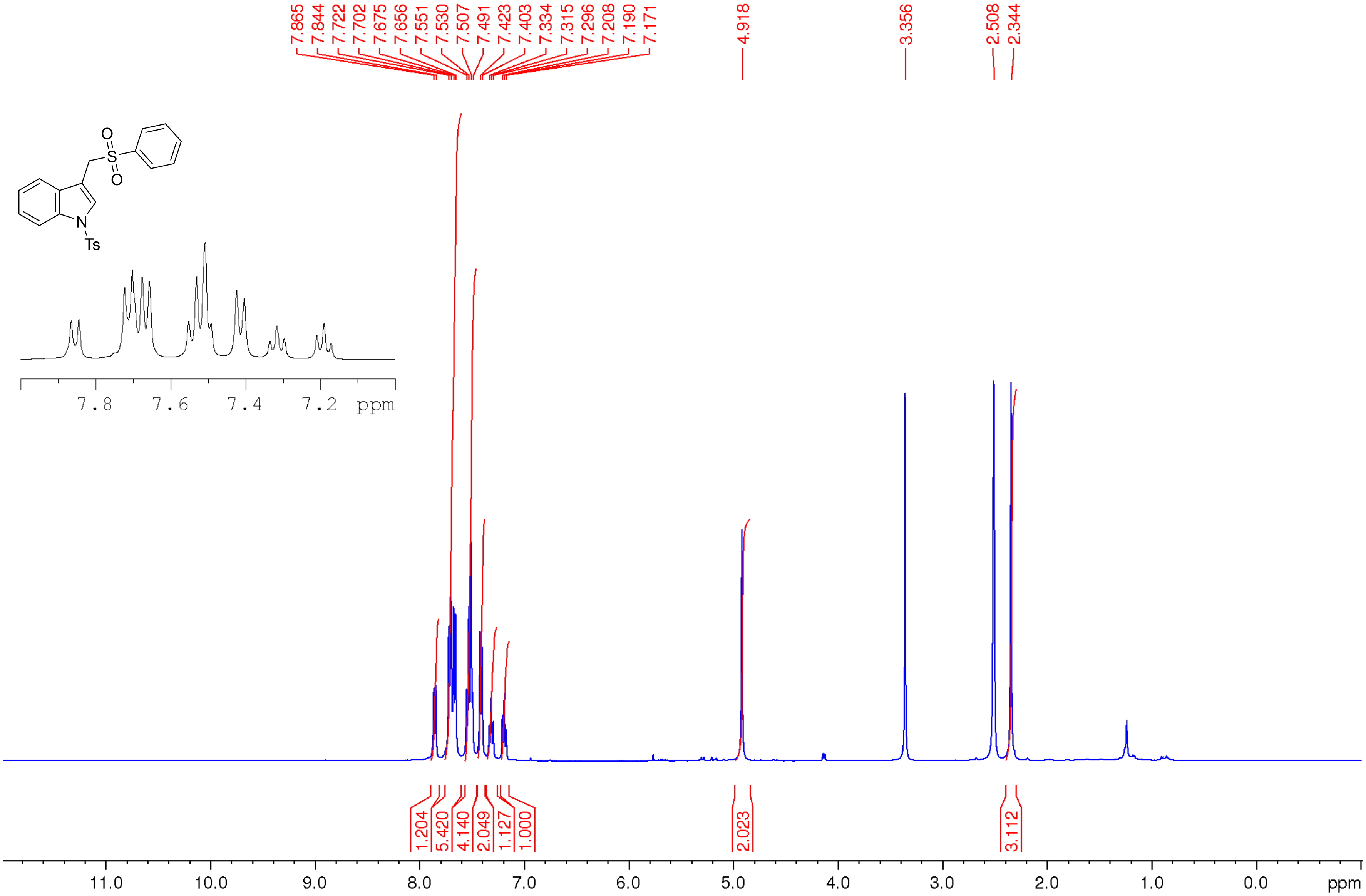

3-((phenylsulfonyl)methyl)-1-tosyl-1*H*-indole **9ab**

^{13}C NMR-spectrum (100.6 MHz) (DMSO-d_6)

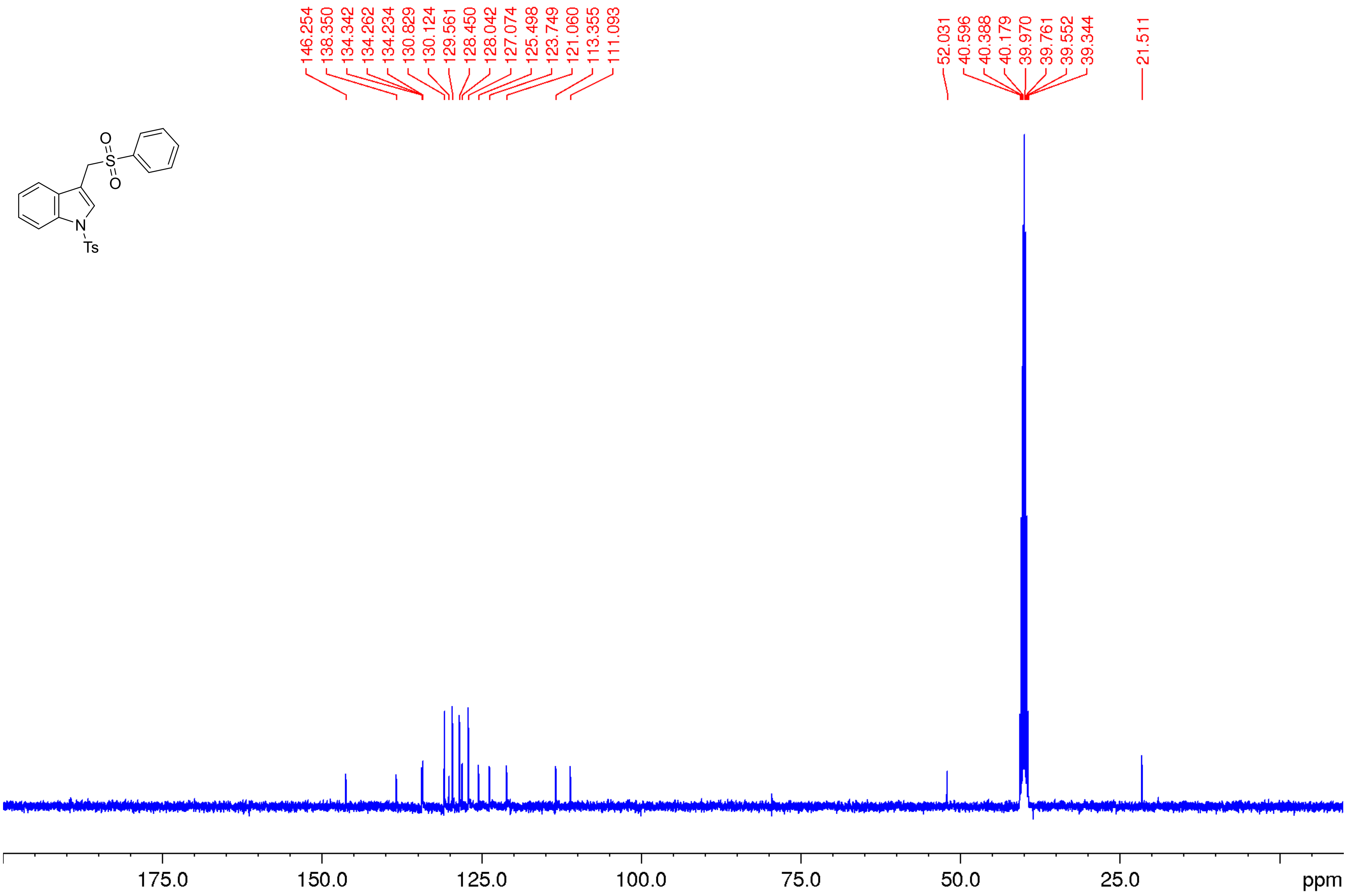

3-((phenylsulfonyl)methyl)-1-tosyl-1*H*-indole **9ab**

DEPT 135 NMR-spectrum ((DMSO-d_6))

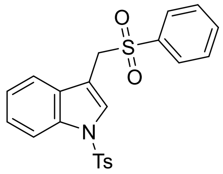

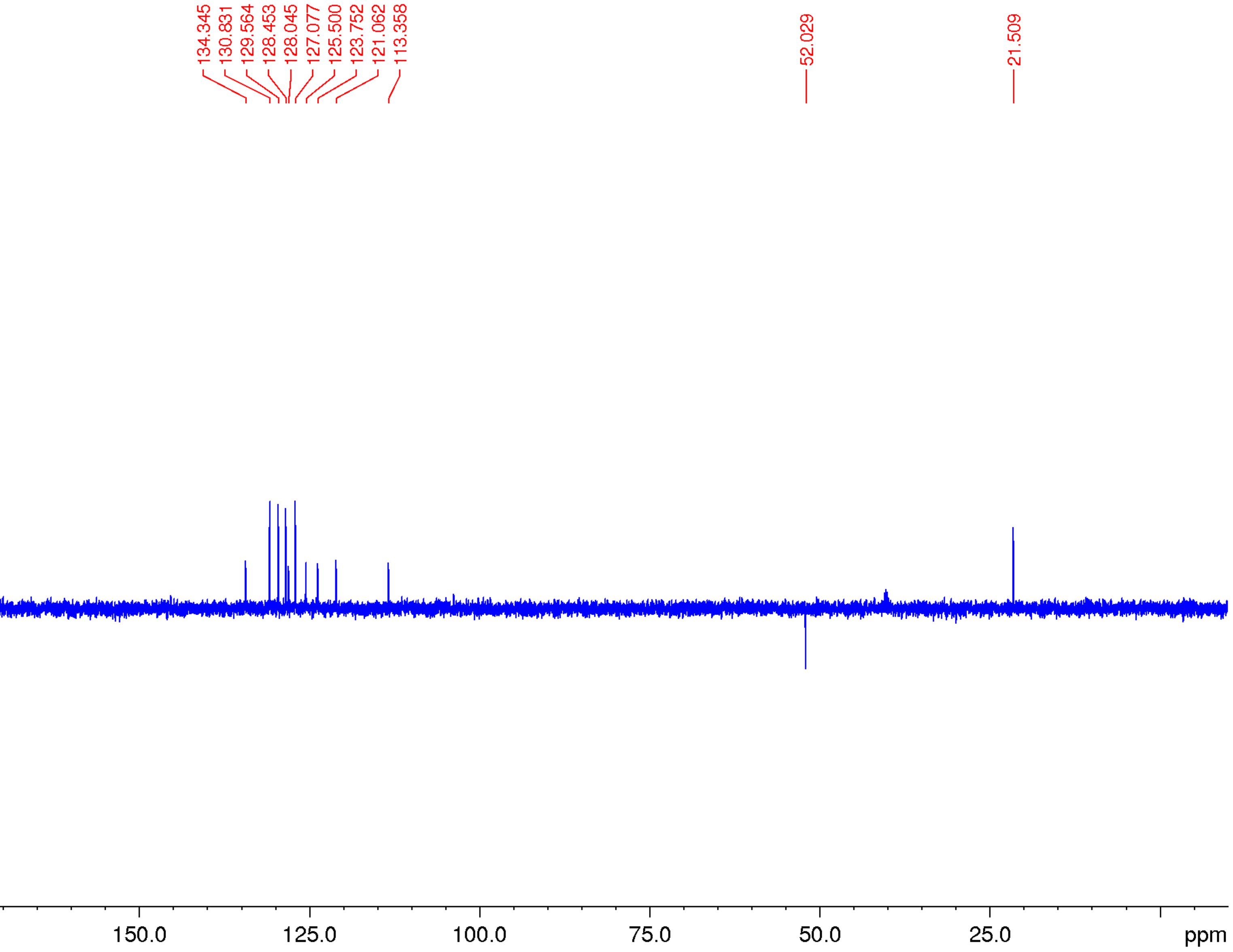

5-methoxy-1-tosyl-3-(tosylmethyl)-1*H*-indole **9da**

^{1}H NMR-spectrum (400.13 MHz) ($CDCl_3$)

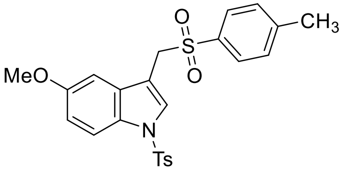

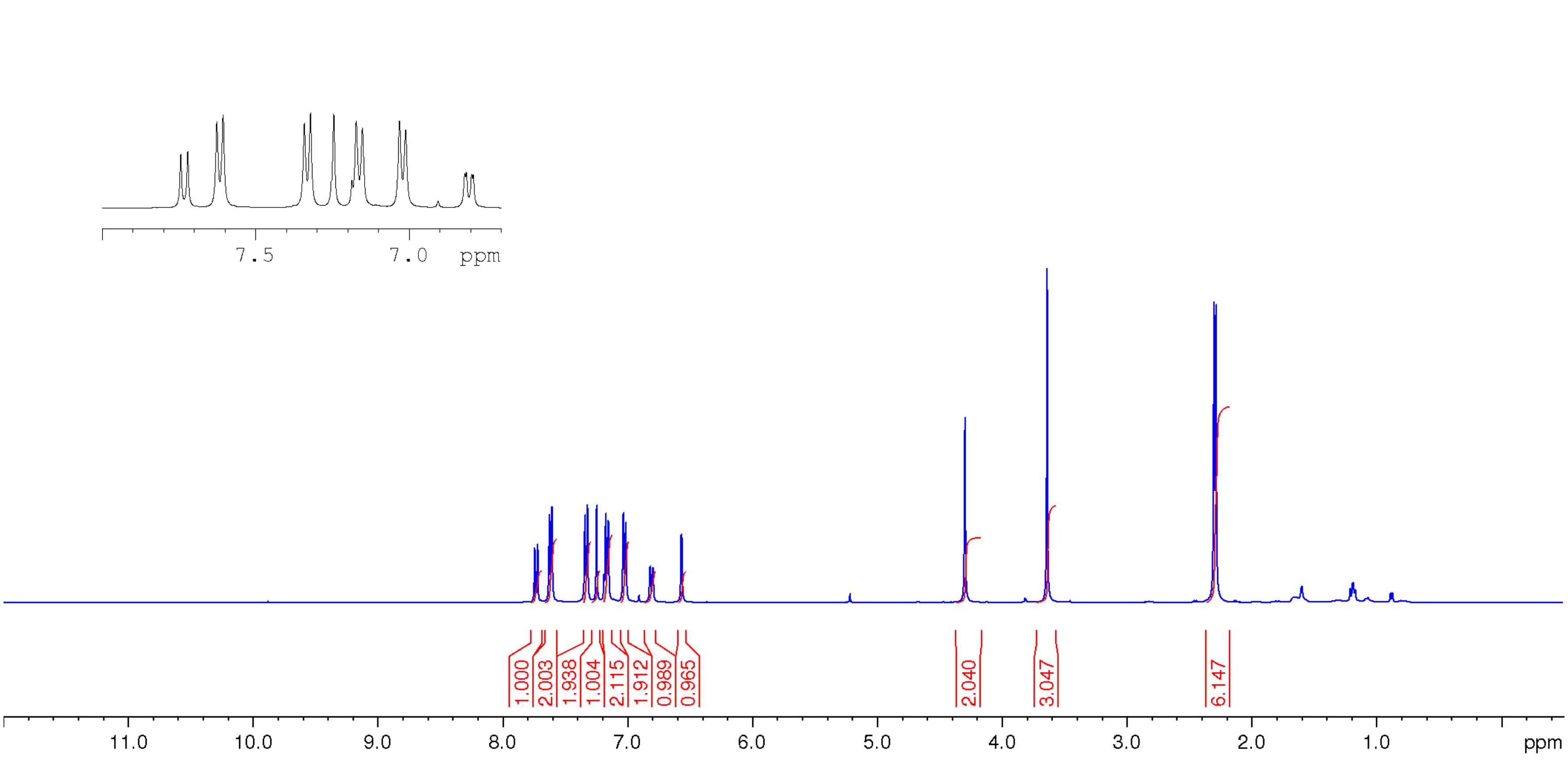

5-methoxy-1-tosyl-3-(tosylmethyl)-1*H*-indole **9da**

^{13}C NMR-spectrum (100.6 MHz) ($CDCl_3$)

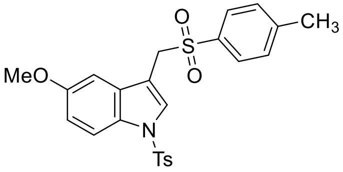

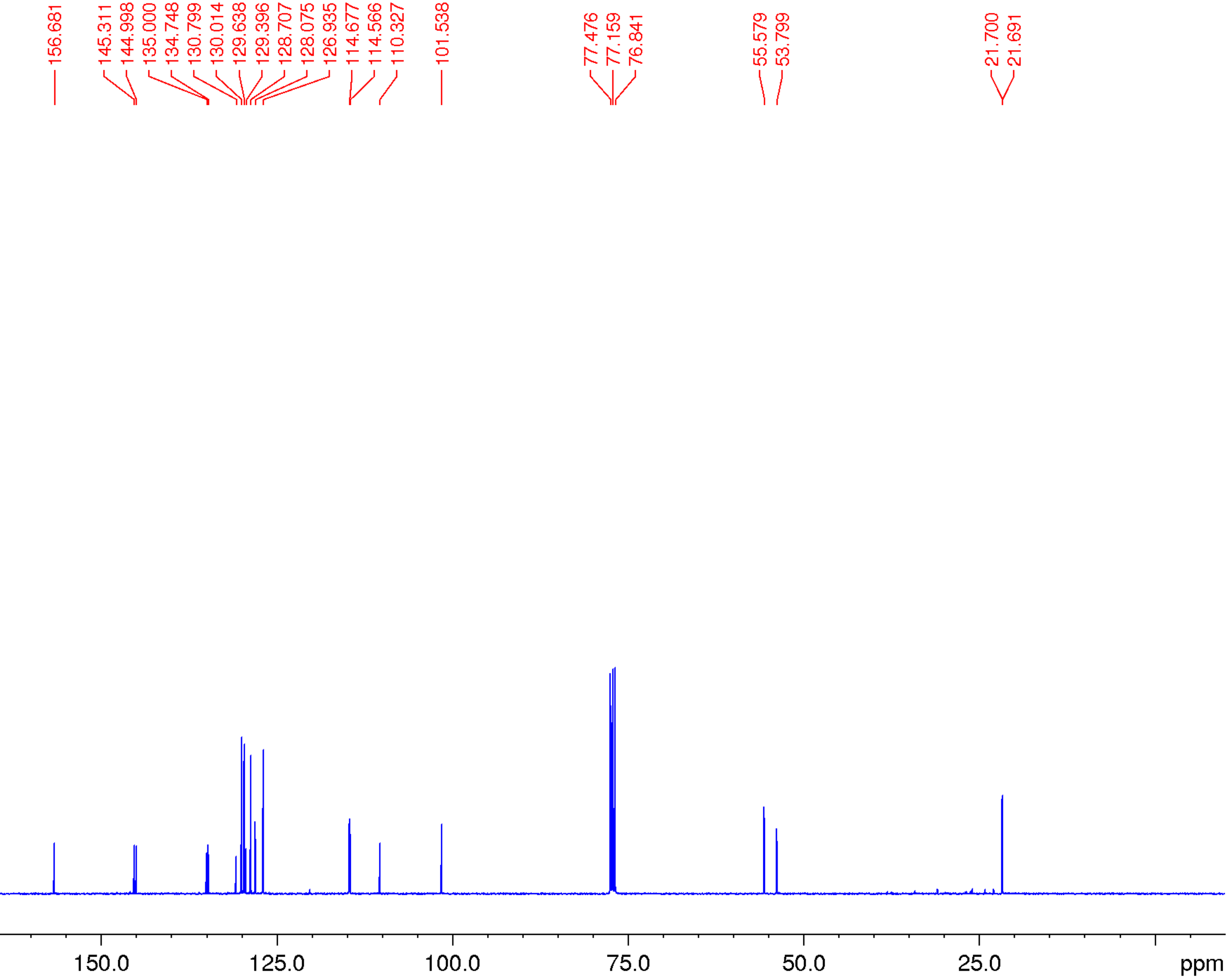

5-methoxy-1-tosyl-3-(tosylmethyl)-1*H*-indole **9da**

DEPT 135 NMR-spectrum ($CDCl_3$)

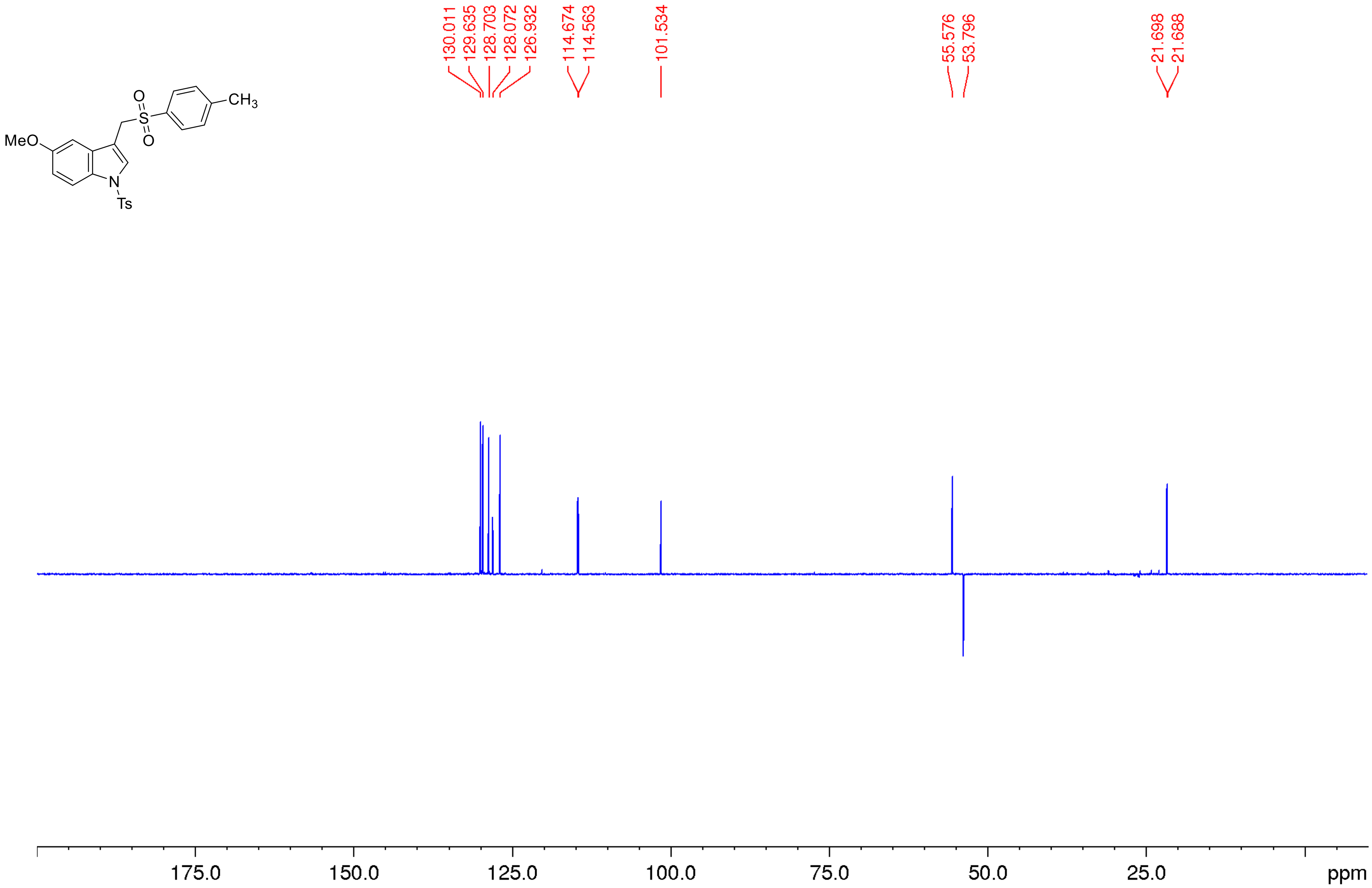

5-methoxy-3-((phenylsulfonyl)methyl)-1-tosyl-1*H*-indole **9db**

^{1}H NMR-spectrum (400.13 MHz) ($CDCl_3$)

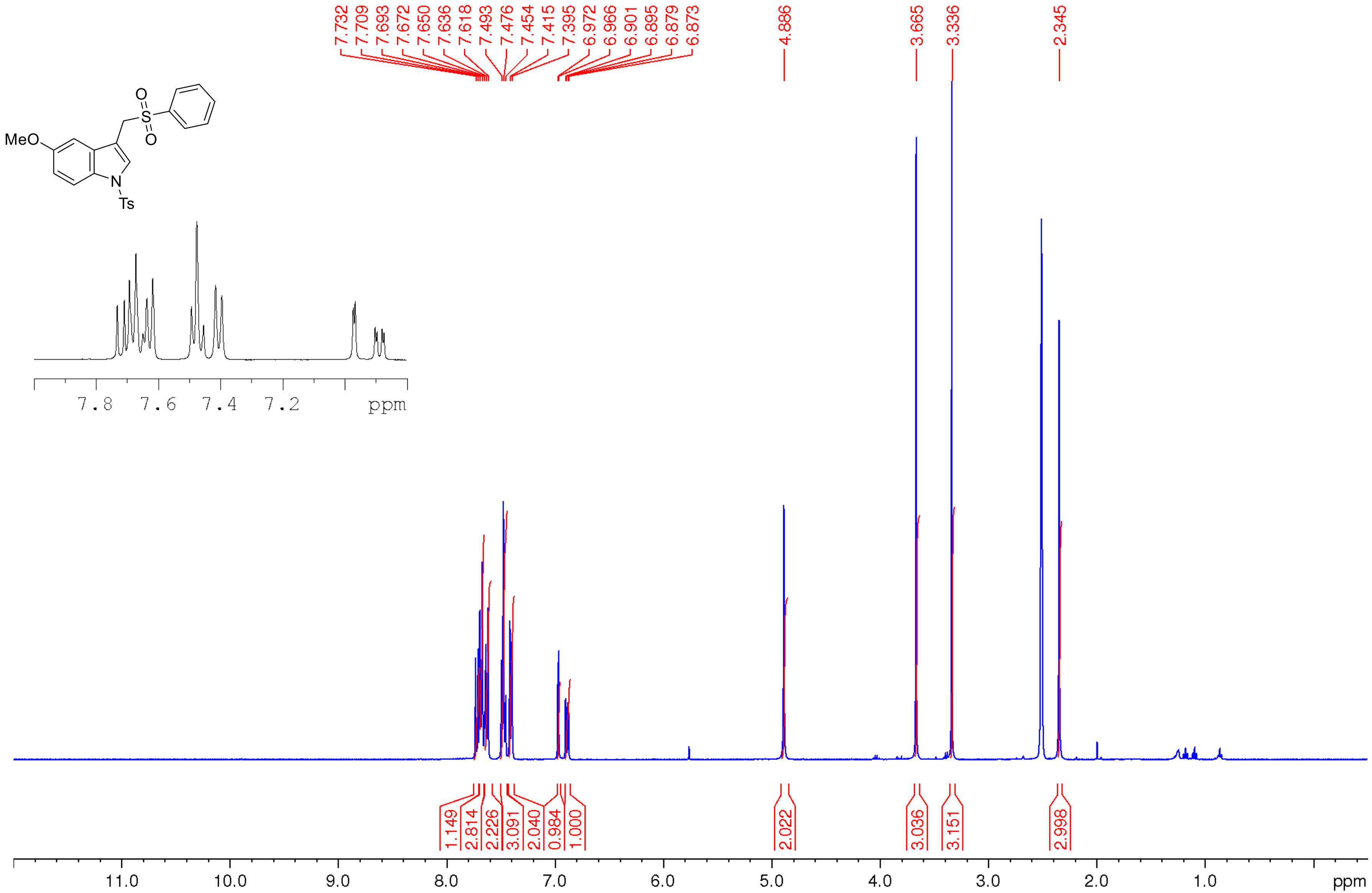

5-methoxy-3-((phenylsulfonyl)methyl)-1-tosyl-1*H*-indole **9db**

^{13}C NMR-spectrum (100.6 MHz) ($CDCl_3$)

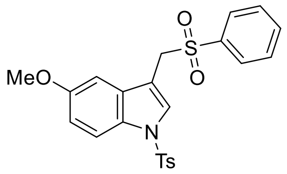

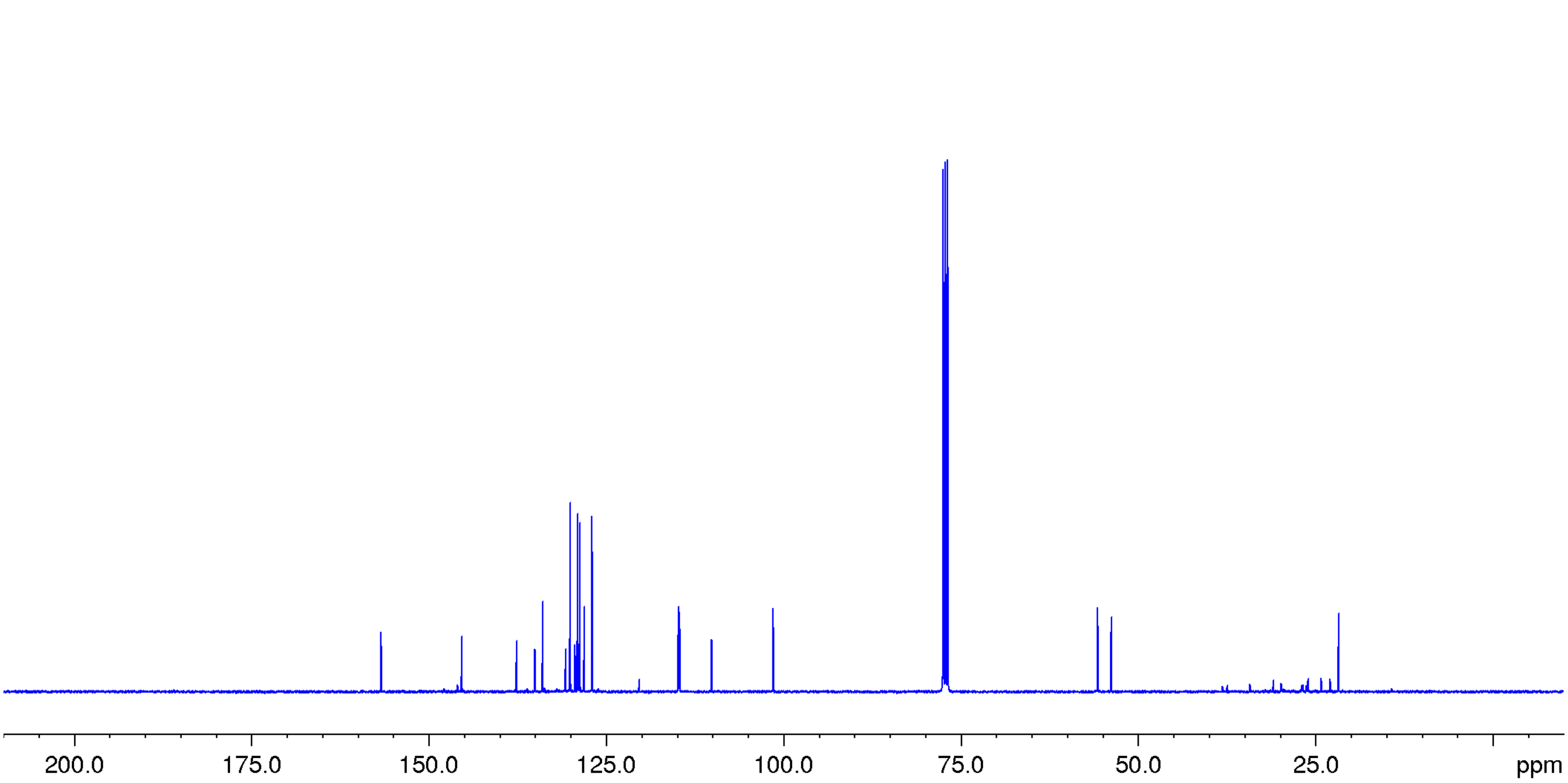

5-methoxy-3-((phenylsulfonyl)methyl)-1-tosyl-1*H*-indole **9db**

DEPT 135 NMR-spectrum ($CDCl_3$)

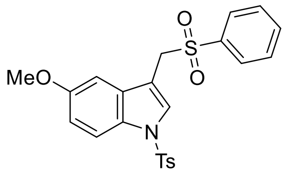

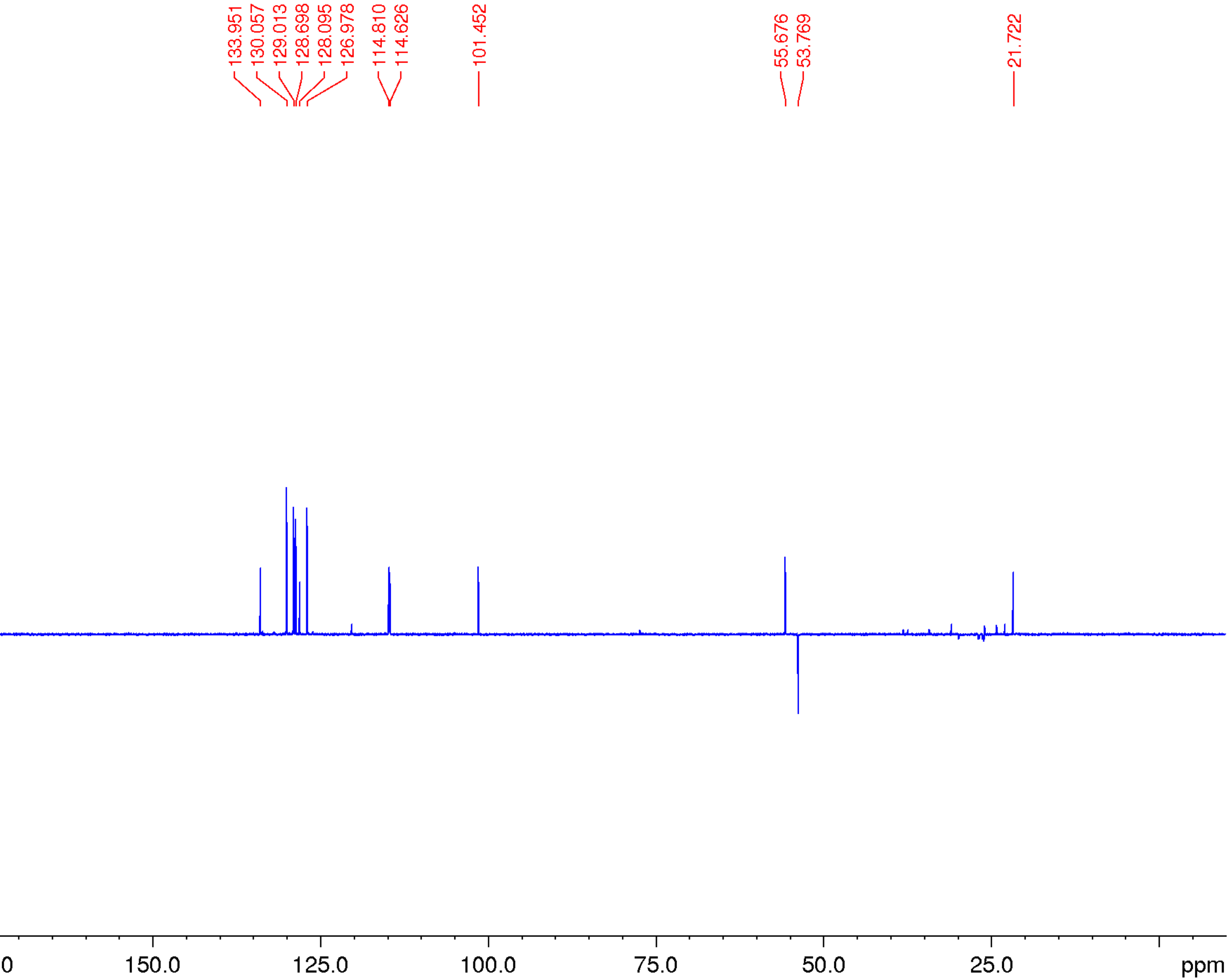

5-phenyl-1-tosyl-3-(tosylmethyl)-1H-indole ***9ea***

^{1}H NMR-spectrum (400.13 MHz) ($CDCl_3$)

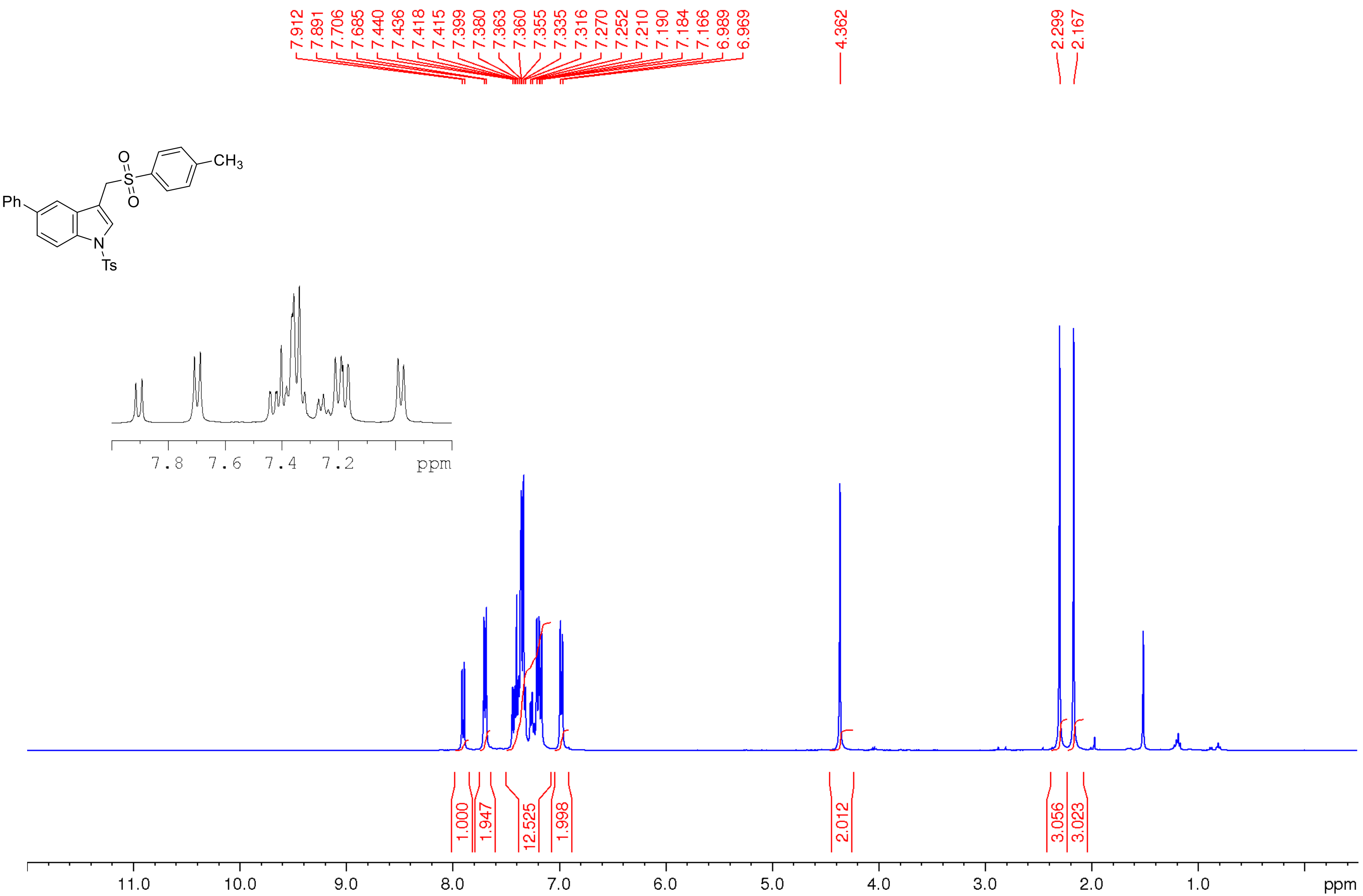

5-phenyl-1-tosyl-3-(tosylmethyl)-1H-indole ***9ea***

^{13}C NMR-spectrum (100.6 MHz) ($CDCl_3$)

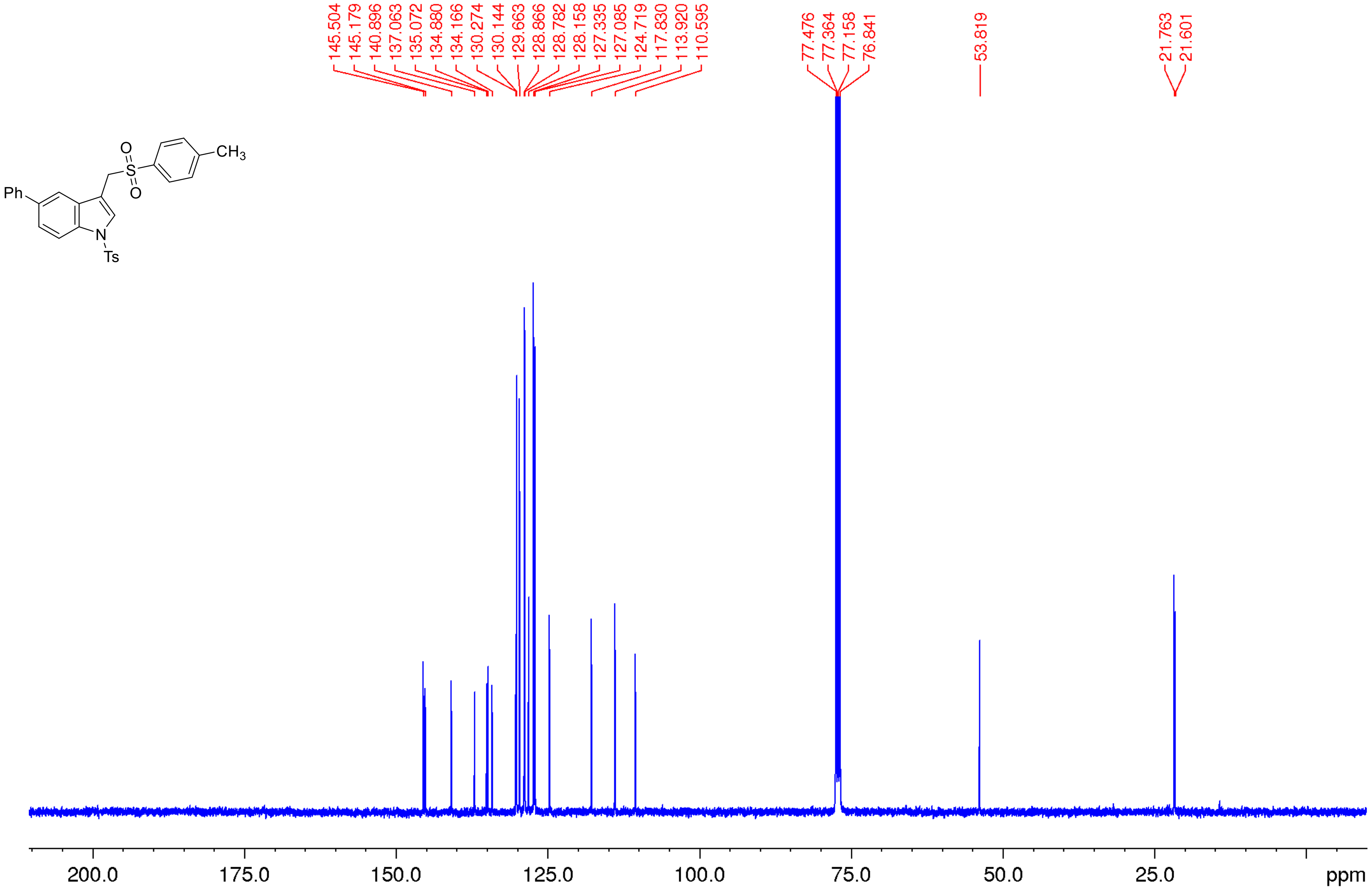

5-phenyl-1-tosyl-3-(tosylmethyl)-1H-indole ***9ea***

DEPT 135 NMR-spectrum ($CDCl_3$)

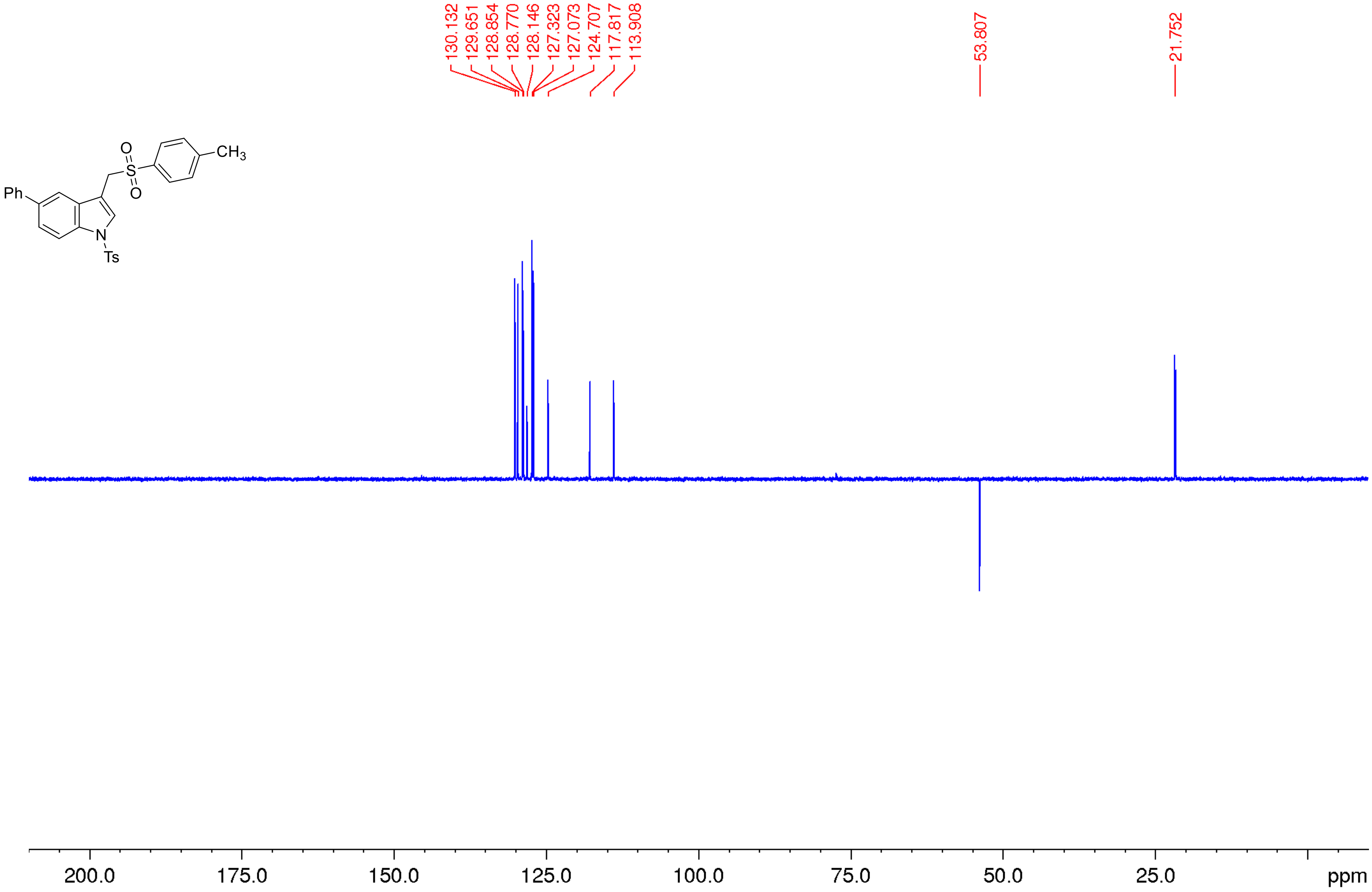

5-phenyl-3-((phenylsulfonyl)methyl)-1-tosyl-1H-indole ***9eb***

^{1}H NMR-spectrum (400.13 MHz) ($CDCl_3$)

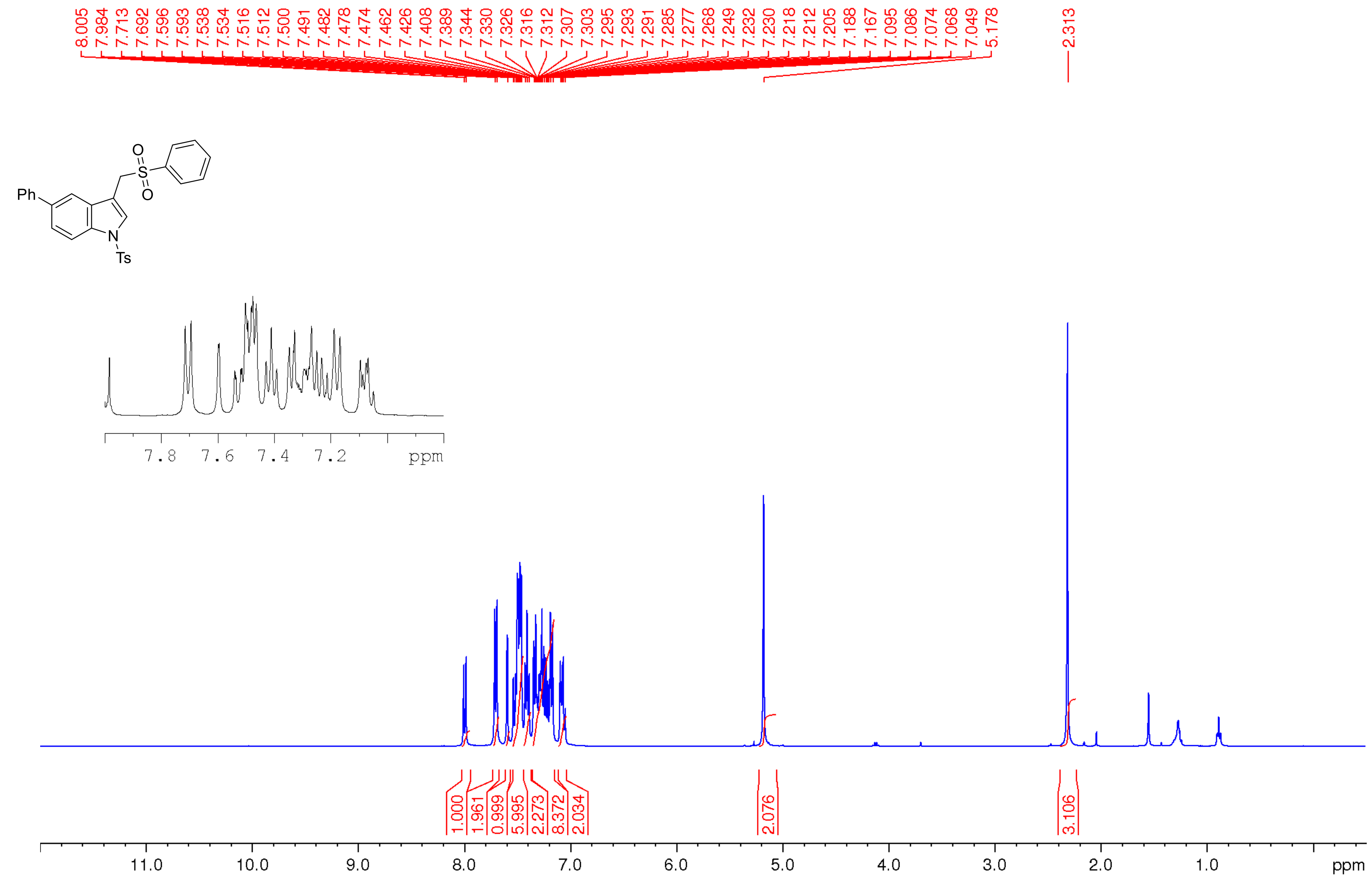

5-phenyl-3-((phenylsulfonyl)methyl)-1-tosyl-1H-indole ***9eb***

^{13}C NMR-spectrum (100.6 MHz) ($CDCl_3$)

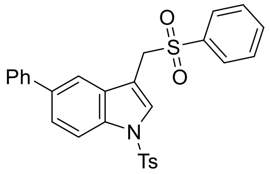

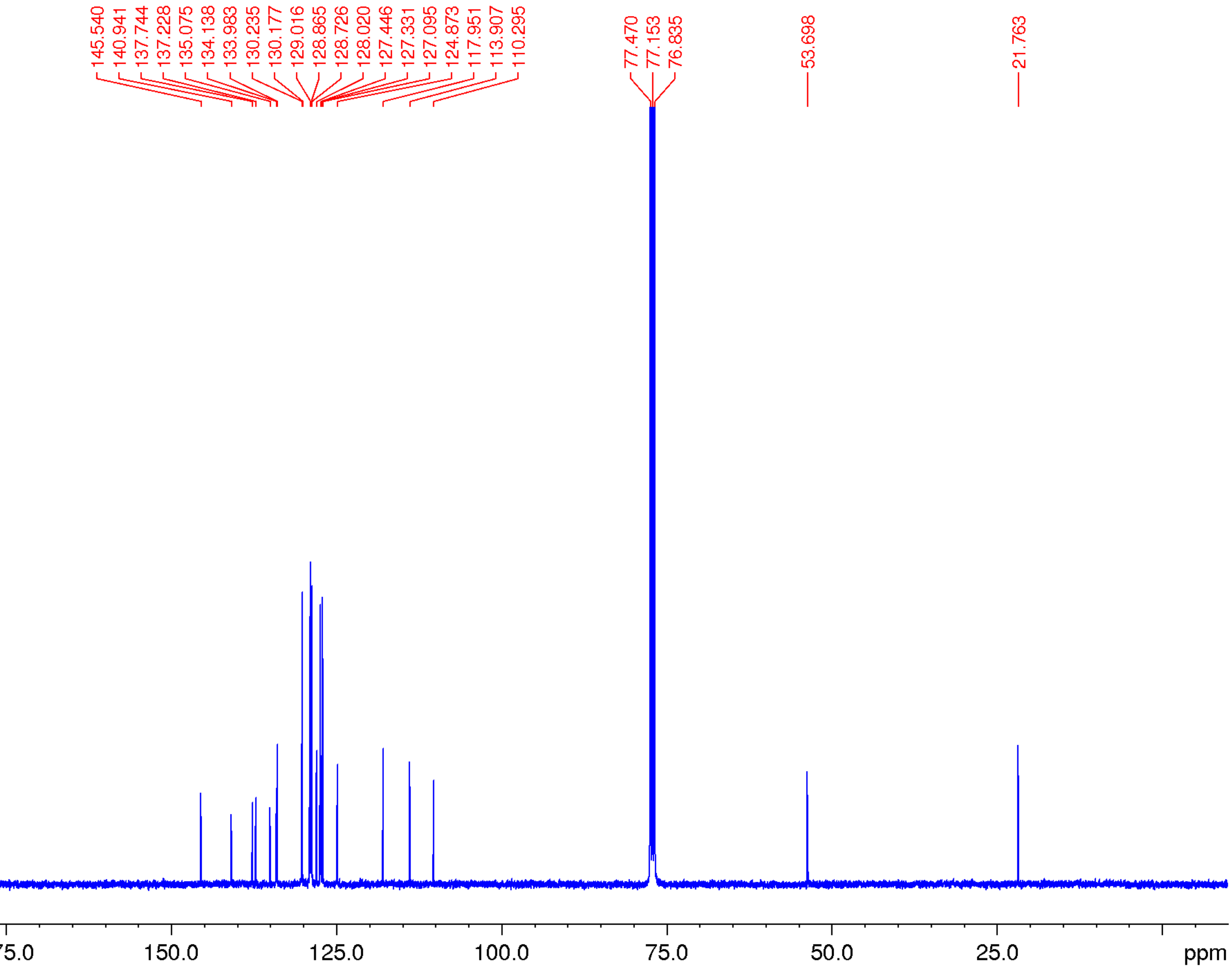

5-phenyl-3-((phenylsulfonyl)methyl)-1-tosyl-1H-indole ***9eb***

DEPT 135 NMR-spectrum ($CDCl_3$)

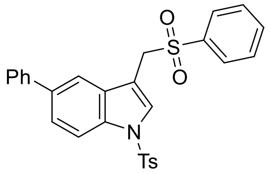

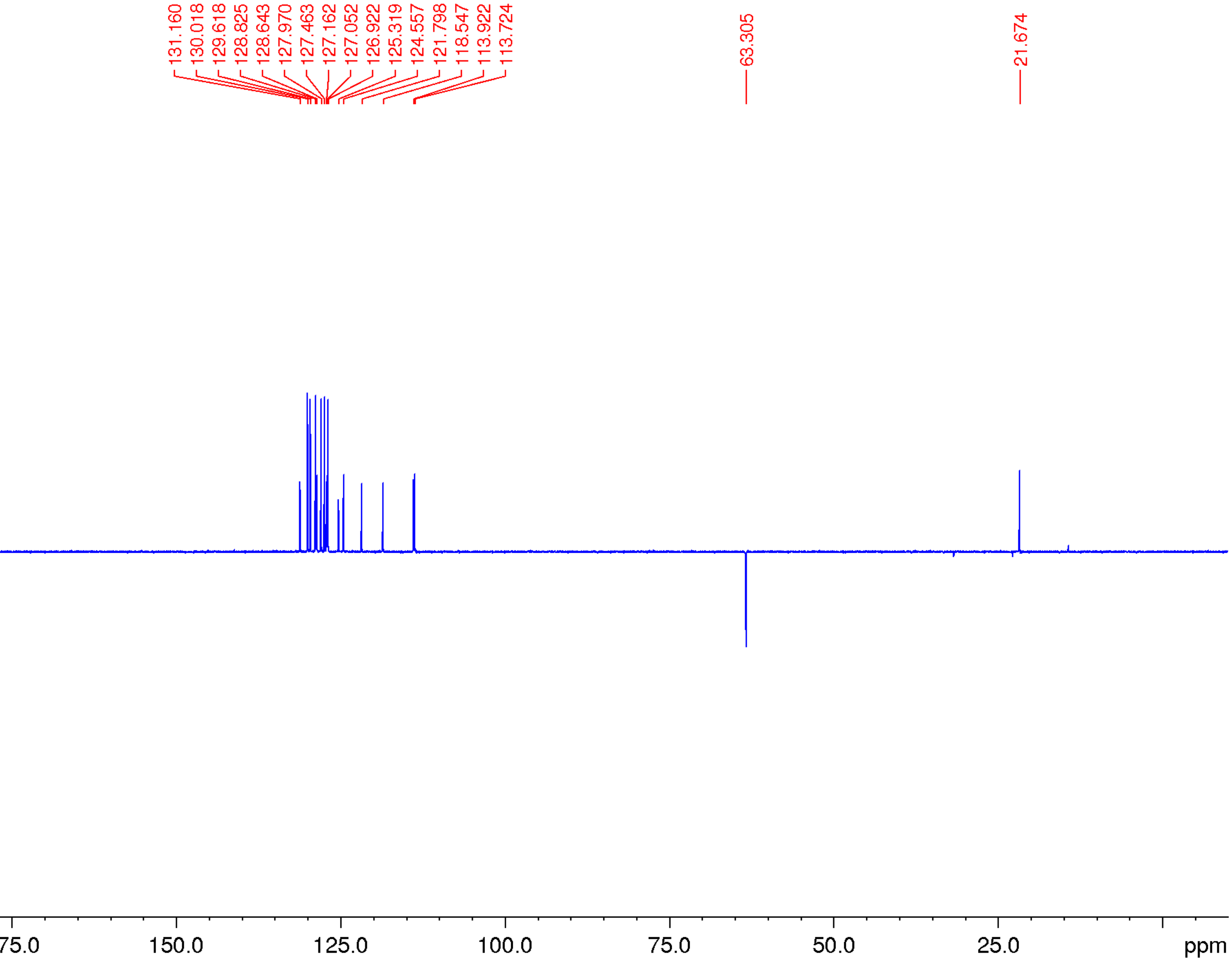

6-chloro-1-tosyl-3-(tosylmethyl)-1H-indole ***9fa***

^{1}H NMR-spectrum (400.13 MHz) ($CDCl_3$)

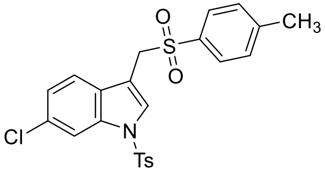

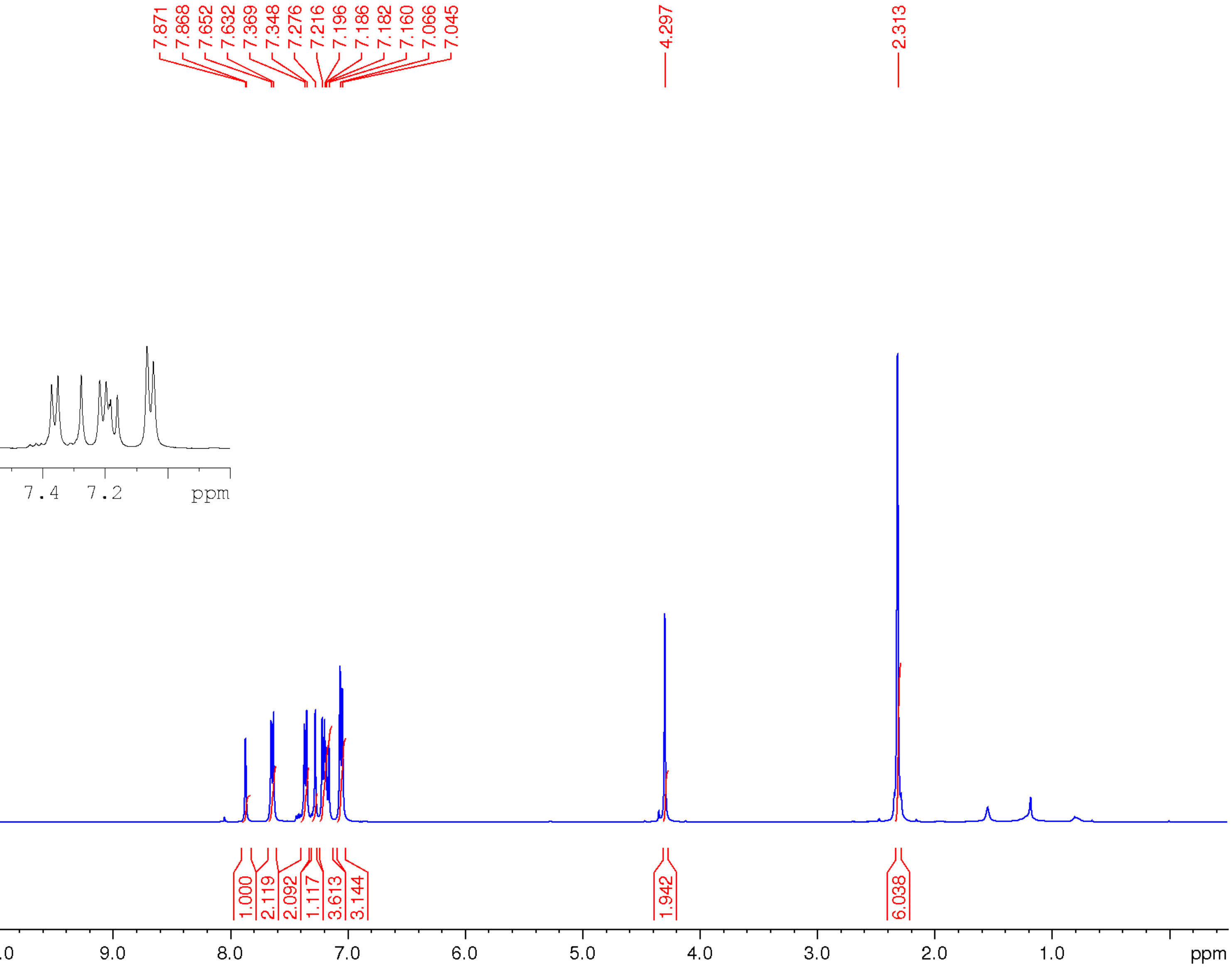

6-chloro-1-tosyl-3-(tosylmethyl)-1H-indole ***9fa***

^{13}C NMR-spectrum (100.6 MHz) ($CDCl_3$)

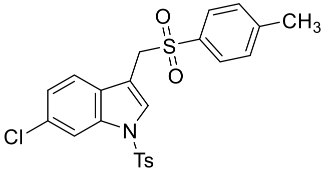

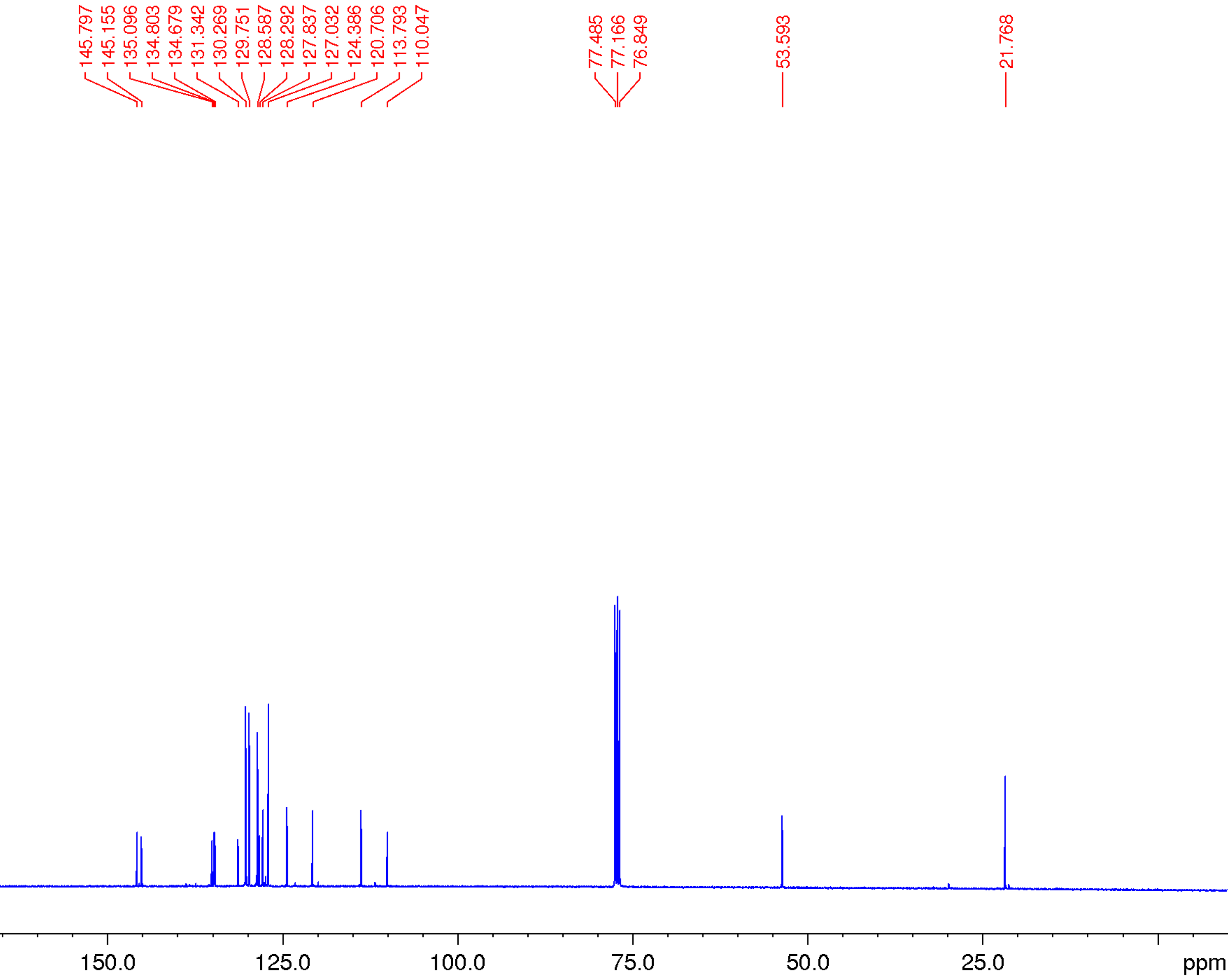

6-chloro-1-tosyl-3-(tosylmethyl)-1H-indole ***9fa***

DEPT 135 NMR-spectrum ($CDCl_3$)

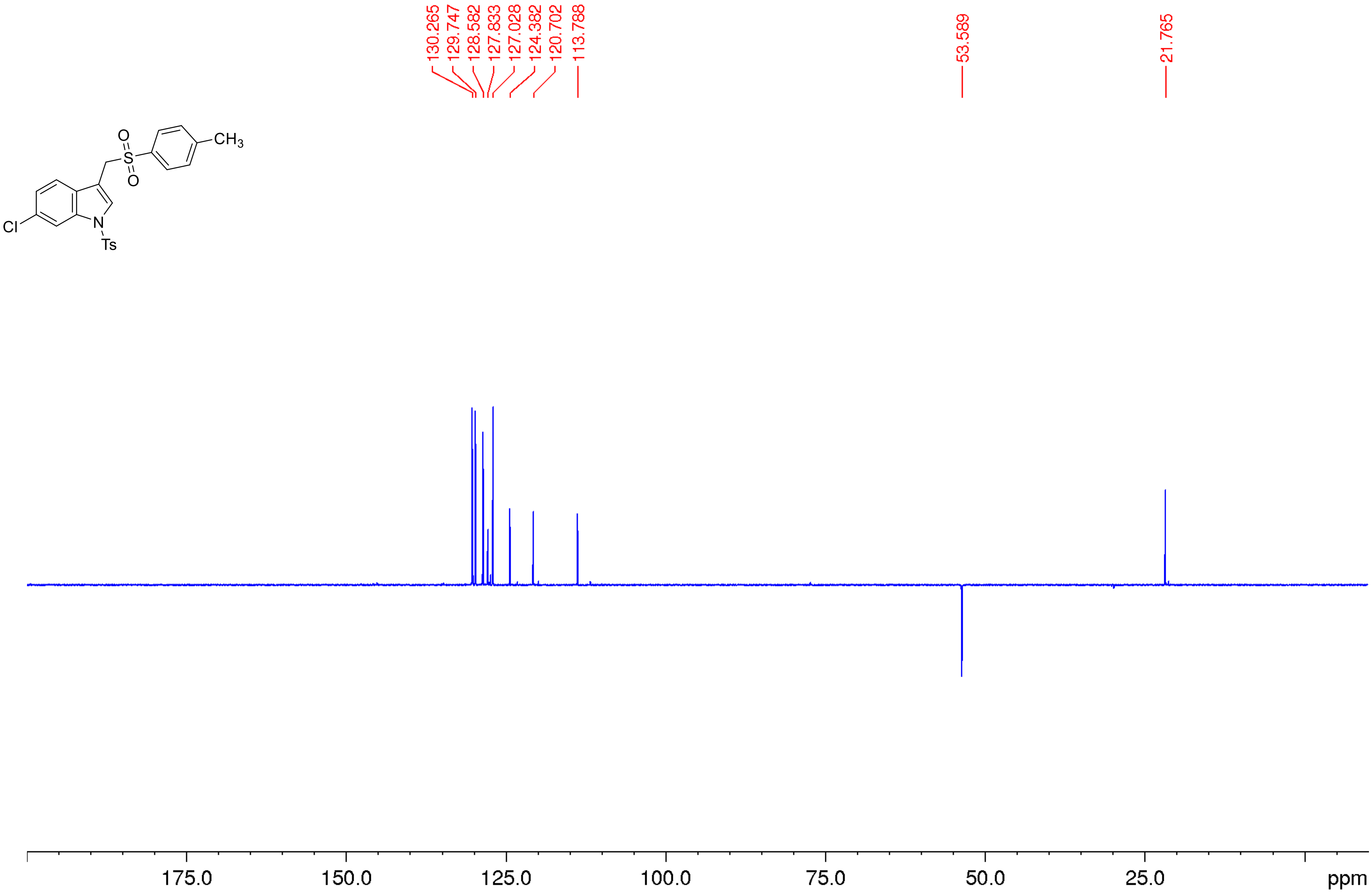

6-chloro-3-((phenylsulfonyl)methyl)-1-tosyl-1H-indole ***9fb***

^{1}H NMR-spectrum (400.13 MHz) ($CDCl_3$)

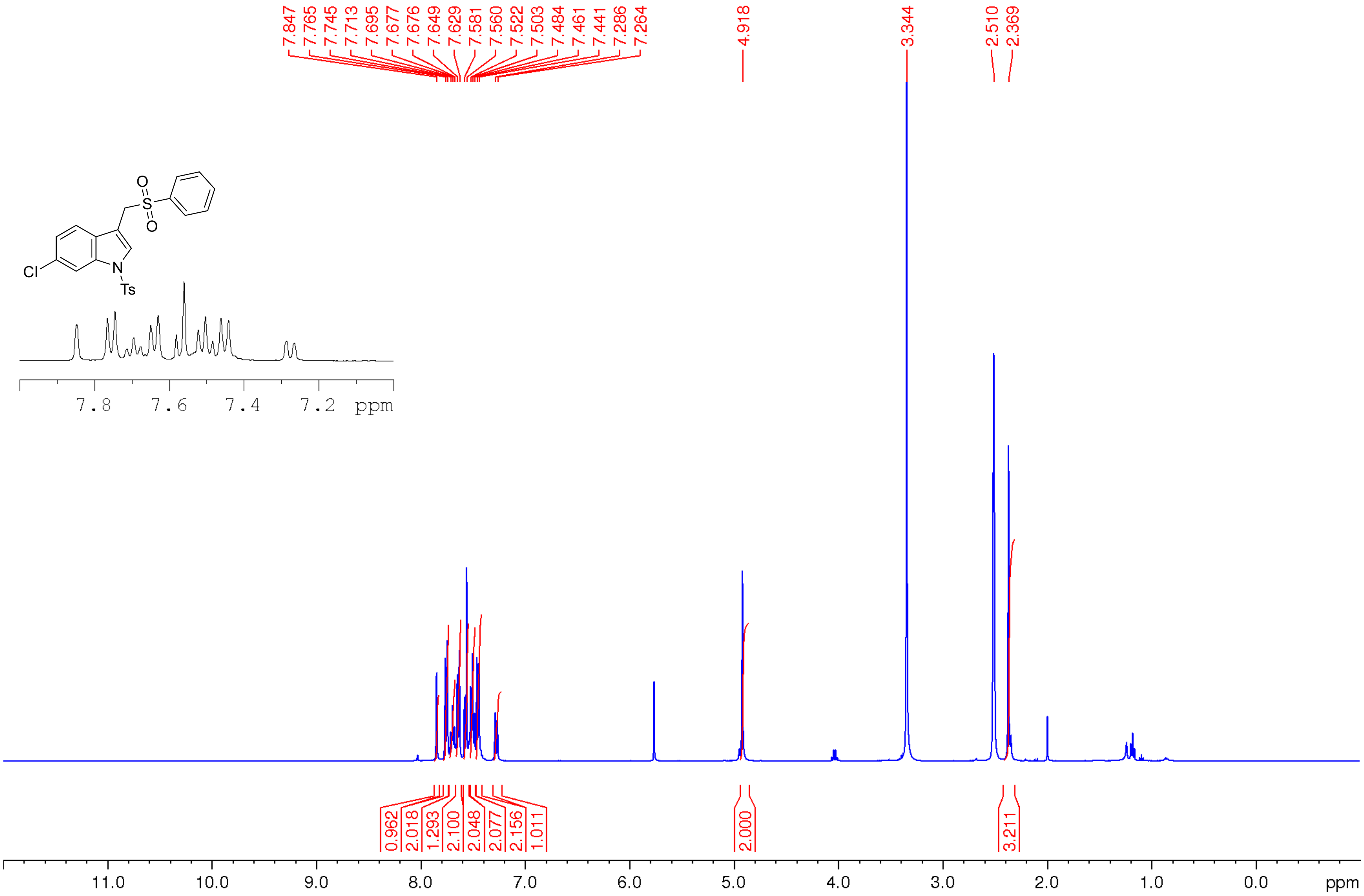

6-chloro-3-((phenylsulfonyl)methyl)-1-tosyl-1H-indole ***9fb***

^{13}C NMR-spectrum (100.6 MHz) ($CDCl_3$)

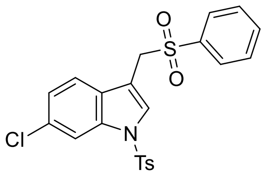

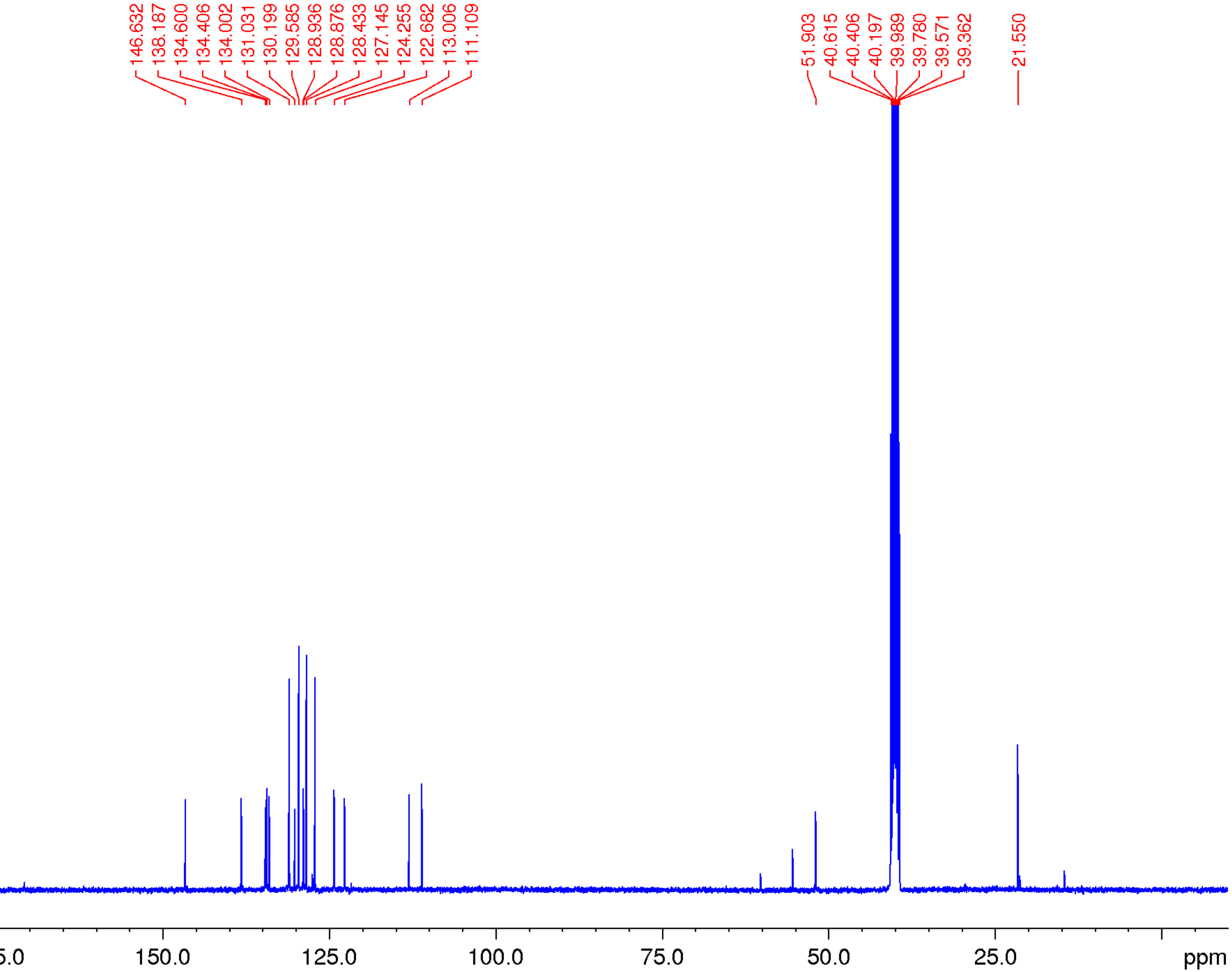

6-chloro-3-((phenylsulfonyl)methyl)-1-tosyl-1H-indole ***9fb***

DEPT 135 NMR-spectrum ($CDCl_3$)

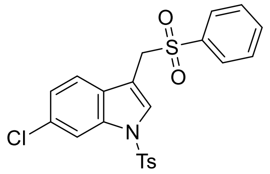

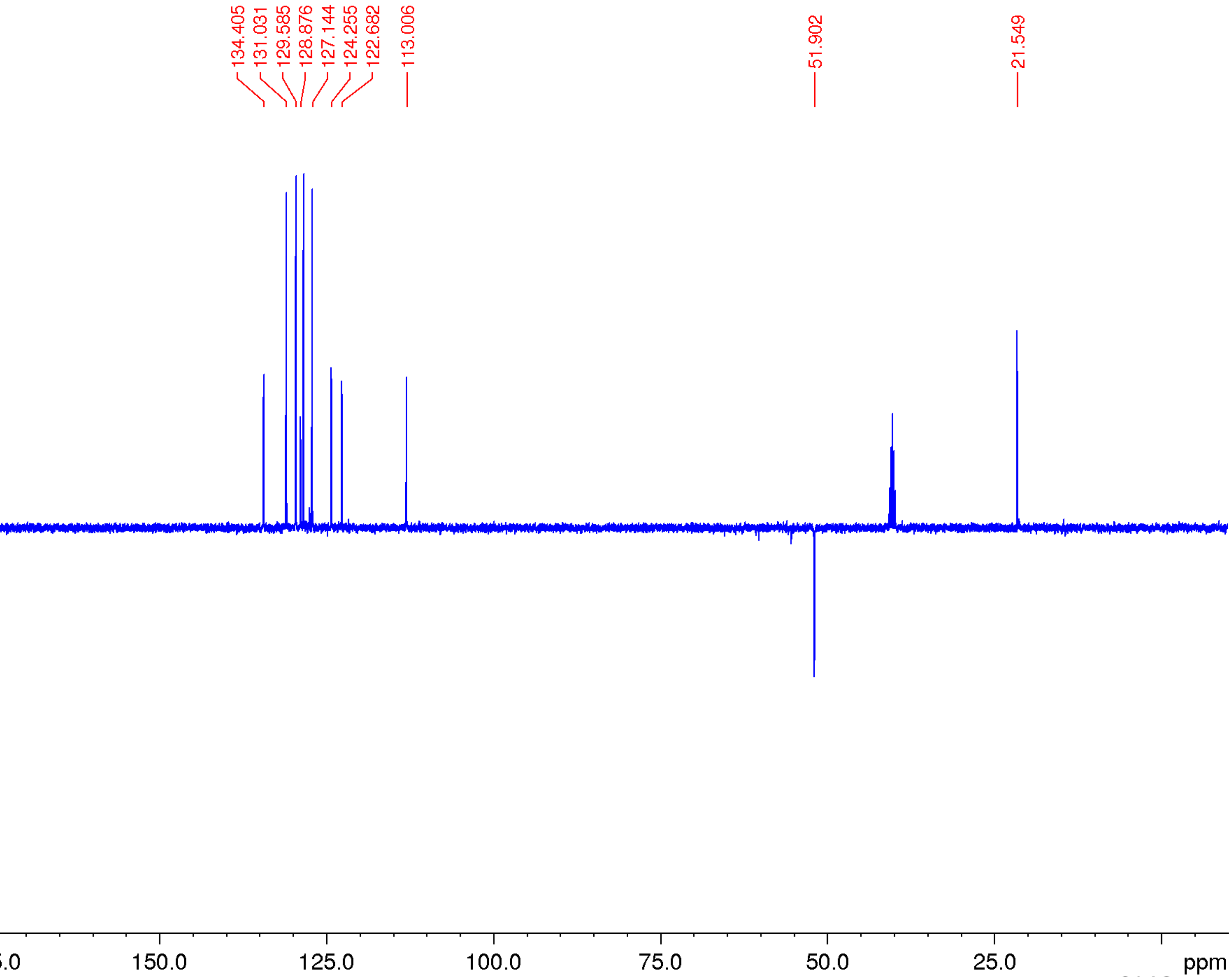

5-nitro-1-tosyl-3-(tosylmethyl)-1H-indole ***9ga***

^{1}H NMR-spectrum (400.13 MHz) ($CDCl_3$)

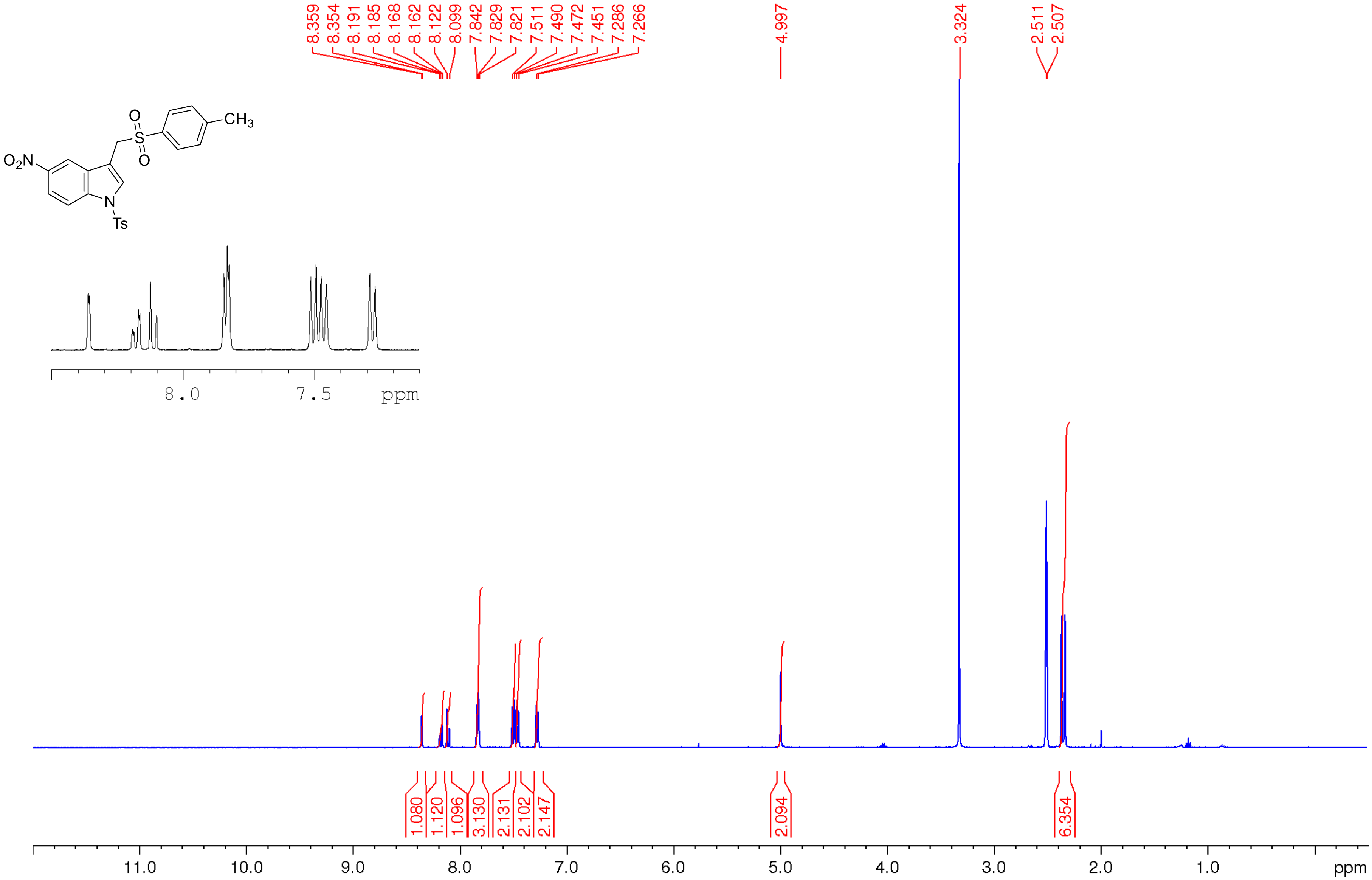

*5-nitro-1-tosyl-3-(tosylmethyl)-1H-indole **9ga***

^{13}C NMR-spectrum (100.6 MHz) ($CDCl_3$)

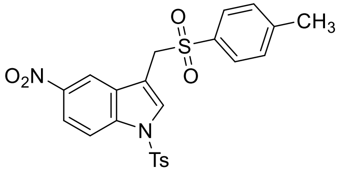

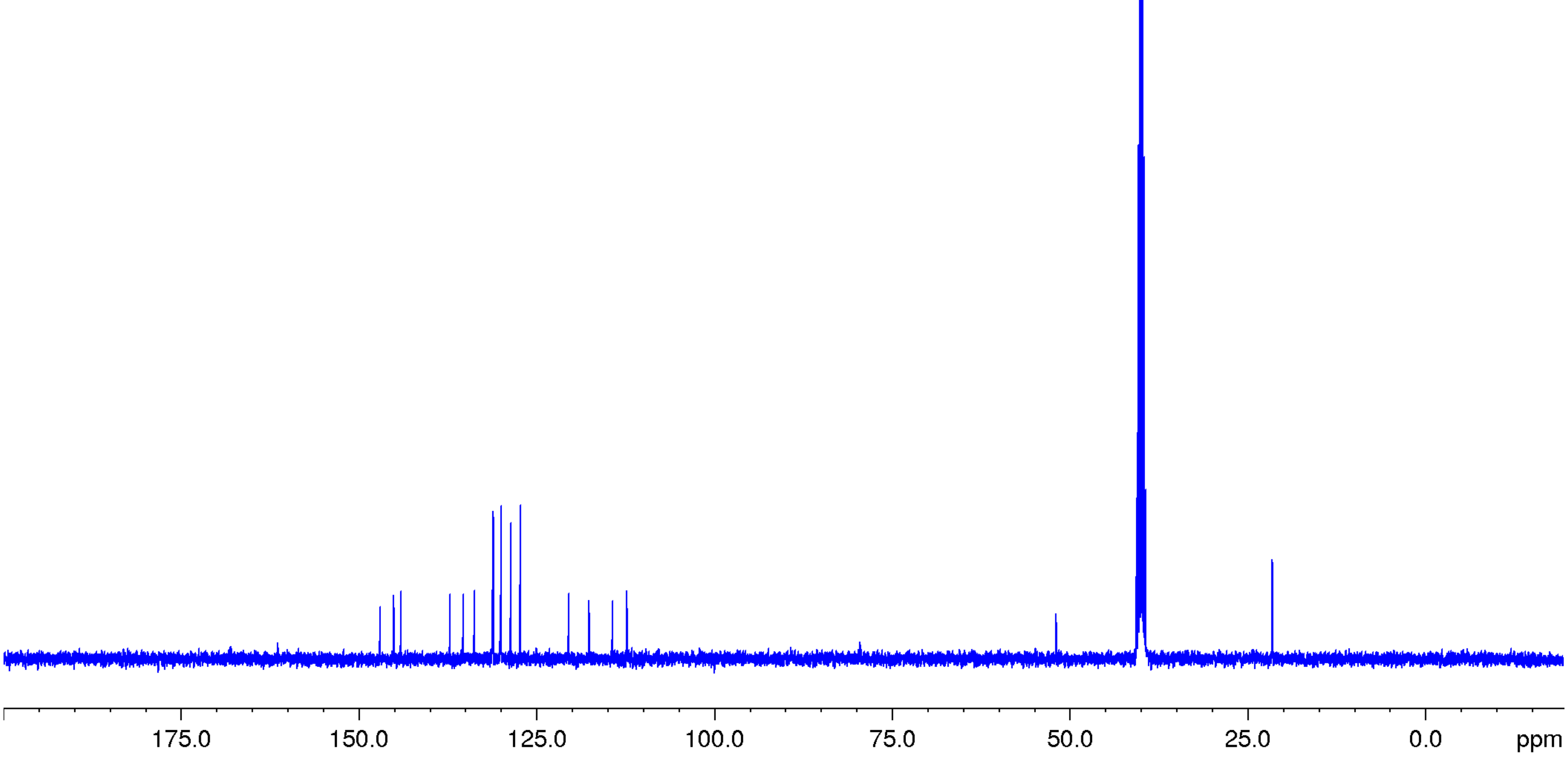

5-nitro-1-tosyl-3-(tosylmethyl)-1H-indole ***9ga***

DEPT 135 NMR-spectrum ($CDCl_3$)

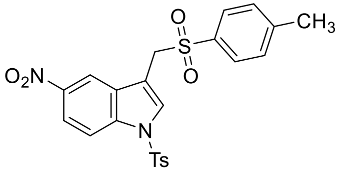

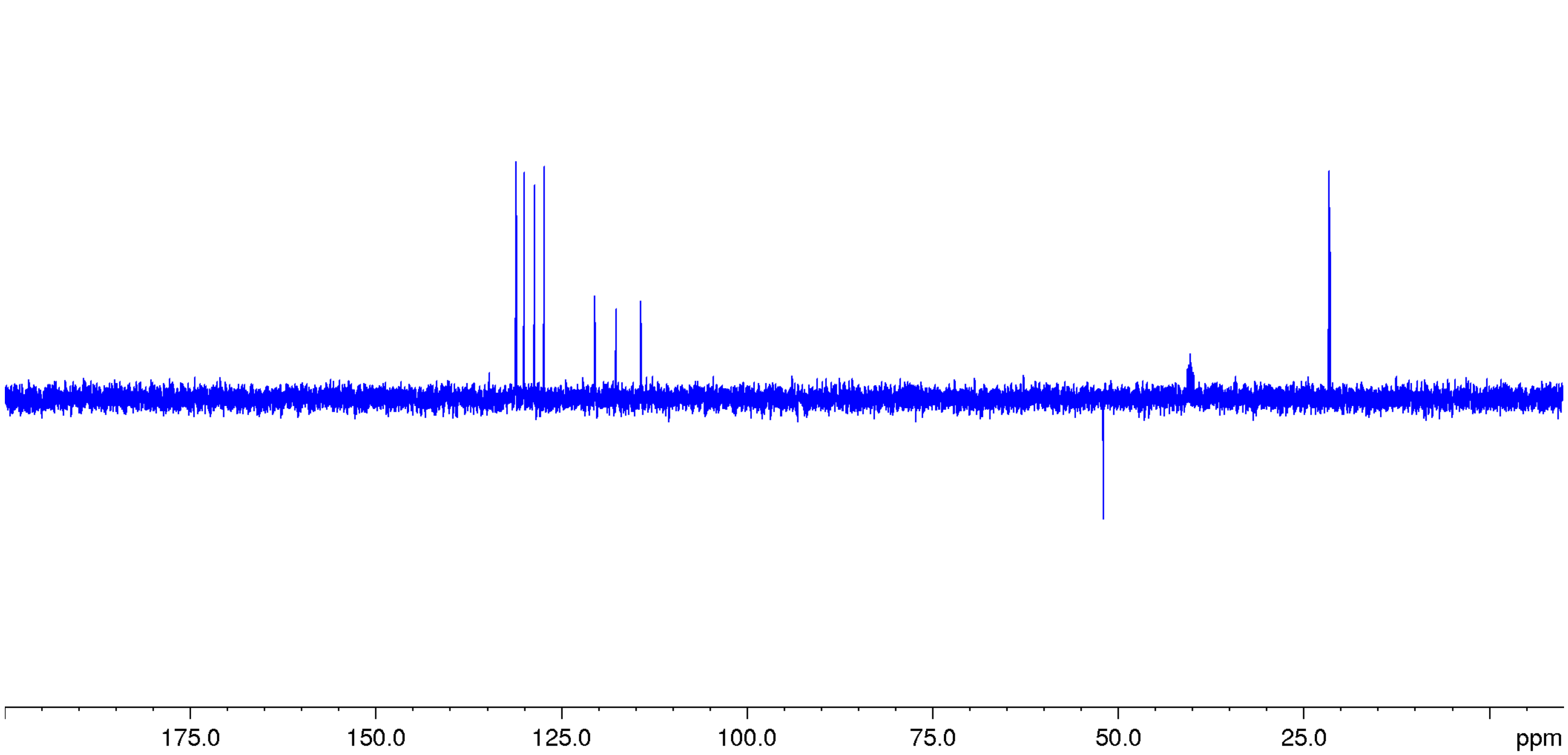

5-nitro-3-((phenylsulfonyl)methyl)-1-tosyl-1H-indole **9gb**

^{1}H NMR-spectrum (400.13 MHz) ($CDCl_3$)

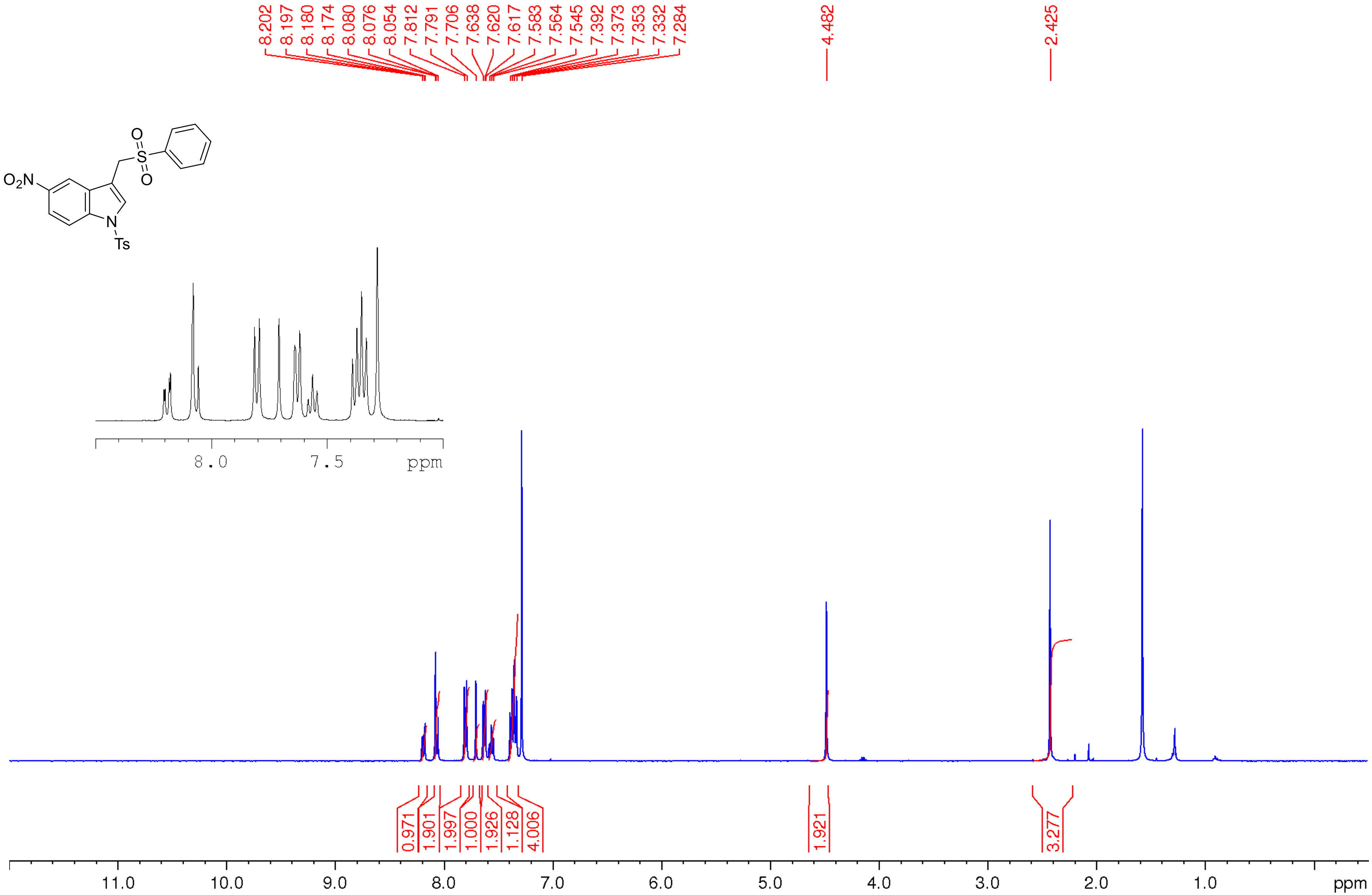

5-nitro-3-((phenylsulfonyl)methyl)-1-tosyl-1H-indole ***9gb***

^{13}C NMR-spectrum (100.6 MHz) ($CDCl_3$)

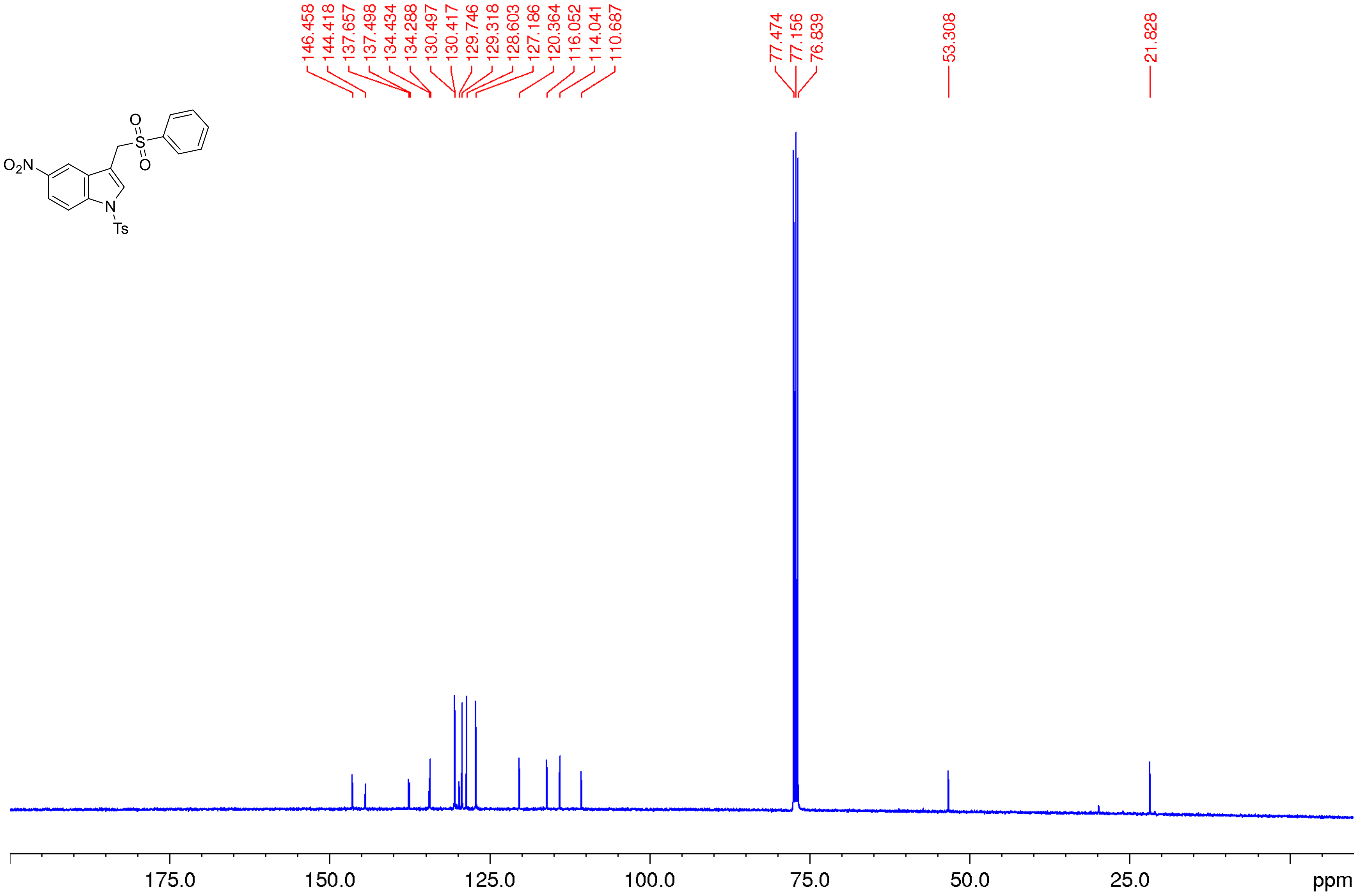

5-nitro-3-((phenylsulfonyl)methyl)-1-tosyl-1H-indole ***9gb***

DEPT 135 NMR-spectrum ($CDCl_3$)

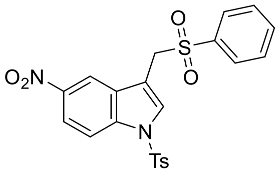

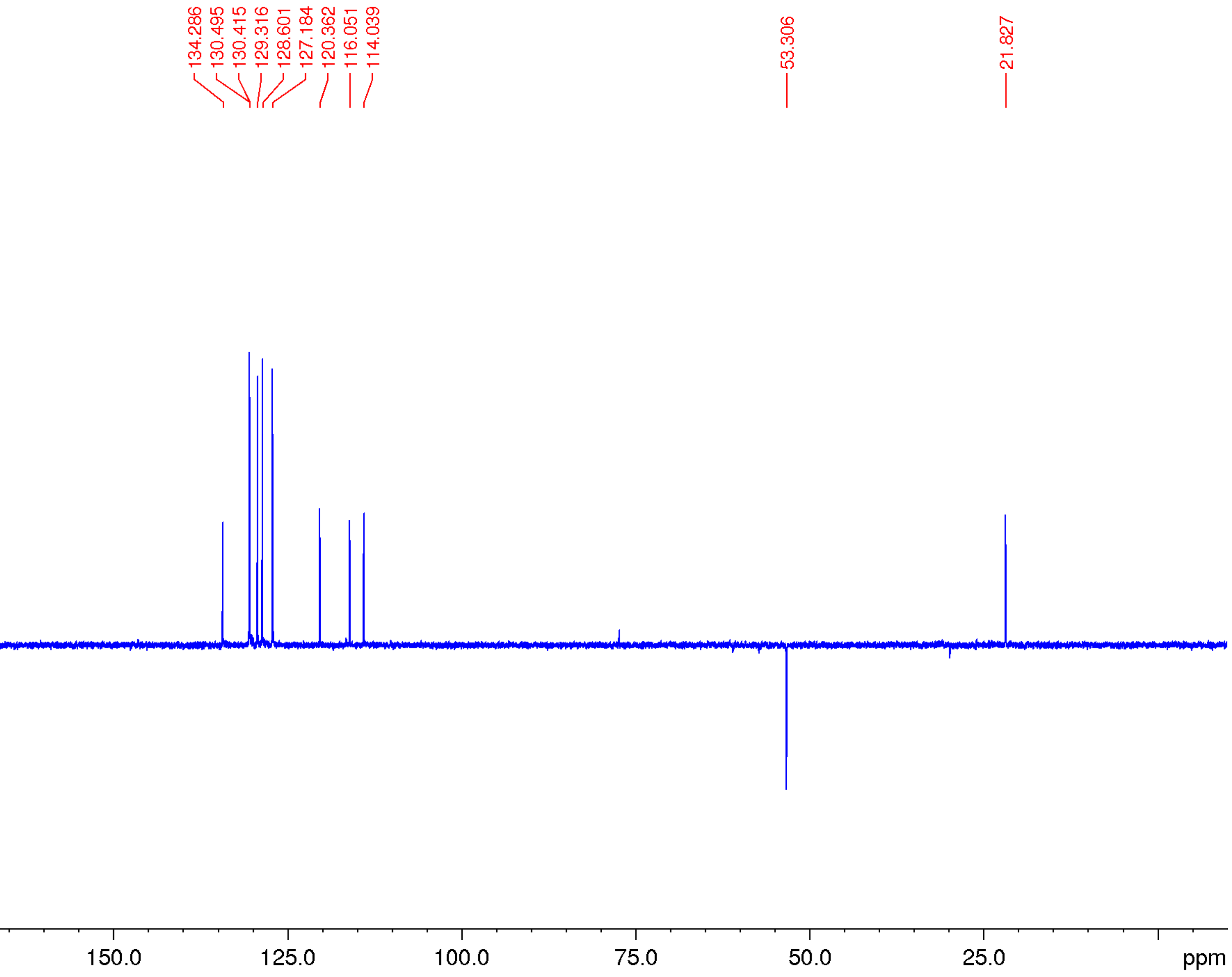

1,1'-ditosyl-3,3'-bis(tosylmethyl)-1H,1'H-6,6'-biindole ***13a***

^{1}H NMR-spectrum (400.13 MHz) ($CDCl_3$)

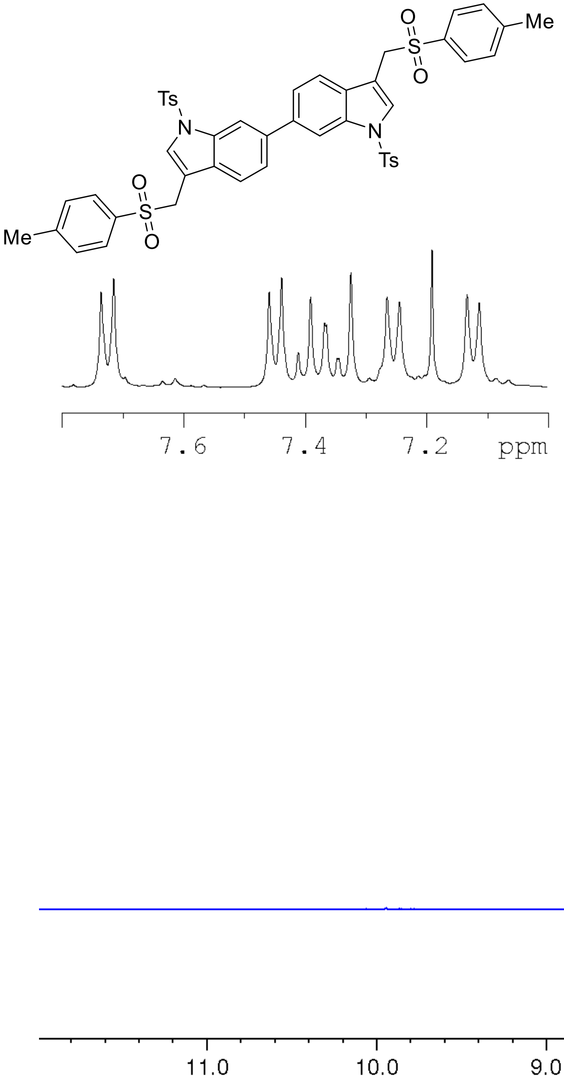

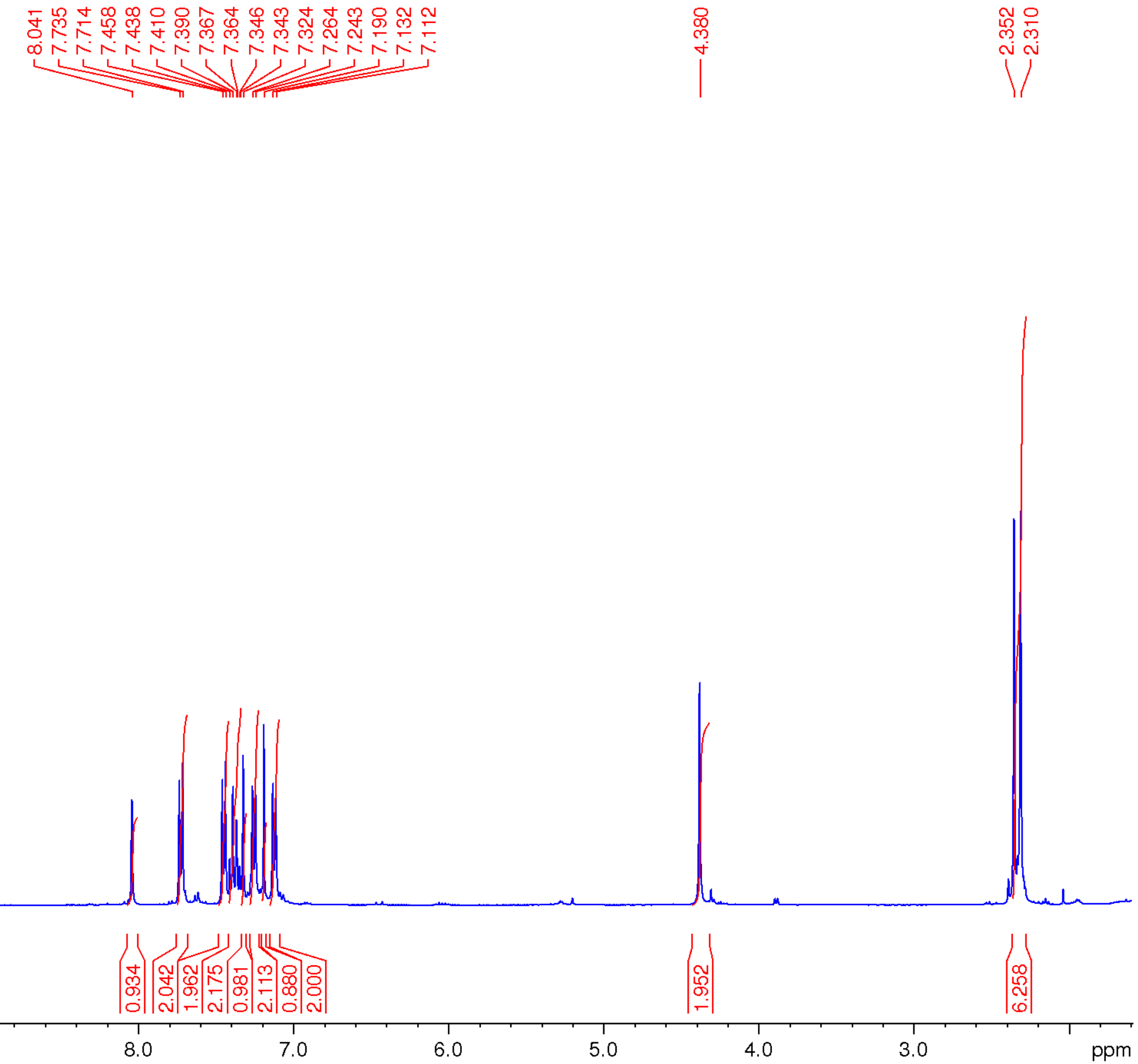

1,1'-ditosyl-3,3'-bis(tosylmethyl)-1H,1'H-6,6'-biindole ***13a***

^{13}C NMR-spectrum (100.6 MHz) ($CDCl_3$)

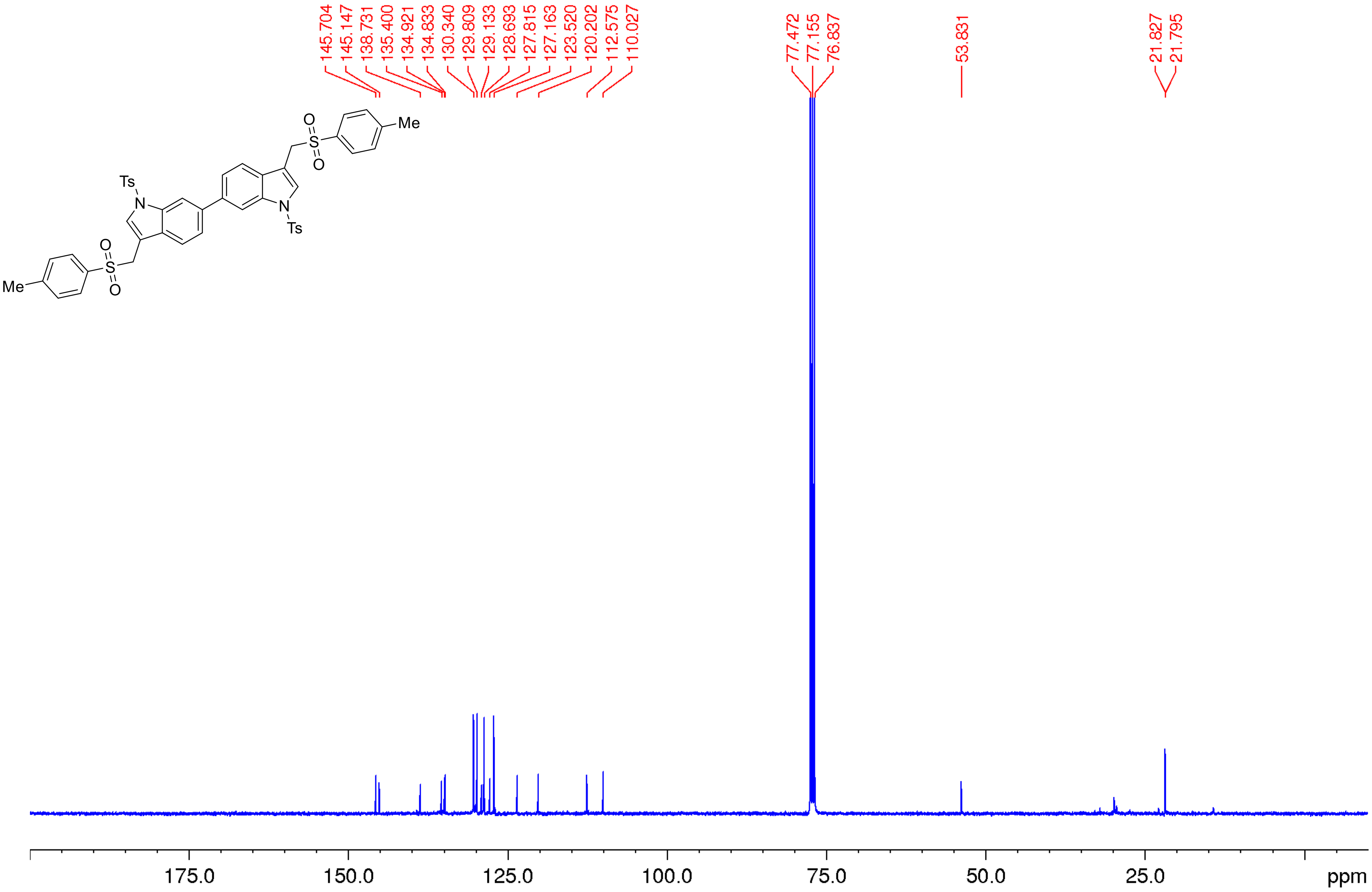

1,1'-ditosyl-3,3'-bis(tosylmethyl)-1H,1'H-6,6'-biindole ***13a***

DEPT 135 NMR-spectrum ($CDCl_3$)

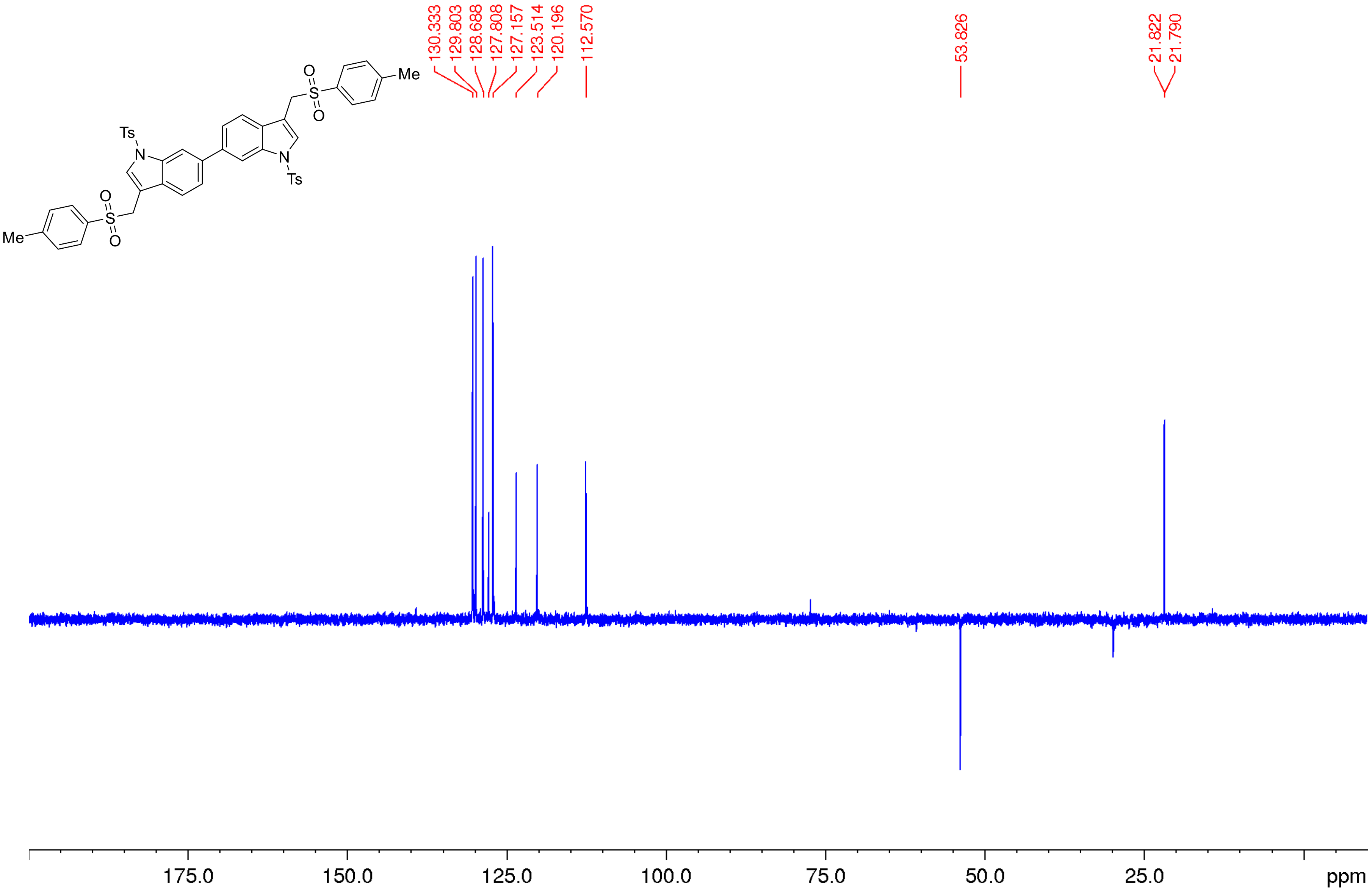

3,3'-bis((phenylsulfonyl)methyl)-1,1'-ditosyl-1H,1'H-6,6'-biindole ***13b*** ^{1}H NMR-spectrum (400.13 MHz) ($CDCl_3$)

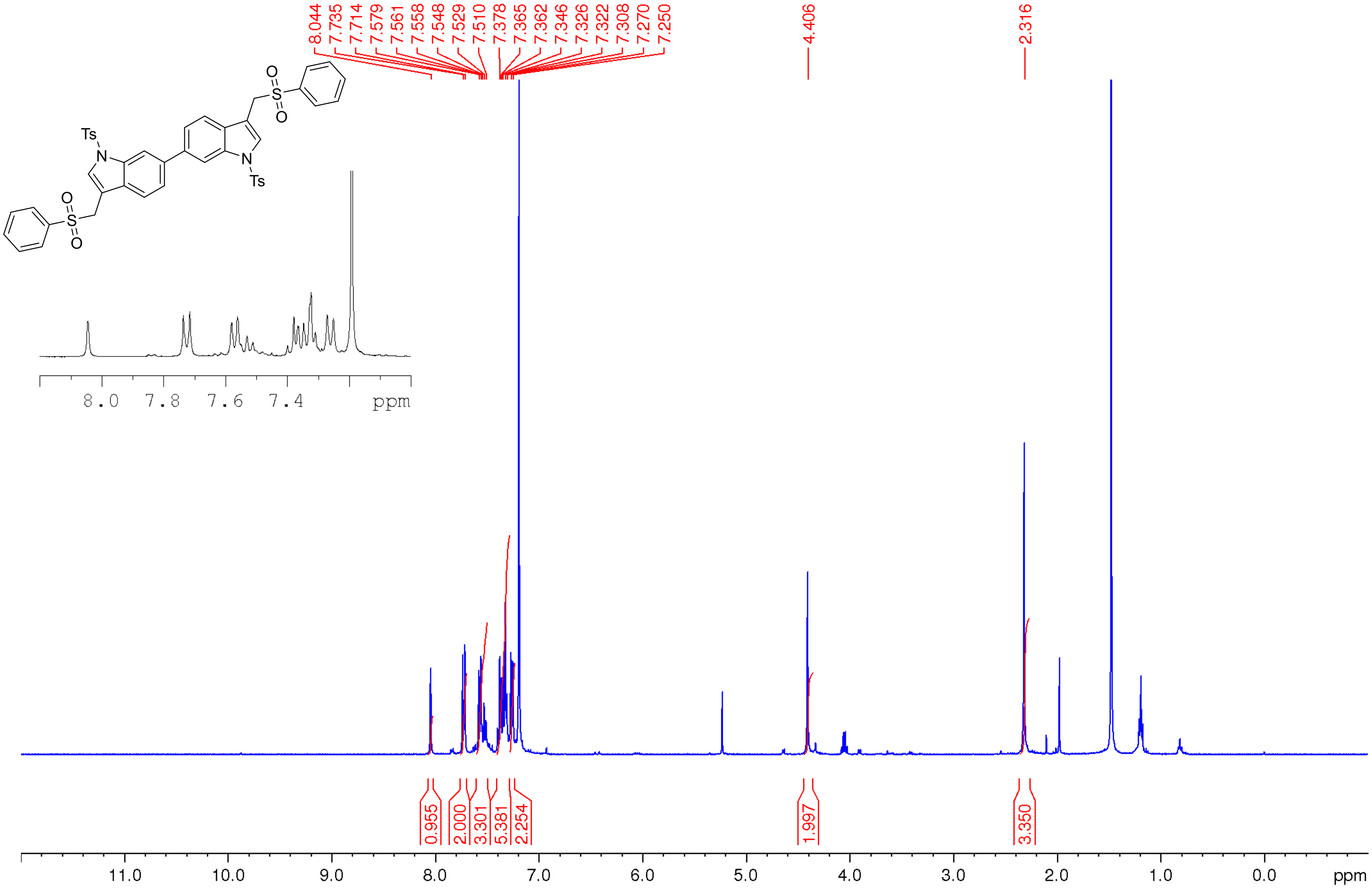

3,3'-bis((phenylsulfonyl)methyl)-1,1'-ditosyl-1H,1'H-6,6'-biindole ***13b*** ^{13}C NMR-spectrum (100.6 MHz) (CDCl$_3$)

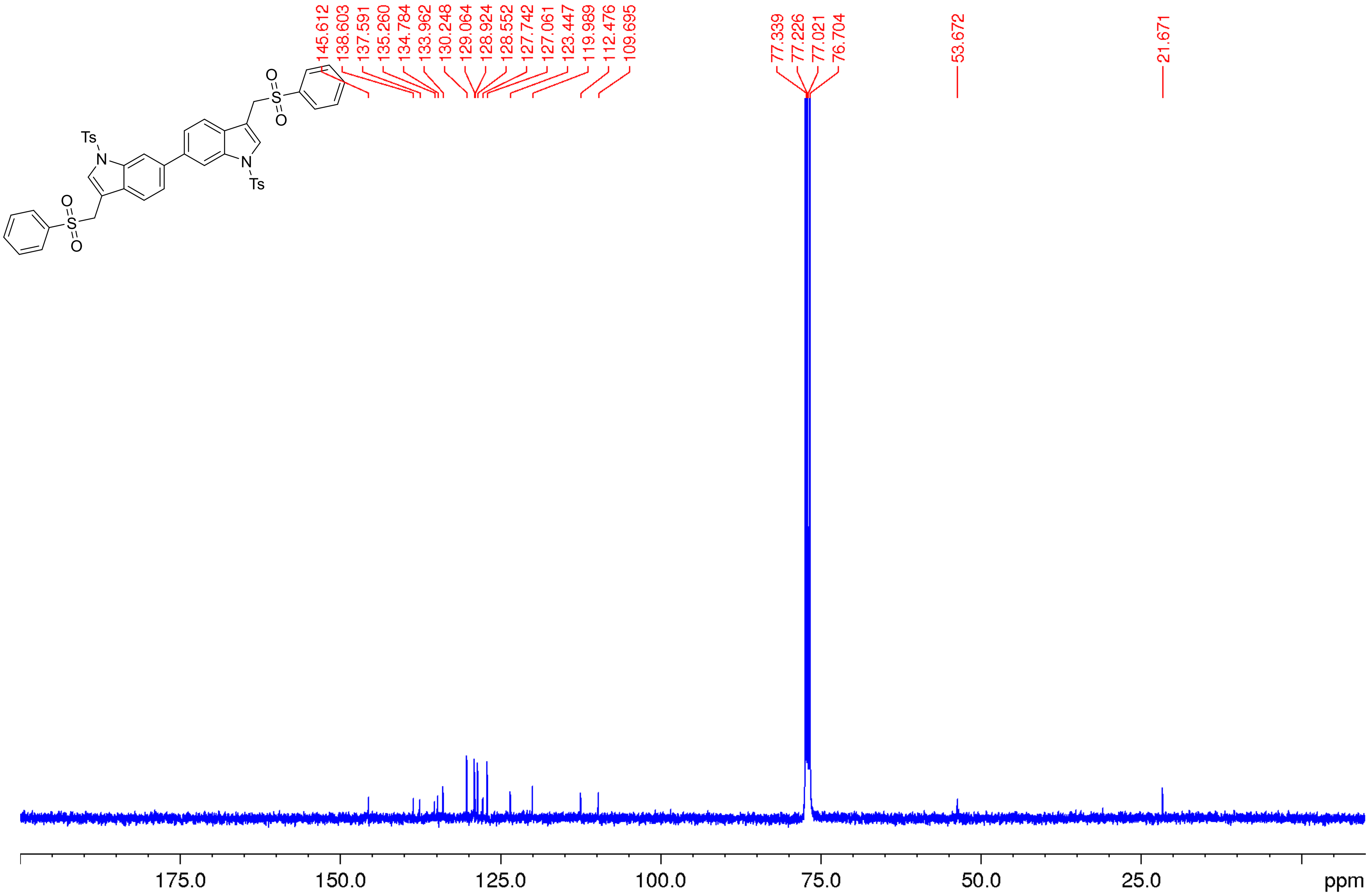

3,3'-bis((phenylsulfonyl)methyl)-1,1'-ditosyl-1H,1'H-6,6'-biindole ***13b*** DEPT 135 NMR-spectrum ($CDCl_3$)

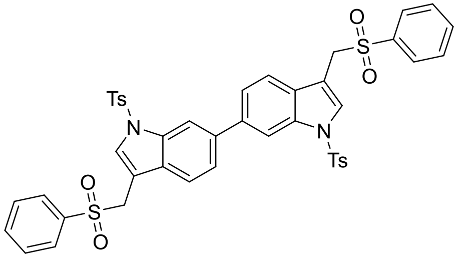

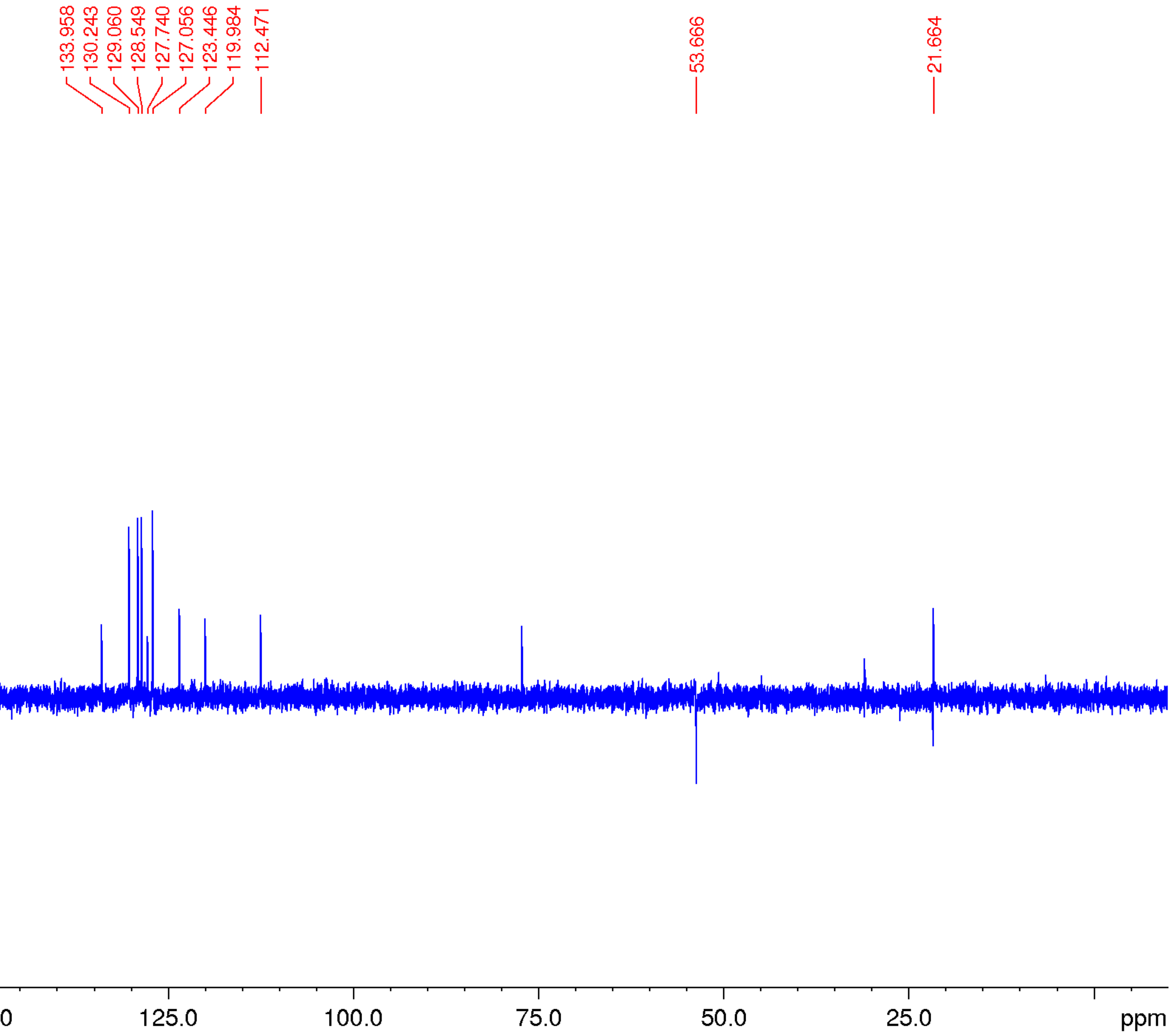

Supplement: Supplementary file 1 [file molecules-29-03434-s001.zip › molecules-3115262-supplementary.pdf]
